# Supplementary material for: Economics of physical activity in low-income and middle- income countries: a systematic review
Source: BMJ Open. 2021 Jan 15;11(1):e037784. doi: 10.1136/bmjopen-2020-037784 (PMC7813307; doi:10.1136/bmjopen-2020-037784)
Supplement: Supplementary data [file bmjopen-2020-037784supp002.pdf]

Search Strategy

Scopus

((TITLE-ABKEY

((*cost* W/2 (*analyses* OR *effectiveness* OR *benefit* OR *minimization* OR *utility* OR *saving* OR *outcome* OR *consequence*))) AND DOCTYPE (*ar* OR *re*)) OR (TITLE-ABS-KEY ((*"demand"* OR *"supply"* OR *"market"* OR *"price"*)) AND DOCTYPE (*ar* OR *re*)) OR (TITLE-ABS-KEY ((*"incentives"* OR *"willingness to pay"* OR *"time use"*)) AND DOCTYPE (*ar* OR *re*)) OR (TITLE-ABS-KEY (*"economics"*) AND DOCTYPE (*ar* OR *re*)) OR (TITLE-ABS-KEY ((*"economic evaluation"* OR *"economic burden"* OR *"health care cost"*)) AND DOCTYPE (*ar* OR *re*))) AND ((TITLE-ABS-KEY (((((*active* W/2 (*lifestyle* OR *living* OR *leisure* OR *play* OR *recreation* OR *commuting*)))))) AND DOCTYPE (*ar* OR *re*)) OR (TITLE-ABS-KEY ((*"physical activity"* OR *"physical exercise"*)) AND DOCTYPE (*ar* OR *re*)) OR (TITLE-ABS-KEY ((*"physical inactivity"* OR *"physical fitness"*)) AND DOCTYPE (*ar* OR *re*)) OR (TITLE-ABS-KEY (*"Sedentary"*) AND DOCTYPE (*ar* OR *re*))) AND (LIMIT-TO (LANGUAGE , *"English"*))

SPORTDiscus

((TITLE-ABS-KEY ((*cost* n/2 (*analyses* OR *effectiveness* OR *benefit* OR *minimization* OR *utility* OR *saving* OR *outcome* OR *consequence*))) AND DOCTYPE (*ar* OR *re*)) OR (TITLE-ABS-KEY ((*"demand"* OR *"supply"* OR *"market"* OR *"price"*)) AND DOCTYPE (*ar* OR *re*)) OR (TITLE-ABS-KEY ((*"incentives"* OR *"willingness to pay"* OR *"time use"*)) AND DOCTYPE (*ar* OR *re*)) OR (TITLE-ABS-KEY (*"economics"*) AND DOCTYPE (*ar* OR *re*)) OR (TITLE-ABS-KEY ((*"economic evaluation"* OR *"economic burden"* OR *"health care cost"*)) AND DOCTYPE (*ar* OR *re*))) AND ((TITLE-ABS-KEY (((((*active* n/2 (*lifestyle* OR *living* OR *leisure* OR *play* OR *recreation* OR *commuting*)))))) AND DOCTYPE (*ar* OR *re*)) OR (TITLE-ABS-KEY ((*"physical activity"* OR *"physical exercise"*)) AND DOCTYPE (*ar* OR *re*)) OR (TITLE-ABS-KEY ((*"physical inactivity"* OR *"physical fitness"*)) AND DOCTYPE (*ar* OR *re*)) OR (TITLE-ABS-

KEY (*"Sedentary"*) AND DOCTYPE (*ar* OR *re*))) AND (LIMIT-TO (LANGUAGE , *"English"*))

Web of Science

((TITLE-ABS-KEY ((*cost* near/15 (*analyses* OR *effectiveness* OR *benefit* OR *minimization* OR *utility* OR *saving* OR *outcome* OR *consequence*))) AND DOCTYPE (*ar* OR *re*)) OR (TITLE-ABS-KEY ((*"demand"* OR *"supply"* OR *"market"* OR *"price"*)) AND DOCTYPE (*ar* OR *re*)) OR (TITLE-ABS-KEY ((*"incentives"* OR *"willingness to pay"* OR *"time use"*)) AND DOCTYPE (*ar* OR *re*)) OR (TITLE-ABS-KEY (*"economics"*) AND DOCTYPE (*ar* OR *re*)) OR (TITLE-ABS-KEY ((*"economic evaluation"* OR *"economic burden"* OR *"health care cost"*)) AND DOCTYPE (*ar* OR *re*))) AND ((TITLE-ABS-KEY (((((*active* near/15 (*lifestyle* OR *living* OR *leisure* OR *play* OR *recreation* OR *commuting*)))))) AND DOCTYPE (*ar* OR *re*)) OR (TITLE-ABS-KEY ((*"physical activity"* OR *"physical exercise"*)) AND DOCTYPE (*ar* OR *re*)) OR (TITLE-ABS-KEY ((*"physical inactivity"* OR *"physical fitness"*)) AND DOCTYPE (*ar* OR *re*)) OR (TITLE-ABS-KEY (*"Sedentary"*) AND DOCTYPE (*ar* OR *re*))) AND (LIMIT-TO (LANGUAGE , *"English"*))
