# Supplementary material for: Economics of physical activity in low-income and middle- income countries: a systematic review
Source: BMJ Open. 2021 Jan 15;11(1):e037784. doi: 10.1136/bmjopen-2020-037784 (PMC7813307; doi:10.1136/bmjopen-2020-037784)
Supplement: Supplementary data [file bmjopen-2020-037784supp003.pdf]

Table 1: List of excluded studies following full text screening with reasons

1. Is Exercise a Good Investment for Adolescents? (1991). Journal of Sport & Exercise Psychology, 13(2), 208-208.	It is a brief one page document and not a research article. country studied also not mentioned
2. Codogno, J. S., Fernandes, R. A., & Monteiro, H. L. (2012). Physical activity and healthcare cost of type 2 diabetic patients seen at basic units of healthcare. [Article]. Arquivos Brasileiros de Endocrinologia e Metabologia, 56(1), 6-11	Article in Portuguese. (though English abstract available)
3. Ng, S. W., & Popkin, B. M. (2012). Time use and physical activity: A shift away from movement across the globe. [Article]. Obesity Reviews, 13(8), 659-680. doi: 10.1111/j.1467-789X.2011.00982.x	It only reports trends in energy expenditure including sedentary time and various physical activities for brazil, India and china and 2 other developed countries. It does not report how time affect the physical activity.
4. Oldridge, N. B. (2008). Economic burden of physical inactivity: healthcare costs associated with cardiovascular disease. European Journal of Cardiovascular Prevention & Rehabilitation, 15(2), 130-139. doi: 10.1097/HJR.0b013e3282f19d42	Though prevalence estimated for developing countries. cost is estimated only for developed countries
5. Kolbe-Alexander, T. L., Conradie, J., & Lambert, E. V. (2013). Clustering of risk factors for non-communicable disease and healthcare expenditure in employees with private health insurance presenting for health risk appraisal: a cross-sectional study. Bmc Public Health, 13. doi: 10.1186/1471-2458-13-1213	Studied clustering of other NCD risk factors with Physical inactivity, and association of clustered risk factors with health care expenditure. Data on expenditure due to physical inactivity not shown separately.
6. Huang, W. Y., & Wong, S. H. (2016). Time use clusters in children and their associations with sociodemographic factors. [Article]. Journal of Public Health (United Kingdom), 38(2), e106-e113. doi: 10.1093/pubmed/fdv088	Identified clusters according to time use for physical activity and sedentary behaviour. It does not assess time effect on physical activity participation.
7. Jim, C. Y., & Chen, W. Y. (2009). Leisure Participation Pattern of Residents in a New Chinese City. Annals of the Association of American Geographers, 99(4), 657-673. doi: 10.1080/00045600903066482	Assessed the association of leisure time and socioeconomic variables. Barriers to leisure participation identified by using a part of the survey questionnaire as a part of this study. HOWEVER, ASSESSED ONLY THE PERCIEVED BARRIERS.
8. Lopes, A. S., Silva, K. S., Filho, V. C. B., Bezerra, J., De Oliveira, E. S. A., & Nahas, M. V. (2014). Trends in screen time on week and weekend days in a representative sample of Southern Brazil students. [Article]. Journal of Public Health (United Kingdom), 36(4), 608-614. doi: 10.1093/pubmed/fdt133	It basically reports the trend of sedentary screen time.
9. Matsudo, V. K. R., Matsudo, S. M., Araújo, T. L., Andrade, D. R., Oliveira, L. C., & Hallal, P. C. (2010). Time Trends in physical activity in the state of São Paulo, Brazil: 2002-2008. [Article].	Reported only the trend in PA participation by socioeconomic status

Medicine and Science in Sports and Exercise, 42(12), 2231-2236. doi: 10.1249/MSS.0b013e3181e1fe8e	
10. Pratt, M., Norris, J., Lobelo, F., Roux, L., & Guijing, W. (2014). The cost of physical inactivity: moving into the 21st century. British Journal of Sports Medicine, 48(3), 171-173.	This is a review
11. Araujo, M. Y. C., et al. (2016). "Association between disability pension, nutritional condition and physical inactivity in adults from a middle-size Brazilian City." Journal of Physical Education (Maringa) 27(1).	Not directly relevant to economics of physical activity as it assesses the association of disability pension and physical activity.
12. Sujatha, T., et al. (2003). "Timed activity studies for assessing the energy expenditure of women from an urban slum in south India." Food and Nutrition Bulletin 24(2): 193-199.	It does not assess the time effect on physical activity
13. Cecchini, M., et al. "Tackling of unhealthy diets, physical inactivity, and obesity: health effects and cost-effectiveness." The Lancet 376(9754): 1775-1784.	This is only a report, proposed a model and analysed cost for interventions for all healthy life styles by intervention type. Not assessed the cost for physical activity promotion by interventions separately or economic evaluation of such intervention
14. Meng, L., et al. (2013). "The Costs and Cost-Effectiveness of a School-Based Comprehensive Intervention Study on Childhood Obesity in China." PLOS ONE 8(10): e77971.	Cost effectiveness assessed only for combined intervention, not for physical activity alone
15. Moraisa. L.C., et al. Health indicators and costs among outpatients according to physical activity level and obesity. *Diabetes & Metabolic Syndrome: Clinical Research & Reviews,* Volume 13, Issue 2, March–April 2019, Pages 1375-137	This has not assessed physical activity separately but coupled with obesity
16. Min, J. Y. and K. B. Min (2016). Excess Medical Care Costs Associated with Physical Inactivity among Korean Adults: Retrospective Cohort Study. *International Journal of Environmental Research and Public Health,* **13**(1).	This study directly does not indicate which part of the Korea. However, the insurance scheme in this study is functioning in South Korea, which is a high-income country.
