# Supplementary material for: Economics of physical activity in low-income and middle- income countries: a systematic review
Source: BMJ Open. 2021 Jan 15;11(1):e037784. doi: 10.1136/bmjopen-2020-037784 (PMC7813307; doi:10.1136/bmjopen-2020-037784)
Supplement: Supplementary data [file bmjopen-2020-037784supp005.pdf]

Summary of studies by country, country category, year of publication and focus

	Study	Country*	Category**	Year of publication	Focus of the study
1	Araujo et al[36]	Brazil	UMC	2017	Association between costs related to productivity loss and modified risk factors among uses of Brazilian National Health System
2	Cheah et al [37]	Malysia	UMC	2017a	Test the hypothesis "income is positively associated with the intensity of physical activity" by using data from a developing country(Malaysia)
3	Ding et al [38]	LMIC	LMIC	2016	Estimate direct health care cost, productivity losses and disability adjusted life years attributable to physical inactivity with standardized methods for 142 countries
4	Mitsunaga et al [39]	Brazil	UMC	2018	Assess the financial impact of a regular physical activity incentive program on medical hospital cost of private health care company
5	Turi et al [40]	Brazil	UMC	2015	Walking and health care expenditure among adults users of Brazilian health care system
6	Cheah et al [41]	Malaysia	UMC	2017b	Factors associated with participation decision and amount of participation among urban dwellers in Malaysia
7	Codongo et al[42]	Brazil	UMC	2015	Association between physical inactivity in different domains and direct public health care expenditure in adults
8	Zhang & Chaban [43]	China	UMC	2012	Total economic burden of physical inactivity in China

9	Turi et al [44]	Brazil	UMC	2017	Identify demographic, socioeconomic , life style and clinical factors associated with direct health care expenditure in primary care among adults through Brazilian health care system
10	Silva A.S [45]	Brazil	UMC	2015	Relationship between sufficient amount of physical activity among Brazilian adolescents and economic and social indicators of cities where they live
11	Patel et al [46]	South Africa	UMC	2011	Participation in fitness related activities of an incentive based health promotion programme and hospital costs
12	Bielmen et al[47]	Brazil	UMC	2014	Physical inactivity related inpatient costs of chronic non communicable diseases
13	Chen et al[48]	China	UMC	2015	Association of socioeconomic status with intensity of different types of physical activity in Chineese adults aiming to outline and projecting socioeconomic disparities among population undergoing rapid nutrient transition
14	Thiago et al[49]	Brazil	UMC	2015	Socioeconomic and regional difference in active transport in Brazil
15	Abdi et al [50]	Iran	UMC	2013	Compare the role of cost, time and attractiveness in predicting the participation level of student of Teheran Universities during their leisure time physical activities
16	Popkin et al[51]	China	UMC	2006	Case study from review of economic costs of diet, physical inactivity and obesity related chronic diseases to provide guidance to utilize the review for economic analysis of obesity
17	Atkinson et al[52]	LMIC	LMIC	2015	Association of occupational category and their physical inactivity and the association of human development, economic development, urbanization and physical inactivity

18	Queiroz et al [53]	Brazil	UMC	2020	Association between quality of life, physical activity, use of medication and costs of treatment for chronic diseases in Primary Care
