# Supplementary material for: Economics of physical activity in low-income and middle- income countries: a systematic review
Source: BMJ Open. 2021 Jan 15;11(1):e037784. doi: 10.1136/bmjopen-2020-037784 (PMC7813307; doi:10.1136/bmjopen-2020-037784)
Supplement: Supplementary data [file bmjopen-2020-037784supp006.pdf]

Quality appraisal of included studies based on standard check lists

1. **Summary of quality appraisal by Larg & Moss Check list for cost of illness studies**

	Ding et al 2016 [38]	Zhang & Chaaban 2013 [43]	Bielemann et al 2015 [47]	Popikin et al 2006 [51]*
Q1(a)	++	++	+	+
Q1(b)	++	+	+	+
Q1(c) i<br>(c) ii<br>(c) iii<br>(c) iv	++<br>+<br>+<br>+(counterfactual population)	+<br>++<br>+<br>NA**	+<br>+<br>+<br>NA	+<br>+<br>NR*<br>NA
Q2(a) i<br>(a)ii<br>(a)iii<br>(a)iv<br>(a)v	-<br>+<br>+<br>+<br>+	-<br>+<br>+<br>-<br>+	-<br>+<br>++<br>+<br>+	-<br>NR<br>NR<br>NR<br>+
Q2(b)i<br>(b)ii<br>(b)iii<br>(b)iv	+<br>NA<br>+<br>+	+<br>NA<br>+<br>+	+<br>NA<br>+<br>+	+<br>NA<br>+<br>-
Q2(c)	+	NR(cost from national data)	NR	NR
Q2(d)	+	NR(cost from national data)	NA	NR
Q2(e)i<br>(e)ii	+<br>++	NR<br>NR	NA<br>NA	NR<br>NR
Q3 (a)	++	++	+	+
Q3(b)	++	-	-	-
Q3 (c)	+	NR	-	NR
Q3(d)i<br>(d)ii<br>(d)iii	+<br>-<br>?	Secondary data<br>Secondary data<br>secondary data	-<br>NR	NR<br>NR<br>NR

			+(CI not reported)NR	
Q3(e)	++	+	+	-
Q3(f)	++	+	-	NR
Q3(g)	++	+	+	+
Q3(h)	++	+	+	+

- Popkin et al case study developed from a review adequate information not available to comment on quality of costing study
- *NR- Not reported
- **NA-Not applicable

2. Summary of quality appraisal by NICE check list for correlations and associations

	Araujo et al 2017[36]	Cheah et al 2017a [37]	Mitsunaga 2018 [39]	Turi et al 2015 [40]	Cheah et al 2017b [41]	Codongo et al 2015 [42]	Turi et al 2017 [44]	Silva A.S 2015 [45]	Patel et al 2011 [46]	Thiago et al 2016 [49]	Abdi et al 2014 [50]	Chen et al 2015 [48]	Atkinson et al 2015 [52]
Q1.1	+	+	+	+	+	++	++	++	+	+	+	+	++
Q1.2	-	+	-	+	+	+	+	+	+	+	+	+	++
Q1.3	+	NR	+	+	+	+	+	+	+	+	-	+	++
Q2.1	NA	NA	+	NA	NA	NA	NA	NA	-	NA	NA	NA	NA
Q2.2	+	+	+	+	+	++	+	+	+	+	+	+	+
Q2.3	NA	NA	+	NA	NA	NA	NA	NA	+	NA	NA	NA	NA
Q2.4	+	+	-	+	+	++	+	-	-	-	-	-	+
Q2.5	This question not applicable for this review												
Q3.1	+	-	+	+	-	+	+	+	+	-	+	+	+
Q3.2	+	+	+	+	+	+	+	+	+	+	+	+	+
Q3.3	+	+	+	+	+	+	+	+	+	+	+	+	+
Q3.4	NA	NA	+	NA	NA	NA	NA	NA	+	NA	NA	NA	NA
Q3.5	NA	NA	+	NA	NA	NA	NA	NA	+	NA	NA	NA	NA
Q4.1*	+	+	-	+	+	+	+	+	+	-	+	+	+

Q4.2	+	+	-	+	+	++	+	-	+	-	-	+	+
Q4.3	+	+	+	+	+	++	+	+	+	-	-	+	+
Q4.4	+	+	-	+	+	++	+	+	+	-	-	+	+
Q5.1(IV)	+	+	++	++	++	++	++	++	++	++	+	++	++
Q5.2(EV)	++	++	++	++	++	++	++	++	++	+	+	++	++
Score	(13/13) (2/3),	(12/13) (2/3)	(9/13) (2/3)	(13/13) (3/3)	(12/13) (3/3)	(13/13) (3/3)	(13/13) (3/3)	(11/13) (3/3)	(11/13) (3/3)	(9/13) (3/3)	(9/13) (2/3)	(13/13) (3/3)	(13/13) (3/3)

*Most of the studies power calculations not done. However, large samples have been used

Quality score
Internal Validity >= 10/13=++ (>75%)
6-9/13=+ (50-75%)
0-5/13=- (<50%)

External Validity =3/3 (++),
= 2/3(+)
=≤1/3(-)

3. Summary of quality appraisal by Drummond and Jefferson 1996- check list for economic evaluation studies

	Queiroz et al 2020[53]
Study design	
Q1	+
Q2	+
Q3	+
Q4	+

	Queiroz et al 2020[53]
Q5	+
Q6	+
Q7	-
Data collection	
Q8	+
Q 9	-
Q 10	NA
Q11	+
Q12	+
Q13	+
Q14	NA
Q 15	NA
Q 16	NC
Q 17	-
Q 18	+
Q 19	-
Q 20	-
Q 21	-
Analysis and interpretation of results	
Q 22	+
Q 23	-
Q 24	-
Q 25	-
Q 26	NC
Q 27	-
Q 28	-
Q 29	-
Q 30	+
Q 31	+

	Queiroz et al 2020[53]
Q 32	NC
Q 33	+
Q 34	+
Q 35	+

Did the economic evaluation use a decision-analytic modelling framework?	No
Overall assessment by Drummond and Jefferson checklist	
Yes +<br>No -<br>Not clear NC<br>Not applicable NA	23 /35<br>(65.7%)
