# Supplementary material for: Economics of physical activity in low-income and middle- income countries: a systematic review
Source: BMJ Open. 2021 Jan 15;11(1):e037784. doi: 10.1136/bmjopen-2020-037784 (PMC7813307; doi:10.1136/bmjopen-2020-037784)
Supplement: Author's manuscript [file bmjopen-2020-037784.draft_revisions.pdf]

BMJ Open is committed to open peer review. As part of this commitment we make the peer review history of every article we publish publicly available.

When an article is published we post the peer reviewers' comments and the authors' responses online. We also post the versions of the paper that were used during peer review. These are the versions that the peer review comments apply to.

The versions of the paper that follow are the versions that were submitted during the peer review process. They are not the versions of record or the final published versions. They should not be cited or distributed as the published version of this manuscript.

BMJ Open is an open access journal and the full, final, typeset and author-corrected version of record of the manuscript is available on our site with no access controls, subscription charges or pay-per-view fees (http://bmjopen.bmj.com).

If you have any questions on BMJ Open's open peer review process please email info.bmjopen@bmj.com

The economics of physical activity in low and middle income countries: systematic review

Journal:	*BMJ Open*
Manuscript ID	bmjopen-2020-037784
Article Type:	Original research
Date Submitted by the Author:	16-Feb-2020

Complete List of Authors:	Ranasinghe, Priyanga; Ministry of Health, Nutrition and Indigenous Medicine, ; Brunel Univeristy London, Clinical Sciences<br>Pokhrel, Subhash; Brunel University London, Health Economics Research Group<br>Anokye, Nana; Brunel University, Health Economics Research Group

Keywords:	HEALTH ECONOMICS, PUBLIC HEALTH, SPORTS MEDICINE

SCHOLARONE™
Manuscripts

I, the Submitting Author has the right to grant and does grant on behalf of all authors of the Work (as defined in the below author licence), an exclusive licence and/or a non-exclusive licence for contributions from authors who are: i) UK Crown employees; ii) where BMJ has agreed a CC-BY licence shall apply, and/or iii) in accordance with the terms applicable for US Federal Government officers or employees acting as part of their official duties; on a worldwide, perpetual, irrevocable, royalty-free basis to BMJ Publishing Group Ltd ("BMJ") its licensees and where the relevant Journal is co-owned by BMJ to the co-owners of the Journal, to publish the Work in this journal and any other BMJ products and to exploit all rights, as set out in our licence.

The Submitting Author accepts and understands that any supply made under these terms is made by BMJ to the Submitting Author unless you are acting as an employee on behalf of your employer or a postgraduate student of an affiliated institution which is paying any applicable article publishing charge ("APC") for Open Access articles. Where the Submitting Author wishes to make the Work available on an Open Access basis (and intends to pay the relevant APC), the terms of reuse of such Open Access shall be governed by a Creative Commons licence – details of these licences and which Creative Commons licence will apply to this Work are set out in our licence referred to above.

Other than as permitted in any relevant BMJ Author's Self Archiving Policies, I confirm this Work has not been accepted for publication elsewhere, is not being considered for publication elsewhere and does not duplicate material already published. I confirm all authors consent to publication of this Work and authorise the granting of this licence.

For peer review only

Title page

The economics of physical activity in low and middle income countries: systematic review

Priyanga Diloshini Ranasinghe [2], Subhash Pokhrel[1], Nana Kwame Anokye[1]

[1] Health Economics Theme, Institute of Environment, Health and Societies, Brunel University, London, United Kingdom

[2] Ministry of Health, Nutrition and Indigenous Medicine, Suwasiripaya, Colombo 10, Sri Lanka /Division of Health Sciences, Department of Clinical Sciences, Collage of Health and Life Sciences, Brunel University, London, United Kingdom

Corresponding author: Priyanga Diloshini Ranasinghe

Postal address: Mary Seacole 2nd floor, Division of Health Sciences, Department of Clinical Sciences, Collage of Health and Life Sciences, Brunel University, London, United Kingdom

Email: priyangaran@yahoo.com/ Priyanga.Ranasinghe@brunel.ac.uk

Telephone number: 0094 777552794/0044 7424515136

Subhash Pokhrel Subhash.Pokhrel@brunel.ac.uk

Nana Kwame Anokye Nana.Anokye@brunel.ac.uk

Keywords: low and middle income countries; health economics; physical activity

Word count:4764(including abstract and summary)

For peer review only - http://bmjopen.bmj.com/site/about/guidelines.xhtml

Abstract

Background: Physical inactivity is a global health challenge. However, evidence on economic costs of physical inactivity, how to mitigate those and the cost effectiveness of doing so, are sparse and scattered in low and middle income countries (LMIC).

Objective: To summarise available evidence on economics of physical activity in LMICs, identify potential target variables for policy, and report gaps in the existing economic evidence alongside research recommendations.

Methods/Design: A systematic review of the electronic databases (Scopus, Web of Science and SPORTDiscuss) and grey literature (websites of World health organization (WHO), NICE international, World Bank) were conducted. All results were screened by titles to remove obviously irrelevant studies and duplications independently by two reviewers. Risk of bias and quality of selected articles were appraised using standard quality assessment checklists by one reviewer and second reviewer independently assessed randomly selected 50% of included studies. Descriptive synthesis of data performed.

Results: Among 17 studies included, 7 were conducted in Brazil. Economic studies were identified under three main themes; cost of physical inactivity, association of physical (in)activity and cost, association of socioeconomic factors and physical inactivity. None of the studies focused on economic evaluations. LMIC have larger proportion of disease burden (75% DALYs attributable to physical inactivity) while high income countries bear the major share of economic burden.

Conclusions: Marked gap in research on economic evaluations for physical activity promotion interventions in LMIC. Economic research agenda in LMIC recommended to be focused on generating evidence on cost effective interventions and strategies to promote physical

For peer review only - http://bmjopen.bmj.com/site/about/guidelines.xhtml

activity in LMIC.

PROSPERO registration number: CRD42018099856.

Strengths and limitations of this study

- This is the first synthesis of literature on economics of physical activity in low-income and middle-income countries (LMICs).
- This study provides evidence-based recommendations for economic research practice on physical activity.
- This review could suffer from publication bias as it excludes studies that are not peer reviewed.
- This review focuses on studies written in the English language and could miss out on relevant literature published in other languages.
- This review not conducted meta-analysis due to wide variation in focus and methods of included studies

For peer review only - http://bmjopen.bmj.com/site/about/guidelines.xhtml

Introduction

Globally, non-communicable diseases (NCD) cause deaths than all other causes of mortality combined and NCD deaths projected to be increased from 38 million in 2012 to 52 million by 2030 [1]. NCD is a major global health challenge and physical inactivity is estimated to cause a large share of main NCDs; approximately around 30% of ischemic heart diseases burden, 27% of diabetes and 21-25% of breast cancer and colon cancer burden [2]. Insufficient physical activity account for 3.2 million deaths annually and in 2010, 69.3 million DALYs in worldwide [1] and is the fourth leading cause of global mortality accounting for 6% of deaths worldwide [2]. Currently, LMIC account for most of the NCD burden worldwide [3]. WHO reports that NCD disproportionately affect people in LMIC where 31 million NCD deaths occur, which is more than three quarter of global NCD deaths [4]. Fifteen million of all NCD deaths globally have occurred between ages of 30-69 years and more than eighty percent of these premature deaths are reported from LMIC [4]. Insufficient physical activity is on increasing trend in LMIC [5] and 1.6 million of global NCD deaths are attributed to this modifiable risk factor; insufficient physical activity [6]. One in four adults are not sufficiently active globally, while 80% of world`s adolescent population is insufficiently physically active [7]. Prevalence of insufficient physical activity among adults in 2010 by world bank income groups; 16.6% in low income countries, 16.8% in lower middle income countries, 25.4% in upper middle income countries [8].

However, physical inactivity is described as "Cinderella of NCD risk factors, defined as poverty of policy attention and resourcing, proportionate to its importance" [9]. It is neither highlighted as a problem in LMIC nor used in advocacy and communication to make the case

For peer review only - http://bmjopen.bmj.com/site/about/guidelines.xhtml

strongly and convincingly for physical activity [9]. Thus, it is of paramount importance to advocate the policy makers, decision makers including political authority's on "economic case" of physical activity promotion, to reveal why this is a worth investment[10]. Furthermore, economic analysis is essential for political engagement and motivation, to bridge gap between policy and its implementation [11], especially in LMIC, facing unlimited competing health needs due to double burden of disease with finite resources. Peer reviewed literature is identified as a good way to inform the budget setting priorities [10]. There is an unmet demand for an evidence base on economics of physical activity in LMIC which can be used as an advocacy tool to prioritize physical activity promotion. Enhancing the understanding of physical activity linking with its economic facets in LMIC could be an important step forward to draw the attention of politicians and policymakers for implementation of physical activity promotion as a National priority. Also, such information will facilitate the efficient allocation of resources.

The aim of this review was to (i) Summarize available evidence on economics of physical activity in LMIC (ii) Describe the focus and methods underpinning research on economics of physical activity in LMIC (iii) Identify potential target variables and cost effective interventions in LMIC for policy formation with regard to physical activity promotion (iv) Identify and report gaps in research on economics of physical activity in LMIC and provide recommendations for economic research agenda in LMIC.

Methods

Study protocol with detailed methods for this review was published prior to the commencement of this review (12).

For peer review only - http://bmjopen.bmj.com/site/about/guidelines.xhtml

Search strategy

Systematic literature search was conducted to identify studies on economics of physical activity in LMIC on the following data bases; Scopus (covers 100% of MEDLINE coverage, 100% of EMBASE coverage, 100% of Compendex coverage [13]), Web of Science and SPORTDiscus. Websites of World health organization (WHO), NICE international, World Bank and reference lists of included studies were searched for any relevant studies. Search included articles from the beginning of records to December 2017 in line with study protocol. The search strategy was informed by a scoping review that covered relevant reviews on economics studies on physical activity [14-20], reviews on physical activity [21-23] and reviews on economic evaluations [24-26]. Final search strategy including search terms for each data base is given in supplementary file 1. Same search terms were used on all three data bases.

Eligibility criteria and screening

All results were screened by titles to remove obviously irrelevant studies and duplications independently by two reviewers (PDR, NKA). Disagreements were discussed with a third reviewer (SP) Remaining studies were further screened by titles and abstracts to include specifically following study types; economic evaluations of physical activity interventions, economic burden of physical inactivity, cost of physical activity participation, demand for physical activity and economic correlates of physical (in)activity. Studies were included if the following criteria were met; (i) Study setting: Any setting of LMIC in accordance with the definition of LMIC by World Bank classification 2017[27] (ii) Population: any age group across life course (iii) Interventions: any physical activity intervention in which the economic evaluation of the intervention has been carried out (iv) Comparator: normal routine, no intervention (intervention and comparator applicable only for intervention studies) (v)

For peer review only - http://bmjopen.bmj.com/site/about/guidelines.xhtml

Outcome: (a) cost effectiveness ratio(CE ratio), QALY, incremental cost effectiveness ratio (ICER) assessed as the outcome of physical activity intervention, (b) cost of physical (in) activity in terms of health care cost and/or productivity loss and/or total cost of physical inactivity (c) measures of association of any economic variable with physical activity are defined as the primary outcomes of this review (vi) Study design: Observational studies (cohort, case control, cross sectional), experimental studies including randomized control trials(RCT),quasi experimental studies including natural experiments and economic evaluation studies (vii) Studies reported in English language

Case reports, case series, letters to the editor, editorials, reviews, qualitative studies, unpublished thesis, conference abstracts and any unobtainable texts were excluded. Studies that did not report cost, economic evaluations, and economic correlates of physical activity, separately from other lifestyle risk factors, were excluded from the review by consensus of all three reviewers. List of excluded studies with reasons given in supplementary file 2.

Data extraction

Data on following main items; general information, characteristics of the study, characteristics of population/intervention/comparator/outcome/study design(PICOS), data sources, analysis, results, conclusions and way forward, challenges were extracted using a word template. Data extraction form was adapted from data extraction forms from relevant reviews [18, 28, 29]. Full data extraction form given in supplementary 3. Data from included studies were extracted by one reviewer (PDR). Quality of data extraction was ensured by

For peer review only - http://bmjopen.bmj.com/site/about/guidelines.xhtml

extraction of data independently by a second reviewer (NKA) in randomly selected 50% (9/17) of included studies.

Risk of bias and quality appraisal

Larg and Moss checklist [30], Check list by National Institute for Health Care Excellence (NICE) [31] were used to assess risk of bias of cost of illness studies and studies on correlations or associations respectively. Drummond check list [32] and Phillips [33] check lists were planned to be used for studies on economic evaluations. However, there weren`t any eligible studies retrieved on economic evaluations related to physical activity in LMIC for this review. One reviewer (PR) assessed the risk of bias using these standard tools for quality appraisal. Second reviewer independently assessed randomly selected 50% (9/17) of included studies. Full agreement on quality appraisal between two reviewers observed. Overall quality grading was allocated to both internal validity and external validity separately based on fulfilment of check list criteria. Overall quality grading was allocated using NICE recommendations [31] irrespective of checklist being used and based on fulfilment of checklist criteria; All or most (>75%) fulfilled (++), some (50%-75%) fulfilled (+), none or few (<50%) fulfilled (-). Percentages of fulfilment of check list criteria as cut-offs was set with full agreement of all three reviewers prior to commencement of the study. Internal validity was appraised based on section 2-4 and external validity by section 1 of NICE check list [30] for studies on correlations or associations. External validity appraised by Q2b (III) and internal validity by rest of the questions in Larg and Moss checklist [29] for studies on cost of illness.

For peer review only - http://bmjopen.bmj.com/site/about/guidelines.xhtml

Data synthesis

Descriptive synthesis of data was performed in order to describe the methods, operationalization of methods, major limitations, quality, suggestions and recommendations for future research. Data from research evidence on economics of physical activity in LMIC was used to prepare summary tables. Identification of potential target variables for policy and the gaps in research on economic of physical activity was focused from descriptive analysis of available evidence.

Results

Search outcomes

Systematic literature search retrieved 16735 records. After removing duplicates and obviously irrelevant records by screening of titles, 81 articles were selected to screen abstracts. Thirty one records were assessed for eligibility by full text screening. Seventeen articles were selected for data extraction. Flow diagram of this process illustrated in figure 1.

Overview of studies

Out of 17 studies; Brazil (n=7, 41%), China (n=3, 18%), Malaysia (n=2, 12%), Korea, South Africa and Iran contributed with one study from each country (n=1, 6% from each) and two global analysis (n=2, 12%) (Figure 2). Brazil predominates in the economic evidence base on physical activity in LMIC. Out of 17 studies, fourteen were (82%, n=14) conducted in upper middle income countries [34-35, 38-49]. All studies were published after 2012. List of

For peer review only - http://bmjopen.bmj.com/site/about/guidelines.xhtml

For peer review only

included studies by the focus of the study is given in supplementary file 4. Four studies were focused on economic burden of physical inactivity [36,41,45,49] (n=4, 24%), six studies have assessed association of physical inactivity and direct or indirect cost [34,37,38,40,42,44] (n=6, 35%) and seven studies have assessed the association of physical (in) activity and economic factors [35,39,43,,46,47,48,50] (n=7, 41%). Economic evidence base on physical activity in LMIC is mainly consisting of research on socioeconomic factors associated with physical activity. Studies on economic evaluations related to physical activity in LMIC were not identified by this review.

Methods underpinning the economic evidence base on physical activity in LMIC

The summary of studies by methods is given in table 1. Majority of the studies have used cross sectional design (n=13/17,76.5%)(34,35,36,38,39,40,41,42,45,46,47,49,50). Four studies have estimated the economic burden of physical inactivity [36, 41, 45, 49]. Both direct and indirect costs were considered in two out of those four studies [36, 41]. DALYs attributable to physical inactivity was estimated only in one of those studies [36]. Similar methodology was applied in other three studies using population attributable fraction (based on prevalence of inactivity, relative risk values (RR), cost of main NCDs related to physical inactivity) [36, 41, 49]. Estimates for RR were obtained from developed countries due to unavailability of data for LMIC. One study has assessed only the physical inactivity related inpatient costs of chronic non communicable diseases [45]. All four studies have used secondary data to estimate the cost of inactivity while prevalence data on inactivity obtained from population surveys or GBD study. There weren`t any study conducted on economic evaluations related to physical activity in LMIC.

For peer review only - http://bmjopen.bmj.com/site/about/guidelines.xhtml

For peer review only

Majority of studies on economics of physical activity in LMIC, have focused on economic correlates of physical activity and assessed; the association of physical (in) activity and direct health cost [37, 38, 40, 42, 44], association of physical (in) activity and productivity lost [34], socioeconomic factors associated with physical activity participation and intensity [35, 39, 43, 46, 47, 48, 50]. Regression analysis was used for the final analysis to establish the association of physical inactivity and direct health care cost or productivity loss [34, 37, 38, 40, 42, 44]. Studies on association and correlation have used different models, statistical tests based on the nature of the study and the type of data [35, 39, 43, 46, 47, 48,50]. A summary of included studies by PICOS, is shown in table 2.

Method of operationalization of physical activity have varied among the studies. This is shown in Table 3. Only one study [36] has used the recommendation of WHO; 150min of physical activity per week to operationalized as physically active while one study has used more strict definition[43]; 300min of being active during last 7 days as the cut off. Baecke questionnaire was the method of choice to define physical activity among Brazilian studies [34, 38, 40, 42]. However, all physical activity data were based on self-report of physical activity level.

Among these studies in LMIC the cost was measured either as direct health care cost or indirect cost by productivity loss. Direct health care cost was operationalized as cost for medications, laboratory tests and medical consultations in Brazilian studies [38, 40, 42] and/or inpatient cost [44] and/or outpatient cost, prescription cost [36] as shown in table 3. Indirect cost was operationalized as loss of productivity due to premature mortality [36] or due to absenteeism and disability retirement [34].

For peer review only - http://bmjopen.bmj.com/site/about/guidelines.xhtml

Quality appraisal of included studies

Majority of studies fulfilled most of the check list criteria of the relevant quality assessment checklist (12/17, 70.6%), while four studies fulfilled some of the criteria, where they haven't fulfilled, conclusions were unlikely to alter (4/17, 23.5%) for internal validity. The information on methods was not adequate to report the quality of one study as it was a case study based on a review [48].

Similarly external validity was good in all studies enabling results to be generalizable. Though, most of the studies have not done power calculation or sample size calculation, have used large samples from population surveys. Table 4 illustrates the summary of quality appraisal of included studies and supplementary file 5 details the results of quality appraisal by Larg and Moss check list [36, 41, 45, 49] and NICE check list for studies on correlations and association [34, 35, 37, 38,39, 40, 42, 43, 44, 46 , 47, 48, 50]

Evidence on economics and physical activity in LMIC

Economic burden of physical inactivity in LMIC, association of physical inactivity and cost, socioeconomic factors associated with physical activity are illustrated in table 5.

Economic burden of physical inactivity in LMIC

Direct cost

The direct health care cost in LMIC attributable to physical inactivity has been 10.3 billion INT$ in 2013 which is 19.1% of direct health care cost attributable to physical inactivity worldwide. Upper middle income countries have contributed to 86% of this economic burden [36].

For peer review only - http://bmjopen.bmj.com/site/about/guidelines.xhtml

For peer review only

Estimated direct health cost in China attributed to physical inactivity in 2007 was 3.5 billion USD[41] while Brazil estimated the cost of hospital admissions in 2013 as 0.73 billion USD by local studies[45]. Estimated direct health care cost attributed to physical inactivity by global analysis was 3.1 billion INT$ for China and 1.6 billion INT$ for Brazil [36]. Inpatient cost and outpatient cost attributed to physical inactivity in 2000 based on a Chinese case study from a review was 0.4 billion USD and 0.9 billion USD in China [49].

Indirect cost and DALYs

Physical inactivity related deaths contribute to productivity loss of 5.3 billion INT$ which is 38.7% of productivity loss due to deaths related to physical activity worldwide [36]. LMIC bare 10.1 million DALYs attributed to physical inactivity which is 75% DALYs worldwide [36]. Indirect cost of physical inactivity in China was estimated 3.3 billion USD in 2007 [41]. Estimated indirect cost due to productivity loss by deaths related to physical inactivity in China was reported by a global analysis study as 1.78 billion INT$.

Economic correlates of physical inactivity

Studies have assessed the association of physical (in) activity and cost in LMIC and economic factors associated with physical activity in LMIC.

Association of physical (in) activity and health care cost in LMIC

Korea reported 11.7% increase in direct health care cost for physical inactive people compared to active people ($p < 0.001$) [37]. Brazil reported Walking (always) during leisure time is associated with lower health care expenditure in primary care (OR_{adj} 0.59, 0.39-0.89) [38]. Higher health care expenditure of medicine was associated with lower physical activity at work (OR 1.58 (1.06-1.35) and sport (OR1.57 (1.12-2.18) in Brazil [40]. Further, expenditure

For peer review only - http://bmjopen.bmj.com/site/about/guidelines.xhtml

related to medicine(r= 0.109, 95%CI 0.046- 0.171) and overall expenditure (r= 0.092, 95%CI 0.046-0.171) was significantly associated with physical inactivity in Brazil [40]. Brazil further revealed that expenditure on medication is associated with moderate physical activity (OR 0.56, 0.38-0.81) [42]. They concluded that physical inactivity as an independent risk factor associated with higher health care expenditure in relation to medication use among adults who lived in Bauru a medium sized city in the state of Sao Paulo. South African a retrospective longitudinal study showed that the odds of hospital admissions was 13% lower for two additional gym visits per week among participants of incentive based fitness related activity programme [44]. Members who were not on the health promotion programme had a significantly higher probability of hospital admissions (p<0.01) and higher claims than participants who change their physical activity status to "active to no change" group or "active to more active group" [44].

Studies conducted in Korea [37], Brazil [38, 40, 42], and South Africa [44], concluded that health care cost is significantly higher for physically inactive individuals than active individuals and specifically the cost on medication [40, 42].

Furthermore, significant effect from physical inactivity on productivity loss associated with health problems was revealed by another study conducted in Brazil. Physical inactivity less than 25thpercentile (≤25P) reported, ORadj 0.29(0.09-0.88) for productivity loss due to absenteeism [34].

Economic factors associated with physical (in) activity in LMIC

Income

For peer review only - http://bmjopen.bmj.com/site/about/guidelines.xhtml

Malaysian study revealed that every additional income of 1 RM reduces the time spent on vigorous physical activity by 0.01min and moderate physical activity by 0.01min [35]. It is further reported by a Malaysian study that 0.1% reduction in likelihood of participation in physical activity and 0.3% reduction in time spent on physical activity with income [39]. Brazilian study using data from Brazil`s National household sample survey 2008 analysed population by income quintiles and its association with active commuting. The study revealed that in urban area the quintile of population with the lowest income was found to have higher active transport (walking and cycling) level than the wealthiest portion (2-5 times higher) of the study population [46]($p<0.001$). Higher income levels were associated with lower physical activity (p for trend and higher sedentary time <0.001) among a sample of Chinese population[47].

Socioeconomic status

Cross sectional study carried out in China reported that people from upper socioeconomic (calculated based on education attainment, income, occupation) status (47.9%) participated in active transport compared to lower class (40.4%), lower middle class(27.7%) , upper middle class(36.2%) and p for trend was <0.0001[46]. Further, this study revealed that people in higher socioeconomic classes had less overall intensity of physical activity in METhours/week (114.4, 95%CI 106.6-122.5) compared to lower class (140.3, 95%CI 132.3-148.5), lower middle class (167.4, 95%CI 163.8-171), upper middle class (141.1. 95%CI 135.6-146.6) and p for trend <0.0001[46].

Employment

Cross sectional study conducted in a national representative sample in Malaysia revealed that employed individuals are 13.6% ($\beta=0.0136$, $p=0.036$) more likely to take participation decision

For peer review only - http://bmjopen.bmj.com/site/about/guidelines.xhtml

in physical activity [39] and amount of participation decision (β=0.167, p=0.032) than unemployed using a lognormal hurdle model [39]. The cross sectional study conducted in China further reported lower intensity of physical activity among unemployed individuals (-136) (95%CI -144.5 to -127.5) and non-manual workers (-25.1) (95%CI -30.6 to-19.6) compared to manual workers (0.0, reference) [48]. A global analysis of occupational structure and physical inactivity reported that individuals working in white-collar industry (84%) compared to agriculture were more likely to be physically inactive (OR=1.84, CI 1.73-1.95)[50].

Health Insurance

The study from Malaysia further reports that individuals who have insurance are 3.2% more likely to participate in physical activity but do not spend more time than their counterparts who are not covered by insurance [39].

Attractiveness/cost for leisure time physical activity

Attractiveness predicted student participation at leisure time physical activity (LTPA) participation of Teheran Universities (OR 1.073) while cost and time unable to predicted LTPA participation of them. The study concluded that constraining effect of cost and time could be adjusted by attractiveness of recreational activities [48]

Macroeconomic indicators

In Brazil, Gini index accounted for 12% variance in PA (adjusted R^2=0.12), if Gini index of the city increase by 1%, prevalence of sufficient physical activity would decrease by 41.5% for girls and 60.2% for boys. If HDI of city increase by 1% the prevalence of physical activity would

For peer review only - http://bmjopen.bmj.com/site/about/guidelines.xhtml

For peer review only

increase by 60.2 % [43]. Global analysis among 47 LMIC reported that HDI values associated with decreased levels of physical inactivity (OR=0.98, 95%CI 0.97-0.99)[50].

Research gaps

Majority of the studies have focused to identify socioeconomic factors associated with physical activity and identifying the economic burden of physical inactivity. There weren`t any study on economic evaluation on physical activity interventions in the evidence base on economics of physical activity in LMIC. Further, number of research is grossly inadequate an only a limited number of countries have studied any aspect of economics related to physical activity. Summary of identified gaps are given in box 1

BOX1

Gaps in evidence on economics of physical activity in LMIC

- Economic evaluations of interventions for physical activity promotion not available
- A standard method to quantify economic burden of physical inactivity at country level is needed
- Standard method of Operationalization of physical inactivity need to be used in economic research in LMIC for transferability of evidence
- Limited evidence available on economics of PA in LMIC compared to HIC
- Only few countries have studied any aspect of economics of PA in LMIC

Discussion

For peer review only - http://bmjopen.bmj.com/site/about/guidelines.xhtml

Economics of physical activity is a grossly neglected area in research agenda of LMIC. Only six LMIC have studied at least some aspect of economics of physical inactivity pioneered by Brazil. Other than few studies from Brazil scarcity of information on effectiveness or cost effectiveness of physical activity enhancement strategies in developing countries were noted in another global review on economic burden of physical activity related to cardiovascular diseases [52]. Though, high income countries bear the major share of economic burden of physical inactivity, LMIC bear the larger proportion of disease burden (75% DALYs attributable to physical inactivity) [36]. Thus, the economic burden of LMIC is predicted to be escalated in near future and considering LMIC separately even the current estimates of economic burden is huge in comparison to the income level of these countries. Though, cannot be directly compared due to methodological differences of these studies, estimates of economic burden for China and Brazil by global analysis [36] is in par with the two studies from China [41, 49] and Brazil [45]. It is of paramount importance that researchers should explore and identify cost effective interventions to improve physical activity within the context of LMIC. More research need to be conducted in local settings to make physical inactivity an "economic case" specially in LMIC to combat the huge disease burden in these countries with available limited resources.

As revealed by this review most of the studies were cross sectional studies to identify association of socioeconomic factors associated with (In) physical activity. None of the studies were focused on economic evaluations of physical activity interventions which is a huge vacuum for the evidence base on physical activity research in LMIC. Global attention should be drawn for the scarcity of research which could be possibly due to lack of capacity to conduct economic evaluations. Overview of economic evaluations in LMIC reports the

For peer review only

For peer review only - http://bmjopen.bmj.com/site/about/guidelines.xhtml

challenge of conducting economic evaluations in this context due to absence of routine cost accounting system and limited patient information systems [51]. Further, they reveal the limited capacity and funding to conduct health economic research in most LMIC [51]. Therefore, global attention needs to be drawn for more investment in training of researchers as on the job training or Master degree training [51] on economic studies with special reference to economic evaluations of new interventions in LMIC. An important suggestion that the findings of present review agree with the overview of economic evaluations in LMIC [51] to encourage economic evaluations in LMIC by advocating funders to request economic evaluations to be included for trails and other evaluations to ensure funding for those research. Further, separate funding streams need to be created specifically for economic evaluations [51] would contribute to fill the vacuum of research on economic evaluations of physical activity interventions in LMIC. In parallel to the increase in conduct and use of economic evaluations in priority settings in LMIC, this review also finds the increase in attention of researchers for research on economics of physical activity in LMIC only since 2006. However, economic evaluation studies on physical activity interventions are yet to be commenced in this context, which should be included to the priority research agenda in LMIC.

This review has shown that macroeconomic indicators of the country are significantly associated with physical activity of the population. Physical activity level increases with the increase of HDI [50] and decreases with Gini index[43], which is a measure of inequality of income and wealth distribution of populations. Though income level alone has shown a negative association with level of PA [35, 39, 46, 47] in LMIC, socioeconomic status of population subgroups as a whole have shown a positive association with the quality and quantity of physical activity in high income countries [53]. This would have contributed to the

For peer review only - http://bmjopen.bmj.com/site/about/guidelines.xhtml

positive association of level of physical activity and macroeconomic indicators of the countries [43, 50]. Employment status and the type of employment also have affected the level of PA [39, 48, 50]. Unemployed individuals have shown lower PA levels compared to employed individuals [39, 48]. Further studies need to be conducted to conclude on effect of health insurance on PA and effect of attractiveness of PA intervention compared to time and cost variables of PA interventions as there were limited evidence on this regard.

The policy makers and authorities need to be advocated on the consistent evidence from LMIC on increased health care cost and productivity loss due to physical inactivity [34, 37,38, 40, 42,44], possibility to escalating the economic burden related to physical inactivity in near future [36] and the need include physical activity promotion across all policies.

Limitations: Meta analysis of data was not possible in this review due to wide variation in focus and methods of the included studies for the review.

Conclusions: Economics of PA in LMIC should be a priority research area in LMIC. Especially the research on economic evaluations related to physical activity should be considered as a priority in research agenda in LMIC.

Contributors: SP and NKA developed the idea for the review with inputs from PDR. PDR and NKA conducted the review with the guidance of SP. PDR wrote the first draft. SP and NKA revised the manuscript. NKA will act as guarantor of the review.

Conflicts of interest: None

Funding: This research received no specific grant from any funding agency in the public, commercial or not-for-profit sectors

For peer review only - http://bmjopen.bmj.com/site/about/guidelines.xhtml

For peer review only

List of references

1. World Health Organization. Global Status Report on Non communicable Diseases 2014. Geneva: World Health Organization; 2014 (http://apps.who.int/iris/bitstream/10665/148114/1/9789241564854_eng.pdf?ua=1

2. Global health risks: mortality and burden of disease attributable to selected major risks. Geneva, World Health Organization, 2009.

3. Abegunde, D. O., Mathers, C. D., Adam, T., Ortegon, M., & Strong, K. (2007). The burden and costs of chronic diseases in low-income and middle-income countries. *The Lancet, 370*(9603), 1929-1938. doi:10.1016/S0140-6736(07)61696-1

4. World Health Organization.2017, *Detail-Non communicable diseases*, viewed on 23.05.2018, http://www.who.int/en/news-room/fact-sheets/detail/noncommunicable-diseases

5. Hallal, P. C., Andersen, L. B., Bull, F. C., Guthold, R., Haskell, W., & Ekelund, U. (2012). Global physical activity levels: surveillance progress, pitfalls, and prospects. *The Lancet, 380*(9838), 247-257. doi:10.1016/S0140-6736(12)60646-1

6. GBD 2015 Risk Factors Collaborators. Global, regional, and national comparative risk assessment of 79 behavioural, environmental and occupational, and metabolic risks or clusters of risks, 1990–2015: a systematic analysis for the Global Burden of Disease Study 2015. Lancet, 2016; 388(10053):1659-1724

For peer review only - http://bmjopen.bmj.com/site/about/guidelines.xhtml

For peer review only

7. World Health Organization.2018, *Detail-Physical activity*, viewed on 21.05.2018, http://www.who.int/en/news-room/fact-sheets/detail/physical-activity

8. WHO. 2015. Prevalence of insufficient physical activity among adults Data by World Bank income groups. Accessed at http://apps.who.int/gho/data/view.main.2487?lang=en

9. Bull, F. C., & Bauman, A. E. (2011). Physical Inactivity: The "Cinderella" Risk Factor for Noncommunicable Disease Prevention. *Journal of Health Communication, 16*(sup2), 13-26. doi:10.1080/10810730.2011.601226

10. National Coalition for Promoting Physical Activity. 2005. Economics of physical activity promotion United States. Accessed at http://www.ncppa.org/sites/default/files/Economics%20of%20Physical%20Activity%20Final.pdf

11. Ding, D., Kolbe-Alexander, T., Nguyen, B., Katzmarzyk, P. T., Pratt, M., & Lawson, K. D. (2017). The economic burden of physical inactivity: a systematic review and critical appraisal. *British Journal of Sports Medicine, 51*(19), 1392-1409. doi:10.1136/bjsports-2016-097385

12. Ranasinghe PD, Pokhrel S, Anokye NK.(2019) The economics of physical activity in low-income and middle-income countries: protocol for a systematic review. *BMJ Open*, 9:e022686. doi: 10.1136/bmjopen-2018-022686

13. Burnham, J. F. (2006). Scopus database: a review. *Biomed Digit Libr, 3*(1), 1742-5581.

For peer review only - http://bmjopen.bmj.com/site/about/guidelines.xhtml

14. Oldridge, N. B. (2008). Economic burden of physical inactivity: healthcare costs associated with cardiovascular disease. *European Journal of Cardiovascular Prevention & Rehabilitation, 15*(2), 130-139. doi: 10.1097/HJR.0b013e3282f19d42

15. Jeroen C. M. Barte & G. C. Wanda Wendel-Vos (2017) A Systematic Review of Financial Incentives for Physical Activity: The Effects on Physical Activity and Related Outcomes, Behavioral Medicine, 43:2, 79-90, DOI: 10.1080/08964289.2015.1074880

16. Abu-Omar, K., Rütten, A., Burlacu, I., Schätzlein, V., Messing, S., & Suhrcke, M. (2017). The cost-effectiveness of physical activity interventions: A systematic review of reviews. *Preventive Medicine Reports*, *8*, 72–78. http://doi.org/10.1016/j.pmedr.2017.08.006

17. Brown, V., Zapata Diomedi, B., Moodie, M., Veerman, J., & Carter, R. (2016). *A systematic review of economic analyses of active transport interventions that include physical activity benefits* (Vol. 45).

18. Pavey, T. G., Taylor, A. H., Fox, K. R., Hillsdon, M., Anokye, N., Campbell, J. L., Taylor, R. S. (2011). Effect of exercise referral schemes in primary care on physical activity and improving health outcomes: systematic review and meta-analysis. *BMJ, 343*. doi: 10.1136/bmj.d6462

19. Campbell, F., Holmes, M., Everson-Hock, E., Davis, S., Woods, H. B., Anokye, N., Kaltenthaler, E. (2015). A systematic review and economic evaluation of exercise referral schemes in primary care: a short report. *Health Technology Assessment, 19*(60), 1-+. doi: 10.3310/hta19600

For peer review only - http://bmjopen.bmj.com/site/about/guidelines.xhtml

20. Suhrcke, M., Boluarte, T. A., & Niessen, L. (2012). A systematic review of economic evaluations of interventions to tackle cardiovascular disease in low- and middle-income countries. [journal article]. *BMC Public Health, 12*(1), 2. doi: 10.1186/1471-2458-12-2

21. Milton, K., Macniven, R., & Bauman, A. (2014). Review of the epidemiological evidence for physical activity and health from low- and middle-income countries. [Review]. *Glob Public Health, 9*(4), 369-381.

22. Lachat, C., Otchere, S., Roberfroid, D., Abdulai, A., Seret, F. M., Milesevic, J., Kolsteren, P. (2013). Diet and physical activity for the prevention of noncommunicable diseases in low- and middle-income countries: a systematic policy review. [Meta-Analysis Review]. *PLoS Med, 10*(6), 11.

23. Hunter, R. F., Christian, H., Veitch, J., Astell-Burt, T., Hipp, J. A., & Schipperijn, J. (2015). The impact of interventions to promote physical activity in urban green space: a systematic review and recommendations for future research. [Research Support, Non-U S Gov't Review]. *Soc Sci Med, 124*, 246-256.

24. Sutton, L., Karan, A., & Mahal, A. (2014). Evidence for cost-effectiveness of lifestyle primary preventions for cardiovascular disease in the Asia-Pacific Region: a systematic review. *Globalization and Health*, *10*, 79. http://doi.org/10.1186/s12992-014-0079-3

25. Santatiwongchai, B., Chantarastapornchit, V., Wilkinson, T., Thiboonboon, K., Rattanavipapong, W., Walker, D. G., Teerawattananon, Y. (2015). Methodological Variation in Economic Evaluations Conducted in Low- and Middle-Income Countries: Information for Reference Case Development. *PLoS ONE, 10*(5), e0123853. doi: 10.1371/journal.pone.0123853

For peer review only - http://bmjopen.bmj.com/site/about/guidelines.xhtml

26. Nosratnejad, S., Rashidian, A., & Dror, D. M. (2016). Systematic Review of Willingness to Pay for Health Insurance in Low and Middle Income Countries. [Review]. *PLoS One, 11*(6).

27. World Bank Country and Lending Groups: Country classification (June 2017). https://datahelpdesk.worldbank.org/knowledgebase/articles/906519-world-bank-country-and-lending-groups>

28. Pokhrel, S., Quigley, M. A., Fox-Rushby, J., McCormick, F., Williams, A., Trueman, P., Renfrew, M. J. (2015). Potential economic impacts from improving breastfeeding rates in the UK. [Research Support, Non-U.S. Gov't]. *Arch Dis Child, 100*(4), 334-340. doi: 10.1136/archdischild-2014-306701

29. Murthy, S., John, D., Godinho, I. P., Godinho, M. A., Guddattu, V., & Nair, N. S. (2017). A protocol for a systematic review of economic evaluation studies conducted on neonatal systemic infections in South Asia. *Syst Rev, 6*(1), 252. doi: 10.1186/s13643-017-0648-7

30. Larg, A., & Moss, J. R. (2011). Cost-of-illness studies: a guide to critical evaluation. [Research Support, Non-U S Gov't]. *Pharmacoeconomics, 29*(8), 653-671.

31. National Institute for Health and Excellence (NICE), 2012. Methods for the development of NICE public health guidance (third edition), Appendix G Quality appraisal checklist-quantitative studies reporting correlations and associations.< https://www.nice.org.uk/process/pmg4/chapter/appendix-g-quality-appraisal-checklist-quantitative-studies-reporting-correlations-and >

For peer review only

For peer review only - http://bmjopen.bmj.com/site/about/guidelines.xhtml

32. Drummond, M. F., & Jefferson, T. O. (1996). Guidelines for authors and peer reviewers of economic submissions to the BMJ. *BMJ, 313*(7052), 275-283. doi: 10.1136/bmj.313.7052.275

33. Philips, Z., Ginnelly, L., Sculpher, M., Claxton, K., Golder, S., Riemsma, R., Glanville, J. (2004). Review of guidelines for good practice in decision-analytic modelling in health technology assessment. [Review]. *Health Technol Assess, 8*(36), 1-158.

34. Araujo, M. Y. C., et al. (2017). "Association between Costs Related to Productivity Loss and Modified Risk Factors among Users of the Brazilian National Health System." Journal of Occupational and Environmental Medicine **59**(3): 313-319.

35. Cheah, Y. K., et al. (2017). "The Income and Demographic Determinants of Physical Activity Intensity: Evidence from a Developing Country." Margin-Journal of Applied Economic Research **11**(3): 348-364.

36. Ding, D., et al. (2016). "The economic burden of physical inactivity: a global analysis of major non-communicable diseases." The Lancet **388**(10051): 1311-1324.

37. Min, J. Y. and K. B. Min (2016). "Excess Medical Care Costs Associated with Physical Inactivity among Korean Adults: Retrospective Cohort Study." International Journal of Environmental Research and Public Health **13**(1).

38. Turi, B. C., et al. (2015). "Walking and health care expenditures among adult users of the Brazilian public healthcare system: Retrospective cross-sectional study." Ciencia e Saude Coletiva **20**(11): 3561-3568.

For peer review only

For peer review only - http://bmjopen.bmj.com/site/about/guidelines.xhtml

39. Cheah, Y. K., et al. (2017). "Factors affecting participation decision and amount of physical activity among urban dwellers in Malaysia." Public Health **146**: 84-91.

40. Codogno, J. S., et al. (2015). "Physical inactivity of adults and 1-year health care expenditures in Brazil." International Journal of Public Health **60**(3): 309-316.

41. Zhang, J. and J. Chaaban (2013). "The economic cost of physical inactivity in China." Preventive Medicine **56**(1): 75-78.

42. Turi, B. C., et al. (2017). "Determinants of outpatient expenditure within primary care in the Brazilian national health system." Sao Paulo Medical Journal **135**(3): 205-212.

43. Silva, D. A. S. (2015). "Relationship between Brazilian adolescents' physical activity and social and economic indicators of the cities where they live." Perceptual and Motor Skills **120**(2): 355-366.

44. Patel, D., et al. (2011). "Participation in Fitness-Related Activities of an Incentive-Based Health Promotion Program and Hospital Costs: A Retrospective Longitudinal Study." American Journal of Health Promotion **25**(5): 341-348.

45. Bielemann, R. M., et al. (2015). "Burden of physical inactivity and hospitalization costs due to chronic diseases." Rev Saude Publica **49**(89102015000100307): 20.

46. Chen, M., et al. (2015). "Socioeconomic Status and Physical Activity in Chinese Adults: A Report from a Community-Based Survey in Jiaxing, China." PLOS ONE **10**(7): e0132918.

For peer review only - http://bmjopen.bmj.com/site/about/guidelines.xhtml

47. de Sá, T. H., et al. (2016). "Socioeconomic and regional differences in active transportation in Brazil." Revista de Saúde Pública **50**: 37.

48. Abdi, K. et al. (2014). Analysis Role of Cost, Time and Attractiveness at leisure time physical activities in students of Tehran universities, Iran. International Journal of Research in Management, (ISI),1(4): 16-29

49. Popkin, B. M., et al. (2006). "Measuring the full economic costs of diet, physical activity and obesity-related chronic diseases." Obes Rev **7**(3): 271-293.

50. Atkinson, K., Lowe, S., & Moore, S. (2016). Human development, occupational structure and physical inactivity among 47 low and middle income countries. *Preventive Medicine Reports, 3*, 40-45. doi:https://doi.org/10.1016/j.pmedr.2015.11.009

51. Pitt, C., Vassall, A., Teerawattananon, Y., Griffiths, U. K., Guinness, L., Walker, D., Hanson, K. (2016). Foreword: Health Economic Evaluations in Low- and Middle-income Countries: Methodological Issues and Challenges for Priority Setting. *Health economics*, 25 Suppl 1(Suppl Suppl 1), 1–5. doi:10.1002/hec.3319

52. Oldridge, N.B., (2008). Economic burden of physical inactivity: healthcare costs associated with cardiovascular disease. *Eur J Cardiovasc Prev Rehabil.* 15(2):130-9. doi: 10.1097/HJR.0b013e3282f19d42.

53. Earl S. Ford, Robert K. Merritt, Gregory W. Heath, Kenneth E. Powell, Richard A. Washburn, Andrea Kriska, Gwendolyn Haile, Physical Activity Behaviors in Lower and Higher Socioeconomic Status Populations, *American Journal of Epidemiology*, Volume 133, Issue 12, 15 June 1991, Pages 1246–1256, https://doi.org/10.1093/oxfordjournals.aje.a115836

For peer review only - http://bmjopen.bmj.com/site/about/guidelines.xhtml

For peer review only

For peer review only - http://bmjopen.bmj.com/site/about/guidelines.xhtml

Tables

Table 1: Summary of studies by focus and methods

	Number (n)	studies
**Focus**		
Economic burden of physical inactivity	4	Ding 2016[36] Zhang & Chaban 2012[41] Bielemann 2015[45] Popkin 2006[49]
Economic correlates of physical inactivity		
• Association of physical (in)activity and direct health cost	5	Min J.Y. & Min K.B. 2016[37] Turi 2015 [38] Codongo 2015[40] Turi 2017[42] Patel 2011[44]
• Association of physical (in)activity and productivity lost	1	Araujo 2017[34]
• Socioeconomic factors associated with physical activity participation and intensity	7	Cheah 2017[35] Cheah 2017[38] Silva A.S 2015[43], Chen 2015[46] Thiago 2016[47] Abdi 2014[48] Atkinson 2016[50]
Economic evaluations related to physical activity	0	
Study design		
Cross sectional	13	Araujo 2017[34] Cheah 2017[35] Ding 2016[36] Turi 2015[38] Cheah 2017[39] Codongo 2015[40] Zhang & Chaban 2012[41] Turi 2017[42] Bielemann 2015[45] Chen 2015[46] Thiago 2016[47] Popkin 2006[49] Atkinson 2016[50]
Ecological/Correlation study	2	Silva A.S 2015[43], Abdi 2014[48]
longitudinal	2	Min J.Y. & Min K.B. 2016[37] Patel 2011[44]
Interventions	0	
**Study population**		
General population	15	Araujo 2017[34] Cheah 2017[35] Ding 2016[36] Min J.Y. & Min K.B. 2016[37] Turi 2015[38] Cheah 2017[39] Codongo 2015[40] Zhang & Chaban 2012[41] Turi 2017[42] Patel 2011[44] Bielemann 2015[45] Thiago 2016[47] Chen 2015[46] Popkin 2006[49] Atkinson 2016[50]
Students	2	Silva A.S 2015[43], Abdi 2014[48]

For peer review only - http://bmjopen.bmj.com/site/about/guidelines.xhtml

Table 2: Summary of studies by PICOS

Author year	*Country/ Country category*	*Type of economic analysis*	*Population*	*Intervention*	*comparator*	*outcome*	*study design*
Araujo 2017[34]	Brazil- UMC	Economic correlates of PA*	≥50y adults	NA	NA	Strength of association between physical inactivity and productivity loss(OR,95%CI)	Cross sectional
Cheah 2017[35]	Malaysia- UMC	Economic correlates of PA	General population	NA	NA	Strength of association of income and physical activity (Coefficient of income)	Cross sectional
Ding 2016[36]	Global analysis-UMC/LMC/LIC	Economic burden	General population	NA	NA	Cost estimates, DALY	Cross sectional
Min J.Y. & Min K.B. 2016[37]	Korea- LIC	Economic correlates of PA	40-69 year adults	NA	NA	% increase (excess) of mean medical care expenditure in inactive adults compared to active and its significance	Retrospective cohort
Turi 2015[38]	Brazil-UMC	Economic correlates of PA	≥50y adults	NA	NA	Association of walking and health care expenditure(OR,95% CI)	Cross sectional
Cheah 2017[39]	Malaysia- UMC	Economic correlates of PA	Urban dwellers	NA	NA	Strength of association between participation decision, amount of physical activity with income, time, employment, insurance (coefficient)	Cross sectional
Codongo 2015[40]	Brazil - UMC	Economic correlates of PA	≥50y adults	NA	NA	Strength of association between physical inactivity and health care expenditure (OR, 95%CI)	Cross sectional

For peer review only - http://bmjopen.bmj.com/site/about/guidelines.xhtml

Zhang & Chaban 2012[41]	China - UMC	Economic burden	General population	NA	NA	Cost estimate of physical inactivity	Cross sectional
Turi 2017[42]	Brazil- UMC	Economic correlates of PA	≥50 years adults	NA	NA	Strength of association of physical activity and outpatient expenditure(OR, 95%CI)	Cross sectional
Silva A.S 2015[43]	Brazil- UMC	Economic correlates of PA	Year 9 students Public and private elementary school's	NA	NA	Strength of association of macroeconomic variables with physical activity(Pearson r,95% CI)	Correlation study
Patel 2011[44]	South Africa- UMC	Economic correlates of PA	Adults who are members of discovery health medical plan	Incentive based health promotion program	Not enrolled in the program	Changes in level of participation in fitness related activities, Subsequent probability of hospital admissions and cost	Retrospective observational study. Not a intervention study
Bielemann 2015[45]	Brazil- UMC	Economic burden	Above 40 year old adults Brazil	NA	NA	Inpatient cost estimates attributed to physical activity	Cross sectional
Thiago 2016[46]	Brazil- UMC	Economic correlates of PA	≥14 year general population Brazil	NA	NA	Strength of association of socioeconomic status and active transport	Cross sectional
Abdi 2014 [47]	Iran- UMC	Economic correlates of PA	University students Teheran Universities	NA	NA	Strength of association of time, cost and attractiveness with LTPA	Correlational survey

For peer review only - http://bmjopen.bmj.com/site/about/guidelines.xhtml

Chen 2015[48]	China - UMC	Economic correlates of PA	30-65 year permanent residents Jiaxing China	NA	NA	Strength of association of socioeconomic level and PA	Cross sectional
Popkin 2006[49]	China- UMC	Economic burden	General population	NA	NA	Cost estimate of physical inactivity	Cross sectional(case study developed from a review)
Atkinson 2016[50]	Global analysis-UMC/LMC/LIC	Economic correlates of PA	General population	NA	NA	Strength of association(OR) of physical activity and macroeconomic variables in 47 LMIC	Cross sectional

*PA- physical activity

For peer review only - http://bmjopen.bmj.com/site/about/guidelines.xhtml

Table 3: Methods of operationalization of physical activity and cost in economic studies of physical activity

	Number	studies
**Method of operationalization of physical activity**		
By WHO definition	1	Ding 2016[36]
Physical activity for >300 min during last 7 days	1	Silva A.S 2015[43]
Based on IPAQ	1	Atkinson 2016[50]
By Baecke questionnaire,	3	Araujo 2017[34],Turi 2015[38] Codongo,2015[40]Turi2017[42]
Self-reported vigorous or moderate physical activity during last 7 days	2	Cheah2017[35] , Cheah 2017[39]
Self-reported exercise 1-2 times or more per week	1	Min J.Y. & Min K.B. 2016[37]
Based on number of Gym visits per annum	1	Patel 2011[44]
Moderate or vigorous physical activity during leisure	1	Abdi 2014[45]
Performing any physical activity during leisure	1	Bielemann 2015[45]
Walking or cycling to work regardless of duration	1	Thiago 2016[47]
Not reported	1	Zhang & Chaban 2012[41]
**Operationalization of cost in economic studies of physical activity**		
**Method of operationalization of direct cost**		
cost of disease using population attributable fraction related to physical inactivity	3	Ding 2016[36] Zhang & Chaban 2012[41]
Inpatient cost	1	Bielemann 2015[45]
Total cost of Inpatient/outpatient/prescription cost	1	Min J.Y. & Min K.B. 2016[37]

For peer review only - http://bmjopen.bmj.com/site/about/guidelines.xhtml

Total cost of Medication dispensed/laboratory tests/ medical consultations	3	Turi 2015[38], Codongo 2015[40] Turi 2017[42]
**Method of operationalization of indirect cost**		
Loss of productivity due to premature mortality	1	Ding 2016[36]
Loss of productivity due to absenteeism and disability retirement	1	Araujo 2017[34]

Table 4: Quality appraisal of studies

		Number of studies	Studies
By NICE check list for quantitative studies on correlations/associations (n=13)			
Internal validity (section 2-4)	++*	11	Araujo 2017[34] Cheah2017[35] Turi 2015[38] Min J.Y. & Min K.B. 2016[37] Cheah 2017[39] Codongo,2015[40] Turi2017[42] Silva A.S 2015[43] Patel 2011[44] Chen 2015[46] Atkinson 2016[50]
	+**	2	Thiago 2016[47], Abdi 2014[48]
	-***	0	
External validity (section 1)	++	9	Turi 2015[38] Min J.Y. & Min K.B. 2016[37] Cheah 2017[39] Codongo,2015[40] Turi2017[42] Silva A.S 2015[43] Patel 2011[44] Chen 2015[46] Atkinson 2016[50]
	+	3	Araujo 2017[34] Cheah2017[35] Abdi 2014[48]
	-	0	
Check list by Larg & Moss for cost of illness studies (n=4****)			
Internal validity	++	1	Ding 2016[36]
	+	2	Zhang & Chaban 2012[41] Bielemann 2015[45]

For peer review only - http://bmjopen.bmj.com/site/about/guidelines.xhtml

	-	0	
External validity (Q2b(III))	++	0	
	+	1	Ding2016[36] Zhang & Chaban 2012[41] Bielemann 2015[45]
	-	0	

* (++) All or most check list criteria fulfilled (>75%) fulfilled

** (+) some checklist criteria (50%-75%) fulfilled

*** (-) None or few checklist criteria (<50%) fulfilled

**** [48] is a case study based on a review, thus information on methods is not adequate to report on quality

Table 5: Evidence on economics of physical activity in LMIC

Evidence	studies
**Economic burden**	
**Direct cost**	
UMC 8886(16.5%), LMC 1366(2.5%), LI 75(0.1%) (INT$ 100000, global %)	Ding 2016[36]
China 3.5 billion USD	Zhang & Chaban 2012[41]
Brazil hospital admission cost 0.73 billion USD	Bielemann 2015[45]
Inpatient and outpatient cost of physical inactivity in China 0.4 billion USD and 0.9 billion USD	Popkin 2006[49]
**Indirect cost** (INT $1000000, global %)	
UMC 3814(27.8%), LMC 1350(9.9%), 130(0.9%)	Ding 2016[36]
China 3.3 billion USD (2007)	Zhang & Chaban 2012[41]
**Economic correlates of physical inactivity**	

For peer review only - http://bmjopen.bmj.com/site/about/guidelines.xhtml

**Association of physical (in)activity and direct health cost**	
Health care cost significantly higher for physically inactive individuals than active individuals	Min J.Y. & Min K.B. 2016[37] Turi 2015[38] Codongo 2015[40] Turi 2017[42] Patel 2011[44]
**Association of physical (in)activity and productivity lost**	
Significant effect from Physical inactivity on productivity loss associated health problems	Araujo 2017[34]
**Socioeconomic factors associated with physical activity participation and intensity**	
negative association of income with PA	Cheah2017[35]Cheah 2017[39] Abdi 2014[47] Thiago 2016[46] Atkinson 2016[50]
Employment status is positively associated with PA	Cheah 2017[39] Chen 2015[48]
socioeconomic disparities associated with PA	Thiago 2016[46]
Association of PA and macroeconomic indicators<br>• PA positively associated with HDI<br>• PA negatively associated with Gini index	 Atkinson 2016[50]<br>Silva A.S 2015[43]
Attractiveness of LTPA* is the predictor of participation of university students in PA over the time and cost factor of PA	Abdi 2014[47]

*leisure time physical a

For peer review only - http://bmjopen.bmj.com/site/about/guidelines.xhtml

For peer review only

For peer review only - http://bmjopen.bmj.com/site/about/guidelines.xhtml

Table 1: Summary of studies by focus and methods

	Number (n)	studies
**Focus**		
Economic burden of physical inactivity	4	Ding 2016[36] Zhang & Chaban 2012[41] Bielemann 2015[45] Popkin 2006[49]
Economic correlates of physical inactivity		
• Association of physical (in)activity and direct health cost	5	Min J.Y. & Min K.B. 2016[37] Turi 2015 [38] Codongo 2015[40] Turi 2017[42] Patel 2011[44]
• Association of physical (in)activity and productivity lost	1	Araujo 2017[34]
• Socioeconomic factors associated with physical activity participation and intensity	7	Cheah 2017[35] Cheah 2017[38] Silva A.S 2015[43], Chen 2015[46] Thiago 2016[47] Abdi 2014[48] Atkinson 2016[50]
Economic evaluations related to physical activity	0	
Study design		
Cross sectional	13	Araujo 2017[34] Cheah 2017[35] Ding 2016[36] Turi 2015[38] Cheah 2017[39] Codongo 2015[40] Zhang & Chaban 2012[41] Turi 2017[42] Bielemann 2015[45] Chen 2015[46] Thiago 2016[47] Popkin 2006[49] Atkinson 2016[50]
Ecological/Correlation study	2	Silva A.S 2015[43], Abdi 2014[48]
longitudinal	2	Min J.Y. & Min K.B. 2016[37] Patel 2011[44]
Interventions	0	
**Study population**		
General population	15	Araujo 2017[34] Cheah 2017[35] Ding 2016[36] Min J.Y. & Min K.B. 2016[37] Turi 2015[38] Cheah 2017[39] Codongo 2015[40] Zhang & Chaban 2012[41] Turi 2017[42] Patel 2011[44] Bielemann 2015[45] Thiago 2016[47] Chen 2015[46] Popkin 2006[49] Atkinson 2016[50]
Students	2	Silva A.S 2015[43], Abdi 2014[48]

For peer review only

Table 2: Summary of studies by PICOS

Author year	*Country/ Country category*	*Type of economic analysis*	*Population*	*Intervention*	*comparator*	*outcome*	*study design*
Araujo 2017[34]	Brazil- UMC	Economic correlates of PA*	≥50y adults	NA	NA	Strength of association between physical inactivity and productivity loss(OR,95%CI)	Cross sectional
Cheah 2017[35]	Malaysia- UMC	Economic correlates of PA	General population	NA	NA	Strength of association of income and physical activity (Coefficient of income)	Cross sectional
Ding 2016[36]	Global analysis- UMC/LMC/LIC	Economic burden	General population	NA	NA	Cost estimates, DALY	Cross sectional
Min J.Y. & Min K.B. 2016[37]	Korea- LIC	Economic correlates of PA	40-69 year adults	NA	NA	% increase (excess) of mean medical care expenditure in inactive adults compared to active and its significance	Retrospective cohort
Turi 2015[38]	Brazil-UMC	Economic correlates of PA	≥50y adults	NA	NA	Association of walking and health care expenditure(OR,95% CI)	Cross sectional
Cheah 2017[39]	Malaysia- UMC	Economic correlates of PA	Urban dwellers	NA	NA	Strength of association between participation decision, amount of physical activity with income, time, employment, insurance (coefficient)	Cross sectional
Codongo 2015[40]	Brazil - UMC	Economic correlates of PA	≥50y adults	NA	NA	Strength of association between physical inactivity and health care expenditure (OR, 95%CI)	Cross sectional
Zhang & Chaban 2012[41]	China - UMC	Economic burden	General population	NA	NA	Cost estimate of physical inactivity	Cross sectional
Turi 2017[42]	Brazil- UMC	Economic correlates of PA	≥50 years adults	NA	NA	Strength of association of physical activity and outpatient expenditure(OR, 95%CI)	Cross sectional

For peer review only

Silva A.S 2015[43]	Brazil- UMC	Economic correlates of PA	Year 9 students Public and private elementary school's	NA	NA	Strength of association of macroeconomic variables with physical activity(Pearson r,95% CI)	Correlation study
Patel 2011[44]	South Africa- UMC	Economic correlates of PA	Adults who are members of discovery health medical plan	Incentive based health promotion program	Not enrolled in the program	Changes in level of participation in fitness related activities, Subsequent probability of hospital admissions and cost	Retrospective observational study. Not a intervention study
Bielemann 2015[45]	Brazil- UMC	Economic burden	Above 40 year old adults Brazil	NA	NA	Inpatient cost estimates attributed to physical activity	Cross sectional
Thiago 2016[46]	Brazil- UMC	Economic correlates of PA	≥14 year general population Brazil	NA	NA	Strength of association of socioeconomic status and active transport	Cross sectional
Abdi 2014 [47]	Iran- UMC	Economic correlates of PA	University students Teheran Universities	NA	NA	Strength of association of time, cost and attractiveness with LTPA	Correlational survey
Chen 2015[48]	China - UMC	Economic correlates of PA	30-65 year permanent residents Jiaxing China	NA	NA	Strength of association of socioeconomic level and PA	Cross sectional
Popkin 2006[49]	China- UMC	Economic burden	General population	NA	NA	Cost estimate of physical inactivity	Cross sectional(case study developed from a review)
Atkinson 2016[50]	Global analysis- UMC/LMC/LIC	Economic correlates of PA	General population	NA	NA	Strength of association(OR) of physical activity and macroeconomic variables in 47 LMIC	Cross sectional

*PA- physical activity

For peer review only

For peer review only

Table 3: Methods of operationalization of physical activity and cost in economic studies of physical activity

	Number	studies
**Method of operationalization of physical activity**		
By WHO definition	1	Ding 2016[36]
Physical activity for >300 min during last 7 days	1	Silva A.S 2015[43]
Based on IPAQ	1	Atkinson 2016[50]
By Baecke questionnaire,	3	Araujo 2017[34],Turi 2015[38] Codongo,2015[40]Turi2017[42]
Self-reported vigorous or moderate physical activity during last 7 days	2	Cheah2017[35] , Cheah 2017[39]
Self-reported exercise 1-2 times or more per week	1	Min J.Y. & Min K.B. 2016[37]
Based on number of Gym visits per annum	1	Patel 2011[44]
Moderate or vigorous physical activity during leisure	1	Abdi 2014[45]
Performing any physical activity during leisure	1	Bielemann 2015[45]
Walking or cycling to work regardless of duration	1	Thiago 2016[47]
Not reported	1	Zhang & Chaban 2012[41]
**Operationalization of cost in economic studies of physical activity**		
**Method of operationalization of direct cost**		
cost of disease using population attributable fraction related to physical inactivity	3	Ding 2016[36] Zhang & Chaban 2012[41]
Inpatient cost	1	Bielemann 2015[45]
Total cost of Inpatient/outpatient/prescription cost	1	Min J.Y. & Min K.B. 2016[37]
Total cost of Medication dispensed/laboratory tests/ medical consultations	3	Turi 2015[38], Codongo 2015[40] Turi 2017[42]
**Method of operationalization of indirect cost**		
Loss of productivity due to premature mortality	1	Ding 2016[36]
Loss of productivity due to absenteeism and disability retirement	1	Araujo 2017[34]

For peer review only - http://bmjopen.bmj.com/site/about/guidelines.xhtml

Table 4: Quality appraisal of studies

		Number of studies	Studies
By NICE check list for quantitative studies on correlations/associations (n=13)			
Internal validity (section 2-4)	++*	11	Araujo 2017[34] Cheah2017[35] Turi 2015[38] Min J.Y. & Min K.B. 2016[37] Cheah 2017[39] Codongo,2015[40] Turi2017[42] Silva A.S 2015[43] Patel 2011[44] Chen 2015[46] Atkinson 2016[50]
	+**	2	Thiago 2016[47], Abdi 2014[48]
	-***	0	
External validity (section 1)	++	9	Turi 2015[38] Min J.Y. & Min K.B. 2016[37] Cheah 2017[39] Codongo,2015[40] Turi2017[42] Silva A.S 2015[43] Patel 2011[44] Chen 2015[46] Atkinson 2016[50]
	+	3	Araujo 2017[34] Cheah2017[35] Abdi 2014[48]
	-	0	
Check list by Larg & Moss for cost of illness studies (n=4****)			
Internal validity	++	1	Ding 2016[36]
	+	2	Zhang & Chaban 2012[41] Bielemann 2015[45]
	-	0	
External validity (Q2b(III))	++	0	
	+	1	Ding2016[36] Zhang & Chaban 2012[41] Bielemann 2015[45]
	-	0	

* (++) All or most check list criteria fulfilled (>75%) fulfilled
** (+) some checklist criteria (50%-75%) fulfilled
*** (-) None or few checklist criteria (<50%) fulfilled
**** [48] is a case study based on a review, thus information on methods is not adequate to report on quality

For peer review only

Table 5: Evidence on economics of physical activity in LMIC

Evidence	studies
**Economic burden**	
**Direct cost**	
UMC 8886(16.5%), LMC 1366(2.5%), LI 75(0.1%) (INT$ 100000, global %)	Ding 2016[36]
China 3.5 billion USD	Zhang & Chaban 2012[41]
Brazil hospital admission cost 0.73 billion USD	Bielemann 2015[45]
Inpatient and outpatient cost of physical inactivity in China 0.4 billion USD and 0.9 billion USD	Popkin 2006[49]
**Indirect cost** (INT $1000000, global %)	
UMC 3814(27.8%), LMC 1350(9.9%), 130(0.9%)	Ding 2016[36]
China 3.3 billion USD (2007))	Zhang & Chaban 2012[41]
**Economic correlates of physical inactivity**	
**Association of physical (in)activity and direct health cost**	
Health care cost significantly higher for physically inactive individuals than active individuals	Min J.Y. & Min K.B. 2016[37] Turi 2015[38] Codongo 2015[40] Turi 2017[42] Patel 2011[44]
**Association of physical (in)activity and productivity lost**	
Significant effect from Physical inactivity on productivity loss associated health problem	Araujo 2017[34]
**Socioeconomic factors associated with physical activity participation and intensity**	
negative association of income with PA	Cheah2017[35]Cheah 2017[39] Abdi 2014[47] Thiago 2016[46] Atkinson 2016[50]
Employment status is positively associated with PA	Cheah 2017[39] Chen 2015[48]
socioeconomic disparities associated with PA	Thiago 2016[46]
Association of PA and macroeconomic indicators<br>• PA positively associated with HDI<br>• PA negatively associated with Gini index	 Atkinson 2016[50]<br>Silva A.S 2015[43]
Attractiveness of LTPA* is the predictor of participation of university students in PA over the time and cost factor of PA	Abdi 2014[47]

*leisure time physical activity

For peer review only

Figure 1

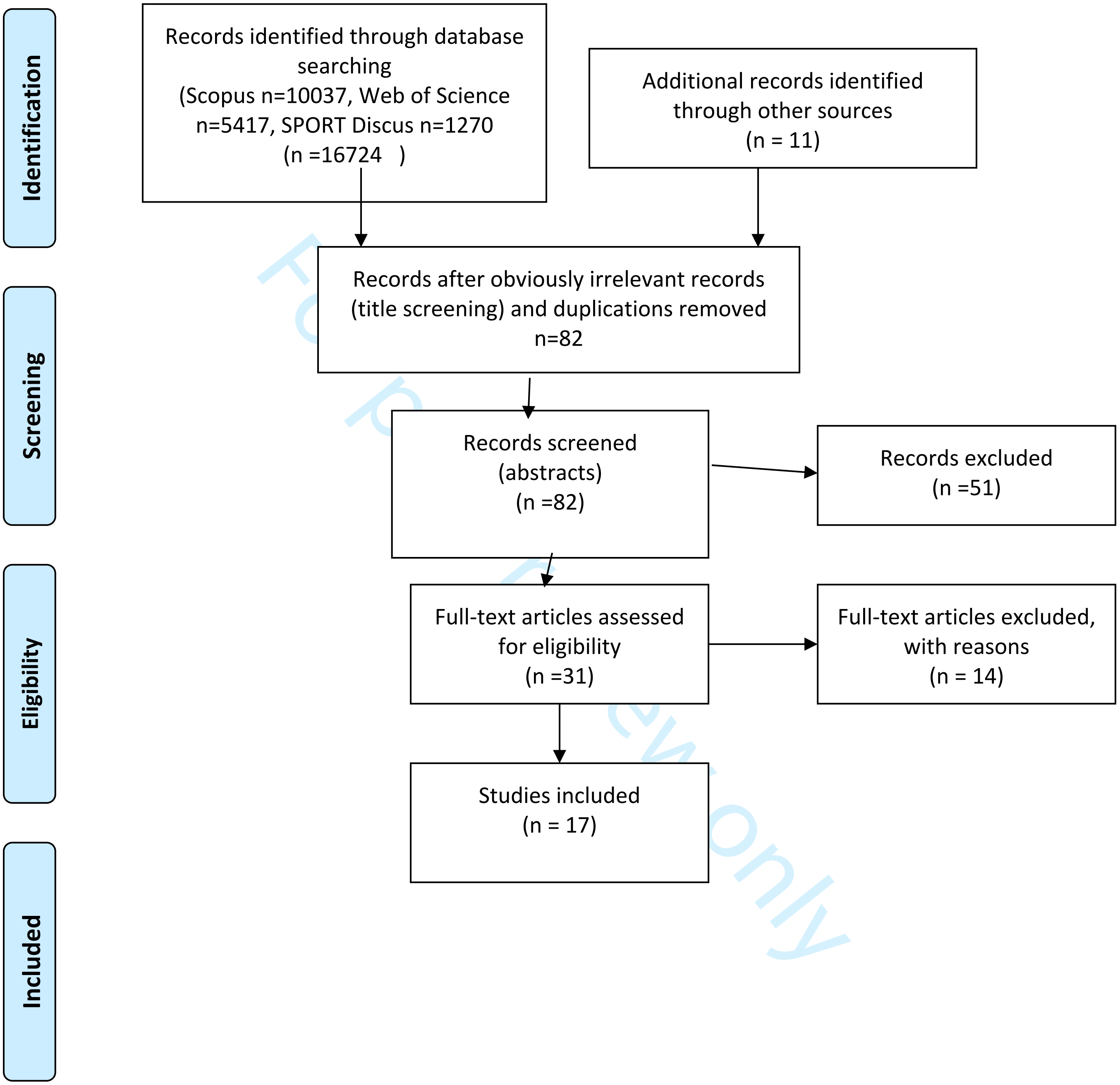

From: Moher D, Liberati A, Tetzlaff J, Altman DG, The PRISMA Group (2009). *P*referred *R*eporting *I*tems for *S*ystematic Reviews and *M*eta-*A*nalyses: The PRISMA Statement. PLoS Med 6(7): e1000097. doi:10.1371/journal.pmed1000097

For peer review only - http://bmjopen.bmj.com/site/about/guidelines.xhtml

Figure 2

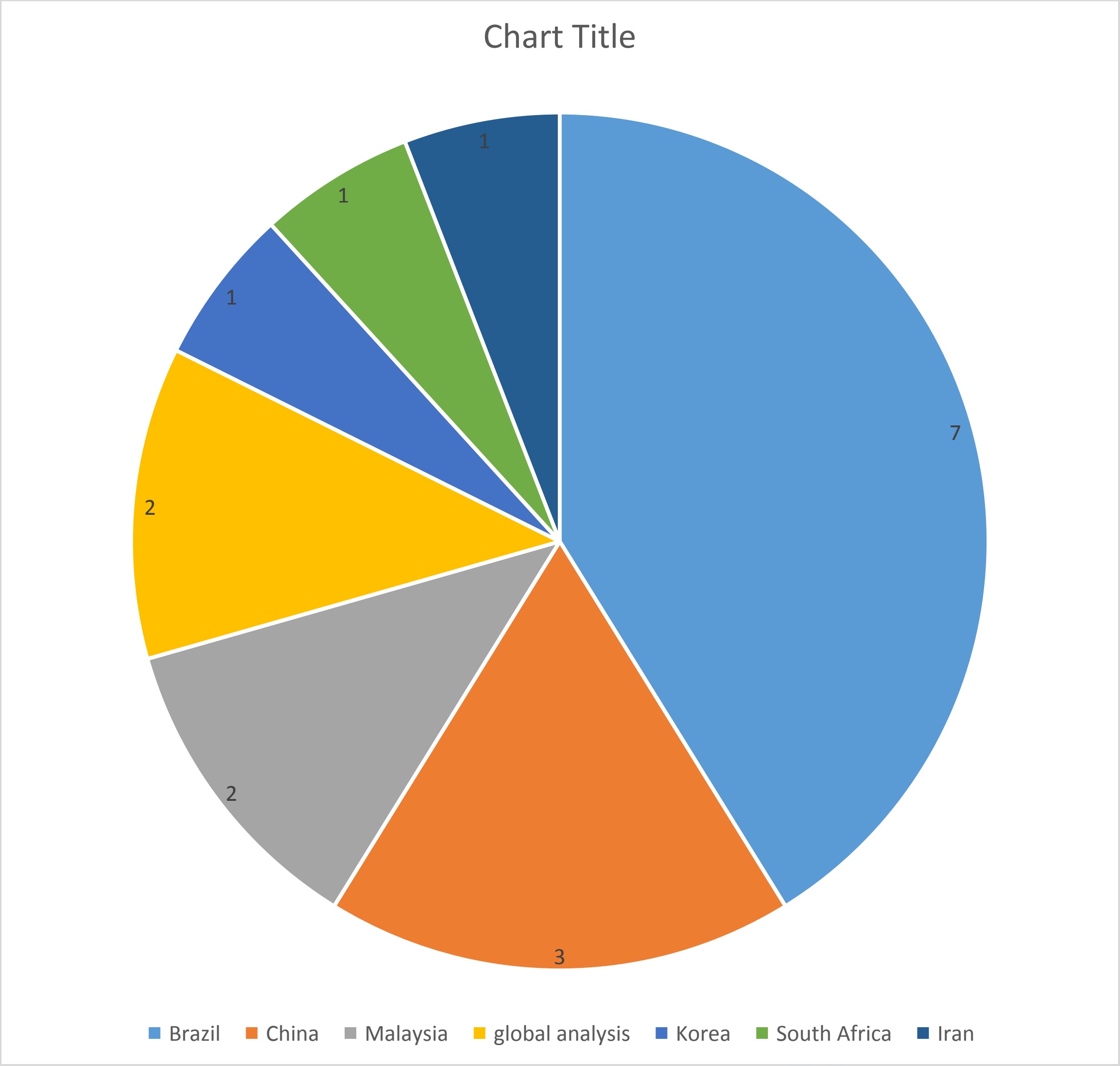
Chart Title
7
3
2
2
1
1
1
Brazil
China
Malaysia
global analysis
Korea
South Africa
Iran

Search Strategy

Scopus

((TITLE-ABKEY

((*cost* W/2 (*analyses* OR *effectiveness* OR *benefit* OR *minimization* OR *utility* OR *saving* OR *outcome* OR *consequence*))) AND DOCTYPE (*ar* OR *re*)) OR (TITLE-ABS-KEY ((*"demand"* OR *"supply"* OR *"market"* OR *"price"*)) AND DOCTYPE (*ar* OR *re*)) OR (TITLE-ABS-KEY ((*"incentives"* OR *"willingness to pay"* OR *"time use"*)) AND DOCTYPE (*ar* OR *re*)) OR (TITLE-ABS-KEY (*"economics"*) AND DOCTYPE (*ar* OR *re*)) OR (TITLE-ABS-KEY ((*"economic evaluation"* OR *"economic burden"* OR *"health care cost"*)) AND DOCTYPE (*ar* OR *re*))) AND ((TITLE-ABS-KEY (((((*active* W/2 (*lifestyle* OR *living* OR *leisure* OR *play* OR *recreation* OR *commuting*)))))) AND DOCTYPE (*ar* OR *re*)) OR (TITLE-ABS-KEY ((*"physical activity"* OR *"physical exercise"*)) AND DOCTYPE (*ar* OR *re*)) OR (TITLE-ABS-KEY ((*"physical inactivity"* OR *"physical fitness"*)) AND DOCTYPE (*ar* OR *re*)) OR (TITLE-ABS-KEY (*"Sedentary"*) AND DOCTYPE (*ar* OR *re*))) AND (LIMIT-TO (LANGUAGE , *"English"*))

SPORTDiscus

((TITLE-ABS-KEY ((*cost* n/2 (*analyses* OR *effectiveness* OR *benefit* OR *minimization* OR *utility* OR *saving* OR *outcome* OR *consequence*))) AND DOCTYPE (*ar* OR *re*)) OR (TITLE-ABS-KEY ((*"demand"* OR *"supply"* OR *"market"* OR *"price"*)) AND DOCTYPE (*ar* OR *re*)) OR (TITLE-ABS-KEY ((*"incentives"* OR *"willingness to pay"* OR *"time use"*)) AND DOCTYPE (*ar* OR *re*)) OR (TITLE-ABS-KEY (*"economics"*) AND DOCTYPE (*ar* OR *re*)) OR (TITLE-ABS-KEY ((*"economic evaluation"* OR *"economic burden"* OR *"health care cost"*)) AND DOCTYPE (*ar* OR *re*))) AND ((TITLE-ABS-KEY (((((*active* n/2 (*lifestyle* OR *living* OR *leisure* OR *play* OR *recreation* OR *commuting*)))))) AND DOCTYPE (*ar* OR *re*)) OR (TITLE-ABS-KEY ((*"physical activity"* OR *"physical exercise"*)) AND DOCTYPE (*ar* OR *re*)) OR (TITLE-ABS-KEY ((*"physical inactivity"* OR *"physical fitness"*)) AND DOCTYPE (*ar* OR *re*)) OR (TITLE-ABS-

For peer review only

KEY (*"Sedentary"*) AND DOCTYPE (*ar* OR *re*))) AND (LIMIT-TO (LANGUAGE , *"English"*))

Web of Science

((TITLE-ABS-KEY ((*cost* near/15 (*analyses* OR *effectiveness* OR *benefit* OR *minimization* OR *utility* OR *saving* OR *outcome* OR *consequence*))) AND DOCTYPE (*ar* OR *re*)) OR (TITLE-ABS-KEY ((*"demand"* OR *"supply"* OR *"market"* OR *"price"*)) AND DOCTYPE (*ar* OR *re*)) OR (TITLE-ABS-KEY ((*"incentives"* OR *"willingness to pay"* OR *"time use"*)) AND DOCTYPE (*ar* OR *re*)) OR (TITLE-ABS-KEY (*"economics"*) AND DOCTYPE (*ar* OR *re*)) OR (TITLE-ABS-KEY ((*"economic evaluation"* OR *"economic burden"* OR *"health care cost"*)) AND DOCTYPE (*ar* OR *re*))) AND ((TITLE-ABS-KEY (((((*active* near/15 (*lifestyle* OR *living* OR *leisure* OR *play* OR *recreation* OR *commuting*)))))) AND DOCTYPE (*ar* OR *re*)) OR (TITLE-ABS-KEY ((*"physical activity"* OR *"physical exercise"*)) AND DOCTYPE (*ar* OR *re*)) OR (TITLE-ABS-KEY ((*"physical inactivity"* OR *"physical fitness"*)) AND DOCTYPE (*ar* OR *re*)) OR (TITLE-ABS-KEY (*"Sedentary"*) AND DOCTYPE (*ar* OR *re*))) AND (LIMIT-TO (LANGUAGE , *"English"*))

For peer review only

Table 1: List of excluded studies following full text screening with reasons

1. Is Exercise a Good Investment for Adolescents? (1991). Journal of Sport & Exercise Psychology, 13(2), 208-208.	It is a brief one page document and not a research article. country studied also not mentioned
2. Codogno, J. S., Fernandes, R. A., & Monteiro, H. L. (2012). Physical activity and healthcare cost of type 2 diabetic patients seen at basic units of healthcare. [Article]. Arquivos Brasileiros de Endocrinologia e Metabologia, 56(1), 6-11	Article in Portuguese. (though English abstract available)
3. Ng, S. W., & Popkin, B. M. (2012). Time use and physical activity: A shift away from movement across the globe. [Article]. Obesity Reviews, 13(8), 659-680. doi: 10.1111/j.1467-789X.2011.00982.x	It only reports trends in energy expenditure including sedentary time and various physical activities for brazil, India and china and 2 other developed countries. It does not report how time affect the physical activity.
4. Oldridge, N. B. (2008). Economic burden of physical inactivity: healthcare costs associated with cardiovascular disease. European Journal of Cardiovascular Prevention & Rehabilitation, 15(2), 130-139. doi: 10.1097/HJR.0b013e3282f19d42	Though prevalence estimated for developing countries. cost is estimated only for developed countries
5. Kolbe-Alexander, T. L., Conradie, J., & Lambert, E. V. (2013). Clustering of risk factors for non-communicable disease and healthcare expenditure in employees with private health insurance presenting for health risk appraisal: a cross-sectional study. Bmc Public Health, 13. doi: 10.1186/1471-2458-13-1213	Studied clustering of other NCD risk factors with Physical inactivity, and association of clustered risk factors with health care expenditure. Data on expenditure due to physical inactivity not shown separately.
6. Huang, W. Y., & Wong, S. H. (2016). Time use clusters in children and their associations with sociodemographic factors. [Article]. Journal of Public Health (United Kingdom), 38(2), e106-e113. doi: 10.1093/pubmed/fdv088	Identified clusters according to time use for physical activity and sedentary behaviour. It does not assess time effect on physical activity participation.
7. Jim, C. Y., & Chen, W. Y. (2009). Leisure Participation Pattern of Residents in a New Chinese City. Annals of the Association of American Geographers, 99(4), 657-673. doi: 10.1080/00045600903066482	Assessed the association of leisure time and socioeconomic variables. Barriers to leisure participation identified by using a part of the survey questionnaire as a part of this study. HOWEVER, ASSESSED ONLY THE PERCIEVED BARRIERS.
8. Lopes, A. S., Silva, K. S., Filho, V. C. B., Bezerra, J., De Oliveira, E. S. A., & Nahas, M. V. (2014). Trends in screen time on week and weekend days in a representative sample of Southern Brazil	It basically report the trend of sedentary screen time.

For peer review only

students. [Article]. Journal of Public Health (United Kingdom), 36(4), 608-614. doi: 10.1093/pubmed/fdt133	
9. Matsudo, V. K. R., Matsudo, S. M., Araújo, T. L., Andrade, D. R., Oliveira, L. C., & Hallal, P. C. (2010). Time Trends in physical activity in the state of São Paulo, Brazil: 2002-2008. [Article]. Medicine and Science in Sports and Exercise, 42(12), 2231-2236. doi: 10.1249/MSS.0b013e3181e1fe8e	Reported only the trend in PA participation by socioeconomic status
10. Pratt, M., Norris, J., Lobelo, F., Roux, L., & Guijing, W. (2014). The cost of physical inactivity: moving into the 21st century. British Journal of Sports Medicine, 48(3), 171-173.	This is a review
11. Araujo, M. Y. C., et al. (2016). "Association between disability pension, nutritional condition and physical inactivity in adults from a middle-size Brazilian City." Journal of Physical Education (Maringa) 27(1).	Not directly relevant to economics of physical activity as it assess the association of disability pension and physical activity.
12. Sujatha, T., et al. (2003). "Timed activity studies for assessing the energy expenditure of women from an urban slum in south India." Food and Nutrition Bulletin 24(2): 193-199.	It does not assess the time effect on physical activity
13. Cecchini, M., et al. "Tackling of unhealthy diets, physical inactivity, and obesity: health effects and cost-effectiveness." The Lancet 376(9754): 1775-1784.	This is only a report, proposed a model and analysed cost for interventions for all healthy life styles by intervention type. Not assessed the cost for physical activity promotion by interventions separately or economic evaluation of such intervention
14. Meng, L., et al. (2013). "The Costs and Cost-Effectiveness of a School-Based Comprehensive Intervention Study on Childhood Obesity in China." PLOS ONE 8(10): e77971.	Cost effectiveness assessed only for combined intervention, not for physical activity alone

For peer review only

DATA EXTRACTION FORM FOR REVIEW ON ECONONOMICS AND PYSICAL ACTIVITY IN LOW AND MIDDLE INCOME COUNTRIES (ADAPTED FROM MURTHEY ET AL., 2017, POKHREL ET AL., 2014, PAVEY ET AL., 2011)

**1. GENERAL INFORMATION**		
1.1	Study ID	
1.2	Review author ID	
1.3	Date of review	
1.4	Title	
1.5	Author(s)	
1.6	Year of publication	
1.7	Country studied	
1.8	Income level of the country	
**2. CHRACTERISTIC OF THE STUDY**		
2.1	Aims and objectives/research question	
2.2	Eligibility decision Inclusion and exclusion criteria (?)	
2.3	Study design	
2.4	Study setting	
2.5	Total study duration	
2.6	Sample size	
2.7	Sample size calculation	
2.8	Recruitment	
2.9	Sample selection	
2.10	Method of allocation	
2.11	Perspective (i.e. Societal, health care system, service provider etc.)	
**3. CHARACTERISTICS OF POPULATION/CONDITION/INTERVENTION**		
3.1	**Population/participants**	
3.1.1	Type of population	

For peer review only

	(i.e. General population university students, group of employees)	
3.1.2	Age	
3.1.3	Gender	
3.1.4	Ethnicity	
3.2	**Condition**	
3.2.1	Definition of physical activity /physical inactivity/sedentary	
3.2.2	Definition of disease condition	
3.3	**Intervention**	
3.3.1	Type of physical activity/programme	
3.3.2	Comparator	
4.	**Data sources/Data analysis/results**	
4.1	**scope**	
4.1.1	Type of analysis (Ie.Costing/economic evaluation/association of economic factors with physical activity/other)	
4.1.2	Form of economic evaluation	
4.1.3	Time horizon	
4.1.4	Study assumptions	
4.2	**Cost**	
4.2.1	Reported costs/cost components	
4.2.2	Data sources for cost measures	
4.2.3	How cost was reported (i.e. marginal cost/ average cost/incremental cost/total cost/other)	
4.2.4	Discount rate	
4.2.5	Year of costing	
4.2.6	Currency unit	
4.2.7	Currency conversion rate	

For peer review only

4.2.8	Inflation adjustments	
4.3	**Outcomes**	
4.3.1	Reported outcomes (i.e. measures of effectiveness/efficacy/measures of effect)	
4.3.2	Data sources for the outcome measures	
4.3.4	Discount rate	
4.4	**Main Analysis**	
4.4.1	Statistical tests used	
4.4.2	Models used	
4.4.3	Economic evaluations	
4.5	**Sensitivity analysis**	
4.5.1	Performed/ not	
4.5.2	Type of analysis (i.e. deterministic/ stochastic)	
4.5.3	Variables used for sensitivity analysis	
4.5.4	Results of sensitivity analysis	
4.6	**Main results**	
4.6.1	Cost / cost effectiveness	
4.6.2	Other	
5.	**CONCLUSIONS AND WAY FORWARD**	
5.1	Key Conclusion(s)	
5.2	Suggestions by author for further research	
6.	**CHALLENGES**	
6.1	Author stated limitations	
6.2	Author stated strengths	
6.3	Miscellaneous comments from author	
6.4	Funding source	
6.5	Conflicts of interest	
**7.**	**QUALITY ASSESSMENT OF STUDIES**	

For peer review only

7.1	Is the study economic evaluation?	Yes/No, if *yes go to* Check list A
7.2	Is the economic evaluation model based?	Yes/No , if *yes* go to Check list B
7.3	Is it a cost of illness/cost of risk factor study?	Yes/No , if *yes* go to Check list C
7.4	Is it a quantitative study reporting correlation or association?	Yes/No , If *yes* go to Checklist D

For peer review only

**Method of rating the quality of studies from each check list (A, B, C, D) in accordance to the quality appraisal by NICE scale (Methods for the development of NICE public health guidance (third edition))**	
• Reviewer is requested to complete in detail the comment section of quality appraisal check lists so that the grade awarded for each study aspect is as transparent as possible<br>• At the end of completion of the check list each study is awarded an overall quality grading for internal validity (IV)and a separate one for external validity (EV)	
All or most of the checklist criteria have been fulfilled, where they have not been fulfilled the conclusions are very unlikely to alter	++
Some of the checklist criteria have been fulfilled, where they have not been fulfilled, or not adequately described, the conclusions are unlikely to alter	+
Few or no checklist criteria have been fulfilled and the conclusions are likely or very likely to alter.	-

CHECKLIST A

QUALITY ASSESSMENT FOR ECONOMIC EVALUATION(CHECKLIST FROM DRUMMOND AND JEFFERSON 1996)	Yes	No	Not clear	Not appropriate
**Study design**				
1) The research question is stated				
2) The economic importance of the research question is stated				
3) The viewpoint(s) of the analysis are clearly stated and justified				
4) The rationale for choosing the alternative programmes orb interventions compared is stated				
5) The alternatives being compared are clearly described				
6) The form of economic evaluation used is stated				
7) The choice of form of economic evaluation is justified in relation to the questions addressed				
**Data collection**				
8) The source(s) of effectiveness estimates used are stated				
9) Details of the design and results of effectiveness study are given (if based on a single study)				
10) Details of the method of synthesis or meta-analysis of estimates are given (if based on an overview of a number of effectiveness studies)				

For peer review only

QUALITY ASSESSMENT FOR ECONOMIC EVALUATION(CHECKLIST FROM DRUMMOND AND JEFFERSON 1996)	Yes	No	Not clear	Not appropriate
11) The primary outcome measure(s) for the economic evaluation are clearly stated				
12) Methods to value health states and other benefits are stated				
13) Details of the subjects from whom valuations were obtained are given				
14) Productivity changes (if included) are reported separately				
15) The relevance of productivity changes to the study question is discussed				
16) Quantities of resources are reported separately from their unit costs				
17) Methods for the estimation of quantities and unit costs are described				
18) Currency and price data are recorded				
19) Details of currency of price adjustments for inflation or currency conversion are given				
20) Details of any model used are given				
21) The choice of model used and the key parameters on which it is based are justified				
**Analysis and interpretation of results**				
22) Time horizon of costs and benefits is stated				
23) The discount rate(s) is stated				
24) The choice of rate(s) is justified				

For peer review only

QUALITY ASSESSMENT FOR ECONOMIC EVALUATION(CHECKLIST FROM DRUMMOND AND JEFFERSON 1996)	Yes	No	Not clear	Not appropriate
25) An explanation is given if costs or benefits are not discounted				
26) Details of statistical tests and confidence intervals are given for stochastic data				
27) The approach to sensitivity analysis is given				
28) The choice of variables for sensitivity analysis is justified				
29) The ranges over which the variables are varied are stated				
30) Relevant alternatives are compared				
31) Incremental analysis is reported				
32) Major outcomes are presented in a disaggregated as well as aggregated form				
33) The answer to the study question is given				
34) Conclusions follow from the data reported				
35) Conclusions are accompanied by the appropriate caveats				

Did the economic evaluation use a decision-analytic modelling framework?	Response: Yes(✓), No (×) , Not applicable (NA)<br><br>Instruction: If *Yes*, assess paper using the questions in Check list B, If *No/NA*, skip check list B

For peer review only

CHECKLIST B

QUALITY ASSESSMENT FOR DECISION-ANALYTIC MODELS(CHECKLIST FROM PHILIPS ET AL. 2004)			
**Quality criterion**	**Question(s)**	**Response (✓, ✗, NA)**	**Comments**
S1	Is there a clear statement of the decision problem?		
	Is the objective of the evaluation and model specified and consistent with the stated decision problem?		
	Is the primary decision maker specified?		
S2	Is the perspective of the model stated clearly?		
	Are the model inputs consistent with the stated perspective?		
	Has the scope of the model been stated and justified?		
	Are the outcomes of the model consistent with the perspective, scope and overall objective of the model?		
S3	Is the structure of the model consistent with a coherent theory of the health condition under evaluation?		
	Are the sources of data used to develop the structure of the model specified?		

For peer review only

QUALITY ASSESSMENT FOR DECISION-ANALYTIC MODELS(CHECKLIST FROM PHILIPS ET AL. 2004)			
**Quality criterion**	**Question(s)**	**Response (✓, ✗, NA)**	**Comments**
	Are the causal relationships described by the model structure justified appropriately?		
S4	Are the structural assumptions transparent and justified?		
	Are the structural assumptions reasonable given the overall objective, perspective and scope of the model?		
S5	Is there a clear definition of the options under evaluation?		
	Have all feasible and practical options been evaluated?		
	Is there justification for the exclusion for the exclusion of feasible options?		
S6	Is the chosen model type appropriate given the decision problem and specified causal relationships within the model?		
S7	Is the time horizon of the model sufficient to reflect all important differences between options?		
	Are the time horizon of the model, the duration of treatment and the duration of treatment effect described and justified?		

For peer review only

QUALITY ASSESSMENT FOR DECISION-ANALYTIC MODELS(CHECKLIST FROM PHILIPS ET AL. 2004)			
**Quality criterion**	**Question(s)**	**Response (✓, ✗, NA)**	**Comments**
S8	Do the disease states (state transition model) or the pathways (decision tree model) reflect the underlying biological process of the disease in question and the impact of interventions?		
S9	Is the cycle length defined and justified in terms of natural history of disease?		
D1	Are the data identification methods transparent and appropriate given the objectives of the model?		
	Where choices have been made between data sources, are these justified appropriately?		
	Has particular attention been paid to identifying data for the important parameters in the model?		
	Has the quality of the data been assessed appropriately?		
	Where expert opinion has been used, are the methods described and justified?		
D2	Is the data modelling methodology based on justifiable statistical and epidemiological techniques?		
D2a	Is the choice of baseline data described and justified?		

For peer review only

QUALITY ASSESSMENT FOR DECISION-ANALYTIC MODELS(CHECKLIST FROM PHILIPS ET AL. 2004)			
**Quality criterion**	**Question(s)**	**Response (✓, ✗, NA)**	**Comments**
	Are transition probabilities calculated appropriately?		
	Has a half-cycle correction been applied to both cost and outcome?		
	If not, has this omission been justified?		
D2b	If relative treatment effects have been derived from trail data, have they been synthesised using appropriate techniques?		
	Have the methods and assumptions used to extrapolate short-term results to final outcomes been documented and justified?		
	Have alternative extrapolation assumptions been explored through sensitivity analysis?		
	Have assumptions regarding the continuing effect of treatment once treatment is complete been documented and justified?		
	Have alternative assumptions regarding the continuing effect of treatment been explored through sensitivity analysis?		
D2c	Are the costs incorporated into the model justified?		
	Has the source for all costs been described?		

For peer review only

QUALITY ASSESSMENT FOR DECISION-ANALYTIC MODELS(CHECKLIST FROM PHILIPS ET AL. 2004)			
**Quality criterion**	**Question(s)**	**Response (✓, ✗, NA)**	**Comments**
	Have discount rates been described and justified given the target decision-maker?		
D2d	Are the utilities incorporated into the model appropriate?		
	Is the source for the utility weights referenced?		
	Are the methods of derivation for the utility weights justified?		
D3	Have all data incorporated into the model been described and referenced in sufficient detail?		
	Has the use of mutually inconsistent data been justified (i.e. are assumptions and choices appropriate)?		
	Is the process of data incorporation transparent?		
	If data have been incorporated as distributions, has the choice of distribution for each parameter been described and justified?		
	If data have been incorporated as distributions, is it clear that second order uncertainty is reflected?		
D4	Have the four principal types of uncertainty been addressed?		

For peer review only

QUALITY ASSESSMENT FOR DECISION-ANALYTIC MODELS(CHECKLIST FROM PHILIPS ET AL. 2004)			
**Quality criterion**	**Question(s)**	**Response (✓, ✗, NA)**	**Comments**
	If not, has the omission of particular forms of uncertainty been justified?		
D4a	Have methodological uncertainties been addressed by running alternative versions of the model with different methodological assumptions?		
D4b	Is there evidence that structural uncertainties have been addressed via sensitivity analysis?		
D4c	Has heterogeneity been dealt with by running the model separately for different subgroups?		
D4d	Are the methods of assessment of parameter uncertainty appropriate?		
	If data are incorporated at point estimates, are the ranges used for sensitivity analysis stated clearly and justified?		
C1	Is there evidence that the mathematical logic of the model has been tested thoroughly before use?		
C2	Are any counterintuitive results from the model explained and justified?		

For peer review only

QUALITY ASSESSMENT FOR DECISION-ANALYTIC MODELS(CHECKLIST FROM PHILIPS ET AL. 2004)			
**Quality criterion**	**Question(s)**	**Response (✓, ✗, NA)**	**Comments**
	If the model has been calibrated against independent data, has any differences been explained and justified?		
	Have the results of the model been compared with those of previous models and any differences in results explained?		

For peer review only

CHECKLIST C

QUALITY ASSESSMNET FOR COST OF ILLNESS STUDIES (ADAPTED FROM LARG AND MOSS, 2011)			
Responses (marked Similar to NICE check list, write the answer where relevant under the comment section)<br><br>++ Indicates that for that particular aspect of study design, the study has been designed or conducted in such a way as to minimise the risk of bias.<br>+ Indicates that either the answer to the checklist question is not clear from the way the study is reported, or that the study may not have addressed all potential sources of bias for that particular aspect of study design.<br>- Should be reserved for those aspects of the study design in which significant sources of bias may persist.<br>NR (Not Reported) - Should be reserved for those aspects in which the study under review fails to report how they have (or might have) been considered.<br>NA (Not Applicable) Should be reserved for those study design aspects that are not applicable given the study design under review (for example, allocation concealment would not be applicable for case–control studies).			
	Question	Response *++/+/-NR/NA*	Comment
1.	Analytical framework: what costs should have been measured?		

	(a) What was the motivation and perspective of the study?<br><br>(b) Was the appropriate epidemiologic approach taken?<br><br>(c) Was the study question well specified?<br>(i) Were all relevant, non-trivial cost components and their stakeholders identified?<br>(ii) Were necessary timeframes specified?<br>(iii) Was a case of disease or risk factor adequately and appropriately defined?<br>(iv) Was the counterfactual population occurrence plausible and meaningful?		
2.	(2) Methodology and data: how well were resource use and productivity losses measured?<br><br>(a) Was an appropriate method(s) of quantification used, such that;<br>(i) Additional, or excess, costs were measured?<br>(ii) only costs specific to (caused by) the health problem were included (confounders controlled)?<br>(iii) All important effects were captured?<br>(iv) Important differences across subpopulations were accounted for?<br>(v) The required level of detail could be provided?<br><br>(b) Was the resource quantification method(s) well executed?<br>(i) For population-based studies, were cost allocation methods, data and assumptions valid?<br>(ii) For person-based studies, were appropriate statistical tests performed and reported?<br>(iii) Were data representative of the study population?<br>(iv) Were there any other relevant resource quantification issues?		

For peer review only

	(c) Were healthcare resources valued appropriately?<br><br>(d) Was the approach for valuing production losses justified, and assumptions valid?<br><br>(e) Was the inclusion of intangible costs appropriate:<br>(i) Was double counting of mortality-related production losses avoided?<br>(ii) Were losses valued appropriately, given the study's perspective?		
3	Analysis and reporting<br>(a) Did the analysis address the study question?<br><br>(b) Was a range of estimates presented?<br><br>(c) Were the main uncertainties identified?<br><br>(d) Was a sensitivity analysis performed on:<br><br>(i) important (uncertain) parameter estimates?<br>(ii) key assumptions? (including the counterfactual)<br>(iii) point estimates? (based on confidence or credible intervals)<br><br>(e) Was adequate documentation and justification given for cost components, data and sources, assumptions and methods?<br><br>(f) Was uncertainty around the estimates and its implications adequately discussed?<br><br>(g) Were important limitations discussed regarding the cost components, data, assumptions and methods?		

For peer review only

	(h) Were the results presented at the appropriate level of detail to answer the study question (cost components; disease subtypes, severity, stage; subpopulation groups, cost bearers)?		

For peer review only

CHECKLIST D

QUALITY ASSESSMENT FOR QUANTITATIVE STUDIES REPORTING CORRELATIONS AND ASSOCIATIONS(NICE,2012 BASED ON 'GRAPHICAL APPRAISAL TOOL FOR EPIDEMIOLOGICAL STUDIES(GATE)` BY JACKSON ET AL. (2006)
Responses:<br><br>++ Indicates that for that particular aspect of study design, the study has been designed or conducted in such a way as to minimise the risk of bias.<br><br>+ Indicates that either the answer to the checklist question is not clear from the way the study is reported, or that the study may not have addressed all potential sources of bias for that particular aspect of study design.<br><br>- Should be reserved for those aspects of the study design in which significant sources of bias may persist.

NR (Not Reported) - Should be reserved for those aspects in which the study under review fails to report how they have (or might have) been considered.<br><br>NA (Not Applicable) Should be reserved for those study design aspects that are not applicable given the study design under review (for example, allocation concealment would not be applicable for case–control studies).			
**Section 1: Population**			
**Number**	**Question**	**Response**<br><br>**++/+/-/ NR/NA**	**Comments**
1.1	Is the source population or source area well described?<br><br>Was the country (e.g. developed or non-developed, type of health care system), setting (primary schools, community centres etc), location (urban, rural), population demographics etc adequately described?		
1.2	Is the eligible population or area representative of the source population or area?<br><br>Was the recruitment of individuals, clusters or areas well defined (e.g. advertisement, birth register)?<br><br>Was the eligible population representative of the source? Were important groups underrepresented?		
1.3	Do the selected participants or areas represent the eligible population or area?<br><br>Was the method of selection of participants from the eligible population well described?<br><br>What % of selected individuals or clusters agreed to participate? Were there any sources of bias?		

For peer review only

	Were the inclusion or exclusion criteria explicit and appropriate?		
**Section 2: Method of selection of exposure (or comparison) group**			
2.1	Selection of exposure (and comparison) group. How was selection bias minimised?<br><br>How was selection bias minimised?		
2.2	Was the selection of explanatory variables based on a sound theoretical basis?<br><br>How sound was the theoretical basis for selecting the explanatory variables?		
2.3	Was the contamination acceptably low?<br><br>Did any in the comparison group receive the exposure?<br><br>If so, was it sufficient to cause important bias?		
2.4	How well were likely confounding factors identified and controlled?<br><br>Were there likely to be other confounding factors not considered or appropriately adjusted for?<br><br>Was this sufficient to cause important bias?		
**Section 3: Outcomes**			
3.1	Were the outcome measures and procedures reliable?<br><br>Were outcome measures subjective or objective (e.g. biochemically validated nicotine levels ++ vs self-reported smoking −)?		

For peer review only

	How reliable were outcome measures (e.g. inter- or intra-rater reliability scores)?<br><br>Was there any indication that measures had been validated (e.g. validated against a gold standard measure or assessed for content validity)?		
3.2	Were the outcome measurements complete?<br><br>Were all or most of the study participants who met the defined study outcome definitions likely to have been identified?		
3.3	Were all important outcomes assessed?<br><br>Were all benefits and harms assessed?<br><br>Was it possible to determine the overall balance of benefits and harms of intervention versus comparisons?		
3.4	Was there a similar follow-up time in exposure and comparison groups?<br><br>If groups are followed for different lengths of time, then more events are likely to occur in the group followed-up for longer distorting the comparison.<br><br>Analyses can be adjusted to allow for differences in length of follow-up (e.g. using person-years).		
3.5	Was follow-up time meaningful?<br><br>Was follow-up long enough to assess long-term benefits and harms?		

For peer review only

	Was it too long, e.g. participants lost to follow-up?		
**Section 4: Analyses**			
4.1	Was the study sufficiently powered to detect an intervention effect (if one exists)?<br><br>A power of 0.8 (i.e. it is likely to see an effect of a given size if one exists, 80% of the time) is the conventionally accepted standard.<br><br>Is a power calculation presented? If not, what is the expected effect size? Is the sample size adequate?		
4.2	Were multiple explanatory variables considered in the analyses?<br><br>Were there sufficient explanatory variables considered in the analysis?		
4.3	Were the analytical methods appropriate?<br><br>Were important differences in follow-up time and likely confounders adjusted for?		
4.4	Was the precision of association given or calculable? Is association meaningful?<br><br>Were confidence intervals or p values for effect estimates given or possible to calculate?<br><br>Were CIs wide or were they sufficiently precise to aid decision-making? If precision is lacking, is this because the study is under-powered?		
**Section 5: Summary**			

For peer review only

5.1	Are the study results internally valid (i.e. unbiased)?<br><br>How well did the study minimise sources of bias (i.e. adjusting for potential confounders)?<br><br>Were there significant flaws in the study design?		
5.2	Are the findings generalizable to the source population (i.e. externally valid)?<br><br>Are there sufficient details given about the study to determine if the findings are generalizable to the source population?<br><br>Consider: participants, interventions and comparisons, outcomes, resource and policy implications		

For peer review only

CHECKLIST D

**QUALITY ASSESSMENT FOR QUANTITATIVE STUDIES REPORTING CORRELATIONS AND ASSOCIATIONS(NICE,2012 BASED ON 'GRAPHICAL APPRAISAL TOOL FOR EPIDEMIOLOGICAL STUDIES(GATE)` BY JACKSON ET AL. (2006)**			
Responses:<br><br>++ Indicates that for that particular aspect of study design, the study has been designed or conducted in such a way as to minimise the risk of bias.<br><br>+ Indicates that either the answer to the checklist question is not clear from the way the study is reported, or that the study may not have addressed all potential sources of bias for that particular aspect of study design.<br><br>- Should be reserved for those aspects of the study design in which significant sources of bias may persist.<br><br>NR (Not Reported) - Should be reserved for those aspects in which the study under review fails to report how they have (or might have) been considered.<br><br>NA (Not Applicable) Should be reserved for those study design aspects that are not applicable given the study design under review (for example, allocation concealment would not be applicable for case–control studies).			
**Section 1: Population**			
**Number**	**Question**	**Response**<br><br>**++/+/-/ NR/NA**	**Comments**
1.1	Is the source population or source area well described?		

	Was the country (e.g. developed or non-developed, type of health care system), setting (primary schools, community centres etc), location (urban, rural), population demographics etc adequately described?		
1.2	Is the eligible population or area representative of the source population or area?<br><br>Was the recruitment of individuals, clusters or areas well defined (e.g. advertisement, birth register)?<br><br>Was the eligible population representative of the source? Were important groups underrepresented?		
1.3	Do the selected participants or areas represent the eligible population or area?<br><br>Was the method of selection of participants from the eligible population well described?<br><br>What % of selected individuals or clusters agreed to participate? Were there any sources of bias?<br><br>Were the inclusion or exclusion criteria explicit and appropriate?		
**Section 2: Method of selection of exposure (or comparison) group**			
2.1	Selection of exposure (and comparison) group. How was selection bias minimised?<br><br>How was selection bias minimised?		

For peer review only

2.2	Was the selection of explanatory variables based on a sound theoretical basis?<br><br>How sound was the theoretical basis for selecting the explanatory variables?		
2.3	Was the contamination acceptably low?<br><br>Did any in the comparison group receive the exposure?<br><br>If so, was it sufficient to cause important bias?		
2.4	How well were likely confounding factors identified and controlled?<br><br>Were there likely to be other confounding factors not considered or appropriately adjusted for?<br><br>Was this sufficient to cause important bias?		
**Section 3: Outcomes**			
3.1	Were the outcome measures and procedures reliable?<br><br>Were outcome measures subjective or objective (e.g. biochemically validated nicotine levels ++ vs self-reported smoking −)?<br><br>How reliable were outcome measures (e.g. inter- or intra-rater reliability scores)?		

For peer review only

	Was there any indication that measures had been validated (e.g. validated against a gold standard measure or assessed for content validity)?		
3.2	Were the outcome measurements complete?<br><br>Were all or most of the study participants who met the defined study outcome definitions likely to have been identified?		
3.3	Were the outcome measurements complete?<br><br>Were all or most of the study participants who met the defined study outcome definitions likely to have been identified?		
3.4	Was there a similar follow-up time in exposure and comparison groups?<br><br>If groups are followed for different lengths of time, then more events are likely to occur in the group followed-up for longer distorting the comparison.<br><br>Analyses can be adjusted to allow for differences in length of follow-up (e.g. using person-years).		
3.5	Was follow-up time meaningful?<br><br>Was follow-up long enough to assess long-term benefits and harms?<br><br>Was it too long, e.g. participants lost to follow-up?		
**Section 4: Analyses**			
4.1	Was the study sufficiently powered to detect an intervention effect (if one exists)?		

For peer review only

	A power of 0.8 (i.e. it is likely to see an effect of a given size if one exists, 80% of the time) is the conventionally accepted standard.<br>Is a power calculation presented? If not, what is the expected effect size? Is the sample size adequate?		
4.2	Were multiple explanatory variables considered in the analyses?<br>Were there sufficient explanatory variables considered in the analysis?		
4.3	Were the analytical methods appropriate?<br>Were important differences in follow-up time and likely confounders adjusted for?		
4.4	Was the precision of association given or calculable? Is association meaningful?<br>Were confidence intervals or p values for effect estimates given or possible to calculate?<br>Were CIs wide or were they sufficiently precise to aid decision-making? If precision is lacking, is this because the study is under-powered?		
**Section 5: Summary**			
5.1	Are the study results internally valid (i.e. unbiased)?<br>How well did the study minimise sources of bias (i.e. adjusting for potential confounders)?<br>Were there significant flaws in the study design?		

For peer review only

5.2	Are the findings generalizable to the source population (i.e. externally valid)?<br><br>Are there sufficient details given about the study to determine if the findings are generalizable to the source population?<br><br>Consider: participants, interventions and comparisons, outcomes, resource and policy implications		

For peer review only

Summary of studies by country, country category, year of publication and focus

	Study	Country*	Category**	Year of publication	Focus of the study
1	Araujo et al[34]	Brazil	UMC	2017	Association between costs related to productivity loss and modified risk factors among uses of Brazilian National Health System
2	Cheah et al [35]	Malysia	UMC	2017a	Test the hypothesis "income is positively associated with the intensity of physical activity" by using data from a developing country(Malaysia)
3	Ding et al [36]	LMIC	LMIC	2016	Estimate direct health care cost, productivity losses and disability adjusted life years attributable to physical inactivity with standardized methods for 142 countries
4	Min JY & Min KB[37]	Korea	LI	2016	Estimate direct medical costs attributable to physical inactivity using 2002-2010
5	Turi et al [38]	Brazil	UMC	2015	Walking and health care expenditure among adults users of Brazilian health care system
6	Cheah et al [39]	Malaysia	UMC	2017b	Factors associated with participation decision and amount of participation among urban dwellers in Malaysia
7	Codongo et al[40]	Brazil	UMC	2015	Association between physical inactivity in different domains and direct public health care expenditure in adults
8	Zhang & Chaban [41]	China	UMC	2012	Total economic burden of physical inactivity in China
9	Turi et al [42]	Brazil	UMC	2017	Identify demographic, socioeconomic , life style and clinical factors associated with direct health care expenditure in primary care among adults through Brazilian health care system

For peer review only

10	Silva A.S [43]	Brazil	UMC	2015	Relationship between sufficient amount of physical activity among Brazilian adolescents and economic and social indicators of cities where they live
11	Patel et al [44]	South Africa	UMC	2011	Participation in fitness related activities of an incentive based health promotion programme and hospital costs
12	Bielmen et al[45]	Brazil	UMC	2014	Physical inactivity related inpatient costs of chronic non communicable diseases
13	Thiago et al[46]	Brazil	UMC	2015	Socioeconomic and regional difference in active transport in Brazil
14	Abdi et al [47]	Iran	UMC	2013	Compare the role of cost, time and attractiveness in predicting the participation level of student of Teheran Universities during their leisure time physical activities
15	Chen et al[48]	China	UMC	2015	Association of socioeconomic status with intensity of different types of physical activity in Chineese adults aiming to outline and projecting socioeconomic disparities among population undergoing rapid nutrient transition
16	Popkin et al[49]	China	UMC	2006	Case study from review of economic costs of diet, physical inactivity and obesity related chronic diseases to provide guidance to utilize the review for economic analysis of obesity
17	Atkinson et al[50]	LMIC	LMIC	2015	Association of occupational category and their physical inactivity and the association of human development, economic development, urbanization and physical inactivity

For peer review only

Quality appraisal of included studies based on standard check lists

1. Summary of quality appraisal by Larg & Moss Check list for cost of illness studies

	Ding et al 2016 [36]	Zhang & Chaaban 2013 [41]	Bielemann et al 2015 [45]	Popikin et al 2006 [49]*
Q1(a)	++	++	+	+
Q1(b)	++	+	+	+
Q1(c) i<br>(c) ii<br>(c) iii<br>(c) iv	++<br>+<br>+<br>+(counterfactual population)	+<br>++<br>+<br>NA**	+<br>+<br>+<br>NA	+<br>+<br>NR*<br>NA
Q2(a) i<br>(a)ii<br>(a)iii<br>(a)iv<br>(a)v	-<br>+<br>+<br>+<br>+	-<br>+<br>+<br>-<br>+	-<br>+<br>++<br>+<br>+	-<br>NR<br>NR<br>NR<br>+
Q2(b)i<br>(b)ii<br>(b)iii<br>(b)iv	+<br>NA<br>+<br>+	+<br>NA<br>+<br>+	+<br>NA<br>+<br>+	+<br>NA<br>+<br>-
Q2(c)	+	NR(cost from national data)	NR	NR
Q2(d)	+	NR(cost from national data)	NA	NR
Q2(e)i<br>(e)ii	+<br>++	NR<br>NR	NA<br>NA	NR<br>NR
Q3 (a)	++	++	+	+
Q3(b)	++	-	-	-
Q3 (c)	+	NR	-	NR
Q3(d)i<br>(d)ii<br>(d)iii	+<br>-<br>?	Secondary data<br>Secondary data<br>secondary data	-<br>NR	NR<br>NR<br>NR

For peer review only

			+(CI not reported)NR	
Q3(e)	++	+	+	-
Q3(f)	++	+	-	NR
Q3(g)	++	+	+	+
Q3(h)	++	+	+	+

- Popkin et al case study developed from a review adequate information not available to comment on quality of costing study
- *NR- Not reported
- **NA-Not applicable

For peer review only

2. Summary of quality appraisal by NICE check list for correlations and associations

	Araujo et al 2017[34]	Cheah et al 2017a [35]	Min JY & Min KB 2016 [37]	Turi et al 2015 [38]	Cheah et al 2017b [39]	Codongo et al 2015 [40]	Turi et al 2017 [42]	Silva A.S 2015 [43]	Patel et al 2011 [44]	Thiago et al 2016 [46]	Abdi et al 2014 [47]	Chen et al 2015 [48]	Atkinson et al 2015 [50]
Q1.1	+	+	+	+	+	++	++	++	+	+	+	+	++
Q1.2	-	+	+	+	+	+	+	+	+	+	+	+	++
Q1.3	+	NR	+	+	+	+	+	+	+	+	-	+	++
Q2.1	NA	NA	-	NA	NA	NA	NA	NA	-	NA	NA	NA	NA
Q2.2	+	+	+	+	+	++	+	+	+	+	+	+	+
Q2.3	NA	NA	-	NA	NA	NA	NA	NA	+	NA	NA	NA	NA
Q2.4	+	+	+	+	+	++	+	-	-	-	-	-	+
Q2.5	This question not applicable for this review												
Q3.1	+	-	+	+	-	+	+	+	+	-	+	+	+
Q3.2	+	+	+	+	+	+	+	+	+	+	+	+	+
Q3.3	+	+	+	+	+	+	+	+	+	+	+	+	+
Q3.4	NA	NA	+	NA	NA	NA	NA	NA	+	NA	NA	NA	NA
Q3.5	NA	NA	+	NA	NA	NA	NA	NA	+	NA	NA	NA	NA

Q4.1*	+	+	+	+	+	+	+	+	+	-	+	+	+
Q4.2	+	+	+	+	+	++	+	-	+	-	-	+	+
Q4.3	+	+	+	+	+	++	+	+	+	-	-	+	+
Q4.6	+	+	+	+	+	++	+	+	+	-	-	+	+
Q5.1(IV)	+	+	++	++	++	++	++	++	++	++	+	++	++
Q5.2(EV)	++	++	++	++	++	++	++	++	++	+	+	++	++
Score	(13/13) (2/3),	(12/13) (2/3)	(11/13) (3/3)	(13/13) (3/3)	(12/13) (3/3)	(13/13) (3/3)	(13/13) (3/3)	(11/13) (3/3)	(11/13) (3/3)	(9/13) (3/3)	(9/13) (2/3)	(13/13) (3/3)	(13/13) (3/3)

*Most of the studies power calculations not done. However, large samples have been used

Quality score
Internal Validity >= 10/13=++ (>75%)
6-9/13=+ (50-75%)
0-5/13=- (<50%)

External Validity =3/3 (++),
= 2/3(+)
=≤1/3(-)

For peer review only

Reporting checklist for systematic review and meta-analysis.

Based on the PRISMA guidelines.

Instructions to authors

Complete this checklist by entering the page numbers from your manuscript where readers will find each of the items listed below.

Your article may not currently address all the items on the checklist. Please modify your text to include the missing information. If you are certain that an item does not apply, please write "n/a" and provide a short explanation.

Upload your completed checklist as an extra file when you submit to a journal.

In your methods section, say that you used the PRISMAreporting guidelines, and cite them as:

Moher D, Liberati A, Tetzlaff J, Altman DG, The PRISMA Group. Preferred Reporting Items for Systematic Reviews and Meta-Analyses: The PRISMA Statement

		Reporting Item	Page Number
**Title**			
	#1	Identify the report as a systematic review, meta-analysis, or both.	01
**Abstract**			

For peer review only

Structured summary	#2	Provide a structured summary including, as applicable: background; objectives; data sources; study eligibility criteria, participants, and interventions; study appraisal and synthesis methods; results; limitations; conclusions and implications of key findings; systematic review registration number	02
**Introduction**			
Rationale	#3	Describe the rationale for the review in the context of what is already known.	04
Objectives	#4	Provide an explicit statement of questions being addressed with reference to participants, interventions, comparisons, outcomes, and study design (PICOS).	05/06
**Methods**			
Protocol and registration	#5	Indicate if a review protocol exists, if and where it can be accessed (e.g., Web address) and, if available, provide registration information including the registration number.	03
Eligibility criteria	#6	Specify study characteristics (e.g., PICOS, length of follow-up) and report characteristics (e.g., years considered, language, publication status) used as criteria for eligibility, giving rational	06/07
Information sources	#7	Describe all information sources in the search (e.g., databases with dates of coverage, contact with study authors to identify additional studies) and date last searched.	06

For peer review only

Search	#8	Present full electronic search strategy for at least one database, including any limits used, such that it could be repeated.	Appendix 1
Study selection	#9	State the process for selecting studies (i.e., for screening, for determining eligibility, for inclusion in the systematic review, and, if applicable, for inclusion in the meta-analysis).	06/07
Data collection process	#10	Describe the method of data extraction from reports (e.g., piloted forms, independently by two reviewers) and any processes for obtaining and confirming data from investigators.	07/08
Data items	#11	List and define all variables for which data were sought (e.g., PICOS, funding sources), and any assumptions and simplifications made.	07/08
Risk of bias in individual studies	#12	Describe methods used for assessing risk of bias in individual studies (including specification of whether this was done at the study or outcome level, or both), and how this information is to be used in any data synthesis.	08
Summary measures	#13	State the principal summary measures (e.g., risk ratio, difference in means).	na
Planned methods of analyis	#14	Describe the methods of handling data and combining results of studies, if done, including measures of consistency (e.g., I2) for each meta-analysis.	09

For peer review only

Risk of bias across studies	#15	Specify any assessment of risk of bias that may affect the cumulative evidence (e.g., publication bias, selective reporting within studies).	na
Additional analyses	#16	Describe methods of additional analyses (e.g., sensitivity or subgroup analyses, meta-regression), if done, indicating which were pre-specified.	na
**Results**			
Study selection	#17	Give numbers of studies screened, assessed for eligibility, and included in the review, with reasons for exclusions at each stage, ideally with a flow diagram.	09
Study characteristics	#18	For each study, present characteristics for which data were extracted (e.g., study size, PICOS, follow-up period) and provide the citation.	09/10
Risk of bias within studies	#19	Present data on risk of bias of each study and, if available, any outcome-level assessment (see Item 12).	12
Results of individual studies	#20	For all outcomes considered (benefits and harms), present, for each study: (a) simple summary data for each intervention group and (b) effect estimates and confidence intervals, ideally with a forest plot.	10-17
Synthesis of results	#21	Present the main results of the review. If meta-analyses are done, include for each, confidence intervals and measures of consistency.	17

For peer review only

Risk of bias across studies	#22	Present results of any assessment of risk of bias across studies (see Item 15).	na
Additional analysis	#23	Give results of additional analyses, if done (e.g., sensitivity or subgroup analyses, meta-regression [see Item 16]).	na
**Discussion**			
Summary of Evidence	#24	Summarize the main findings, including the strength of evidence for each main outcome; consider their relevance to key groups (e.g., health care providers, users, and policy makers	18-20
Limitations	#25	Discuss limitations at study and outcome level (e.g., risk of bias), and at review level (e.g., incomplete retrieval of identified research, reporting bias).	20
Conclusions	#26	Provide a general interpretation of the results in the context of other evidence, and implications for future research.	21
**Funding**			
Funding	#27	Describe sources of funding or other support (e.g., supply of data) for the systematic review; role of funders for the systematic review.	21

None The PRISMA checklist is distributed under the terms of the Creative Commons Attribution License CC-BY. This checklist can be completed online using https://www.goodreports.org/, a tool made by the EQUATOR Network in collaboration with Penelope.ai

For peer review only

Open access Protocol

BMJ Open

The economics of physical activity in low-income and middle-income countries: protocol for a systematic review

Priyanga Diloshini Ranasinghe,[1,2] Subhash Pokhrel,[3] Nana Kwame Anokye[3]

To cite: Ranasinghe PD, Pokhrel S, Anokye NK. The economics of physical activity in low-income and middle-income countries: protocol for a systematic review. *BMJ Open* 2019;**9**:e022686. doi:10.1136/bmjopen-2018-022686

► Prepublication history and additional material for this paper are available online. To view these files, please visit the journal online (http://dx.doi.org/10.1136/bmjopen-2018-022686).

Received 5 March 2018
Revised 9 October 2018
Accepted 23 November 2018

Check for updates

© Author(s) (or their employer(s)) 2019. Re-use permitted under CC BY-NC. No commercial re-use. See rights and permissions. Published by BMJ.

[1]Department of Health, Ministry of Health, Nutrition and Indigenous Medicine, Colombo, Sri Lanka
[2]Division of Health Sciences, Department of Clinical Sciences, Collage of Health and Life Sciences, Brunel University, London, UK
[3]Health Economics Theme, Institute of Environment, Health and Societies, Brunel University, London, UK

Correspondence to
Dr Priyanga Diloshini Ranasinghe; priyangaran@yahoo.com, Priyanga.Ranasinghe@brunel.ac.uk

ABSTRACT

Introduction Evidence on the economic costs of physical inactivity and the cost-effectiveness of physical activity interventions in low-income and middle-income countries (LMICs) is sparse, and fragmented where they are available. This is the first review aimed to summarise available evidence on economics of physical activity in LMICs, identify potential target variables for policy, and identify and report gaps in the current knowledge on economics of physical activity in LMICs.

Methods and analysis Peer-reviewed journal articles of observational, experimental, quasi-experimental and mixed-method studies on economics of physical activity in LMICs will be identified by a search of electronic databases; Scopus, Web of Science and SPORTDiscus. Websites of WHO, the National Institute for Health and Care Excellence international, World Bank and reference lists of included studies will be searched for relevant studies. The study selection process will be a two-stage approach; title and abstract screen for inclusion, followed by a review of selected full-text articles by two independent reviewers. Disagreements will be resolved by consensus and discussion with a third reviewer. Data will be extracted using standardised piloted data extraction forms. Risk of bias will be critically appraised using standard checklists based on study designs. Descriptive synthesis of data is planned. Where relevant, summaries of studies will be classified according to type of economic analysis, country or country category, population, intervention, comparator, outcome and study design. Meta-analysis will be performed where appropriate. This protocol for systematic review is prepared according to the Preferred Reporting Items for Systematic review and Meta-analysis for Protocols –2015 statement.

Ethics and dissemination Ethical approval is not obtained as original data will not be collected as part of this review. The completed review will be submitted for publication in a peer-reviewed journal and presented at conferences.

PROSPERO registration number CRD42018099856.

Strengths and limitations of this study

- This is the first synthesis of literature on economics of physical activity in low-income and middle-income countries (LMICs).
- This study provides evidence-based recommendations for economic research practice on physical activity.
- This review presents evidence on the business case for physical activity interventions for decision making by policy makers in LMIC settings.
- This review could suffer from publication bias as it excludes studies that are not peer reviewed.
- This review focuses on studies written in the English language and could miss out on relevant literature published in other languages.

INTRODUCTION

Rationale

Physical inactivity is a global health challenge, the fourth leading cause of mortality worldwide[1] and the key risk factor for non-communicable diseases (NCDs) such as cardiovascular diseases, cancer and diabetes.[2] Almost a third of the global health burden comprises NCDs which account for 1.4 billion disability-adjusted life years (DALYs).[3] At present most of the global NCD burden is from low-income and middle-income countries (LMICs) (as defined by[4] the World Bank, Classification 2017)[4] and this burden is largely attributable to levels of physical inactivity in the population.[5 6] Prevalence of physical inactivity in 2010 in upper middle-income countries was 25.4% (19.1%–33.7%), followed by 16.8% (11.8%–26.4%)%) in lower middle-income countries and 16.6% (11.8%–26.4%)%) in low-income countries showing that large populations in LMICs[7] are affected by physical inactivity and are therefore at risk of developing NCDs. The data available for the year 2004 show that 3.2 million deaths worldwide were due to physical inactivity of which 2.6 million were reported from LMICs.[8] LMICs have experienced urbanisation, shift of occupations from agriculture to industrial practice and modernisation including automation, and this has resulted in increased levels of physical inactivity in these populations.[9]

Increasing physical activity and reducing the burden of NCDs is the main goal of public health policies in LMICs.[10] It is a personal, corporate and government responsibility to

For peer review only - http://bmjopen.bmj.com/site/about/guidelines.xhtml

BMJ Open: first published as 10.1136/bmjopen-2018-022686 on 17 January 2019. Downloaded from http://bmjopen.bmj.com/ on February 9, 2020 at Sri Lanka:BMJ-PG Sponsored. Protected by copyright.

reduce the economic burden of unhealthy lifestyles in LMICs.[10] Economic analysis could uncover subtle characteristics of individual decision making on lifestyle choices, estimate the opportunity costs of not doing anything to improve population levels of physical activity and help evaluate what interventions work efficiently to achieve the goal of addressing the physical inactivity pandemic, particularly in LMICs.[11] The current focus of promoting physical activity has shifted from the traditional education approach to environment and policy approaches. Economic perspectives and public health perspectives complement each other in promotion of physical activity.[12] Economic perspectives inform the design of effective physical activity interventions (identifying economic barriers to physical activity) and the efficient allocation of resources—critical to the economies of LMICs.[12]

Our scoping exercise did not identify any published reviews that address the economics of physical activity in LMICs. Thus economic evidence on physical activity in LMICs could be limited or scattered.[13] Therefore, we designed this review to address the following research questions: (1) What is the available evidence base and research gaps on the economics of physical activity in LMICs? (2) What is the focus of and methods for underpinning economic research on physical activity in LMICs? (3) What are the target variables and cost-effective interventions for physical activity policy in LMICs?

This review will add to the scientific literature, provide an overview of the economic evidence base of physical activity in LMICs and fill the gaps in the available evidence regarding this. Providing an up-to-date synthesis of the economic evidence base is an efficient way of highlighting current research practices and new findings to inform researchers, and formulation of cost-effective physical activity programmes and policies.

Objectives

The review will:

- Summarise available evidence on economics of physical activity in LMICs.
- Describe the focus and methods underpinning research on economics of physical activity in LMICs.
- Identify potential target variables and cost-effective interventions for physical activity in LMICs for policy formation.
- Identify and report gaps in research on economics of physical activity in LMICs to provide recommendations for the economic research agenda in LMICs.

METHODS

The methods for this review were informed by previous reviews of economic analyses of physical activity[10 13–19] and are in line with recommendations on review of economic evidence.[20–22] This will be based on the 'Preferred Reporting Items for Systematic Review and Meta Analyses Protocols (PRISMA-P)' Statement[23] (online supplementary file 1).

Eligibility criteria

Economic studies on physical activity in LMICs will be included. Specifically, the following study types will be included: (1) Economic evaluations of physical activity interventions. (2) Economic burden of physical inactivity. (3) Cost of physical activity participation. (4) Demand for physical activity. (5) Economic correlates of physical (in) activity. Physical activity is defined as any bodily movement produced by skeletal muscles that require energy expenditure—including activities undertaken while walking, playing, carrying out household chores, travelling and engaging in recreational pursuits.[24] Physical inactivity is considered as a lack of physical activity.[25]

Physical activity interventions will not be specifically defined for this review in order to identify any physical activity intervention in LMICs. However, studies of which an economic analysis was carried out along with the intervention will be included in this review. This strategy will enable consolidation of all available evidence on economics of physical activity related to physical activity interventions.

Eligibility criteria are determined by relevant elements of the population, intervention, comparator, outcome, study design (PICOS criteria).[26] Interventions and comparators will be applicable only to intervention studies.

Inclusion criteria

- Study setting: Any setting of LMICs in accordance with the definition of LMIC by World Bank, Classification 2017.[4]
- Population: Any population in any age group across the life course.
- Intervention: Any physical activity intervention in which the economic evaluation of the intervention has been carried out.
- Comparator: Normal routine, no intervention.
- Outcomes: (1) Cost-effectiveness ratio, quality-adjusted life years (QUALY), incremental cost-effectiveness ratio assessed as the outcomes of physical activity interventions. (2) Cost of physical (in) activity in terms of healthcare cost and/or productivity loss and/or total cost of physical inactivity. (3) Measures of association of any economic variable with physical activity are defined as the primary outcomes of this review.
- Study design: Observational studies (cohort, case control, cross-sectional); correlational studies, experimental studies including randomised controlled trials; quasi-experimental studies; natural experiments; and economic evaluation studies.
- Studies reported only in the English language.

Exclusion criteria

- Case reports, case series, letters to the editor, editorials, reviews, qualitative studies, unpublished theses, conference abstracts and any unobtainable texts.
- Studies published in a language other than English.

BMJ Open: first published as 10.1136/bmjopen-2018-022686 on 17 January 2019. Downloaded from http://bmjopen.bmj.com/ on February 9, 2020 at Sri Lanka:BMJ-PG Sponsored. Protected by copyright.

For peer review only - http://bmjopen.bmj.com/site/about/guidelines.xhtml

Search strategy

The selection of databases was performed by a scoping review of methods of systematic reviews in the field.[13 17 18 27 28] We will search the following electronic databases to identify studies: Scopus (covers 100% MEDLINE coverage, 100% of EMBASE coverage and 100% of Compendex coverage[29]), Web of Science and SPORTDiscus. Websites of WHO, National Institute for Health and Care Excellence (NICE) international and World Bank will be searched for relevant studies. The reference lists of included studies will be searched for any relevant articles. Searches will include publications up to December 2017.

The search strategy was developed based on the scoping review that covered relevant reviews on economic studies on physical activity,[10 13–19] reviews on physical activity[27 30 31] and reviews on economic evaluations.[28 32 33] The draft search strategy was then reviewed by subject experts and a subject liaison librarian to optimise the sensitivity and specificity of the search. Online supplementary file 2 shows a sample search strategy.

Study selection

A two-stage approach involving three independent reviewers will be used to select relevant papers.

At the first stage, two reviewers (PDR and NKA) will independently screen the titles and abstracts of identified papers. Disagreement will be resolved through discussions with a third reviewer (SP). We will include a study if in doubt about its inclusion. For example, if the country setting of a study is unclear in the title and abstract, we will take it forward to the next stage.

At the second stage, the full text of papers selected from stage 1, will be reviewed independently by two reviewers (PDR and NKA). Selected and excluded papers, with reasons, will be discussed by the two reviewers and a third reviewer (SP) at a consensus meeting and disagreement will be resolved by real time consensus. Corresponding authors will be contacted via email for clarification if needed. The study selection process will be presented in a PRISMA flow chart[34] along with the reasons for the exclusion of studies.

Data management and extraction

Endnote X7 software will be used to manage the search results. Data extraction will be performed using a standardised pilot-tested data extraction form developed based on relevant data extraction forms from relevant reviews.[17 35 36] The adaptation process involved matching the review objectives with the available data extraction forms. Data from the final selected full-text articles will be extracted by one reviewer (PDR). To ensure quality of data extraction, a second reviewer (NKA) will independently extract data from a random selection of 50% of the final articles. Disagreements between the two reviewers will be discussed and resolved through discussions with the third reviewer (SP). Any unresolved disagreements will be reported in the final report. Online supplementary file 3 shows the draft data extraction form. Data will be extracted for the following items: general information, characteristics of the study, characteristics of the population/condition/intervention, data sources/data analysis/outcomes, conclusions and the way forward suggested by the authors, challenges and quality assessment.

Risk of bias and quality assessment

Risk of bias will be assessed by a one reviewer (PDR) for all the selected articles using standard checklists based on the study design. A second reviewer (NKA) will also independently assess the risk of bias on 50% of the randomly selected articles included.

Checklists are considered a reliable means of ensuring that all studies included are critically appraised in a standard way.[26] These will assess the information bias which could occur due to the methods, study design, data collection, data analysis and interpretation, and selection bias, where relevant. The most appropriate checklists for each expected study design for risk of bias assessment were selected based on relevant literature in this area[15 17 36–40] and by consensus with all three reviewers (online supplementary file 4). Risk of bias will be assessed using the following tools:

- Economic evaluation studies
  The Drummond checklist[41] which consists of 35 items will be used. It mainly assesses the quality of the study in relation to methods, study design, data collection, data analysis and interpretations. If an economic evaluation study is based on a decision analytical model, the Phillips[42] checklist will be used in addition.
- Cost of illness studies
  The Larg & Moss checklist[43] will be used to assess the quality of the study. It would assess the possible biases related to the analytical framework: what costs should have been measured, methodology and data; how well were resources used and productivity losses measured including representativeness of data for the study population, analysis and reporting. Both internal validity and external validity will be appraised.
- Studies on correlation or association
  The checklist recommended by NICE[44] will be used. Risk of bias related to sample selection, method of selection of exposure/comparison group, outcomes and analysis will be assessed. Both internal and external validity will be appraised.

Quality grading will be given to each included study based on the overall quality of the study. Grading for internal validity and external validity will be reported separately, where relevant. The grading process will follow the NICE recommendations[37] (figure 1):

- Good quality (++): almost all checklist criteria have been fulfilled; where they have not been fulfilled the conclusions are very unlikely to alter.
- Moderate quality (+): some of the checklist criteria are fulfilled; where they have not been fulfilled or not adequately described, conclusions are unlikely to alter.

For peer review only

For peer review only - http://bmjopen.bmj.com/site/about/guidelines.xhtml

BMJ Open: first published as 10.1136/bmjopen-2018-022686 on 17 January 2019. Downloaded from http://bmjopen.bmj.com/ on February 9, 2020 at Sri Lanka:BMJ-PG Sponsored. Protected by copyright.

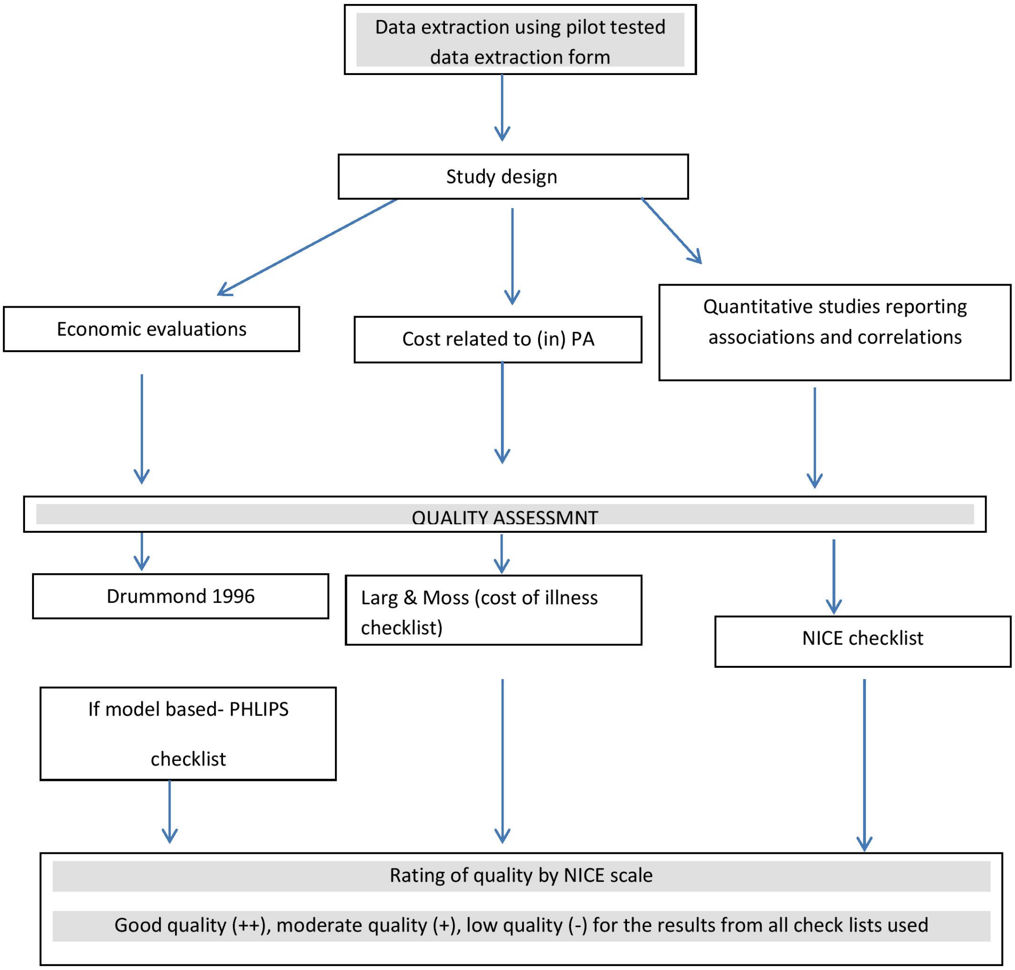

Figure 1 Economics of physical activity (PA) in low-income and middle-income countries (LMICs)—plan for data extraction and the quality assessment process. NICE, National Institute for Health and Care Excellence.

- Low quality (−): few or no checklist criteria are fulfilled, and conclusions are likely or very likely to alter.
- The quality of the included studies will be reported and critically appraised for each study. However, studies will not be excluded based on quality, as this review is particularly aimed to capture the evidence base on economics of physical activity in LMICs and appraise the methods underpinning the evidence base.

Data synthesis and reporting

A descriptive synthesis of data is planned due to the expected heterogeneity of the studies. Summaries of studies will be classified according to type of economic analysis, country or country category, characteristics of the study by PICOS criteria (where applicable); population, intervention, comparator, outcome, study design and quality of studies to describe the overview of available economic evidence base of physical activity in LMICs. Where the numbers permit, summaries of the studies will be categorised based on the focus of the study; economic burden of physical inactivity, economic analysis of physical activity interventions, economic correlates of physical activity including association of physical activity and cost (healthcare cost and/or productivity loss). Where appropriate, we will conduct a meta-analysis to quantify the effect sizes. Such analyses will adjust for between-study heterogeneity using random-effects models. The I-squared (I^2) statistic will be used to evaluate the heterogeneity related to the findings of the papers. Quantitative analysis will be conducted using STATA V.13 software.

We will report the number of studies where data extraction items were not applicable, as an indicator of the quality of reporting. Author-stated limitations of included studies and recommendations for future research and policy will be presented. This review will be reported in accordance with the PRISMA 2009 statement.[34] The study selection process will be illustrated by a flow diagram that will include the reasons for exclusion of studies at each stage. The search strategy, data extraction forms and quality assessment tools will be published as online supplementary files. This protocol will not be amended and any changes will be described and discussed in the final report.

BMJ Open: first published as 10.1136/bmjopen-2018-022686 on 17 January 2019. Downloaded from http://bmjopen.bmj.com/ on February 9, 2020 at Sri Lanka:BMJ-PG Sponsored. Protected by copyright.

For peer review only - http://bmjopen.bmj.com/site/about/guidelines.xhtml

Study status

A scoping review of economics of physical activity in LMICs was carried out in November 2017 to inform the methods of the current review. It identified systematic reviews conducted on economics of physical activity and the gaps in knowledge. It has not been submitted for publication. A review of economics of physical activity in LMICs was designed and methods were developed in December 2017. The search strategy was piloted in January 2018 and the protocol was developed in February 2018. The first submission of the protocol for peer review was on 1 March 2018. Data collection will be commenced upon approval of the protocol for publication and will be completed in 2 months.

Patient and public involvement

Patients and public are not directly involved in this study as original data will not be collected.

Best practice guidelines to conduct systematic reviews[33] will be followed.

DISCUSSION

The level of physical inactivity of the population in LMICs is likely to increase giving rise to a multitude of public health and economic consequences, including rising healthcare costs due to increased NCD-related treatments, increased DALYs and productivity losses. Economic research on physical activity from the perspective of LMICs should therefore be considered a priority. This is because increased physical inactivity leads to uncontrolled healthcare needs in LMICs, where resources (including that for healthcare) are limited and often shrinking in real terms. Pooling of the available research evidence on economics of physical activity in LMICs will thus reveal the current knowledge on economics of physical activity in LMICs, thereby guiding the future of national, regional and local policies around physical activity.

This will be the first study to review the evidence base on economics of physical activity in the context of LMICs which can be considered as the main strength of this study. This will identify gaps in knowledge to support future studies in this area. However, we will only include those studies that are published as peer-reviewed articles and are published in the English language. Thus, possibility of publication bias and language bias cannot be excluded. Furthermore, by assessing and reporting the quality of included studies using standard quality assessment checklists, the possibility of reporting bias of this review can be minimised.

WHO is in the process of drafting the 'Global action plan on physical activity (GAPPA) to be implemented from 2018 to 2030'.[45] This is a timely study as the evidence from this research will provide useful information for implementation and prioritise the actions of GAPPA in LMICs. Evidence from this research will be useful for policy makers and stakeholders dealing with physical activity promotion at individual, local, national, regional and global levels, with special reference to LMICs. Further, this review will be an important base for a research agenda on economics of physical activity in LMICs. This will be a useful guide for researchers to design research on economics and physical activity with sound methodology, based on the research needs of LMICs. Furthermore, information gathered from this research will guide funding agencies for effective allocation of resources.

Contributors SP and NKA developed the idea for the review with inputs from PDR. PDR wrote the first draft. SP and NKA revised the protocol. NKA will act as guarantor of the review.

Funding The authors have not declared a specific grant for this research from any funding agency in the public, commercial or not-for-profit sectors.

Competing interests None declared.

Patient consent for publication Not required.

Provenance and peer review Not commissioned; externally peer reviewed.

Open access This is an open access article distributed in accordance with the Creative Commons Attribution Non Commercial (CC BY-NC 4.0) license, which permits others to distribute, remix, adapt, build upon this work non-commercially, and license their derivative works on different terms, provided the original work is properly cited, appropriate credit is given, any changes made indicated, and the use is non-commercial. See: http://creativecommons.org/licenses/by-nc/4.0/.

REFERENCES

1. World Health Organization. *Global recommendations on physical activity for health*. Geneva: World Health Organization, 2010.
2. World Health Organization. Physical activity [Fact Sheet – Media centre]. 2017 http://www.who.int/mediacentre/factsheets/fs385/en/ (Accessed 6 Feb 2018).
3. Murray CJ, Barber RM, Foreman KJ, *et al*. Global, regional, and national disability-adjusted life years (DALYs) for 306 diseases and injuries and healthy life expectancy (HALE) for 188 countries, 1990-2013: quantifying the epidemiological transition. *Lancet* 2015;386:2145–91.
4. Bank W. World bank country and lending groups: country classification. 2017 https://datahelpdesk.worldbank.org/knowledgebase/articles/906519-world-bank-country-and-lending-groups (Accessed 1st Dec 2017).
5. Abegunde DO, Mathers CD, Adam T, *et al*. The burden and costs of chronic diseases in low-income and middle-income countries. *Lancet* 2007;370:1929–38.
6. Hallal PC, Andersen LB, Bull FC, *et al*. Global physical activity levels: surveillance progress, pitfalls, and prospects. *Lancet* 2012;380:247–57.
7. WHO. Prevalence of insufficient physical activity among adults, data by world bank income groups. 2015 http://apps.who.int/gho/data/view.main.2487?lang=en (Accessed 29th Jan 2018).
8. WHO. Global health risks: mortality and burden of disease attributable to selected major risks. ISBN 978 92 4 156387 1. 2009. Retrieved from http://www.who.int/healthinfo/global_burden_disease/GlobalHealthRisks_report_full.pdf (Accessed 25th Jan 2018).
9. Atkinson K, Lowe S, Moore S. Human development, occupational structure and physical inactivity among 47 low and middle income countries. *Prev Med Rep* 2016;3:40–5.
10. Oldridge NB. Economic burden of physical inactivity: healthcare costs associated with cardiovascular disease. *Eur J Cardiovasc Prev Rehabil* 2008;15:130–9.
11. Islam SM, Purnat TD, Phuong NT, *et al*. Non-communicable diseases (NCDs) in developing countries: a symposium report. *Global Health* 2014;10:81.
12. Sturm R. Economics and physical activity: a research agenda. *Am J Prev Med* 2005;28:141–9.
13. Ding D, Kolbe-Alexander T, Nguyen B, *et al*. The economic burden of physical inactivity: a systematic review and critical appraisal. *Br J Sports Med* 2017;51:1392–409.
14. Barte JCM, Wendel-Vos GCW. A systematic review of financial incentives for physical activity: the effects on physical activity and related outcomes. *Behav Med* 2017;43:79–90.
15. Abu-Omar K, Rütten A, Burlacu I, *et al*. The cost-effectiveness of physical activity interventions: a systematic review of reviews. *Prev Med Rep* 2017;8:72–8.

For peer review only

For peer review only - http://bmjopen.bmj.com/site/about/guidelines.xhtml

BMJ Open: first published as 10.1136/bmjopen-2018-022686 on 17 January 2019. Downloaded from http://bmjopen.bmj.com/ on February 9, 2020 at Sri Lanka:BMJ-PG Sponsored. Protected by copyright.

16. Brown V, Zapata Diomedi B, Moodie M, *et al*. A systematic review of economic analyses of active transport interventions that include physical activity benefits. *BMJ Open* 2016;45.
17. Pavey TG, Taylor AH, Fox KR, *et al*. Effect of exercise referral schemes in primary care on physical activity and improving health outcomes: systematic review and meta-analysis. *BMJ* 2011;343:d6462.
18. Campbell F, Holmes M, Everson-Hock E, *et al*. A systematic review and economic evaluation of exercise referral schemes in primary care: a short report. *Health Technol Assess* 2015;19:1–110.
19. Suhrcke M, Boluarte TA, Niessen L. A systematic review of economic evaluations of interventions to tackle cardiovascular disease in low- and middle-income countries. *BMC Public Health* 2012;12:2.
20. Kaltenthaler E, Tappenden P, Paisley S. Reviewing the evidence to inform the population of cost-effectiveness models within health technology assessments. *Value Health* 2013;16:830–6.
21. Zechmeister-Koss I, Schnell-Inderst P, Zauner G. Appropriate evidence sources for populating decision analytic models within health technology assessment (HTA): a systematic review of HTA manuals and health economic guidelines. *Med Decis Making* 2014;34:288–99.
22. NICE. *The guideline manual process and methods (PMG6)*: NICE, 2012.
23. Moher D, Shamseer L, Clarke M, *et al*. Preferred reporting items for systematic review and meta-analysis protocols (PRISMA-P) 2015 statement. [Research Support, Non-U S Gov't Research Support, U S Gov't, P H S]. *Syst Rev* 2015;4:2046–4053.
24. WHO. *Physical Activity*. [Fact sheet]. 2018 http://www.who.int/en/news-room/fact-sheets/detail/physical-activity (Accessed 15th Jan 2018).
25. WHO. Global Strategy on Diet, Physical activity and Health. 2018. Retrieved from https://www.who.int/dietphysicalactivity/pa/en/ (Accessed 6th Oct 2018).
26. University of York. *Systematic reviews: CRD's guidance for undertaking reviews in health care*. York: University of York, 2009.
27. Milton K, Macniven R, Bauman A. Review of the epidemiological evidence for physical activity and health from low- and middle-income countries. *Glob Public Health* 2014;9:369–81.
28. Nosratnejad S, Rashidian A, Dror DM. Systematic review of willingness to pay for health insurance in low and middle income countries. *PLoS One* 2016;11:e0157470.
29. Burnham JF. Scopus database: a review. *Biomed Digit Libr* 2006;3:1742–5581.
30. Lachat C, Otchere S, Roberfroid D, *et al*. Diet and physical activity for the prevention of noncommunicable diseases in low- and middle-income countries: a systematic policy review. *PLoS Med* 2013;10:e1001465.
31. Hunter RF, Christian H, Veitch J, *et al*. The impact of interventions to promote physical activity in urban green space: a systematic review and recommendations for future research. *Soc Sci Med* 2015;124:246–56.
32. Sutton L, Karan A, Mahal A. Evidence for cost-effectiveness of lifestyle primary preventions for cardiovascular disease in the Asia-Pacific Region: a systematic review. *Global Health* 2014;10:79.
33. Santatiwongchai B, Chantarastapornchit V, Wilkinson T, *et al*. Methodological variation in economic evaluations conducted in low- and middle-income countries: information for reference case development. *PLoS One* 2015;10:e0123853.
34. Moher D, Liberati A, Tetzlaff J, *et al*. Preferred reporting items for systematic reviews and meta-analyses: the PRISMA statement. [Guideline Research Support, Non-U S Gov't]. *J Clin Epidemiol* 2009;62:1006–12.
35. Pokhrel S, Quigley MA, Fox-Rushby J, *et al*. Potential economic impacts from improving breastfeeding rates in the UK. [Research Support, Non-U.S. Gov't]. *Arch Dis Child* 2015;100:334–40.
36. Murthy S, John D, Godinho IP, *et al*. A protocol for a systematic review of economic evaluation studies conducted on neonatal systemic infections in South Asia. *Syst Rev* 2017;6:252.
37. Aminde LN, Veerman L. Interventions for the prevention of cardiovascular diseases: a protocol for a systematic review of economic evaluations in low-income and middle-income countries. *BMJ Open* 2016;6:e013668.
38. Ruifrok AE, Rogozinska E, van Poppel MN, *et al*. Study protocol: differential effects of diet and physical activity based interventions in pregnancy on maternal and fetal outcomes--individual patient data (IPD) meta-analysis and health economic evaluation. *Syst Rev* 2014;3:131.
39. Gc V, Wilson EC, Suhrcke M, *et al*. Are brief interventions to increase physical activity cost-effective? A systematic review. *Br J Sports Med* 2016;50:408–17.
40. Tonmukayakul U, Calache H, Clark R, *et al*. Systematic review and quality appraisal of economic evaluation publications in dentistry. *J Dent Res* 2015;94:1348–54.
41. Drummond MF, Jefferson TO. Guidelines for authors and peer reviewers of economic submissions to the BMJ. *BMJ* 1996;313:275–83.
42. Philips Z, Ginnelly L, Sculpher M, *et al*. Review of guidelines for good practice in decision-analytic modelling in health technology assessment. *Health Technol Assess* 2004;8:1–158.
43. Larg A, Moss JR. Cost-of-illness studies: a guide to critical evaluation. [Research Support, Non-U S Gov't]. *Pharmacoeconomics* 2011;29:653–71.
44. National Institute for Health and Excellence (NICE). Methods for the development of NICE public health guidance (third edition), Appendix G Quality appraisal checklist-quantitative studies reporting correlations and associations. 2012 https://www.nice.org.uk/process/pmg4/chapter/appendix-g-quality-appraisal-checklist-quantitative-studies-reporting-correlations-and (Accessed 2nd Jan 2018).
45. World Health Organization. *Non communicable diseases and their risk factors, governance*: Development of draft global action plan to promote physical activity, 2018.

For peer review only

BMJ Open: first published as 10.1136/bmjopen-2018-022686 on 17 January 2019. Downloaded from http://bmjopen.bmj.com/ on February 9, 2020 at Sri Lanka:BMJ-PG Sponsored. Protected by copyright.

For peer review only - http://bmjopen.bmj.com/site/about/guidelines.xhtml

BMJ Open

The economics of physical activity in low and middle income countries: systematic review

Journal:	*BMJ Open*
Manuscript ID	bmjopen-2020-037784.R1
Article Type:	Original research
Date Submitted by the Author:	06-Sep-2020
Complete List of Authors:	Ranasinghe, Priyanga; Ministry of Health, Nutrition and Indigenous Medicine, ; Brunel Univeristy London, Clinical Sciences<br>Pokhrel, Subhash; Brunel University London, Health Economics Research Group<br>Anokye, Nana; Brunel University, Health Economics Research Group
**Primary Subject Heading**:	Health economics
Secondary Subject Heading:	Health economics, Public health, Sports and exercise medicine, Health policy
Keywords:	HEALTH ECONOMICS, PREVENTIVE MEDICINE, PUBLIC HEALTH, SPORTS MEDICINE, Health policy < HEALTH SERVICES ADMINISTRATION & MANAGEMENT

SCHOLARONE™
Manuscripts

I, the Submitting Author has the right to grant and does grant on behalf of all authors of the Work (as defined in the below author licence), an exclusive licence and/or a non-exclusive licence for contributions from authors who are: i) UK Crown employees; ii) where BMJ has agreed a CC-BY licence shall apply, and/or iii) in accordance with the terms applicable for US Federal Government officers or employees acting as part of their official duties; on a worldwide, perpetual, irrevocable, royalty-free basis to BMJ Publishing Group Ltd ("BMJ") its licensees and where the relevant Journal is co-owned by BMJ to the co-owners of the Journal, to publish the Work in this journal and any other BMJ products and to exploit all rights, as set out in our licence.

The Submitting Author accepts and understands that any supply made under these terms is made by BMJ to the Submitting Author unless you are acting as an employee on behalf of your employer or a postgraduate student of an affiliated institution which is paying any applicable article publishing charge ("APC") for Open Access articles. Where the Submitting Author wishes to make the Work available on an Open Access basis (and intends to pay the relevant APC), the terms of reuse of such Open Access shall be governed by a Creative Commons licence – details of these licences and which Creative Commons licence will apply to this Work are set out in our licence referred to above.

Other than as permitted in any relevant BMJ Author's Self Archiving Policies, I confirm this Work has not been accepted for publication elsewhere, is not being considered for publication elsewhere and does not duplicate material already published. I confirm all authors consent to publication of this Work and authorise the granting of this licence.

For peer review only

Title page

The economics of physical activity in low and middle income countries: systematic review

Priyanga Diloshini Ranasinghe [2], Subhash Pokhrel[1], Nana Kwame Anokye[1]

[1] Health Economics Theme, Institute of Environment, Health and Societies, Brunel University, London, United Kingdom

[2] Ministry of Health, Nutrition and Indigenous Medicine, Suwasiripaya, Colombo 10, Sri Lanka /Division of Health Sciences, Department of Clinical Sciences, Collage of Health and Life Sciences, Brunel University, London, United Kingdom

Corresponding author: Priyanga Diloshini Ranasinghe

Postal address: Mary Seacole 2nd floor, Division of Health Sciences, Department of Clinical Sciences, Collage of Health and Life Sciences, Brunel University, London, United Kingdom

Email: priyangaran@yahoo.com/ Priyanga.Ranasinghe@brunel.ac.uk

Telephone number: 0094 777552794/0044 7424515136

Subhash Pokhrel Subhash.Pokhrel@brunel.ac.uk

Nana Kwame Anokye Nana.Anokye@brunel.ac.uk

For peer review only

For peer review only - http://bmjopen.bmj.com/site/about/guidelines.xhtml

Keywords: low- and middle-income countries; health economics; physical activity

Word count:5006 (including abstract and summary)

Abstract

Background: Physical activity promotion among populations is a global health investment. However, evidence on economic aspects of physical activity, are sparse and scattered in low- and middle-income countries (LMIC).

Objective: To summarise available evidence on economics of physical activity (PA) in LMICs, identify potential target variables for policy, and report gaps in the existing economic evidence alongside research recommendations.

Data sources: A systematic review of the electronic databases (Scopus, Web of Science and SPORTDiscus) and grey literature.

Study eligibility-criteria: Cost of illness studies, economic evaluations, interventions and descriptive studies on economic factors associated with PA using pre-set eligibility criteria

Study appraisal and synthesis of methods: Screening, study selection, quality appraisal based on standard checklists performed by two reviewers with consensus of a third reviewer. Descriptive synthesis of data performed.

Results: Majority of the studies were from upper-middle-income countries (n=16,88.8%), and mainly from Brazil (n=9,50%). Only one economic

For peer review only - http://bmjopen.bmj.com/site/about/guidelines.xhtml

evaluation study was found. The focus of the reviewed literature spanned the economic burden of physical inactivity (n=4,22%), relationship between PA and costs (n=6,46%), and socioeconomic determinants of PA (n=7,39%). The findings showed a considerable economic burden due to insufficient PA, with LMIC accounting for 75% DALYs globally due to insufficient PA. Socioeconomic correlates of PA were identified and inverse relationship of PA with cost of chronic diseases was established. Regular PA along with drug treatment as a treatment scheme for chronic diseases, showed advantageous with a cost utility ratio of $3.21 /QALY compared to drug treatment only group ($3.92 /QALY) by the only economic evaluation conducted in LMIC; Brazil.

Limitations: Meta-analysis not performed due to heterogeneity of the studies.

Conclusions & recommendations: Economic evaluation studies for physical activity promotion interventions/strategies and local research from low income countries are grossly inadequate. Setting economic research agenda in LMIC ought to be prioritized in those areas.

For peer review only - http://bmjopen.bmj.com/site/about/guidelines.xhtml

PROSPERO registration number: CRD42018099856

Strengths and limitations of this study

- This is the first synthesis of literature on economics of physical activity in LMICs.
- This study provides evidence-based recommendations for research practice on economics of physical activity.
- This review could suffer from publication bias as it excludes studies that are not peer reviewed.
- This review focuses on studies written in the English language and could miss out on relevant literature published in other languages.
- This review not conducted meta-analysis due to wide variation in focus and methods of included studies

For peer review only - http://bmjopen.bmj.com/site/about/guidelines.xhtml

Introduction

Regular physical activity is proven to have multiple health benefits including preventing and treating non communicable diseases (NCD), as well as improving mental health and quality of life [1]. Of all lifestyle behaviours, physical activity has the largest positive impact on the risk of all-cause mortality (33% reduction) [2]. Insufficient physical activity accounts for 3.2 million deaths annually (including 9% premature deaths) and in 2010, 69.3 million DALYs were attributed to physical inactivity in worldwide [3]. Non communicable diseases are a major global health challenge and insufficient physical activity is estimated to cause a large share of the burden of main NCDs; 6% (3.2% to 7.8%) of chronic heart diseases burden, 7% (3.9% to 9.6%) of type II diabetes, 10%, of breast cancer(5.6% to 14.1%) and 10% (5.7% to 13.8%) of colon cancer burden [4]. NCD's causes deaths than all other causes of mortality combined and the trend is expected to increase, with a projected increase from 38 million in 2012 to 52 million by 2030[3]. WHO reports that NCD s are disproportionately rising in LMIC and in 2016 over three quarter of NCD deaths (31.5 million) occurred in LMIC of which about 46% of deaths occurred before the age of 70 in these countries[5].

Physical activity (PA) is defined by World Health Organization (WHO) as "any bodily movement produced by skeletal muscles that requires energy expenditure – including activities undertaken while working, playing, carrying out household chores, travelling, and engaging in recreational pursuits" [6]. WHO recommends at least, 150 min moderate intensity PA or 75min vigorous intensity PA throughout the week for adults/elderly and 60 min moderate to vigorous intensity PA daily for 5-17-year-old children [6]. Insufficient physical activity (IPA) or physical inactivity is defined as absence or insufficient level of physical activity required to meet the current physical activity recommendations [1].

For peer review only - http://bmjopen.bmj.com/site/about/guidelines.xhtml

The prevalence of insufficient PA among adults in 2016 was 27.5% (25·0–32.2%) globally, 16·2% (14·2–17·9%) in low income countries and 26·0% (22·6–31·8) in middle income countries [7].The rate was even higher among school-going adolescents in low income countries (84·9%) and middle income countries (81%) [8]. Prevalence of insufficient PA has been stagnant over last 15 years in these countries [7,8]

To achieve the WHO target of 10% relative reduction in the prevalence of insufficient PA by 2025 in these countries requires urgent, physical activity promotion. To date, progress of implementation has been plagued by lack of awareness and investment [1]. This is partially attributable to the paucity of evidence base on the economic gains accruable from promoting physical activity in LMIC settings [9,10]. There is an unmet demand for an evidence base on economics of physical activity in LMIC which can be used as an advocacy tool to prioritize physical activity promotion. This is critical especially in LMIC, given the unlimited competing health needs and developing economies. Whilst there few economic studies on physical activity, the evidence base is scattered. Systematic review is a high-quality source to inform formulation of policies and setting of budget priorities and build a coherent research base [9~~10~~] but such studies are lacking in economics of physical activity. A few exceptions include a comprehensive review of studies estimating economic burden of physical inactivity worldwide [11]. This current study has a broader scope in terms of the economic evidence (all types of economic analyses) but with limited geographic settings. In line with the standard definition of economics [12], we categorise economic research on physical activity in to; demand analyses of physical activity, impacts of physical (in) activity on economic outcomes such as costs, quality of life, labour market outcomes and economic evaluation studies on physical activity interventions [9,10]. This broad perspective is to facilitate a broader mapping of the multiple facets of the evidence base in LMIC. A specific

For peer review only - http://bmjopen.bmj.com/site/about/guidelines.xhtml

For peer review only

review for LMIC is required considering the differing dynamics of the health systems [13] and the urgent need to better understand the research base and map gaps in knowledge given the growing burden of NCDs [6]. Health research in low- and middle-income countries (LMICs) is warranted to overcome global health challenges [14].

Thus, the aim of this review was to (i) Summarize available evidence on economics of physical activity in LMIC (ii) Describe the focus and methods underpinning research on economics of physical activity in LMIC (iii) Identify potential target variables and cost effective interventions in LMIC for policy formation with regard to physical activity promotion (iv) Identify and report gaps in research on economics of physical activity in LMIC and provide recommendations for economic research agenda in LMIC.

For peer review only - http://bmjopen.bmj.com/site/about/guidelines.xhtml

Methods

Study protocol with detailed methods for this review was published prior to the commencement of this review [15] and shown in supplementary file 1.

Search strategy

Systematic literature search was conducted to identify studies on economics of physical activity in LMIC using the following data bases; Scopus (covers 100% of MEDLINE coverage, 100% of EMBASE coverage, 100% of Compendex coverage [16]), Web of Science and SPORTDiscus. Websites of World Health Organization (WHO), National Institute for Health and Care Excellence (NICE) international, World Bank and reference lists of included studies were searched for any relevant studies. The search period covered beginning of records to December 2017 in line with study protocol. To identify any publications after the search end date (2017), a more recent search was conducted in August 2020 using SCOPUS, the biggest bibliographic database. The search strategy was informed by a scoping review that covered relevant reviews on economics studies on physical activity [17-23], reviews on physical activity [24-26] and reviews on economic evaluations [27-29]. Final search strategy including search terms for each data base is given in supplementary file 2.

For peer review only - http://bmjopen.bmj.com/site/about/guidelines.xhtml

Eligibility criteria and screening

All results were screened by titles to remove irrelevant studies and duplications independently by two reviewers (PDR, NKA). Disagreements were discussed with a third reviewer (SP). Remaining studies were further screened by titles and abstracts prior to full text screening. Studies were included if the following criteria were met; (i) Study setting: Any setting of LMIC in accordance with the definition of LMIC by World Bank classification 2017[13~~14~~] (ii) Population: any age group across life course (iii) Interventions: any physical activity intervention (iv) Comparator: normal routine, no intervention (intervention and comparator applicable only for intervention studies) (v) Outcomes: (a) cost effectiveness ratio(CE ratio), QALY, incremental cost effectiveness ratio (ICER) assessed as the outcome of physical activity intervention, (b) cost of physical (in) activity in terms of health care cost and/or productivity loss and/or total cost of physical inactivity (c) measures of association of any economic variable with PA (vi) Study design: Observational studies (cohort, case control, cross sectional), experimental studies including randomized control trials(RCT),quasi experimental studies including natural experiments and economic evaluation studies (vii) Studies reported in English language

Case reports, case series, letters to the editor, editorials, reviews, qualitative studies, unpublished thesis, conference abstracts and any unobtainable texts were excluded. Studies that did not report cost, economic evaluations, and economic correlates of physical activity, separately from other lifestyle risk factors, were excluded from the review by consensus of all three reviewers. List of excluded studies with reasons given in supplementary file 3.

For peer review only

For peer review only - http://bmjopen.bmj.com/site/about/guidelines.xhtml

Data extraction

Data on following main items; general information, characteristics of the study, characteristics of population/intervention/comparator/outcome/study design(PICOS), data sources, analysis, results, conclusions, and limitations were extracted using a word template. Data extraction form was adapted from data extraction forms from relevant reviews [21, 30,31]. The full data extraction form is shown in supplementary 4.

Risk of bias and quality appraisal

Larg and Moss checklist [32], check list by NICE [33] were used to assess risk of bias of cost of illness studies and studies on correlations or associations respectively. Drummond [34] and Phillips check lists [35] were used for economic evaluations studies. The latter more specifically for model based economic evaluations.

Overall quality grading was given for both internal validity and external validity using NICE recommendations [33]. The internal validity of studies on correlations was assessed based on section 2-4 and external validity section 1 of NICE check list [33]. For cost of illness studies, external validity was assessed by Q2b (III) and internal validity by remaining questions in Larg and Moss checklist [32].

Studies meeting all or most (>75%) were scored '++', some criteria met (50%-75%) had '+' and few (<50%) had '-'. The cut-offs were set with full agreement of all three reviewers prior to commencement of the study.

For peer review only - http://bmjopen.bmj.com/site/about/guidelines.xhtml

Data from included studies were extracted by one reviewer (PDR). A second reviewer (NKA) independently extracted data from 50% (9/18) of the included papers, randomly selected. Disagreements were discussed with a third reviewer (SP). Same pattern was repeated for quality appraisal.

Data synthesis

Descriptive synthesis of data was performed in order to describe the methods, operationalization of methods, quality, major limitations, outcome, suggestions and recommendations for future research. Data from research evidence on economics of physical activity in LMIC was used to prepare summary tables. Identification of potential target variables for policy and the gaps in research on economic of physical activity was gleaned from descriptive analysis of available evidence.

Patient and Public Involvement

The review was based on already available published research. Patients or public were not directly involved in designing and conduct of the study. The results of the review will be disseminated through this scientific publication, conference presentations, and social media networks available in public domain.

For peer review only - http://bmjopen.bmj.com/site/about/guidelines.xhtml

Results

Search outcomes

The systematic literature search (in 2018) retrieved 16 724 records from the initial data base search, 11 records from other sources and 2962 records from updated search (2020) search. After removing duplicates and irrelevant records by screening of titles, 97 articles were selected for abstract screening. Thirty-four records were assessed for eligibility at full text screening. Eighteen articles were selected for data extraction [36-53]. Flow diagram of this process is illustrated in figure 1.

Overview of studies

The 18 studies originated from Brazil (n=9, 50.0%), China (n=3, 16.7%), Malaysia (n=2, 11.1%), South Africa and Iran (n=1, 5.6% from each). There were two global analysis (n=2, 11.1%) (Figure 2). Majority of studies were conducted in upper middle-income countries (n=16,88.8%) [36-37, 39-51, 53]. All studies were published after 2012. Supplementary file 5, provides an overview of the aim of the study. Four studies focused on economic burden of physical inactivity [38,43,47,51] (n=4, 22.2%), six studies assessed association of physical (in)activity and direct or indirect cost [36,39,40,42,44,46] (n=6,33.3%) and seven studies examined the association of physical (in)activity and economic factors [37,41,45,48,49,50,51] (n=7, 38.8%). Only one economic evaluation study was identified (n=1, 5.6%) [53].

For peer review only - http://bmjopen.bmj.com/site/about/guidelines.xhtml

Methods of reviewed studies

The summary of studies by methods is given in table 1. Majority of the studies have used cross sectional design (n=14,77.8%) (36-44,47-49,51,52).

All four studies on economic burden of physical activity, used secondary data to estimate the cost of inactivity while prevalence data on inactivity was obtained from population surveys or GBD study.

DALYs lost due to physical inactivity in LMIC, was estimated by one study as a part of a global analysis [38]. Similar methodology was applied in the other three studies using population attributable fraction (based on prevalence of inactivity, relative risk values (RR), cost of main NCDs related to physical inactivity) [38, 43, 51]. Estimates for RR were obtained from developed countries due to unavailability of data for LMIC. One study has assessed only the physical inactivity related inpatient costs of chronic non communicable diseases [47].

Majority of studies examined the association of physical (in)activity and economic factors. The economic variables identified were direct health cost [39,40,42,44,46], productivity lost [36], and socioeconomic indicators [37, 41,45,48,50,52]. The method of analyses was regression based particularly for the direct health care cost and productivity loss analyses [36,40,42,44,46]., and bivariate for the rest [37,41,45,48,49,50,52]. A summary of included studies by PICOS, is shown in table 2.

Table 3 shows that the method of operationalization of physical activity varied across the studies. Only two studies used the WHO recommended definition of physical activeness; 150min of physical activity per week [38,39]. One study used more strict definition of activeness; 300min during

For peer review only - http://bmjopen.bmj.com/site/about/guidelines.xhtml

last 7 days [45]. All physical activity data were based on self-reports with the Baecke questionnaire identified as the most frequently used survey instrument [36,40,42,44,53].

Cost was measured either as direct health care cost or indirect cost. Direct health care cost was specified in practice as cost for medications, laboratory tests and medical consultations [39,40,42,44] and/or inpatient cost [39,46] and/or outpatient cost, prescription cost [38](Table 3). Indirect cost was operationalized as loss of productivity due to premature mortality [38], absenteeism and disability retirement [36].

Quality appraisal of included studies

Table 4 summarises quality appraisal of included studies and supplementary file 6 details the results of quality appraisal by Larg and Moss check list [38,43,47,51], NICE check list for studies on correlations and association [36,37,39-42,44-46,48-50,52], and Drummond check list for the economic evaluation study[53].

Majority of studies fulfilled most of the criteria (n=11,61.1%) [36, 37, 38, 40, 41, 42, 44, 45, 47, 48,52], while five studies fulfilled some of the criteria (6/18, 33.3%) [49, 50, 43,47,53] for internal validity. Insufficient information on methods precluded quality appraisal for one study [51]. Similarly, external validity was good in all studies.

For peer review only - http://bmjopen.bmj.com/site/about/guidelines.xhtml

Empirical results from reviewed studies

The empirical evidence from the reviewed papers are summarised below in the account of the main aims of the papers (Table 5).

Economic burden of physical inactivity in LMIC

Direct cost

The direct health care cost in LMIC attributable to physical inactivity was found to be 10.3 billion INT$ in 2013 which is 19.1% of direct health care cost attributable to physical inactivity worldwide. Upper middle-income countries contributed to 86% of this economic burden [38]. Estimated direct health cost in China attributed to physical inactivity in 2007 was 3.5 billion USD [43] while cost of hospital admissions in Brazil in 2013 was estimated as 0.73 billion USD by local studies [47]. Estimated direct health care cost attributed to physical inactivity was 3.1 billion INT$ for China and 1.6 billion INT$ for Brazil in 2013 by global analysis [38]. Inpatient cost and outpatient cost attributed to physical inactivity in 2000 was 0.4 billion USD and 0.9 billion USD in China [51]. Cost of chronic diseases attributed to physical inactivity in China accounted for 15.2% of total cost of main NCDs in 2007 while 15% of inpatient costs from Brazilian Health Care System in 2013 was related to insufficient PA.

For peer review only - http://bmjopen.bmj.com/site/about/guidelines.xhtml

Indirect cost and DALYs

Physical inactivity related deaths in LMIC settings contributed to productivity loss of 5.3 billion INT$ covering 38.7% of productivity loss due to deaths related to physical inactivity worldwide [38]. LMIC bear 10.1 million DALYs attributed to physical inactivity which is 75% DALYs worldwide [38]. Indirect cost of physical inactivity in China was estimated as 3.3 billion USD in 2007 [43]. Estimated indirect cost due to productivity loss by deaths related to physical inactivity in China was reported as 1.78 billion INT$.

Association of physical (in) activity and health care cost in LMIC

Walking (always) during leisure time was showed to be associated with lower health care expenditure in primary care (OR_{adj} 0.59, 0.39-0.89) [40]. Higher health care expenditure for medicine was associated with lower participation in physical activity at work (OR 1.58 (1.06-1.35) and sport (OR1.57 (1.12-2.18) in Brazil [42]. Further, expenditure related to medicine (r= 0.109, 95%CI 0.046- 0.171) and overall expenditure (r= 0.092, 95%CI 0.046-0.171) was significantly associated with physical inactivity in Brazil [42].In addition, a significant association of expenditure on medication was with moderate physical activity showed by another Brazilin study (OR 0.56, 0.38-0.81) [44]. A retrospective longitudinal study in South Africa showed that the odds of hospital admissions were 13% lower for two additional gym visits per week among participants of incentive-based fitness related activity programme [46]. Members who were not on a health promotion programme had a significantly higher probability of hospital admissions (p<0.01) and higher claims than participants who changed their physical activity status to “active to no change”

For peer review only - http://bmjopen.bmj.com/site/about/guidelines.xhtml

group or “active to more active group” in South Africa [46]. However, a Brazilian study showed inconclusive results for association of adherence to an incentive based physical activity program and direct health care cost among employees of a private health care company [39].

Physical inactivity was found to be correlated with productivity losses. A Brazilian study showed that physical activity was associated with productivity loss due to absenteeism OR_{adj} 0.29(0.09-0.88) [36].

Economic factors associated with physical (in) activity in LMIC

A number of economic factors were showed to be potential target variables for physical activity promotion policies.

Income

Two Malaysian studies revealed that individuals with higher levels of income, are less likely to participate in physical activity [37,41]. This was corroborated by a Brazilian study using data from Brazil`s National household sample survey 2008 to analyse income quintiles by active commuting. The study revealed that in urban population lowest income quintile had higher active transport (walking and cycling) level than the wealthiest population (2-5 times higher) [49] (p<0.001). Similarly, in China income levels were associated with lower physical activity [48].

For peer review only - http://bmjopen.bmj.com/site/about/guidelines.xhtml

Socioeconomic status

Cross sectional study carried out in China reported that people from upper socioeconomic status use active transport more compared to lower social classes (p <0.0001) [48]. However, this study further, revealed that people in upper socioeconomic classes had less overall intensity of physical activity in MET hours per week (114.4, 95%CI 106.6-122.5) compared to lower class (140.3, 95%CI 132.3-148.5), lower middle class (167.4, 95%CI 163.8-171), upper middle class (141.1. 95%CI 135.6-146.6) [48].

Employment

Cross sectional study conducted in a national representative sample in Malaysia revealed that employed individuals were more likely to participate in physical activity compared to unemployed individuals (LR 0.035, p<0.05) [41]. A global analysis of occupational structure and physical inactivity reported that individuals working in white-collar industry compared to agriculture were more likely to be physically inactive (OR=1.84, CI 1.73-1.95) [52].

Health Insurance

In Malaysia, individuals who have insurance coverage were found to be 3.2% more likely to participate in physical activity, but do not spend more time than their counterparts who are not covered by insurance [41].

For peer review only - http://bmjopen.bmj.com/site/about/guidelines.xhtml

Attractiveness/cost for leisure time physical activity

Attractiveness of recreational leisure time physical activity (LTPA) was more likely to predict the participation in LTPA among students of Teheran Universities (OR 1.073), but time and money costs were not significant predictors [50].

Macroeconomic indicators

In Brazil, Gini index accounted for 12% variance in Physical activity (adjusted R^2=0.12), if Gini index of the city increase by 1%, prevalence of sufficient physical activity would decrease by 41.5% for girls and 60.2% for boys [45]. Global analysis among forty-seven LMIC, reported that increase of Human Development Index (HDI) value is associated with decreased levels of insufficient physical activity (OR=0.98, 95%CI 0.97-0.99) [52].

Cost effectiveness of physical activity interventions

Recent study conducted in Brazil revealed the value for money of combining physical activity and drug medication for the treatment of chronic diseases as the treatment scheme for >50year old attendees of selected primary health care settings [53]. Higher cost utility ratio ($ 3.92 / QALY) was reported among individuals on “medications only” compared to those on “medication and regular physical activity” ($ 3.21 / QALY) and “no medication or physical activity” groups ($ 0.12 / QALY).

For peer review only - http://bmjopen.bmj.com/site/about/guidelines.xhtml

Research gaps

Box 1 summarises the gaps in the evidence base. Majority of the studies either assessed the socioeconomic factors associated with physical activity or the economic burden of physical inactivity. Limited evidence on economic evaluation in LMIC.

BOX1

Gaps in evidence on economics of physical activity in LMIC

- Economic evaluations of interventions and practices for physical activity promotion is minimal
- A standard method to quantify economic burden of physical inactivity at country level is lacking
- No standard method of operationalization of physical inactivity to enable transferability of evidence
- Limited evidence available on economics of PA in LMIC compared to HIC
- No evidence from local led research from low income countries.

For peer review only - http://bmjopen.bmj.com/site/about/guidelines.xhtml

Discussion

Economic burden of insufficient physical activity is consistently highlighted as problem of HIC while larger proportion of disease burden is a problem of LMIC [38]. Thus, the economic burden of physical inactivity in LMIC could be predicted to be escalated in near future. However, considering LMIC as separate entity, even the current estimates of economic burden is huge in comparison to the income level of these countries.

Economic burden of insufficient PA [43,47] and the inverse association of PA with economic cost of chronic diseases [36,40,42,44,46], revealed by locally led research in this review. This is an important evidence to draw attention of authorities and policy makers on PA promotion as a priority measure in LMIC. Global analysis on economic burden of insufficient PA including LMIC, further enhanced this evidence [38]. However, locally led research were mostly contributed by Brazilian studies. The situation could be the same in other LMIC, though the evidence is limited from other countries. It is well evident that investing on physical activity promotion will pave the path towards achieving the sustainable development goals 2030 in LMIC [1].

The cost estimates of studies from China and Brazil cannot be directly compared with the results from global analysis due to methodological differences and difference in year of cost estimate of these studies. There is no evidence on economic burden of physical inactivity from LIC by local studies. Thus, capacity building of researchers of LMIC should be done to apply more consistent, robust methodology of cost estimation and to obtain better epidemiological and economic data for cost estimation, particularly in LMIC [38].

For peer review only - http://bmjopen.bmj.com/site/about/guidelines.xhtml

Majority of research from economic evidence base in LMIC were focused on socioeconomic factors associated with participation in PA. These are also important to identify prioritize population groups for physical activity promotion. Upper socioeconomic class, people in higher income category, unemployed individuals and white-collar job employees are at higher risk of less physical activity participation, low intensity and shorter duration of PA. Hence, these groups need to be prioritized in physical activity promotion in LMIC.

This review has shown that macroeconomic indicators of the country are significantly associated with physical activity of the population. Physical activity level increases with the increase of HDI [52] and decreases with Gini index, which is a measure of inequality of income and wealth distribution of populations [45]. Though income level alone has shown a negative association with level of PA [37, 41, 48, 49] in LMIC, socioeconomic status of population subgroups as a whole have shown a positive association with the quality and quantity of physical activity in high income countries [54]. This would have contributed to the positive association of level of physical activity and HDI of the countries [45, 52]. Based on one study we are unable to generalize the usefulness of health insurance schemes and attractiveness of PA options in physical activity promotion. However, more studies need to be conducted to conclude on effect of health insurance on PA and effect of attractiveness of PA intervention compared to time and cost variables.

It is of paramount importance to understand cost effective interventions and strategies for physical activity promotion in LMIC. There was only one recent research on cost utility analysis. This initiation to conduct economic evaluation studies in LMIC is an achievement. Prescribing "PA along with medication" or "PA alone" as a treatment regime for chronic diseases, which was proved to be advantageous through cost utility

For peer review only - http://bmjopen.bmj.com/site/about/guidelines.xhtml

analysis, should be considered to implement in LMIC. This is with great value for treatment policy for NCD in LMIC. Positive association of regular walking with less medical expenditure, further supported above evidence [40]. Health impact of exercise prescription was also well documented by studies from HIC [55].

Economic evidence base on PA is biased towards MIC, especially towards Brazil, as most of these researches were conducted in Brazil. Another global review on economic burden of physical inactivity related to cardiovascular diseases also noted scarcity of research from LMIC other than few Brazilian studies [56]. Locally led research from low income countries not available. Same study population of >50-year-old adults seeking treatment from primary health care settings were studied in majority of Brazilian studies. Adolescents as a study population need to be prioritized in the future research agenda on economic aspects of PA. This review showed that economics of physical activity is a grossly neglected area in the research agenda of LMIC. Furthermore, locally led research in LMIC are much needed to overcome global health challenges [57]. Thus, more local research needs to be encouraged in this field in both middle income and low-income countries as a global priority to combat the global pandemic of physical inactivity. It is of paramount importance that researchers should explore and identify cost effective interventions to improve physical activity within the context of LMIC. Health research capacity of LMIC is insufficient and developing their capacity is not prioritized globally [56]. Overview of economic evaluations in LMIC reports the challenge of conducting economic evaluations in this context due to absence of routine cost accounting system and limited patient information systems [58]. Further, they reveal the limited capacity and funding to conduct health economic research in most LMIC [58]. An important suggestion that the findings of present review agree with the overview

For peer review only - http://bmjopen.bmj.com/site/about/guidelines.xhtml

of economic evaluations in LMIC [58] is to encourage economic evaluation studies in LMIC by advocating funders to request economic evaluations to be included for trails and other evaluations to ensure funding for those researches.

Despite the limited number of studies in LMIC on economics of PA, they reported a good methodological quality. Though, sensitivity analysis not performed in the study on cost utility analysis, it also reported a considerable quality, based on Drummond check list criteria.

The policy makers and authorities need to be advocated on the consistent evidence from LMIC on increased health care cost and productivity loss due to physical inactivity [34, 37,38, 40, 42,44], possibility of escalating the economic burden related to physical inactivity in near future [36] and the need include physical activity promotion across all policies. Further, prioritization and investment for research on economics of PA in LMIC need to be a global priority.

Limitations: Meta-analysis of data was not possible in this review due to wide variation in focus and methods of the included studies for the review. All locally led research were from MIC. Though we interpret the results for LMIC, there is a considerable socio-economic disparity between LIC and MIC.

Conclusions and Recommendations: Considerable cost of chronic diseases and hospital admissions are connected to physical inactivity in LMIC. High income, high socio-economic level, unemployment status and white-collar jobs associated with low PA levels. PA should be prescribed alone or along with medication to reduce the cost per QALY among patients with chronic disease. Research on economics of PA is grossly

For peer review only - http://bmjopen.bmj.com/site/about/guidelines.xhtml

For peer review only

inadequate in LMIC. It should be a priority research area in LMIC. Especially the research on economic evaluations related to physical activity should be considered as a priority in research agenda in LMIC.

Contributors: SP and NKA developed the idea for the review with inputs from PDR. PDR and NKA conducted the review with the guidance of SP. PDR wrote the first draft. SP and NKA revised the manuscript. NKA will act as guarantor of the review.

Conflicts of interest: None

Funding: This research received no specific grant from any funding agency in the public, commercial or not-for-profit sectors

Data sharing statement: No additional data available

Data sharing statement: All data relevant to the study are included in the article and uploaded as supplementary files. If any further information is required, will be provided on request to authors by email.

Completed reporting checklist for systematic review and meta-analysis based on the PRISMA guidelines given in supplementary file 7.

Figure 1: Flow diagram of article selection process

Figure 2: Number of articles on economics of physical activity by country

For peer review only - http://bmjopen.bmj.com/site/about/guidelines.xhtml

List of references

1. World Health Organization. Action plan on physical activity 2018–2030: more active people for a healthier world. Geneva: World Health Organization; 2018. Licence: CC BY-NC-SA 3.0 IGO.
2. Centres for Disease Control and Prevention. 2020, Benefits of Physical Activity, Accessed on [23.07.2020] https://www.cdc.gov/ physicalactivity/basics/pa-health/index.htm
3. World Health Organization. Global Status Report on Non communicable Diseases 2014. Geneva: World Health Organization; 2014. http://apps.who.int/iris/bitstream/10665/148114/1/9789241564854_eng.pdf?ua=1
4. Lee, I. M., Shiroma, E. J., Lobelo, F., Puska, P., Blair, S. N., Katzmarzyk, P. T., & Lancet Physical Activity Series Working Group (2012). Effect of physical inactivity on major non-communicable diseases worldwide: an analysis of burden of disease and life expectancy. *Lancet (London, England)*, *380*(9838), 219–229. https://doi.org/10.1016/S0140-6736(12)61031-9
5. World Health Organization. 2016, Global Health Observatory (GHO) data, NCD mortality and morbidity, Accessed on 30.07.2020 https://www.who.int/gho/ncd/mortality_morbidity/ncd_four/en/
6. World Health Organization.2018, *Detail-Physical activity*, viewed on 21.05.2018, http://www.who.int/en/news-room/fact-sheets/detail/physical-activity
7. Guthold, R., Stevens, G.A., Riley, L.M., Bull, F.C., (2018). Worldwide trends in insufficient physical activity from 2001 to 2016: a pooled analysis of 358 population-based surveys with 1·9 million. *Lancet Glob Health,* 6: e1077–86 http://dx.doi.org/10.1016/ S2214-109X(18)30357-7

For peer review only - http://bmjopen.bmj.com/site/about/guidelines.xhtml

8. Guthold, R., Stevens, G.A., Riley, L.M., Bull, F.C., (2020). Global trends in insufficient physical activity among adolescents: a pooled analysis of 298 population-based surveys with 1·6 million participants, *Lancet Child Adolesc Health*, 4: 23–35 https://doi.org/10.1016/ S2352-4642(19)30323-2

9. National Coalition for Promoting Physical Activity. 2005. Economics of physical activity promotion United States. Accessed on 25.07.2020 http://www.ncppa.org/sites/default/files/Economics%20of%20Physical%20Activity%20Final.pdf

10. Sturm R. (2005). Economics and physical activity: a research agenda. *Am J Prev Med*, 28:141–9. 13.

11. Ding D, Kolbe-Alexander T, Nguyen B, et al. (2017). The economic burden of physical inactivity: a systematic review and critical appraisal. *British Journal of Sports Medicine*; 51:1392-1409.

12. Backhouse, R.E., (2019). Founder of Modern Economics: Paul A. Samuelson: Volume 1: Becoming Samuelson, 1915-1948 (Oxford Studies in History of Economics), 1st Edition, Oxford University Press 2019, ISBN: 0190664118

13. Bank W. World bank country and leading groups: country classification. 2017, https://datahelpdesk.worldbank.org/ knowledgebase/articles/906519-world-bank-country-and-lendinggroups (Accessed on 1st Dec 2017)

14. Dye, C., Boerma, T., Evans, D., et al. The World Health Report 2013: research for universal health coverage. Geneva: World Health Organisation, 2013.

15. Ranasinghe, P.D., Pokhrel, S., Anokye, N.K. (2019) The economics of physical activity in low-income and middle-income countries: protocol for a systematic review. *BMJ Open*, 9:e022686. doi: 10.1136/bmjopen-2018-022686

16. Burnham, J. F. (2006). Scopus database: a review. *Biomed Digit Libr,* 3(1), 1742-5581.

For peer review only - http://bmjopen.bmj.com/site/about/guidelines.xhtml

17. . Oldridge, N. B. (2008). Economic burden of physical inactivity: healthcare costs associated with cardiovascular disease. *European Journal of Cardiovascular Prevention & Rehabilitation*, 15(2), 130-139. doi: 10.1097/HJR.0b013e3282f19d42 15.

18. Jeroen C. M. Barte & G. C. Wanda Wendel-Vos (2017) A Systematic Review of Financial Incentives for Physical Activity: The Effects on Physical Activity and Related Outcomes, *Behavioral Medicine*, 43:2, 79-90, DOI: 10.1080/08964289.2015.1074880 16.

19. Abu-Omar, K., Rütten, A., Burlacu, I., Schätzlein, V., Messing, S., & Suhrcke, M. (2017). The cost-effectiveness of physical activity interventions: A systematic review of reviews. *Preventive Medicine Reports*, 8, 72–78. http://doi.org/10.1016/j.pmedr.2017.08.006 17.

20. Brown, V., Zapata Diomedi, B., Moodie, M., Veerman, J., & Carter, R. (2016). A systematic review of economic analyses of active transport interventions that include physical activity benefits. Transport Policy, 45(18)190-208.

21. Pavey, T. G., Taylor, A. H., Fox, K. R., Hillsdon, M., Anokye, N., Campbell, J. L., Taylor, R. S. (2011). Effect of exercise referral schemes in primary care on physical activity and improving health outcomes: systematic review and meta-analysis. *BMJ,* 343. doi: 10.1136/bmj.d6462 19

22. Campbell, F., Holmes, M., Everson-Hock, E., Davis, S., Woods, H. B., Anokye, N., Kaltenthaler, E. (2015). A systematic review and economic evaluation of exercise referral schemes in primary care: a short report. *Health Technology Assessment,* 19(60), 1-+. doi: 10.3310/hta19600 Page 24 of 95

23. Suhrcke, M., Boluarte, T. A., & Niessen, L. (2012). A systematic review of economic evaluations of interventions to tackle cardiovascular disease in low- and middle-income countries. [journal article]. *BMC Public Health,* 12(1), 2. doi: 10.1186/1471-2458-12-2

24. Milton, K., Macniven, R., & Bauman, A. (2014). Review of the epidemiological evidence for physical activity and health from low- and middle-income countries. [Review]. *Glob Public Health*, 9(4), 369-381.

For peer review only - http://bmjopen.bmj.com/site/about/guidelines.xhtml

25. Lachat, C., Otchere, S., Roberfroid, D., Abdulai, A., Seret, F. M., Milesevic, J., Kolsteren, P. (2013). Diet and physical activity for the prevention of noncommunicable diseases in low and middle-income countries: a systematic policy review. [Meta-Analysis Review]. *PLoS Med,* 10(6), 11. 23.

26. Hunter, R. F., Christian, H., Veitch, J., Astell-Burt, T., Hipp, J. A., & Schipperijn, J. (2015). The impact of interventions to promote physical activity in urban green space: a systematic review and recommendations for future research. [Research Support, Non-U S Gov't Review]. *Soc Sci Med,* 124, 246-256.

27. Sutton, L., Karan, A., & Mahal, A. (2014). Evidence for cost-effectiveness of lifestyle primary preventions for cardiovascular disease in the Asia-Pacific Region: a systematic review. *Globalization and Health*, 10, 79. http://doi.org/10.1186/s12992-014-0079-3

28. Santatiwongchai, B., Chantarastapornchit, V., Wilkinson, T., Thiboonboon, K., Rattanavipapong, W., Walker, D. G., Teerawattananon, Y. (2015). Methodological Variation in Economic Evaluations Conducted in Low- and Middle-Income Countries: Information for Reference Case Development. *PLoS ONE*, 10(5), e0123853. doi: 10.1371/journal.pone.0123853

29. Nosratnejad, S., Rashidian, A., & Dror, D. M. (2016). Systematic Review of Willingness to Pay for Health Insurance in Low- and Middle-Income Countries. [Review]. *PLoS One,* 11(6).

30. Pokhrel, S., Quigley, M. A., Fox-Rushby, J., McCormick, F., Williams, A., Trueman, P., Renfrew, M. J. (2015). Potential economic impacts from improving breastfeeding rates in the UK. [Research Support, Non-U.S. Gov't]. *Arch Dis Child,* 100(4), 334-340. doi: 10.1136/archdischild-2014-306701

31. Murthy, S., John, D., Godinho, I. P., Godinho, M. A., Guddattu, V., & Nair, N. S. (2017). A protocol for a systematic review of economic evaluation studies conducted on neonatal systemic infections in South Asia. *Syst Rev,* 6(1), 252. doi: 10.1186/s13643-017-0648-7

For peer review only - http://bmjopen.bmj.com/site/about/guidelines.xhtml

32. Larg, A., & Moss, J. R. (2011). Cost-of-illness studies: a guide to critical evaluation. [Research Support, Non-U S Gov't]. *Pharmacoeconomics, 29*(8), 653-671.

33. National Institute for Health and Excellence (NICE), 2012. Methods for the development of NICE public health guidance (third edition), Appendix G Quality appraisal checklist-quantitative studies reporting correlations and associations. https://www.nice.org.uk/process/pmg4/chapter/appendix-g-quality-appraisal-checklist-quantitative-studies-reporting-correlations-and

34. Drummond, M. F., & Jefferson, T. O. (1996). Guidelines for authors and peer reviewers of economic submissions to the BMJ. *BMJ, 313*(7052), 275-283. doi: 10.1136/bmj.313.7052.275

35. Philips, Z., Ginnelly, L., Sculpher, M., Claxton, K., Golder, S., Riemsma, R., Glanville, J. (2004). Review of guidelines for good practice in decision-analytic modelling in health technology assessment. [Review]. *Health Technol Assess, 8*(36), 1-158.

36. Araujo, M. Y. C., et al. (2017). Association between Costs Related to Productivity Loss and Modified Risk Factors among Users of the Brazilian National Health System. *Journal of Occupational and Environmental Medicine*, **59**(3): 313-319.

37. Cheah, Y. K., et al. (2017). The Income and Demographic Determinants of Physical Activity Intensity: Evidence from a Developing Country. *Margin-Journal of Applied Economic Research*, **11**(3): 348-364.

38. Ding, D., et al. (2016). The economic burden of physical inactivity: a global analysis of major non-communicable diseases. *The Lancet*, **388**(10051): 1311-1324.

39. Mitsunaga, P.K., Bandini, M.,Lucca, S.R.,Moreira-Filho, D.C. (2018). Assessment of physical activity program on the medical-hospital costs of a health care company. *Rev Bras Med Trab*.2018;16(2):121-7

For peer review only - http://bmjopen.bmj.com/site/about/guidelines.xhtml

40. Turi, B. C., et al. (2015). Walking and health care expenditures among adult users of the Brazilian public healthcare system: Retrospective cross-sectional study. *Ciencia e Saude Coletiva*, **20**(11): 3561-3568.

41. Cheah, Y. K., et al. (2017). Factors affecting participation decision and amount of physical activity among urban dwellers in Malaysia. *Public Health*, **46**: 84-91.

42. Codogno, J. S., et al. (2015). "Physical inactivity of adults and 1-year health care expenditures in Brazil." International Journal of Public Health **60**(3): 309-316.

43. Zhang, J. and J. Chaaban (2013). The economic cost of physical inactivity in China. *Preventive Medicine*, **56**(1): 75-78.

44. Turi, B. C., et al. (2017). Determinants of outpatient expenditure within primary care in the Brazilian national health system. *Sao Paulo Medical Journal,* **135**(3): 205-212.

45. Silva, D. A. S. (2015). Relationship between Brazilian adolescents physical activity and social and economic indicators of the cities where they live. *Perceptual and Motor Skills,* **120**(2): 355-366.

46. Patel, D., et al. (2011). Participation in Fitness-Related Activities of an Incentive-Based Health Promotion Program and Hospital Costs: A Retrospective Longitudinal Study. *American Journal of Health Promotion*, **25**(5): 341-348.

47. Bielemann, R. M., et al. (2015). Burden of physical inactivity and hospitalization costs due to chronic diseases. *Rev Saude Publica,* **49**(89102015000100307): 20.

48. Chen, M., et al. (2015). Socioeconomic Status and Physical Activity in Chinese Adults: A Report from a Community-Based Survey in Jiaxing, China. *PLOS ONE,* **10**(7): e0132918.

For peer review only - http://bmjopen.bmj.com/site/about/guidelines.xhtml

49. Thiago. S., H., et al. (2016). Socioeconomic and regional differences in active transportation in Brazil. *Revista de Saúde Pública*,**50**: 37.

50. Abdi, K. et al. (2014). Analysis Role of Cost, Time and Attractiveness at leisure time physical activities in students of Tehran universities, Iran. *International Journal of Research in Management*(ISI),1(4): 16-29

51. Popkin, B. M., et al. (2006). Measuring the full economic costs of diet, physical activity and obesity-related chronic diseases. *Obes Rev*, **7**(3): 271-293.

52. Atkinson, K., Lowe, S., & Moore, S. (2016). Human development, occupational structure and physical inactivity among 47 low and middle income countries. *Preventive Medicine Reports, 3*, 40-45.

53. Queiroz,C.D., Turi, B.C., Sarti, F.M., Santos Ferro, I., Morais , L.C., Codogno, J.S.(2020). Association between quality of life, physical activity, use of medication and costs of treatment for chronic diseases in Primary Care, *The Journal of Sports Medicine and Physical Fitness*, 60(3):456-63 DOI: 10.23736/S0022-4707.19.10170-3

54. Earl S. Ford, Robert K. Merritt, Gregory W. Heath, Kenneth E. Powell, Richard A. Washburn, Andrea Kriska, Gwendolyn Haile, Physical Activity Behaviors in Lower and Higher Socioeconomic Status Populations, *American Journal of Epidemiology*, Volume 133, Issue 12, 15 June 1991, Pages 12461256, https://doi.org/10.1093/oxfordjournals.aje.a115836

55. Robert Sallis (2015) Exercise is medicine: a call to action for physicians to assess and prescribe exercise, *The Physician and Sports medicine,* 43:1, 22-26, DOI: 10.1080/00913847.2015.1001938

56. Oldridge, N.B., (2008). Economic burden of physical inactivity: healthcare costs associated with cardiovascular disease. *Eur J Cardiovasc Prev Rehabil.* 15(2):130-9. doi: 10.1097/HJR.0b013e3282f19d42.

For peer review only - http://bmjopen.bmj.com/site/about/guidelines.xhtml

57. Franzen, S.R.P., Chandler, C, Lang, T., Health research capacity development in low and middle income countries: reality or rhetoric? A systematic meta-narrative review of the qualitative literature. *BMJ Open*, 2017;7:e012332. doi:10.1136/bmjopen-2016- 012332

58. Pitt, C., Vassall, A., Teerawattananon, Y., Griffiths, U. K., Guinness, L., Walker, D., Hanson, K. (2016). Foreword: Health Economic Evaluations in Low- and Middle-income Countries: Methodological Issues and Challenges for Priority Setting. *Health economics*, 25 Suppl 1(Suppl Suppl 1), 1–5. doi:10.1002/hec.3319

For peer review only

Table 1: Summary of studies by focus and study design

	Number (n)	studies
**Focus**		
Economic burden of physical inactivity	4	Ding 2016[38] Zhang & Chaban 2012[43] Bielemann 2015[47] Popkin 2006[51]
Association of physical (in)activity and direct health cost	5	Mitsunga 2018[39] Turi 2015 [40] Codongo 2015[42] Turi 2017[44] Patel 2011[46]
Association of physical (in)activity and productivity lost	1	Araujo 2017[36]
Socioeconomic factors associated with physical activity participation and intensity	7	Cheah 2017[37] Cheah 2017[41] Silva A.S 2015[45], Chen 2015[48] Thiago 2016[49] Abdi 2014[50]Atkinson 2016[52]
Economic evaluations related to physical activity	1	Queiroz et al 2020[53]
Study design		
Cross sectional	14	Araujo 2017[36] Cheah 2017[37] Ding 2016[38] Mitsunga 2018[39]Turi 2015[40] Cheah 2017[41] Codongo 2015[42] Zhang & Chaban 2012[43] Turi 2017[44] Bielemann 2015[47] Chen 2015[48] Thiago 2016[49] Popkin 2006[51] Atkinson 2016[52]
Ecological/Correlation study	2	Silva A.S 2015[45], Abdi 2014[50]
longitudinal	1	Patel 2011[46]
Intervention studies	0	
Economic evaluation studies	1	Queiroz et al 2020[53]

For peer review only - http://bmjopen.bmj.com/site/about/guidelines.xhtml

Table 2: Summary of studies by PICOS

Author year	*Country/ Country category*	*Type of economic analysis*	*Population*	*Intervention*	*comparator*	*outcome*	*study design*
Araujo 2017[36]	Brazil- UMC	Economic correlates of PA*	≥50y adults	NA	NA	Strength of association between physical inactivity and productivity loss(OR,95%CI)	Cross sectional
Cheah 2017[37]	Malaysia-UMC	Economic correlates of PA	General population	NA	NA	Strength of association of income and physical activity (Coefficient of income)	Cross sectional
Ding 2016[38]	Global analysis-UMC/LMC/LIC	Economic burden	General population	NA	NA	Cost estimates, DALY	Cross sectional
Mitsunaga 2018[39]	Brazil-UMC	Association of PA program with medical hospital cost	Employees of a private health care company	NA	NA	Correlation of program attendance frequency with cost per capita Association of program adherence level with the average cost per capita/year	Cross sectional (study design not specified in the original article. Based on the study pattern and type of analysis we categorized as cross sectional)
Turi 2015[40]	Brazil-UMC	Association of PA and economic costs	≥50y adults	NA	NA	Association of walking and health care expenditure (OR,95% CI)	Cross sectional
Cheah 2017[41]	Malaysia-UMC	Economic correlates of PA	Urban dwellers	NA	NA	Strength of association between participation decision, amount of physical activity with income, time, employment, insurance (coefficient)	Cross sectional

For peer review only - http://bmjopen.bmj.com/site/about/guidelines.xhtml

Codongo 2015[42]	Brazil - UMC	\Association of PA with economic costs	≥50y adults	NA	NA	Strength of association between physical inactivity and health care expenditure (OR, 95%CI)	Cross sectional
Zhang & Chaban 2012[43]	China - UMC	Economic burden	General population	NA	NA	Cost estimate of physical inactivity	Cross sectional
Turi 2017[44]	Brazil- UMC	Association of PA and economic cost	≥50 years adults	NA	NA	Strength of association of physical activity and outpatient expenditure (OR, 95%CI)	Cross sectional
Silva A.S 2015[45]	Brazil- UMC	Economic correlates of PA	Year 9 students Public and private elementary school's	NA	NA	Strength of association of macroeconomic variables with physical activity(Pearson r,95% CI)	Correlation study
Patel 2011[46]	South Africa-UMC	Association of PA with economic cost	Adults who are members of discovery health medical plan	Incentive based health promotion program	Not enrolled in the program	Changes in level of participation in fitness related activities, Subsequent probability of hospital admissions and cost	Retrospective observational study. Not a intervention study
Bielemann 2015[47]	Brazil- UMC	Economic burden	Above 40 year old adults Brazil	NA	NA	Inpatient cost estimates attributed to physical activity	Cross sectional
Chen 2015[48]	China - UMC	Economic correlates of PA	30-65 year permanent residents Jiaxing China	NA	NA	Strength of association of socioeconomic level and PA	Cross sectional
Thiago 2016[49]	Brazil- UMC	Economic correlates of PA	≥14 year general population Brazil	NA	NA	Strength of association of socioeconomic status and active transport	Cross sectional
Abdi 2014 [50]	Iran- UMC	Economic correlates of PA	University students Teheran Universities	NA	NA	Strength of association of time, cost and attractiveness with LTPA	Correlational survey
Popkin 2006[51]	China- UMC	Economic burden	General population	NA	NA	Cost estimate of physical inactivity	Cross sectional(case study

For peer review only - http://bmjopen.bmj.com/site/about/guidelines.xhtml

							developed from a review)
Atkinson 2016[52]	Global analysis- UMC/LMC/LIC	Economic correlates of PA	General population	NA	NA	Strength of association (OR) of physical activity and macroeconomic variables in 47 LMIC	Cross sectional
Queiroz et al 2020[53]	Brazil- UMC	Cost utility analysis	General population	NA	NA	cost-utility and incremental cost-utility of the treatments involved in physical activity compared to drug only treatment group. QALY values and the mean cost of 12 months for each treatment scheme.	Economic evaluation study

*PA- physical activity

For peer review only

Table 3: Methods of operationalization of physical activity and cost in economic studies of physical activity

	Number	studies
**Method of operationalization of physical activity**		
By WHO definition	1	Ding 2016[38], Mitsunaga 2018[39]
Physical activity for >300 min during last 7 days	1	Silva A.S 2015[45]
Based on IPAQ	1	Atkinson 2016[52]
By Baecke questionnaire,	3	Araujo 2017[36],Turi 2015[40] Codongo,2015[42]Turi2017[44] Queiroz et al 2020[53]
Self-reported vigorous or moderate physical activity during last 7 days	2	Cheah2017[37], Cheah 2017[41]
Based on number of Gym visits per annum	1	Patel 2011[46]
Moderate or vigorous physical activity during leisure	1	Abdi 2014[50]
Performing any physical activity during leisure	1	Bielemann 2015[47]
Walking or cycling to work regardless of duration	1	Thiago 2016[49]
Not reported	1	Zhang & Chaban 2012[43]
**Operationalization of cost in economic studies of physical activity**		
**Method of operationalization of direct cost**		
cost of disease using population attributable fraction related to physical inactivity	3	Ding 2016[38] Zhang & Chaban 2012[43]
Inpatient cost	1	Bielemann 2015[47]
Total cost of consultations, diagnostic tests, hospital admissions, therapeutic procedures, emergency care paid by insurance	1	Mitsunaga 2018[39]
Total cost of Medication dispensed/laboratory tests/ medical consultations	3	Turi 2015[40], Codongo 2015[42] Turi 2017[44]
**Method of operationalization of indirect cost**		
Loss of productivity due to premature mortality	1	Ding 2016[38]
Loss of productivity due to absenteeism and disability retirement	1	Araujo 2017[36]

For peer review only - http://bmjopen.bmj.com/site/about/guidelines.xhtml

Table 4: Quality appraisal of included studies

		Number of studies	Studies
By NICE check list for quantitative studies on correlations/associations (n=13)			
Internal validity (section 2-4)	++*	10	Araujo 2017[36] Cheah2017[37] Turi 2015[40] Cheah 2017[41] Codongo,2015[42] Turi2017[44] Silva A.S 2015[45] Patel 2011[47] Chen 2015[48] Atkinson 2016[52]
	+**	3	Mitsunaga[39],Thiago 2016[49], Abdi 2014[50]
	-***	0	
External validity (section 1)	++	9	Turi 2015[40] Min J.Y. & Min K.B. 2016[39] Cheah 2017[41] Codongo,2015[42]Turi2017[44] Silva A.S 2015[45] Patel 2011[46] Chen 2015[48] Atkinson 2016[52]
	+	3	Araujo 2017[36] Cheah2017[37] Abdi 2014[50]
	-	0	
Check list by Larg & Moss for cost of illness studies (n=4****)			
Internal validity	++	1	Ding 2016[38]
	+	2	Zhang & Chaban 2012[43] Bielemann 2015[47]
	-	0	
External validity (Q2b(III))	++	0	
	+	1	Ding2016[38] Zhang & Chaban 2012[43] Bielemann 2015[47]
	-	0	
check list for economic evaluation studies by Drummond and Jefferson 1996 (n=1)			
Validity	+	1	Queiroz 2020[53]

***** (++) All or most check list criteria fulfilled (>75%) fulfilled

For peer review only - http://bmjopen.bmj.com/site/about/guidelines.xhtml

** (+) some checklist criteria (50%-75%) fulfilled

*** (-) None or few checklist criteria (<50%) fulfilled

**** [48] is a case study based on a review, thus information on methods is not adequate to report on quality

Table 5: Evidence from economic studies related to physical activity in LMIC

**Evidence**	**studies**
**Economic burden**	
**Direct cost**	
UMC 8886(16.5%), LMC 1366(2.5%), LI 75(0.1%) (INT$ 100000, global %) in 2013	Ding 2016[38]
China 3.075INT$, Brazil 1.634billion INT$ in 2013	Ding 2016[38]
China 3.5 billion USD in 2007	Zhang & Chaban 2012[43]
Brazil hospital admission cost 0.73 billion USD in 2013	Bielemann 2015[47]
Inpatient and outpatient cost of physical inactivity in China 0.4 billion USD and 0.9 billion USD in 2000	Popkin 2006[51]
**Indirect cost** (INT $1000000, global %)	
UMC 3814(27.8%), LMC 1350(9.9%), 130(0.9%)	Ding 2016[38]
China 3.3 billion USD (2007)	Zhang & Chaban 2012[43]
**Association of physical a(in)activity and economic cost**	
**Association of physical (in)activity and direct health cost**	
Health care cost significantly higher for physically inactive individuals than active individuals	Turi 2015[40] Codongo 2015[42] Turi 2017[44] Patel 2011[46]
association of adherence to a physical activity program by employees of a private health care company and direct medical costs is inconclusive	Mitsunaga 2018[39]
**Association of physical (in)activity and productivity lost**	
Significant effect from Physical inactivity on productivity loss associated health problem	Araujo 2017[36]

For peer review only - http://bmjopen.bmj.com/site/about/guidelines.xhtml

Socioeconomic factors associated with physical activity participation and intensity	
negative association of income with PA	Cheah2017[37]Cheah 2017[41] Abdi 2014[50] Thiago 2016[49] Atkinson 2016[52]
Employment status is positively associated with PA	Cheah 2017[41] Chen 2015[48]
socioeconomic disparities associated with PA	Thiago 2016[49]
Association of PA and macroeconomic indicators<br>• PA positively associated with HDI<br>• PA negatively associated with Gini index	 Atkinson 2016[52]<br>Silva A.S 2015[45]
Attractiveness of LTPA* is the predictor of participation of university students in PA over the time and cost factor of PA	Abdi 2014[50]
**Economic evaluation studies**	
The cost-utility ratio was higher among those who reported taking medications(M) compared to group with medication and regular physical activity (PA),<br>• M only- $ 3.92 / QALY<br>• M+PA -$ 3.21 / QALY<br>• No on medication OR PA only- $ 0.12 / QALY<br>The incremental utility cost advantageous for the medication + regular physical activity group	Queiroz 2020 [53]

*leisure time physical activity

For peer review only - http://bmjopen.bmj.com/site/about/guidelines.xhtml

For peer review only

For peer review only - http://bmjopen.bmj.com/site/about/guidelines.xhtml

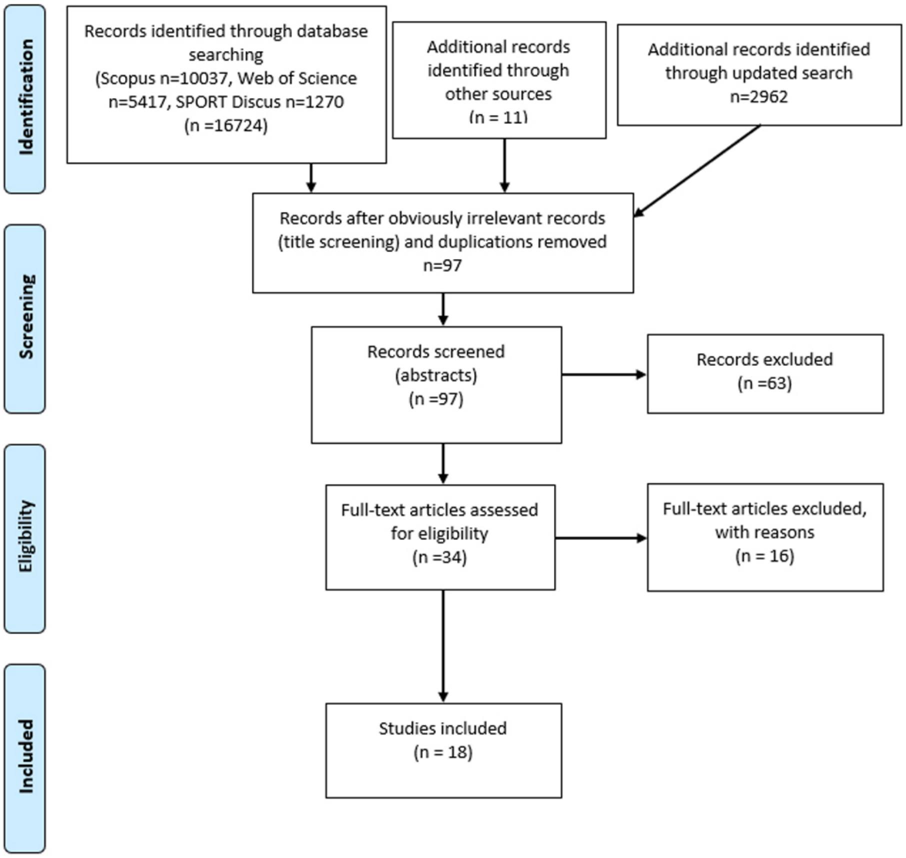

Figure 1: Flow diagram of article selection process

From: Moher D, Liberati A, Tetzlaff J, Altman DG, The PRISMA Group (2009). Preferred Reporting Items for Systematic Reviews and Meta-Analyses: The PRISMA Statement. PLoS Med 6(7): e1000097. doi:10.1371/journal.pmed1000097

Figure 1: Flow diagram of article selection process

90x90mm (300 x 300 DPI)

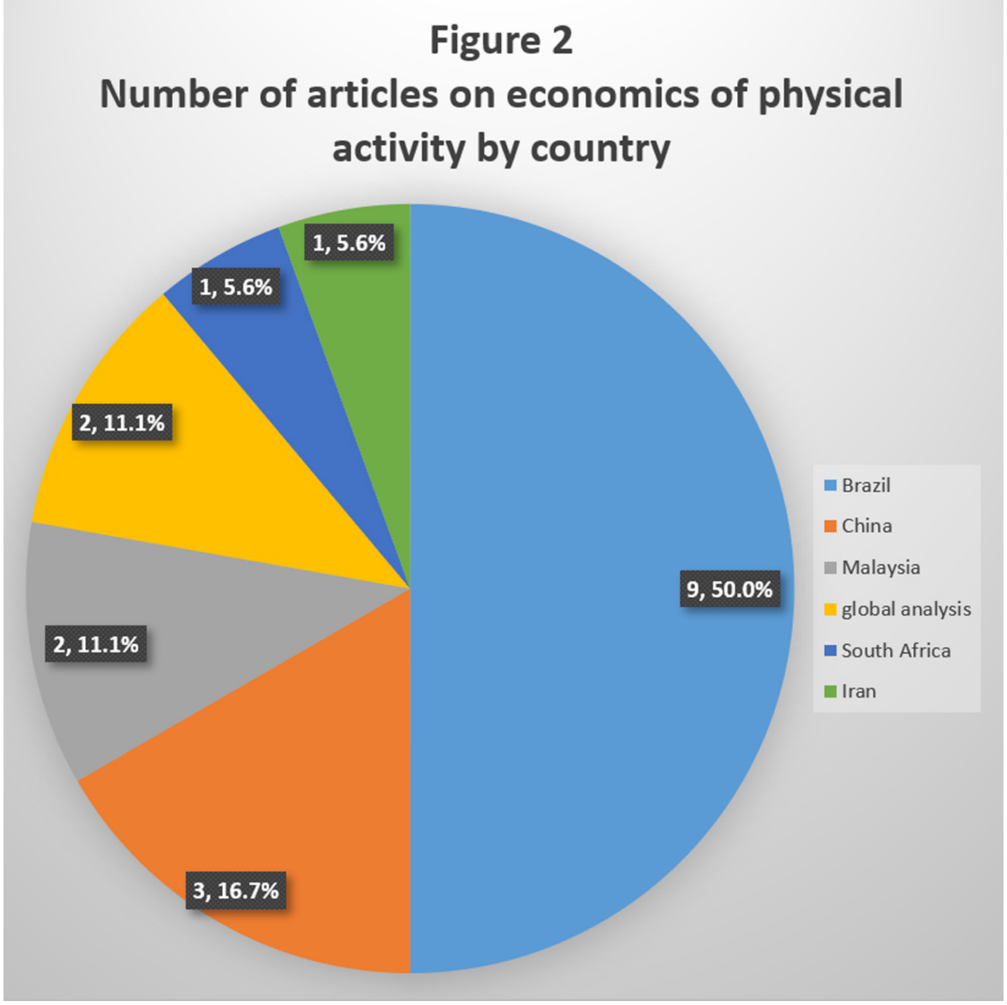

Figure 2: Number of articles on economics of physical activity by country

90x90mm (300 x 300 DPI)

Open access Protocol

BMJ Open

The economics of physical activity in low-income and middle-income countries: protocol for a systematic review

Priyanga Diloshini Ranasinghe,[1,2] Subhash Pokhrel,[3] Nana Kwame Anokye[3]

To cite: Ranasinghe PD, Pokhrel S, Anokye NK. The economics of physical activity in low-income and middle-income countries: protocol for a systematic review. *BMJ Open* 2019;**9**:e022686. doi:10.1136/bmjopen-2018-022686

► Prepublication history and additional material for this paper are available online. To view these files, please visit the journal online (http://dx.doi.org/10.1136/bmjopen-2018-022686).

Received 5 March 2018
Revised 9 October 2018
Accepted 23 November 2018

Check for updates

© Author(s) (or their employer(s)) 2019. Re-use permitted under CC BY-NC. No commercial re-use. See rights and permissions. Published by BMJ.

[1]Department of Health, Ministry of Health, Nutrition and Indigenous Medicine, Colombo, Sri Lanka
[2]Division of Health Sciences, Department of Clinical Sciences, Collage of Health and Life Sciences, Brunel University, London, UK
[3]Health Economics Theme, Institute of Environment, Health and Societies, Brunel University, London, UK

Correspondence to
Dr Priyanga Diloshini Ranasinghe;
priyangaran@yahoo.com,
Priyanga.Ranasinghe@brunel.ac.uk

ABSTRACT

Introduction Evidence on the economic costs of physical inactivity and the cost-effectiveness of physical activity interventions in low-income and middle-income countries (LMICs) is sparse, and fragmented where they are available. This is the first review aimed to summarise available evidence on economics of physical activity in LMICs, identify potential target variables for policy, and identify and report gaps in the current knowledge on economics of physical activity in LMICs.

Methods and analysis Peer-reviewed journal articles of observational, experimental, quasi-experimental and mixed-method studies on economics of physical activity in LMICs will be identified by a search of electronic databases; Scopus, Web of Science and SPORTDiscus. Websites of WHO, the National Institute for Health and Care Excellence international, World Bank and reference lists of included studies will be searched for relevant studies. The study selection process will be a two-stage approach; title and abstract screen for inclusion, followed by a review of selected full-text articles by two independent reviewers. Disagreements will be resolved by consensus and discussion with a third reviewer. Data will be extracted using standardised piloted data extraction forms. Risk of bias will be critically appraised using standard checklists based on study designs. Descriptive synthesis of data is planned. Where relevant, summaries of studies will be classified according to type of economic analysis, country or country category, population, intervention, comparator, outcome and study design. Meta-analysis will be performed where appropriate. This protocol for systematic review is prepared according to the Preferred Reporting Items for Systematic review and Meta-analysis for Protocols –2015 statement.

Ethics and dissemination Ethical approval is not obtained as original data will not be collected as part of this review. The completed review will be submitted for publication in a peer-reviewed journal and presented at conferences.

PROSPERO registration number CRD42018099856.

Strengths and limitations of this study

- This is the first synthesis of literature on economics of physical activity in low-income and middle-income countries (LMICs).
- This study provides evidence-based recommendations for economic research practice on physical activity.
- This review presents evidence on the business case for physical activity interventions for decision making by policy makers in LMIC settings.
- This review could suffer from publication bias as it excludes studies that are not peer reviewed.
- This review focuses on studies written in the English language and could miss out on relevant literature published in other languages.

INTRODUCTION

Rationale

Physical inactivity is a global health challenge, the fourth leading cause of mortality worldwide[1] and the key risk factor for non-communicable diseases (NCDs) such as cardiovascular diseases, cancer and diabetes.[2] Almost a third of the global health burden comprises NCDs which account for 1.4 billion disability-adjusted life years (DALYs).[3] At present most of the global NCD burden is from low-income and middle-income countries (LMICs) (as defined by[4] the World Bank, Classification 2017)[4] and this burden is largely attributable to levels of physical inactivity in the population.[5 6] Prevalence of physical inactivity in 2010 in upper middle-income countries was 25.4% (19.1%–33.7%), followed by 16.8% (11.8%–26.4%)%) in lower middle-income countries and 16.6% (11.8%–26.4%)%) in low-income countries showing that large populations in LMICs[7] are affected by physical inactivity and are therefore at risk of developing NCDs. The data available for the year 2004 show that 3.2 million deaths worldwide were due to physical inactivity of which 2.6 million were reported from LMICs.[8] LMICs have experienced urbanisation, shift of occupations from agriculture to industrial practice and modernisation including automation, and this has resulted in increased levels of physical inactivity in these populations.[9]

Increasing physical activity and reducing the burden of NCDs is the main goal of public health policies in LMICs.[10] It is a personal, corporate and government responsibility to

For peer review only

BMJ Open: first published as 10.1136/bmjopen-2018-022686 on 17 January 2019. Downloaded from http://bmjopen.bmj.com/ on August 18, 2020 at Sri Lanka:BMJ-PG Sponsored. Protected by copyright.

For peer review only - http://bmjopen.bmj.com/site/about/guidelines.xhtml

reduce the economic burden of unhealthy lifestyles in LMICs.[10] Economic analysis could uncover subtle characteristics of individual decision making on lifestyle choices, estimate the opportunity costs of not doing anything to improve population levels of physical activity and help evaluate what interventions work efficiently to achieve the goal of addressing the physical inactivity pandemic, particularly in LMICs.[11] The current focus of promoting physical activity has shifted from the traditional education approach to environment and policy approaches. Economic perspectives and public health perspectives complement each other in promotion of physical activity.[12] Economic perspectives inform the design of effective physical activity interventions (identifying economic barriers to physical activity) and the efficient allocation of resources—critical to the economies of LMICs.[12]

Our scoping exercise did not identify any published reviews that address the economics of physical activity in LMICs. Thus economic evidence on physical activity in LMICs could be limited or scattered.[13] Therefore, we designed this review to address the following research questions: (1) What is the available evidence base and research gaps on the economics of physical activity in LMICs? (2) What is the focus of and methods for underpinning economic research on physical activity in LMICs? (3) What are the target variables and cost-effective interventions for physical activity policy in LMICs?

This review will add to the scientific literature, provide an overview of the economic evidence base of physical activity in LMICs and fill the gaps in the available evidence regarding this. Providing an up-to-date synthesis of the economic evidence base is an efficient way of highlighting current research practices and new findings to inform researchers, and formulation of cost-effective physical activity programmes and policies.

Objectives

The review will:

- Summarise available evidence on economics of physical activity in LMICs.
- Describe the focus and methods underpinning research on economics of physical activity in LMICs.
- Identify potential target variables and cost-effective interventions for physical activity in LMICs for policy formation.
- Identify and report gaps in research on economics of physical activity in LMICs to provide recommendations for the economic research agenda in LMICs.

METHODS

The methods for this review were informed by previous reviews of economic analyses of physical activity[10 13–19] and are in line with recommendations on review of economic evidence.[20–22] This will be based on the 'Preferred Reporting Items for Systematic Review and Meta Analyses Protocols (PRISMA-P)' Statement[23] (online supplementary file 1).

Eligibility criteria

Economic studies on physical activity in LMICs will be included. Specifically, the following study types will be included: (1) Economic evaluations of physical activity interventions. (2) Economic burden of physical inactivity. (3) Cost of physical activity participation. (4) Demand for physical activity. (5) Economic correlates of physical (in) activity. Physical activity is defined as any bodily movement produced by skeletal muscles that require energy expenditure—including activities undertaken while walking, playing, carrying out household chores, travelling and engaging in recreational pursuits.[24] Physical inactivity is considered as a lack of physical activity.[25]

Physical activity interventions will not be specifically defined for this review in order to identify any physical activity intervention in LMICs. However, studies of which an economic analysis was carried out along with the intervention will be included in this review. This strategy will enable consolidation of all available evidence on economics of physical activity related to physical activity interventions.

Eligibility criteria are determined by relevant elements of the population, intervention, comparator, outcome, study design (PICOS criteria).[26] Interventions and comparators will be applicable only to intervention studies.

Inclusion criteria

- Study setting: Any setting of LMICs in accordance with the definition of LMIC by World Bank, Classification 2017.[4]
- Population: Any population in any age group across the life course.
- Intervention: Any physical activity intervention in which the economic evaluation of the intervention has been carried out.
- Comparator: Normal routine, no intervention.
- Outcomes: (1) Cost-effectiveness ratio, quality-adjusted life years (QUALY), incremental cost-effectiveness ratio assessed as the outcomes of physical activity interventions. (2) Cost of physical (in) activity in terms of healthcare cost and/or productivity loss and/or total cost of physical inactivity. (3) Measures of association of any economic variable with physical activity are defined as the primary outcomes of this review.
- Study design: Observational studies (cohort, case control, cross-sectional); correlational studies, experimental studies including randomised controlled trials; quasi-experimental studies; natural experiments; and economic evaluation studies.
- Studies reported only in the English language.

Exclusion criteria

- Case reports, case series, letters to the editor, editorials, reviews, qualitative studies, unpublished theses, conference abstracts and any unobtainable texts.
- Studies published in a language other than English.

For peer review only - http://bmjopen.bmj.com/site/about/guidelines.xhtml
BMJ Open: first published as 10.1136/bmjopen-2018-022686 on 17 January 2019. Downloaded from http://bmjopen.bmj.com/ on August 18, 2020 at Sri Lanka:BMJ-PG Sponsored. Protected by copyright.

Search strategy

The selection of databases was performed by a scoping review of methods of systematic reviews in the field.[13 17 18 27 28] We will search the following electronic databases to identify studies: Scopus (covers 100% MEDLINE coverage, 100% of EMBASE coverage and 100% of Compendex coverage[29]), Web of Science and SPORTDiscus. Websites of WHO, National Institute for Health and Care Excellence (NICE) international and World Bank will be searched for relevant studies. The reference lists of included studies will be searched for any relevant articles. Searches will include publications up to December 2017.

The search strategy was developed based on the scoping review that covered relevant reviews on economic studies on physical activity,[10 13–19] reviews on physical activity[27 30 31] and reviews on economic evaluations.[28 32 33] The draft search strategy was then reviewed by subject experts and a subject liaison librarian to optimise the sensitivity and specificity of the search. Online supplementary file 2 shows a sample search strategy.

Study selection

A two-stage approach involving three independent reviewers will be used to select relevant papers.

At the first stage, two reviewers (PDR and NKA) will independently screen the titles and abstracts of identified papers. Disagreement will be resolved through discussions with a third reviewer (SP). We will include a study if in doubt about its inclusion. For example, if the country setting of a study is unclear in the title and abstract, we will take it forward to the next stage.

At the second stage, the full text of papers selected from stage 1, will be reviewed independently by two reviewers (PDR and NKA). Selected and excluded papers, with reasons, will be discussed by the two reviewers and a third reviewer (SP) at a consensus meeting and disagreement will be resolved by real time consensus. Corresponding authors will be contacted via email for clarification if needed. The study selection process will be presented in a PRISMA flow chart[34] along with the reasons for the exclusion of studies.

Data management and extraction

Endnote X7 software will be used to manage the search results. Data extraction will be performed using a standardised pilot-tested data extraction form developed based on relevant data extraction forms from relevant reviews.[17 35 36] The adaptation process involved matching the review objectives with the available data extraction forms. Data from the final selected full-text articles will be extracted by one reviewer (PDR). To ensure quality of data extraction, a second reviewer (NKA) will independently extract data from a random selection of 50% of the final articles. Disagreements between the two reviewers will be discussed and resolved through discussions with the third reviewer (SP). Any unresolved disagreements will be reported in the final report. Online supplementary file 3 shows the draft data extraction form. Data will be extracted for the following items: general information, characteristics of the study, characteristics of the population/condition/intervention, data sources/data analysis/outcomes, conclusions and the way forward suggested by the authors, challenges and quality assessment.

Risk of bias and quality assessment

Risk of bias will be assessed by a one reviewer (PDR) for all the selected articles using standard checklists based on the study design. A second reviewer (NKA) will also independently assess the risk of bias on 50% of the randomly selected articles included.

Checklists are considered a reliable means of ensuring that all studies included are critically appraised in a standard way.[26] These will assess the information bias which could occur due to the methods, study design, data collection, data analysis and interpretation, and selection bias, where relevant. The most appropriate checklists for each expected study design for risk of bias assessment were selected based on relevant literature in this area[15 17 36–40] and by consensus with all three reviewers (online supplementary file 4). Risk of bias will be assessed using the following tools:

- Economic evaluation studies
  The Drummond checklist[41] which consists of 35 items will be used. It mainly assesses the quality of the study in relation to methods, study design, data collection, data analysis and interpretations. If an economic evaluation study is based on a decision analytical model, the Phillips[42] checklist will be used in addition.
- Cost of illness studies
  The Larg & Moss checklist[43] will be used to assess the quality of the study. It would assess the possible biases related to the analytical framework: what costs should have been measured, methodology and data; how well were resources used and productivity losses measured including representativeness of data for the study population, analysis and reporting. Both internal validity and external validity will be appraised.
- Studies on correlation or association
  The checklist recommended by NICE[44] will be used. Risk of bias related to sample selection, method of selection of exposure/comparison group, outcomes and analysis will be assessed. Both internal and external validity will be appraised.

Quality grading will be given to each included study based on the overall quality of the study. Grading for internal validity and external validity will be reported separately, where relevant. The grading process will follow the NICE recommendations[37] (figure 1):

- Good quality (++): almost all checklist criteria have been fulfilled; where they have not been fulfilled the conclusions are very unlikely to alter.
- Moderate quality (+): some of the checklist criteria are fulfilled; where they have not been fulfilled or not adequately described, conclusions are unlikely to alter.

For peer review only - http://bmjopen.bmj.com/site/about/guidelines.xhtml
BMJ Open: first published as 10.1136/bmjopen-2018-022686 on 17 January 2019. Downloaded from http://bmjopen.bmj.com/ on August 18, 2020 at Sri Lanka:BMJ-PG Sponsored. Protected by copyright.

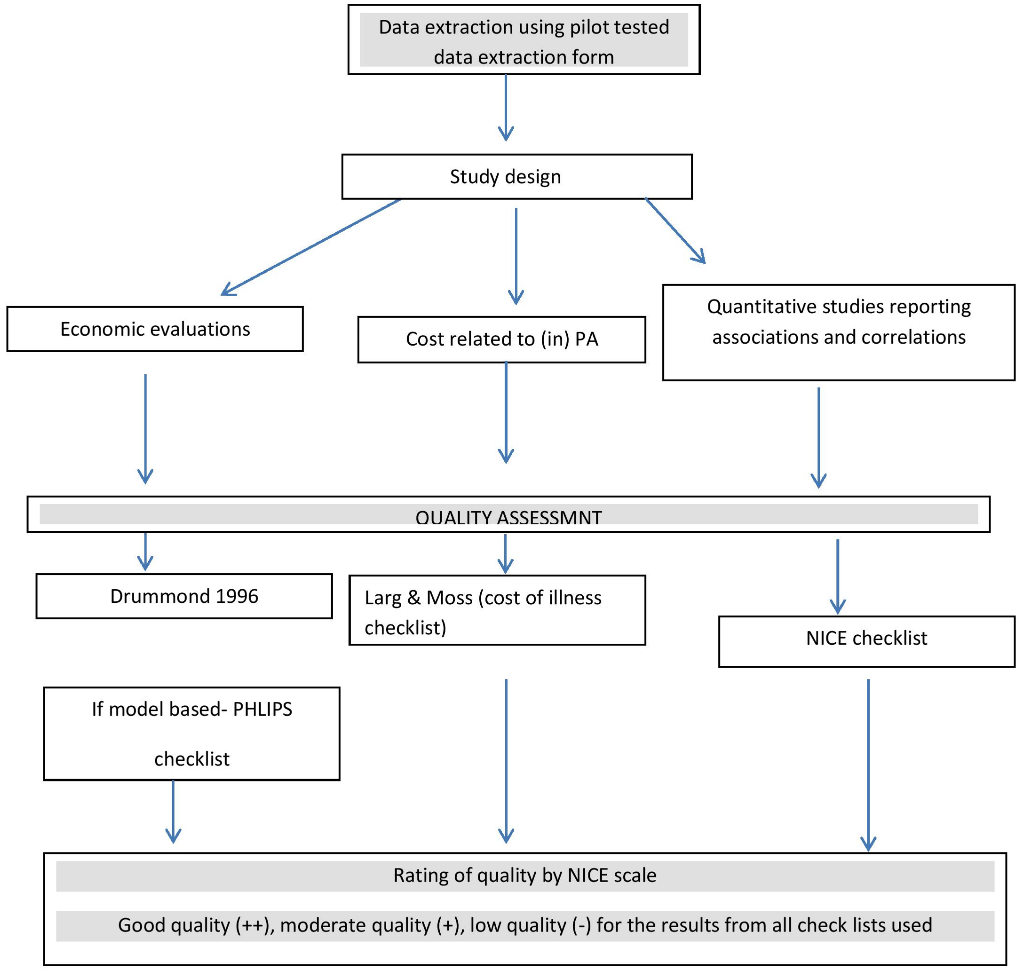

Figure 1 Economics of physical activity (PA) in low-income and middle-income countries (LMICs)—plan for data extraction and the quality assessment process. NICE, National Institute for Health and Care Excellence.

- Low quality (−): few or no checklist criteria are fulfilled, and conclusions are likely or very likely to alter.
- The quality of the included studies will be reported and critically appraised for each study. However, studies will not be excluded based on quality, as this review is particularly aimed to capture the evidence base on economics of physical activity in LMICs and appraise the methods underpinning the evidence base.

Data synthesis and reporting

A descriptive synthesis of data is planned due to the expected heterogeneity of the studies. Summaries of studies will be classified according to type of economic analysis, country or country category, characteristics of the study by PICOS criteria (where applicable); population, intervention, comparator, outcome, study design and quality of studies to describe the overview of available economic evidence base of physical activity in LMICs. Where the numbers permit, summaries of the studies will be categorised based on the focus of the study; economic burden of physical inactivity, economic analysis of physical activity interventions, economic correlates of physical activity including association of physical activity and cost (healthcare cost and/or productivity loss). Where appropriate, we will conduct a meta-analysis to quantify the effect sizes. Such analyses will adjust for between-study heterogeneity using random-effects models. The I-squared (I^2) statistic will be used to evaluate the heterogeneity related to the findings of the papers. Quantitative analysis will be conducted using STATA V.13 software.

We will report the number of studies where data extraction items were not applicable, as an indicator of the quality of reporting. Author-stated limitations of included studies and recommendations for future research and policy will be presented. This review will be reported in accordance with the PRISMA 2009 statement.[34] The study selection process will be illustrated by a flow diagram that will include the reasons for exclusion of studies at each stage. The search strategy, data extraction forms and quality assessment tools will be published as online supplementary files. This protocol will not be amended and any changes will be described and discussed in the final report.

For peer review only - http://bmjopen.bmj.com/site/about/guidelines.xhtml

BMJ Open: first published as 10.1136/bmjopen-2018-022686 on 17 January 2019. Downloaded from http://bmjopen.bmj.com/ on August 18, 2020 at Sri Lanka:BMJ-PG Sponsored. Protected by copyright.

Study status

A scoping review of economics of physical activity in LMICs was carried out in November 2017 to inform the methods of the current review. It identified systematic reviews conducted on economics of physical activity and the gaps in knowledge. It has not been submitted for publication. A review of economics of physical activity in LMICs was designed and methods were developed in December 2017. The search strategy was piloted in January 2018 and the protocol was developed in February 2018. The first submission of the protocol for peer review was on 1 March 2018. Data collection will be commenced upon approval of the protocol for publication and will be completed in 2 months.

Patient and public involvement

Patients and public are not directly involved in this study as original data will not be collected.

Best practice guidelines to conduct systematic reviews[33] will be followed.

DISCUSSION

The level of physical inactivity of the population in LMICs is likely to increase giving rise to a multitude of public health and economic consequences, including rising healthcare costs due to increased NCD-related treatments, increased DALYs and productivity losses. Economic research on physical activity from the perspective of LMICs should therefore be considered a priority. This is because increased physical inactivity leads to uncontrolled healthcare needs in LMICs, where resources (including that for healthcare) are limited and often shrinking in real terms. Pooling of the available research evidence on economics of physical activity in LMICs will thus reveal the current knowledge on economics of physical activity in LMICs, thereby guiding the future of national, regional and local policies around physical activity.

This will be the first study to review the evidence base on economics of physical activity in the context of LMICs which can be considered as the main strength of this study. This will identify gaps in knowledge to support future studies in this area. However, we will only include those studies that are published as peer-reviewed articles and are published in the English language. Thus, possibility of publication bias and language bias cannot be excluded. Furthermore, by assessing and reporting the quality of included studies using standard quality assessment checklists, the possibility of reporting bias of this review can be minimised.

WHO is in the process of drafting the 'Global action plan on physical activity (GAPPA) to be implemented from 2018 to 2030'.[45] This is a timely study as the evidence from this research will provide useful information for implementation and prioritise the actions of GAPPA in LMICs. Evidence from this research will be useful for policy makers and stakeholders dealing with physical activity promotion at individual, local, national, regional and global levels, with special reference to LMICs. Further, this review will be an important base for a research agenda on economics of physical activity in LMICs. This will be a useful guide for researchers to design research on economics and physical activity with sound methodology, based on the research needs of LMICs. Furthermore, information gathered from this research will guide funding agencies for effective allocation of resources.

Contributors SP and NKA developed the idea for the review with inputs from PDR. PDR wrote the first draft. SP and NKA revised the protocol. NKA will act as guarantor of the review.

Funding The authors have not declared a specific grant for this research from any funding agency in the public, commercial or not-for-profit sectors.

Competing interests None declared.

Patient consent for publication Not required.

Provenance and peer review Not commissioned; externally peer reviewed.

Open access This is an open access article distributed in accordance with the Creative Commons Attribution Non Commercial (CC BY-NC 4.0) license, which permits others to distribute, remix, adapt, build upon this work non-commercially, and license their derivative works on different terms, provided the original work is properly cited, appropriate credit is given, any changes made indicated, and the use is non-commercial. See: http://creativecommons.org/licenses/by-nc/4.0/.

REFERENCES

1. World Health Organization. *Global recommendations on physical activity for health*. Geneva: World Health Organization, 2010.
2. World Health Organization. Physical activity [Fact Sheet – Media centre]. 2017 http://www.who.int/mediacentre/factsheets/fs385/en/ (Accessed 6 Feb 2018).
3. Murray CJ, Barber RM, Foreman KJ, *et al*. Global, regional, and national disability-adjusted life years (DALYs) for 306 diseases and injuries and healthy life expectancy (HALE) for 188 countries, 1990-2013: quantifying the epidemiological transition. *Lancet* 2015;386:2145–91.
4. Bank W. World bank country and lending groups: country classification. 2017 https://datahelpdesk.worldbank.org/knowledgebase/articles/906519-world-bank-country-and-lending-groups (Accessed 1st Dec 2017).
5. Abegunde DO, Mathers CD, Adam T, *et al*. The burden and costs of chronic diseases in low-income and middle-income countries. *Lancet* 2007;370:1929–38.
6. Hallal PC, Andersen LB, Bull FC, *et al*. Global physical activity levels: surveillance progress, pitfalls, and prospects. *Lancet* 2012;380:247–57.
7. WHO. Prevalence of insufficient physical activity among adults, data by world bank income groups. 2015 http://apps.who.int/gho/data/view.main.2487?lang=en (Accessed 29th Jan 2018).
8. WHO. Global health risks: mortality and burden of disease attributable to selected major risks. ISBN 978 92 4 156387 1. 2009. Retrieved from http://www.who.int/healthinfo/global_burden_disease/GlobalHealthRisks_report_full.pdf (Accessed 25th Jan 2018).
9. Atkinson K, Lowe S, Moore S. Human development, occupational structure and physical inactivity among 47 low and middle income countries. *Prev Med Rep* 2016;3:40–5.
10. Oldridge NB. Economic burden of physical inactivity: healthcare costs associated with cardiovascular disease. *Eur J Cardiovasc Prev Rehabil* 2008;15:130–9.
11. Islam SM, Purnat TD, Phuong NT, *et al*. Non-communicable diseases (NCDs) in developing countries: a symposium report. *Global Health* 2014;10:81.
12. Sturm R. Economics and physical activity: a research agenda. *Am J Prev Med* 2005;28:141–9.
13. Ding D, Kolbe-Alexander T, Nguyen B, *et al*. The economic burden of physical inactivity: a systematic review and critical appraisal. *Br J Sports Med* 2017;51:1392–409.
14. Barte JCM, Wendel-Vos GCW. A systematic review of financial incentives for physical activity: the effects on physical activity and related outcomes. *Behav Med* 2017;43:79–90.
15. Abu-Omar K, Rütten A, Burlacu I, *et al*. The cost-effectiveness of physical activity interventions: a systematic review of reviews. *Prev Med Rep* 2017;8:72–8.

For peer review only - http://bmjopen.bmj.com/site/about/guidelines.xhtml

BMJ Open: first published as 10.1136/bmjopen-2018-022686 on 17 January 2019. Downloaded from http://bmjopen.bmj.com/ on August 18, 2020 at Sri Lanka:BMJ-PG Sponsored. Protected by copyright.

16. Brown V, Zapata Diomedi B, Moodie M, *et al*. A systematic review of economic analyses of active transport interventions that include physical activity benefits. *BMJ Open* 2016;45.
17. Pavey TG, Taylor AH, Fox KR, *et al*. Effect of exercise referral schemes in primary care on physical activity and improving health outcomes: systematic review and meta-analysis. *BMJ* 2011;343:d6462.
18. Campbell F, Holmes M, Everson-Hock E, *et al*. A systematic review and economic evaluation of exercise referral schemes in primary care: a short report. *Health Technol Assess* 2015;19:1–110.
19. Suhrcke M, Boluarte TA, Niessen L. A systematic review of economic evaluations of interventions to tackle cardiovascular disease in low- and middle-income countries. *BMC Public Health* 2012;12:2.
20. Kaltenthaler E, Tappenden P, Paisley S. Reviewing the evidence to inform the population of cost-effectiveness models within health technology assessments. *Value Health* 2013;16:830–6.
21. Zechmeister-Koss I, Schnell-Inderst P, Zauner G. Appropriate evidence sources for populating decision analytic models within health technology assessment (HTA): a systematic review of HTA manuals and health economic guidelines. *Med Decis Making* 2014;34:288–99.
22. NICE. *The guideline manual process and methods (PMG6)*: NICE, 2012.
23. Moher D, Shamseer L, Clarke M, *et al*. Preferred reporting items for systematic review and meta-analysis protocols (PRISMA-P) 2015 statement. [Research Support, Non-U S Gov't Research Support, U S Gov't, P H S]. *Syst Rev* 2015;4:2046–4053.
24. WHO. *Physical Activity*. [Fact sheet]. 2018 http://www.who.int/en/news-room/fact-sheets/detail/physical-activity (Accessed 15th Jan 2018).
25. WHO. Global Strategy on Diet, Physical activity and Health. 2018. Retrieved from https://www.who.int/dietphysicalactivity/pa/en/ (Accessed 6th Oct 2018).
26. University of York. *Systematic reviews: CRD's guidance for undertaking reviews in health care*. York: University of York, 2009.
27. Milton K, Macniven R, Bauman A. Review of the epidemiological evidence for physical activity and health from low- and middle-income countries. *Glob Public Health* 2014;9:369–81.
28. Nosratnejad S, Rashidian A, Dror DM. Systematic review of willingness to pay for health insurance in low and middle income countries. *PLoS One* 2016;11:e0157470.
29. Burnham JF. Scopus database: a review. *Biomed Digit Libr* 2006;3:1742–5581.
30. Lachat C, Otchere S, Roberfroid D, *et al*. Diet and physical activity for the prevention of noncommunicable diseases in low- and middle-income countries: a systematic policy review. *PLoS Med* 2013;10:e1001465.
31. Hunter RF, Christian H, Veitch J, *et al*. The impact of interventions to promote physical activity in urban green space: a systematic review and recommendations for future research. *Soc Sci Med* 2015;124:246–56.
32. Sutton L, Karan A, Mahal A. Evidence for cost-effectiveness of lifestyle primary preventions for cardiovascular disease in the Asia-Pacific Region: a systematic review. *Global Health* 2014;10:79.
33. Santatiwongchai B, Chantarastapornchit V, Wilkinson T, *et al*. Methodological variation in economic evaluations conducted in low- and middle-income countries: information for reference case development. *PLoS One* 2015;10:e0123853.
34. Moher D, Liberati A, Tetzlaff J, *et al*. Preferred reporting items for systematic reviews and meta-analyses: the PRISMA statement. [Guideline Research Support, Non-U S Gov't]. *J Clin Epidemiol* 2009;62:1006–12.
35. Pokhrel S, Quigley MA, Fox-Rushby J, *et al*. Potential economic impacts from improving breastfeeding rates in the UK. [Research Support, Non-U.S. Gov't]. *Arch Dis Child* 2015;100:334–40.
36. Murthy S, John D, Godinho IP, *et al*. A protocol for a systematic review of economic evaluation studies conducted on neonatal systemic infections in South Asia. *Syst Rev* 2017;6:252.
37. Aminde LN, Veerman L. Interventions for the prevention of cardiovascular diseases: a protocol for a systematic review of economic evaluations in low-income and middle-income countries. *BMJ Open* 2016;6:e013668.
38. Ruifrok AE, Rogozinska E, van Poppel MN, *et al*. Study protocol: differential effects of diet and physical activity based interventions in pregnancy on maternal and fetal outcomes--individual patient data (IPD) meta-analysis and health economic evaluation. *Syst Rev* 2014;3:131.
39. Gc V, Wilson EC, Suhrcke M, *et al*. Are brief interventions to increase physical activity cost-effective? A systematic review. *Br J Sports Med* 2016;50:408–17.
40. Tonmukayakul U, Calache H, Clark R, *et al*. Systematic review and quality appraisal of economic evaluation publications in dentistry. *J Dent Res* 2015;94:1348–54.
41. Drummond MF, Jefferson TO. Guidelines for authors and peer reviewers of economic submissions to the BMJ. *BMJ* 1996;313:275–83.
42. Philips Z, Ginnelly L, Sculpher M, *et al*. Review of guidelines for good practice in decision-analytic modelling in health technology assessment. *Health Technol Assess* 2004;8:1–158.
43. Larg A, Moss JR. Cost-of-illness studies: a guide to critical evaluation. [Research Support, Non-U S Gov't]. *Pharmacoeconomics* 2011;29:653–71.
44. National Institute for Health and Excellence (NICE). Methods for the development of NICE public health guidance (third edition), Appendix G Quality appraisal checklist-quantitative studies reporting correlations and associations. 2012 https://www.nice.org.uk/process/pmg4/chapter/appendix-g-quality-appraisal-checklist-quantitative-studies-reporting-correlations-and (Accessed 2nd Jan 2018).
45. World Health Organization. *Non communicable diseases and their risk factors, governance*: Development of draft global action plan to promote physical activity, 2018.

For peer review only

BMJ Open: first published as 10.1136/bmjopen-2018-022686 on 17 January 2019. Downloaded from http://bmjopen.bmj.com/ on August 18, 2020 at Sri Lanka:BMJ-PG Sponsored. Protected by copyright.

For peer review only - http://bmjopen.bmj.com/site/about/guidelines.xhtml

Search Strategy

Scopus

((TITLE-ABKEY

((*cost* W/2 (*analyses* OR *effectiveness* OR *benefit* OR *minimization* OR *utility* OR *saving* OR *outcome* OR *consequence*))) AND DOCTYPE (*ar* OR *re*)) OR (TITLE-ABS-KEY ((*"demand"* OR *"supply"* OR *"market"* OR *"price"*)) AND DOCTYPE (*ar* OR *re*)) OR (TITLE-ABS-KEY ((*"incentives"* OR *"willingness to pay"* OR *"time use"*)) AND DOCTYPE (*ar* OR *re*)) OR (TITLE-ABS-KEY (*"economics"*) AND DOCTYPE (*ar* OR *re*)) OR (TITLE-ABS-KEY ((*"economic evaluation"* OR *"economic burden"* OR *"health care cost"*)) AND DOCTYPE (*ar* OR *re*))) AND ((TITLE-ABS-KEY (((((*active* W/2 (*lifestyle* OR *living* OR *leisure* OR *play* OR *recreation* OR *commuting*)))))) AND DOCTYPE (*ar* OR *re*)) OR (TITLE-ABS-KEY ((*"physical activity"* OR *"physical exercise"*)) AND DOCTYPE (*ar* OR *re*)) OR (TITLE-ABS-KEY ((*"physical inactivity"* OR *"physical fitness"*)) AND DOCTYPE (*ar* OR *re*)) OR (TITLE-ABS-KEY (*"Sedentary"*) AND DOCTYPE (*ar* OR *re*))) AND (LIMIT-TO (LANGUAGE , *"English"*))

SPORTDiscus

((TITLE-ABS-KEY ((*cost* n/2 (*analyses* OR *effectiveness* OR *benefit* OR *minimization* OR *utility* OR *saving* OR *outcome* OR *consequence*))) AND DOCTYPE (*ar* OR *re*)) OR (TITLE-ABS-KEY ((*"demand"* OR *"supply"* OR *"market"* OR *"price"*)) AND DOCTYPE (*ar* OR *re*)) OR (TITLE-ABS-KEY ((*"incentives"* OR *"willingness to pay"* OR *"time use"*)) AND DOCTYPE (*ar* OR *re*)) OR (TITLE-ABS-KEY (*"economics"*) AND DOCTYPE (*ar* OR *re*)) OR (TITLE-ABS-KEY ((*"economic evaluation"* OR *"economic burden"* OR *"health care cost"*)) AND DOCTYPE (*ar* OR *re*))) AND ((TITLE-ABS-KEY (((((*active* n/2 (*lifestyle* OR *living* OR *leisure* OR *play* OR *recreation* OR *commuting*)))))) AND DOCTYPE (*ar* OR *re*)) OR (TITLE-ABS-KEY ((*"physical activity"* OR *"physical exercise"*)) AND DOCTYPE (*ar* OR *re*)) OR (TITLE-ABS-KEY ((*"physical inactivity"* OR *"physical fitness"*)) AND DOCTYPE (*ar* OR *re*)) OR (TITLE-ABS-

For peer review only

KEY (*"Sedentary"*) AND DOCTYPE (*ar* OR *re*))) AND (LIMIT-TO (LANGUAGE , *"English"*))

Web of Science

((TITLE-ABS-KEY ((*cost* near/15 (*analyses* OR *effectiveness* OR *benefit* OR *minimization* OR *utility* OR *saving* OR *outcome* OR *consequence*))) AND DOCTYPE (*ar* OR *re*)) OR (TITLE-ABS-KEY ((*"demand"* OR *"supply"* OR *"market"* OR *"price"*)) AND DOCTYPE (*ar* OR *re*)) OR (TITLE-ABS-KEY ((*"incentives"* OR *"willingness to pay"* OR *"time use"*)) AND DOCTYPE (*ar* OR *re*)) OR (TITLE-ABS-KEY (*"economics"*) AND DOCTYPE (*ar* OR *re*)) OR (TITLE-ABS-KEY ((*"economic evaluation"* OR *"economic burden"* OR *"health care cost"*)) AND DOCTYPE (*ar* OR *re*))) AND ((TITLE-ABS-KEY (((((*active* near/15 (*lifestyle* OR *living* OR *leisure* OR *play* OR *recreation* OR *commuting*)))))) AND DOCTYPE (*ar* OR *re*)) OR (TITLE-ABS-KEY ((*"physical activity"* OR *"physical exercise"*)) AND DOCTYPE (*ar* OR *re*)) OR (TITLE-ABS-KEY ((*"physical inactivity"* OR *"physical fitness"*)) AND DOCTYPE (*ar* OR *re*)) OR (TITLE-ABS-KEY (*"Sedentary"*) AND DOCTYPE (*ar* OR *re*))) AND (LIMIT-TO (LANGUAGE , *"English"*))

For peer review only

Table 1: List of excluded studies following full text screening with reasons

1. Is Exercise a Good Investment for Adolescents? (1991). Journal of Sport & Exercise Psychology, 13(2), 208-208.	It is a brief one page document and not a research article. country studied also not mentioned
2. Codogno, J. S., Fernandes, R. A., & Monteiro, H. L. (2012). Physical activity and healthcare cost of type 2 diabetic patients seen at basic units of healthcare. [Article]. Arquivos Brasileiros de Endocrinologia e Metabologia, 56(1), 6-11	Article in Portuguese. (though English abstract available)
3. Ng, S. W., & Popkin, B. M. (2012). Time use and physical activity: A shift away from movement across the globe. [Article]. Obesity Reviews, 13(8), 659-680. doi: 10.1111/j.1467-789X.2011.00982.x	It only reports trends in energy expenditure including sedentary time and various physical activities for brazil, India and china and 2 other developed countries. It does not report how time affect the physical activity.
4. Oldridge, N. B. (2008). Economic burden of physical inactivity: healthcare costs associated with cardiovascular disease. European Journal of Cardiovascular Prevention & Rehabilitation, 15(2), 130-139. doi: 10.1097/HJR.0b013e3282f19d42	Though prevalence estimated for developing countries. cost is estimated only for developed countries
5. Kolbe-Alexander, T. L., Conradie, J., & Lambert, E. V. (2013). Clustering of risk factors for non-communicable disease and healthcare expenditure in employees with private health insurance presenting for health risk appraisal: a cross-sectional study. Bmc Public Health, 13. doi: 10.1186/1471-2458-13-1213	Studied clustering of other NCD risk factors with Physical inactivity, and association of clustered risk factors with health care expenditure. Data on expenditure due to physical inactivity not shown separately.
6. Huang, W. Y., & Wong, S. H. (2016). Time use clusters in children and their associations with sociodemographic factors. [Article]. Journal of Public Health (United Kingdom), 38(2), e106-e113. doi: 10.1093/pubmed/fdv088	Identified clusters according to time use for physical activity and sedentary behaviour. It does not assess time effect on physical activity participation.
7. Jim, C. Y., & Chen, W. Y. (2009). Leisure Participation Pattern of Residents in a New Chinese City. Annals of the Association of American Geographers, 99(4), 657-673. doi: 10.1080/00045600903066482	Assessed the association of leisure time and socioeconomic variables. Barriers to leisure participation identified by using a part of the survey questionnaire as a part of this study. HOWEVER, ASSESSED ONLY THE PERCIEVED BARRIERS.
8. Lopes, A. S., Silva, K. S., Filho, V. C. B., Bezerra, J., De Oliveira, E. S. A., & Nahas, M. V. (2014). Trends in screen time on week and weekend days in a representative sample of Southern Brazil students. [Article]. Journal of Public Health (United Kingdom), 36(4), 608-614. doi: 10.1093/pubmed/fdt133	It basically reports the trend of sedentary screen time.
9. Matsudo, V. K. R., Matsudo, S. M., Araújo, T. L., Andrade, D. R., Oliveira, L. C., & Hallal, P. C. (2010). Time Trends in physical activity in the state of São Paulo, Brazil: 2002-2008. [Article].	Reported only the trend in PA participation by socioeconomic status

For peer review only

Medicine and Science in Sports and Exercise, 42(12), 2231-2236. doi: 10.1249/MSS.0b013e3181e1fe8e	
10. Pratt, M., Norris, J., Lobelo, F., Roux, L., & Guijing, W. (2014). The cost of physical inactivity: moving into the 21st century. British Journal of Sports Medicine, 48(3), 171-173.	This is a review
11. Araujo, M. Y. C., et al. (2016). "Association between disability pension, nutritional condition and physical inactivity in adults from a middle-size Brazilian City." Journal of Physical Education (Maringa) 27(1).	Not directly relevant to economics of physical activity as it assesses the association of disability pension and physical activity.
12. Sujatha, T., et al. (2003). "Timed activity studies for assessing the energy expenditure of women from an urban slum in south India." Food and Nutrition Bulletin 24(2): 193-199.	It does not assess the time effect on physical activity
13. Cecchini, M., et al. "Tackling of unhealthy diets, physical inactivity, and obesity: health effects and cost-effectiveness." The Lancet 376(9754): 1775-1784.	This is only a report, proposed a model and analysed cost for interventions for all healthy life styles by intervention type. Not assessed the cost for physical activity promotion by interventions separately or economic evaluation of such intervention
14. Meng, L., et al. (2013). "The Costs and Cost-Effectiveness of a School-Based Comprehensive Intervention Study on Childhood Obesity in China." PLOS ONE 8(10): e77971.	Cost effectiveness assessed only for combined intervention, not for physical activity alone
15. Moraisa. L.C., et al. Health indicators and costs among outpatients according to physical activity level and obesity. *Diabetes & Metabolic Syndrome: Clinical Research & Reviews,* Volume 13, Issue 2, March–April 2019, Pages 1375-137	This has not assessed physical activity separately but coupled with obesity
16. Min, J. Y. and K. B. Min (2016). Excess Medical Care Costs Associated with Physical Inactivity among Korean Adults: Retrospective Cohort Study. *International Journal of Environmental Research and Public Health,* **13**(1).	This study directly does not indicate which part of the Korea. However, the insurance scheme in this study is functioning in South Korea, which is a high-income country.

For peer review only

DATA EXTRACTION FORM FOR REVIEW ON ECONONOMICS AND PYSICAL ACTIVITY IN LOW AND MIDDLE INCOME COUNTRIES (ADAPTED FROM MURTHEY ET AL., 2017, POKHREL ET AL., 2014, PAVEY ET AL., 2011)

**1. GENERAL INFORMATION**		
1.1	Study ID	
1.2	Review author ID	
1.3	Date of review	
1.4	Title	
1.5	Author(s)	
1.6	Year of publication	
1.7	Country studied	
1.8	Income level of the country	
**2. CHRACTERISTIC OF THE STUDY**		
2.1	Aims and objectives/research question	
2.2	Eligibility decision Inclusion and exclusion criteria (?)	
2.3	Study design	
2.4	Study setting	
2.5	Total study duration	
2.6	Sample size	
2.7	Sample size calculation	
2.8	Recruitment	
2.9	Sample selection	
2.10	Method of allocation	
2.11	Perspective (i.e. Societal, health care system, service provider etc.)	
**3. CHARACTERISTICS OF POPULATION/CONDITION/INTERVENTION**		
3.1	**Population/participants**	
3.1.1	Type of population	

For peer review only

	(i.e. General population university students, group of employees)	
3.1.2	Age	
3.1.3	Gender	
3.1.4	Ethnicity	
3.2	**Condition**	
3.2.1	Definition of physical activity /physical inactivity/sedentary	
3.2.2	Definition of disease condition	
3.3	**Intervention**	
3.3.1	Type of physical activity/programme	
3.3.2	Comparator	
4.	**Data sources/Data analysis/results**	
4.1	**scope**	
4.1.1	Type of analysis (Ie.Costing/economic evaluation/association of economic factors with physical activity/other)	
4.1.2	Form of economic evaluation	
4.1.3	Time horizon	
4.1.4	Study assumptions	
4.2	**Cost**	
4.2.1	Reported costs/cost components	
4.2.2	Data sources for cost measures	
4.2.3	How cost was reported (i.e. marginal cost/ average cost/incremental cost/total cost/other)	
4.2.4	Discount rate	
4.2.5	Year of costing	
4.2.6	Currency unit	
4.2.7	Currency conversion rate	

For peer review only

4.2.8	Inflation adjustments	
4.3	**Outcomes**	
4.3.1	Reported outcomes (i.e. measures of effectiveness/efficacy/measures of effect)	
4.3.2	Data sources for the outcome measures	
4.3.4	Discount rate	
4.4	**Main Analysis**	
4.4.1	Statistical tests used	
4.4.2	Models used	
4.4.3	Economic evaluations	
4.5	**Sensitivity analysis**	
4.5.1	Performed/ not	
4.5.2	Type of analysis (i.e. deterministic/ stochastic)	
4.5.3	Variables used for sensitivity analysis	
4.5.4	Results of sensitivity analysis	
4.6	**Main results**	
4.6.1	Cost / cost effectiveness	
4.6.2	Other	
5.	**CONCLUSIONS AND WAY FORWARD**	
5.1	Key Conclusion(s)	
5.2	Suggestions by author for further research	
6.	**CHALLENGES**	
6.1	Author stated limitations	
6.2	Author stated strengths	
6.3	Miscellaneous comments from author	
6.4	Funding source	
6.5	Conflicts of interest	
**7.**	**QUALITY ASSESSMENT OF STUDIES**	

For peer review only

7.1	Is the study economic evaluation?	Yes/No, if *yes go to* Check list A
7.2	Is the economic evaluation model based?	Yes/No , if *yes* go to Check list B
7.3	Is it a cost of illness/cost of risk factor study?	Yes/No , if *yes* go to Check list C
7.4	Is it a quantitative study reporting correlation or association?	Yes/No , If *yes* go to Checklist D

**Method of rating the quality of studies from each check list (A, B, C, D) in accordance to the quality appraisal by NICE scale (Methods for the development of NICE public health guidance (third edition))**	
• Reviewer is requested to complete in detail the comment section of quality appraisal check lists so that the grade awarded for each study aspect is as transparent as possible<br>• At the end of completion of the check list each study is awarded an overall quality grading for internal validity (IV)and a separate one for external validity (EV)	
All or most of the checklist criteria have been fulfilled, where they have not been fulfilled the conclusions are very unlikely to alter	++
Some of the checklist criteria have been fulfilled, where they have not been fulfilled, or not adequately described, the conclusions are unlikely to alter	+
Few or no checklist criteria have been fulfilled and the conclusions are likely or very likely to alter.	-

For peer review only

CHECKLIST A

QUALITY ASSESSMENT FOR ECONOMIC EVALUATION(CHECKLIST FROM DRUMMOND AND JEFFERSON 1996)	Yes	No	Not clear	Not appropriate
**Study design**				
1) The research question is stated				
2) The economic importance of the research question is stated				
3) The viewpoint(s) of the analysis are clearly stated and justified				
4) The rationale for choosing the alternative programmes orb interventions compared is stated				
5) The alternatives being compared are clearly described				
6) The form of economic evaluation used is stated				
7) The choice of form of economic evaluation is justified in relation to the questions addressed				
**Data collection**				
8) The source(s) of effectiveness estimates used are stated				
9) Details of the design and results of effectiveness study are given (if based on a single study)				
10) Details of the method of synthesis or meta-analysis of estimates are given (if based on an overview of a number of effectiveness studies)				
11) The primary outcome measure(s) for the economic evaluation are clearly stated				
12) Methods to value health states and other benefits are stated				

For peer review only

QUALITY ASSESSMENT FOR ECONOMIC EVALUATION(CHECKLIST FROM DRUMMOND AND JEFFERSON 1996)	Yes	No	Not clear	Not appropriate
13) Details of the subjects from whom valuations were obtained are given				
14) Productivity changes (if included) are reported separately				
15) The relevance of productivity changes to the study question is discussed				
16) Quantities of resources are reported separately from their unit costs				
17) Methods for the estimation of quantities and unit costs are described				
18) Currency and price data are recorded				
19) Details of currency of price adjustments for inflation or currency conversion are given				
20) Details of any model used are given				
21) The choice of model used and the key parameters on which it is based are justified				
**Analysis and interpretation of results**				
22) Time horizon of costs and benefits is stated				
23) The discount rate(s) is stated				
24) The choice of rate(s) is justified				
25) An explanation is given if costs or benefits are not discounted				
26) Details of statistical tests and confidence intervals are given for stochastic data				

For peer review only

QUALITY ASSESSMENT FOR ECONOMIC EVALUATION(CHECKLIST FROM DRUMMOND AND JEFFERSON 1996)	Yes	No	Not clear	Not appropriate
27) The approach to sensitivity analysis is given				
28) The choice of variables for sensitivity analysis is justified				
29) The ranges over which the variables are varied are stated				
30) Relevant alternatives are compared				
31) Incremental analysis is reported				
32) Major outcomes are presented in a disaggregated as well as aggregated form				
33) The answer to the study question is given				
34) Conclusions follow from the data reported				
35) Conclusions are accompanied by the appropriate caveats				

Did the economic evaluation use a decision-analytic modelling framework?	Response: Yes(✓), No (×) , Not applicable (NA)<br><br>Instruction: If *Yes*, assess paper using the questions in Check list B, If *No/NA*, skip check list B

CHECKLIST B

For peer review only

QUALITY ASSESSMENT FOR DECISION-ANALYTIC MODELS(CHECKLIST FROM PHILIPS ET AL. 2004)			
**Quality criterion**	**Question(s)**	**Response (✓, ×, NA)**	**Comments**
S1	Is there a clear statement of the decision problem?		
	Is the objective of the evaluation and model specified and consistent with the stated decision problem?		
	Is the primary decision maker specified?		
S2	Is the perspective of the model stated clearly?		
	Are the model inputs consistent with the stated perspective?		
	Has the scope of the model been stated and justified?		
	Are the outcomes of the model consistent with the perspective, scope and overall objective of the model?		
S3	Is the structure of the model consistent with a coherent theory of the health condition under evaluation?		
	Are the sources of data used to develop the structure of the model specified?		
	Are the causal relationships described by the model structure justified appropriately?		
S4	Are the structural assumptions transparent and justified?		

For peer review only

QUALITY ASSESSMENT FOR DECISION-ANALYTIC MODELS(CHECKLIST FROM PHILIPS ET AL. 2004)			
**Quality criterion**	**Question(s)**	**Response (✓, ✗, NA)**	**Comments**
	Are the structural assumptions reasonable given the overall objective, perspective and scope of the model?		
S5	Is there a clear definition of the options under evaluation?		
	Have all feasible and practical options been evaluated?		
	Is there justification for the exclusion for the exclusion of feasible options?		
S6	Is the chosen model type appropriate given the decision problem and specified causal relationships within the model?		
S7	Is the time horizon of the model sufficient to reflect all important differences between options?		
	Are the time horizon of the model, the duration of treatment and the duration of treatment effect described and justified?		
S8	Do the disease states (state transition model) or the pathways (decision tree model) reflect the underlying biological process of the disease in question and the impact of interventions?		
S9	Is the cycle length defined and justified in terms of natural history of disease?		

For peer review only

QUALITY ASSESSMENT FOR DECISION-ANALYTIC MODELS(CHECKLIST FROM PHILIPS ET AL. 2004)			
**Quality criterion**	**Question(s)**	**Response (✓, ×, NA)**	**Comments**
D1	Are the data identification methods transparent and appropriate given the objectives of the model?		
	Where choices have been made between data sources, are these justified appropriately?		
	Has particular attention been paid to identifying data for the important parameters in the model?		
	Has the quality of the data been assessed appropriately?		
	Where expert opinion has been used, are the methods described and justified?		
D2	Is the data modelling methodology based on justifiable statistical and epidemiological techniques?		
D2a	Is the choice of baseline data described and justified?		
	Are transition probabilities calculated appropriately?		
	Has a half-cycle correction been applied to both cost and outcome?		
	If not, has this omission been justified?		

For peer review only

QUALITY ASSESSMENT FOR DECISION-ANALYTIC MODELS(CHECKLIST FROM PHILIPS ET AL. 2004)			
**Quality criterion**	**Question(s)**	**Response (✓, ✗, NA)**	**Comments**
D2b	If relative treatment effects have been derived from trail data, have they been synthesised using appropriate techniques?		
	Have the methods and assumptions used to extrapolate short-term results to final outcomes been documented and justified?		
	Have alternative extrapolation assumptions been explored through sensitivity analysis?		
	Have assumptions regarding the continuing effect of treatment once treatment is complete been documented and justified?		
	Have alternative assumptions regarding the continuing effect of treatment been explored through sensitivity analysis?		
D2c	Are the costs incorporated into the model justified?		
	Has the source for all costs been described?		
	Have discount rates been described and justified given the target decision-maker?		
D2d	Are the utilities incorporated into the model appropriate?		
	Is the source for the utility weights referenced?		

For peer review only

QUALITY ASSESSMENT FOR DECISION-ANALYTIC MODELS(CHECKLIST FROM PHILIPS ET AL. 2004)			
**Quality criterion**	**Question(s)**	**Response (✓, ✗, NA)**	**Comments**
	Are the methods of derivation for the utility weights justified?		
D3	Have all data incorporated into the model been described and referenced in sufficient detail?		
	Has the use of mutually inconsistent data been justified (i.e. are assumptions and choices appropriate)?		
	Is the process of data incorporation transparent?		
	If data have been incorporated as distributions, has the choice of distribution for each parameter been described and justified?		
	If data have been incorporated as distributions, is it clear that second order uncertainty is reflected?		
D4	Have the four principal types of uncertainty been addressed?		
	If not, has the omission of particular forms of uncertainty been justified?		
D4a	Have methodological uncertainties been addressed by running alternative versions of the model with different methodological assumptions?		

For peer review only

QUALITY ASSESSMENT FOR DECISION-ANALYTIC MODELS(CHECKLIST FROM PHILIPS ET AL. 2004)			
**Quality criterion**	**Question(s)**	**Response (✓, ✗, NA)**	**Comments**
D4b	Is there evidence that structural uncertainties have been addressed via sensitivity analysis?		
D4c	Has heterogeneity been dealt with by running the model separately for different subgroups?		
D4d	Are the methods of assessment of parameter uncertainty appropriate?		
	If data are incorporated at point estimates, are the ranges used for sensitivity analysis stated clearly and justified?		
C1	Is there evidence that the mathematical logic of the model has been tested thoroughly before use?		
C2	Are any counterintuitive results from the model explained and justified?		
	If the model has been calibrated against independent data, has any differences been explained and justified?		
	Have the results of the model been compared with those of previous models and any differences in results explained?		

For peer review only

CHECKLIST C

QUALITY ASSESSMNET FOR COST OF ILLNESS STUDIES (ADAPTED FROM LARG AND MOSS, 2011)			
Responses (marked Similar to NICE check list, write the answer where relevant under the comment section)<br><br>++ Indicates that for that particular aspect of study design, the study has been designed or conducted in such a way as to minimise the risk of bias.<br>+ Indicates that either the answer to the checklist question is not clear from the way the study is reported, or that the study may not have addressed all potential sources of bias for that particular aspect of study design.<br>- Should be reserved for those aspects of the study design in which significant sources of bias may persist.<br>NR (Not Reported) - Should be reserved for those aspects in which the study under review fails to report how they have (or might have) been considered.<br>NA (Not Applicable) Should be reserved for those study design aspects that are not applicable given the study design under review (for example, allocation concealment would not be applicable for case–control studies).			
	Question	Response ***++/+/-NR/NA***	Comment
1.	Analytical framework: what costs should have been measured?<br>(a) What was the motivation and perspective of the study?<br>(b) Was the appropriate epidemiologic approach taken?<br>(c) Was the study question well specified?<br>(i) Were all relevant, non-trivial cost components and their stakeholders identified?<br>(ii) Were necessary timeframes specified?<br>(iii) Was a case of disease or risk factor adequately and appropriately defined?<br>(iv) Was the counterfactual population occurrence plausible and meaningful?		
2.	(2) Methodology and data: how well were resource use and productivity losses measured?<br>(a) Was an appropriate method(s) of quantification used, such that;		

	(i) Additional, or excess, costs were measured?<br>(ii) only costs specific to (caused by) the health problem were included (confounders controlled)?<br>(iii) All important effects were captured?<br>(iv) Important differences across subpopulations were accounted for?<br>(v) The required level of detail could be provided?<br><br>(b) Was the resource quantification method(s) well executed?<br>(i) For population-based studies, were cost allocation methods, data and assumptions valid?<br>(ii) For person-based studies, were appropriate statistical tests performed and reported?<br>(iii) Were data representative of the study population?<br>(iv) Were there any other relevant resource quantification issues?<br><br>(c) Were healthcare resources valued appropriately?<br><br>(d) Was the approach for valuing production losses justified, and assumptions valid?<br><br>(e) Was the inclusion of intangible costs appropriate:<br>(i) Was double counting of mortality-related production losses avoided?<br>(ii) Were losses valued appropriately, given the study's perspective?		
3	Analysis and reporting<br>(a) Did the analysis address the study question?<br><br>(b) Was a range of estimates presented?<br><br>(c) Were the main uncertainties identified?		

For peer review only

	(d) Was a sensitivity analysis performed on:<br><br>(i) important (uncertain) parameter estimates?<br>(ii) key assumptions? (including the counterfactual)<br>(iii) point estimates? (based on confidence or credible intervals)<br><br>(e) Was adequate documentation and justification given for cost components, data and sources, assumptions and methods?<br><br>(f) Was uncertainty around the estimates and its implications adequately discussed?<br><br>(g) Were important limitations discussed regarding the cost components, data, assumptions and methods?<br><br>(h) Were the results presented at the appropriate level of detail to answer the study<br>question (cost components; disease subtypes, severity, stage; subpopulation groups, cost bearers)?		

For peer review only

CHECKLIST D

**QUALITY ASSESSMENT FOR QUANTITATIVE STUDIES REPORTING CORRELATIONS AND ASSOCIATIONS(NICE,2012 BASED ON 'GRAPHICAL APPRAISAL TOOL FOR EPIDEMIOLOGICAL STUDIES(GATE)` BY JACKSON ET AL. (2006)**			
Responses:<br><br>++ Indicates that for that particular aspect of study design, the study has been designed or conducted in such a way as to minimise the risk of bias.<br><br>+ Indicates that either the answer to the checklist question is not clear from the way the study is reported, or that the study may not have addressed all potential sources of bias for that particular aspect of study design.<br><br>- Should be reserved for those aspects of the study design in which significant sources of bias may persist.<br><br>NR (Not Reported) - Should be reserved for those aspects in which the study under review fails to report how they have (or might have) been considered.<br><br>NA (Not Applicable) Should be reserved for those study design aspects that are not applicable given the study design under review (for example, allocation concealment would not be applicable for case–control studies).			
**Section 1: Population**			
**Number**	**Question**	**Response**<br><br>**++/+/-/ NR/NA**	**Comments**
1.1	Is the source population or source area well described?		

For peer review only

	Was the country (e.g. developed or non-developed, type of health care system), setting (primary schools, community centres etc), location (urban, rural), population demographics etc adequately described?		
1.2	Is the eligible population or area representative of the source population or area?<br><br>Was the recruitment of individuals, clusters or areas well defined (e.g. advertisement, birth register)?<br><br>Was the eligible population representative of the source? Were important groups underrepresented?		
1.3	Do the selected participants or areas represent the eligible population or area?<br><br>Was the method of selection of participants from the eligible population well described?<br><br>What % of selected individuals or clusters agreed to participate? Were there any sources of bias?<br><br>Were the inclusion or exclusion criteria explicit and appropriate?		
**Section 2: Method of selection of exposure (or comparison) group**			
2.1	Selection of exposure (and comparison) group. How was selection bias minimised?<br><br>How was selection bias minimised?		
2.2	Was the selection of explanatory variables based on a sound theoretical basis?<br><br>How sound was the theoretical basis for selecting the explanatory variables?		

For peer review only

2.3	Was the contamination acceptably low?<br><br>Did any in the comparison group receive the exposure?<br><br>If so, was it sufficient to cause important bias?		
2.4	How well were likely confounding factors identified and controlled?<br><br>Were there likely to be other confounding factors not considered or appropriately adjusted for?<br><br>Was this sufficient to cause important bias?		
**Section 3: Outcomes**			
3.1	Were the outcome measures and procedures reliable?<br><br>Were outcome measures subjective or objective (e.g. biochemically validated nicotine levels ++ vs self-reported smoking −)?<br><br>How reliable were outcome measures (e.g. inter- or intra-rater reliability scores)?<br><br>Was there any indication that measures had been validated (e.g. validated against a gold standard measure or assessed for content validity)?		
3.2	Were the outcome measurements complete?<br><br>Were all or most of the study participants who met the defined study outcome definitions likely to have been identified?		
3.3	Were all important outcomes assessed?		

For peer review only

	Were all benefits and harms assessed?<br><br>Was it possible to determine the overall balance of benefits and harms of intervention versus comparisons?		
3.4	Was there a similar follow-up time in exposure and comparison groups?<br><br>If groups are followed for different lengths of time, then more events are likely to occur in the group followed-up for longer distorting the comparison.<br><br>Analyses can be adjusted to allow for differences in length of follow-up (e.g. using person-years).		
3.5	Was follow-up time meaningful?<br><br>Was follow-up long enough to assess long-term benefits and harms?<br><br>Was it too long, e.g. participants lost to follow-up?		
**Section 4: Analyses**			
4.1	Was the study sufficiently powered to detect an intervention effect (if one exists)?<br><br>A power of 0.8 (i.e. it is likely to see an effect of a given size if one exists, 80% of the time) is the conventionally accepted standard.<br><br>Is a power calculation presented? If not, what is the expected effect size? Is the sample size adequate?		

For peer review only

4.2	Were multiple explanatory variables considered in the analyses?<br><br>Were there sufficient explanatory variables considered in the analysis?		
4.3	Were the analytical methods appropriate?<br><br>Were important differences in follow-up time and likely confounders adjusted for?		
4.4	Was the precision of association given or calculable? Is association meaningful?<br><br>Were confidence intervals or p values for effect estimates given or possible to calculate?<br><br>Were CIs wide or were they sufficiently precise to aid decision-making? If precision is lacking, is this because the study is under-powered?		
**Section 5: Summary**			
5.1	Are the study results internally valid (i.e. unbiased)?<br><br>How well did the study minimise sources of bias (i.e. adjusting for potential confounders)?<br><br>Were there significant flaws in the study design?		
5.2	Are the findings generalizable to the source population (i.e. externally valid)?<br><br>Are there sufficient details given about the study to determine if the findings are generalizable to the source population?		

For peer review only

	Consider: participants, interventions and comparisons, outcomes, resource and policy implications		

For peer review only

CHECKLIST D

**QUALITY ASSESSMENT FOR QUANTITATIVE STUDIES REPORTING CORRELATIONS AND ASSOCIATIONS(NICE,2012 BASED ON 'GRAPHICAL APPRAISAL TOOL FOR EPIDEMIOLOGICAL STUDIES(GATE)` BY JACKSON ET AL. (2006)**
Responses:<br><br>++ Indicates that for that particular aspect of study design, the study has been designed or conducted in such a way as to minimise the risk of bias.

+ Indicates that either the answer to the checklist question is not clear from the way the study is reported, or that the study may not have addressed all potential sources of bias for that particular aspect of study design.<br>- Should be reserved for those aspects of the study design in which significant sources of bias may persist.<br>NR (Not Reported) - Should be reserved for those aspects in which the study under review fails to report how they have (or might have) been considered.<br>NA (Not Applicable) Should be reserved for those study design aspects that are not applicable given the study design under review (for example, allocation concealment would not be applicable for case–control studies).			
**Section 1: Population**			
**Number**	**Question**	**Response**<br>**++/+/-/ NR/NA**	**Comments**
1.1	Is the source population or source area well described?<br>Was the country (e.g. developed or non-developed, type of health care system), setting (primary schools, community centres etc), location (urban, rural), population demographics etc adequately described?		
1.2	Is the eligible population or area representative of the source population or area?<br>Was the recruitment of individuals, clusters or areas well defined (e.g. advertisement, birth register)?<br>Was the eligible population representative of the source? Were important groups underrepresented?		
1.3	Do the selected participants or areas represent the eligible population or area?		

For peer review only

	Was the method of selection of participants from the eligible population well described?<br><br>What % of selected individuals or clusters agreed to participate? Were there any sources of bias?<br><br>Were the inclusion or exclusion criteria explicit and appropriate?		
**Section 2: Method of selection of exposure (or comparison) group**			
2.1	Selection of exposure (and comparison) group. How was selection bias minimised?<br><br>How was selection bias minimised?		
2.2	Was the selection of explanatory variables based on a sound theoretical basis?<br><br>How sound was the theoretical basis for selecting the explanatory variables?		
2.3	Was the contamination acceptably low?<br><br>Did any in the comparison group receive the exposure?<br><br>If so, was it sufficient to cause important bias?		
2.4	How well were likely confounding factors identified and controlled?<br><br>Were there likely to be other confounding factors not considered or appropriately adjusted for?		

For peer review only

	Was this sufficient to cause important bias?		
**Section 3: Outcomes**			
3.1	Were the outcome measures and procedures reliable?<br><br>Were outcome measures subjective or objective (e.g. biochemically validated nicotine levels ++ vs self-reported smoking −)?<br><br>How reliable were outcome measures (e.g. inter- or intra-rater reliability scores)?<br><br>Was there any indication that measures had been validated (e.g. validated against a gold standard measure or assessed for content validity)?		
3.2	Were the outcome measurements complete?<br><br>Were all or most of the study participants who met the defined study outcome definitions likely to have been identified?		
3.3	Were the outcome measurements complete?<br><br>Were all or most of the study participants who met the defined study outcome definitions likely to have been identified?		
3.4	Was there a similar follow-up time in exposure and comparison groups?		

For peer review only

	If groups are followed for different lengths of time, then more events are likely to occur in the group followed-up for longer distorting the comparison.<br><br>Analyses can be adjusted to allow for differences in length of follow-up (e.g. using person-years).		
3.5	Was follow-up time meaningful?<br><br>Was follow-up long enough to assess long-term benefits and harms?<br><br>Was it too long, e.g. participants lost to follow-up?		
**Section 4: Analyses**			
4.1	Was the study sufficiently powered to detect an intervention effect (if one exists)?<br><br>A power of 0.8 (i.e. it is likely to see an effect of a given size if one exists, 80% of the time) is the conventionally accepted standard.<br><br>Is a power calculation presented? If not, what is the expected effect size? Is the sample size adequate?		
4.2	Were multiple explanatory variables considered in the analyses?<br><br>Were there sufficient explanatory variables considered in the analysis?		
4.3	Were the analytical methods appropriate?<br><br>Were important differences in follow-up time and likely confounders adjusted for?		

For peer review only

4.4	Was the precision of association given or calculable? Is association meaningful?<br><br>Were confidence intervals or p values for effect estimates given or possible to calculate?<br><br>Were CIs wide or were they sufficiently precise to aid decision-making? If precision is lacking, is this because the study is under-powered?		
**Section 5: Summary**			
5.1	Are the study results internally valid (i.e. unbiased)?<br><br>How well did the study minimise sources of bias (i.e. adjusting for potential confounders)?<br><br>Were there significant flaws in the study design?		
5.2	Are the findings generalizable to the source population (i.e. externally valid)?<br><br>Are there sufficient details given about the study to determine if the findings are generalizable to the source population?<br><br>Consider: participants, interventions and comparisons, outcomes, resource and policy implications		

For peer review only

Summary of studies by country, country category, year of publication and focus

	Study	Country*	Category**	Year of publication	Focus of the study
1	Araujo et al[36]	Brazil	UMC	2017	Association between costs related to productivity loss and modified risk factors among uses of Brazilian National Health System
2	Cheah et al [37]	Malysia	UMC	2017a	Test the hypothesis "income is positively associated with the intensity of physical activity" by using data from a developing country(Malaysia)
3	Ding et al [38]	LMIC	LMIC	2016	Estimate direct health care cost, productivity losses and disability adjusted life years attributable to physical inactivity with standardized methods for 142 countries
4	Mitsunaga et al [39]	Brazil	UMC	2018	Assess the financial impact of a regular physical activity incentive program on medical hospital cost of private health care company
5	Turi et al [40]	Brazil	UMC	2015	Walking and health care expenditure among adults users of Brazilian health care system
6	Cheah et al [41]	Malaysia	UMC	2017b	Factors associated with participation decision and amount of participation among urban dwellers in Malaysia
7	Codongo et al[42]	Brazil	UMC	2015	Association between physical inactivity in different domains and direct public health care expenditure in adults
8	Zhang & Chaban [43]	China	UMC	2012	Total economic burden of physical inactivity in China

For peer review only

9	Turi et al [44]	Brazil	UMC	2017	Identify demographic, socioeconomic , life style and clinical factors associated with direct health care expenditure in primary care among adults through Brazilian health care system
10	Silva A.S [45]	Brazil	UMC	2015	Relationship between sufficient amount of physical activity among Brazilian adolescents and economic and social indicators of cities where they live
11	Patel et al [46]	South Africa	UMC	2011	Participation in fitness related activities of an incentive based health promotion programme and hospital costs
12	Bielmen et al[47]	Brazil	UMC	2014	Physical inactivity related inpatient costs of chronic non communicable diseases
13	Chen et al[48]	China	UMC	2015	Association of socioeconomic status with intensity of different types of physical activity in Chineese adults aiming to outline and projecting socioeconomic disparities among population undergoing rapid nutrient transition
14	Thiago et al[49]	Brazil	UMC	2015	Socioeconomic and regional difference in active transport in Brazil
15	Abdi et al [50]	Iran	UMC	2013	Compare the role of cost, time and attractiveness in predicting the participation level of student of Teheran Universities during their leisure time physical activities
16	Popkin et al[51]	China	UMC	2006	Case study from review of economic costs of diet, physical inactivity and obesity related chronic diseases to provide guidance to utilize the review for economic analysis of obesity
17	Atkinson et al[52]	LMIC	LMIC	2015	Association of occupational category and their physical inactivity and the association of human development, economic development, urbanization and physical inactivity

For peer review only

18	Queiroz et al [53]	Brazil	UMC	2020	Association between quality of life, physical activity, use of medication and costs of treatment for chronic diseases in Primary Care

For peer review only

Quality appraisal of included studies based on standard check lists

1. **Summary of quality appraisal by Larg & Moss Check list for cost of illness studies**

	Ding et al 2016 [38]	Zhang & Chaaban 2013 [43]	Bielemann et al 2015 [47]	Popikin et al 2006 [51]*
Q1(a)	++	++	+	+
Q1(b)	++	+	+	+
Q1(c) i<br>(c) ii<br>(c) iii<br>(c) iv	++<br>+<br>+<br>+(counterfactual population)	+<br>++<br>+<br>NA**	+<br>+<br>+<br>NA	+<br>+<br>NR*<br>NA
Q2(a) i<br>(a)ii<br>(a)iii<br>(a)iv<br>(a)v	-<br>+<br>+<br>+<br>+	-<br>+<br>+<br>-<br>+	-<br>+<br>++<br>+<br>+	-<br>NR<br>NR<br>NR<br>+
Q2(b)i<br>(b)ii<br>(b)iii<br>(b)iv	+<br>NA<br>+<br>+	+<br>NA<br>+<br>+	+<br>NA<br>+<br>+	+<br>NA<br>+<br>-
Q2(c)	+	NR(cost from national data)	NR	NR
Q2(d)	+	NR(cost from national data)	NA	NR
Q2(e)i<br>(e)ii	+<br>++	NR<br>NR	NA<br>NA	NR<br>NR
Q3 (a)	++	++	+	+
Q3(b)	++	-	-	-
Q3 (c)	+	NR	-	NR
Q3(d)i<br>(d)ii<br>(d)iii	+<br>-<br>?	Secondary data<br>Secondary data<br>secondary data	-<br>NR	NR<br>NR<br>NR

For peer review only

			+(CI not reported)NR	
Q3(e)	++	+	+	-
Q3(f)	++	+	-	NR
Q3(g)	++	+	+	+
Q3(h)	++	+	+	+

- Popkin et al case study developed from a review adequate information not available to comment on quality of costing study
- *NR- Not reported
- **NA-Not applicable

2. Summary of quality appraisal by NICE check list for correlations and associations

	Araujo et al 2017[36]	Cheah et al 2017a [37]	Mitsunaga 2018 [39]	Turi et al 2015 [40]	Cheah et al 2017b [41]	Codongo et al 2015 [42]	Turi et al 2017 [44]	Silva A.S 2015 [45]	Patel et al 2011 [46]	Thiago et al 2016 [49]	Abdi et al 2014 [50]	Chen et al 2015 [48]	Atkinson et al 2015 [52]
Q1.1	+	+	+	+	+	++	++	++	+	+	+	+	++
Q1.2	-	+	-	+	+	+	+	+	+	+	+	+	++
Q1.3	+	NR	+	+	+	+	+	+	+	+	-	+	++
Q2.1	NA	NA	+	NA	NA	NA	NA	NA	-	NA	NA	NA	NA
Q2.2	+	+	+	+	+	++	+	+	+	+	+	+	+
Q2.3	NA	NA	+	NA	NA	NA	NA	NA	+	NA	NA	NA	NA
Q2.4	+	+	-	+	+	++	+	-	-	-	-	-	+
Q2.5	This question not applicable for this review												
Q3.1	+	-	+	+	-	+	+	+	+	-	+	+	+
Q3.2	+	+	+	+	+	+	+	+	+	+	+	+	+
Q3.3	+	+	+	+	+	+	+	+	+	+	+	+	+
Q3.4	NA	NA	+	NA	NA	NA	NA	NA	+	NA	NA	NA	NA
Q3.5	NA	NA	+	NA	NA	NA	NA	NA	+	NA	NA	NA	NA
Q4.1*	+	+	-	+	+	+	+	+	+	-	+	+	+

For peer review only

Q4.2	+	+	-	+	+	++	+	-	+	-	-	+	+
Q4.3	+	+	+	+	+	++	+	+	+	-	-	+	+
Q4.4	+	+	-	+	+	++	+	+	+	-	-	+	+
Q5.1(IV)	+	+	++	++	++	++	++	++	++	++	+	++	++
Q5.2(EV)	++	++	++	++	++	++	++	++	++	+	+	++	++
Score	(13/13) (2/3),	(12/13) (2/3)	(9/13) (2/3)	(13/13) (3/3)	(12/13) (3/3)	(13/13) (3/3)	(13/13) (3/3)	(11/13) (3/3)	(11/13) (3/3)	(9/13) (3/3)	(9/13) (2/3)	(13/13) (3/3)	(13/13) (3/3)

*Most of the studies power calculations not done. However, large samples have been used

Quality score
Internal Validity >= 10/13=++ (>75%)
6-9/13=+ (50-75%)
0-5/13=- (<50%)

External Validity =3/3 (++),
= 2/3(+)
=≤1/3(-)

3. **Summary of quality appraisal by Drummond and Jefferson 1996- check list for economic evaluation studies**

	Queiroz et al 2020[53]
Study design	
Q1	+
Q2	+
Q3	+
Q4	+

	Queiroz et al 2020[53]
Q5	+
Q6	+
Q7	-
Data collection	
Q8	+
Q 9	-
Q 10	NA
Q11	+
Q12	+
Q13	+
Q14	NA
Q 15	NA
Q 16	NC
Q 17	-
Q 18	+
Q 19	-
Q 20	-
Q 21	-
Analysis and interpretation of results	
Q 22	+
Q 23	-
Q 24	-
Q 25	-
Q 26	NC
Q 27	-
Q 28	-
Q 29	-
Q 30	+
Q 31	+

For peer review only

	Queiroz et al 2020[53]
Q 32	NC
Q 33	+
Q 34	+
Q 35	+

Did the economic evaluation use a decision-analytic modelling framework?	No
Overall assessment by Drummond and Jefferson checklist	
Yes +<br>No -<br>Not clear NC<br>Not applicable NA	23 /35<br>(65.7%)

For peer review only

Reporting checklist for systematic review and meta-analysis.

Based on the PRISMA guidelines.

Instructions to authors

Complete this checklist by entering the page numbers from your manuscript where readers will find each of the items listed below.

Your article may not currently address all the items on the checklist. Please modify your text to include the missing information. If you are certain that an item does not apply, please write "n/a" and provide a short explanation.

Upload your completed checklist as an extra file when you submit to a journal.

In your methods section, say that you used the PRISMAreporting guidelines, and cite them as:

Moher D, Liberati A, Tetzlaff J, Altman DG, The PRISMA Group. Preferred Reporting Items for Systematic Reviews and Meta-Analyses: The PRISMA Statement

		Reporting Item	Page Number
**Title**			
	#1	Identify the report as a systematic review, meta-analysis, or both.	01
**Abstract**			
Structured summary	#2	Provide a structured summary including, as applicable: background; objectives; data sources; study eligibility criteria, participants, and interventions; study appraisal and synthesis methods; results; limitations; conclusions and implications of key findings; systematic review registration number	02
**Introduction**			
Rationale	#3	Describe the rationale for the review in the context of what is already known.	06/07

For peer review only

Objectives	#4	Provide an explicit statement of questions being addressed with reference to participants, interventions, comparisons, outcomes, and study design (PICOS).	07
**Methods**			
Protocol and registration	#5	Indicate if a review protocol exists, if and where it can be accessed (e.g., Web address) and, if available, provide registration information including the registration number.	03/08<br>Supplementary file 1
Eligibility criteria	#6	Specify study characteristics (e.g., PICOS, length of follow-up) and report characteristics (e.g., years considered, language, publication status) used as criteria for eligibility, giving rational	09
Information sources	#7	Describe all information sources in the search (e.g., databases with dates of coverage, contact with study authors to identify additional studies) and date last searched.	08
Search	#8	Present full electronic search strategy for at least one database, including any limits used, such that it could be repeated.	Supplementary file 2
Study selection	#9	State the process for selecting studies (i.e., for screening, for determining eligibility, for inclusion in the systematic review, and, if applicable, for inclusion in the meta-analysis).	09
Data collection process	#10	Describe the method of data extraction from reports (e.g., piloted forms, independently by two reviewers) and any processes for obtaining and confirming data from investigators.	09
Data items	#11	List and define all variables for which data were sought (e.g., PICOS, funding sources), and any assumptions and simplifications made.	10
Risk of bias in individual studies	#12	Describe methods used for assessing risk of bias in individual studies (including specification of whether this	10/11

For peer review only

		was done at the study or outcome level, or both), and how this information is to be used in any data synthesis.	
Summary measures	#13	State the principal summary measures (e.g., risk ratio, difference in means).	NA
Planned methods of analyis	#14	Describe the methods of handling data and combining results of studies, if done, including measures of consistency (e.g., I2) for each meta-analysis.	11
Risk of bias across studies	#15	Specify any assessment of risk of bias that may affect the cumulative evidence (e.g., publication bias, selective reporting within studies).	NA
Additional analyses	#16	Describe methods of additional analyses (e.g., sensitivity or subgroup analyses, meta-regression), if done, indicating which were pre-specified.	NA
**Results**			
Study selection	#17	Give numbers of studies screened, assessed for eligibility, and included in the review, with reasons for exclusions at each stage, ideally with a flow diagram.	12<br>Figure 1
Study characteristics	#18	For each study, present characteristics for which data were extracted (e.g., study size, PICOS, follow-up period) and provide the citation.	09/10
Risk of bias within studies	#19	Present data on risk of bias of each study and, if available, any outcome-level assessment (see Item 12).	14
Results of individual studies	#20	For all outcomes considered (benefits and harms), present, for each study: (a) simple summary data for each intervention group and (b) effect estimates and confidence intervals, ideally with a forest plot.	15-20
Synthesis of results	#21	Present the main results of the review. If meta-analyses are done, include for each, confidence intervals and measures of consistency.	Table 5
Risk of bias across studies	#22	Present results of any assessment of risk of bias across studies (see Item 15).	NA

For peer review only

Additional analysis	#23	Give results of additional analyses, if done (e.g., sensitivity or subgroup analyses, meta-regression [see Item 16]).	NA
**Discussion**			
Summary of Evidence	#24	Summarize the main findings, including the strength of evidence for each main outcome; consider their relevance to key groups (e.g., health care providers, users, and policy makers	21-24
Limitations	#25	Discuss limitations at study and outcome level (e.g., risk of bias), and at review level (e.g., incomplete retrieval of identified research, reporting bias).	24
Conclusions	#26	Provide a general interpretation of the results in the context of other evidence, and implications for future research.	24
**Funding**			
Funding	#27	Describe sources of funding or other support (e.g., supply of data) for the systematic review; role of funders for the systematic review.	25

None The PRISMA checklist is distributed under the terms of the Creative Commons Attribution License CC-BY. This checklist can be completed online using https://www.goodreports.org/, a tool made by the EQUATOR Network in collaboration with Penelope.ai

For peer review only

BMJ Open

The economics of physical activity in low and middle income countries: systematic review

Journal:	*BMJ Open*
Manuscript ID	bmjopen-2020-037784.R2
Article Type:	Original research
Date Submitted by the Author:	06-Oct-2020
Complete List of Authors:	Ranasinghe, Priyanga; Ministry of Health, Nutrition and Indigenous Medicine, ; Brunel Univeristy London, Clinical Sciences<br>Pokhrel, Subhash; Brunel University London, Health Economics Research Group<br>Anokye, Nana; Brunel University, Health Economics Research Group
**Primary Subject Heading**:	Health economics
Secondary Subject Heading:	Health economics, Public health, Sports and exercise medicine, Health policy
Keywords:	HEALTH ECONOMICS, PREVENTIVE MEDICINE, PUBLIC HEALTH, SPORTS MEDICINE, Health policy < HEALTH SERVICES ADMINISTRATION & MANAGEMENT

SCHOLARONE™
Manuscripts

I, the Submitting Author has the right to grant and does grant on behalf of all authors of the Work (as defined in the below author licence), an exclusive licence and/or a non-exclusive licence for contributions from authors who are: i) UK Crown employees; ii) where BMJ has agreed a CC-BY licence shall apply, and/or iii) in accordance with the terms applicable for US Federal Government officers or employees acting as part of their official duties; on a worldwide, perpetual, irrevocable, royalty-free basis to BMJ Publishing Group Ltd ("BMJ") its licensees and where the relevant Journal is co-owned by BMJ to the co-owners of the Journal, to publish the Work in this journal and any other BMJ products and to exploit all rights, as set out in our licence.

The Submitting Author accepts and understands that any supply made under these terms is made by BMJ to the Submitting Author unless you are acting as an employee on behalf of your employer or a postgraduate student of an affiliated institution which is paying any applicable article publishing charge ("APC") for Open Access articles. Where the Submitting Author wishes to make the Work available on an Open Access basis (and intends to pay the relevant APC), the terms of reuse of such Open Access shall be governed by a Creative Commons licence – details of these licences and which Creative Commons licence will apply to this Work are set out in our licence referred to above.

Other than as permitted in any relevant BMJ Author's Self Archiving Policies, I confirm this Work has not been accepted for publication elsewhere, is not being considered for publication elsewhere and does not duplicate material already published. I confirm all authors consent to publication of this Work and authorise the granting of this licence.

For peer review only

Title page

The economics of physical activity in low and middle income countries: systematic review

Priyanga Diloshini Ranasinghe [1,2], Subhash Pokhrel[1], Nana Kwame Anokye[1]

[1] Health Economics Theme, Institute of Environment, Health and Societies, Brunel University London, Uxbridge, United Kingdom

[2] Ministry of Health, Nutrition and Indigenous Medicine, Suwasiripaya, Colombo 10, Sri Lanka

Corresponding author: Priyanga Diloshini Ranasinghe

Postal address: Mary Seacole 2nd floor, Division of Health Sciences, Department of Clinical Sciences, Collage of Health and Life Sciences, Brunel University, London, United Kingdom

Email: priyangaran@yahoo.com/ Priyanga.Ranasinghe@brunel.ac.uk

Telephone number: 0094 777552794/0044 7424515136

Subhash Pokhrel Subhash.Pokhrel@brunel.ac.uk

Nana Kwame Anokye Nana.Anokye@brunel.ac.uk

Keywords: low- and middle-income countries; health economics; physical activity

Word count:5026(including abstract and summary)

For peer review only

Abstract

Background: Physical activity promotion among populations is a global health investment. However, evidence on economic aspects of physical activity, are sparse and scattered in low- and middle-income countries (LMIC).

Objective: To summarise available evidence on economics of physical activity (PA) in LMICs, identify potential target variables for policy, and report gaps in the existing economic evidence alongside research recommendations.

Data sources: A systematic review of the electronic databases (Scopus, Web of Science and SPORTDiscus) and grey literature.

Study eligibility-criteria: Cost of illness studies, economic evaluations, interventions and descriptive studies on economic factors associated with PA using pre-set eligibility criteria

Study appraisal and synthesis of methods: Screening, study selection, quality appraisal based on standard checklists performed by two reviewers with consensus of a third reviewer. Descriptive synthesis of data performed.

Results: Majority of the studies were from upper-middle-income countries (n=16,88.8%), and mainly from Brazil (n=9,50%). Only one economic evaluation study was found. The focus of the reviewed literature spanned the economic burden of physical inactivity (n=4,22%), relationship between PA and costs (n=6,46%), and socioeconomic determinants of PA (n=7,39%). The findings showed a considerable economic burden due to insufficient PA, with LMIC accounting for 75% DALYs globally due to insufficient PA. Socioeconomic correlates of PA were identified and inverse relationship of PA with cost of chronic diseases was established. Regular PA along with drug treatment as a treatment scheme for chronic

For peer review only

diseases, showed advantageous with a cost utility ratio of $3.21 /QALY compared to drug treatment only group ($3.92 /QALY) by the only economic evaluation conducted in LMIC; Brazil.

Limitations: Meta-analysis not performed due to heterogeneity of the studies.

Conclusions & recommendations: Economic evaluation studies for physical activity promotion interventions/strategies and local research from low income countries are grossly inadequate. Setting economic research agenda in LMIC ought to be prioritized in those areas.

PROSPERO registration number: CRD42018099856

For peer review only

For peer review only - http://bmjopen.bmj.com/site/about/guidelines.xhtml

Strengths and limitations of this study

- This is the first synthesis of literature on all types of economic analysis on physical activity in LMICs.
- The study uses a comprehensive search strategy that covers literature from academic databases and websites of international organisations such as World Health Organisation
- This review focuses on studies written in the English language and could miss out on relevant literature published in other languages.
- Meta-analysis was not possible due to wide variation in focus and methods of included studies

eview only

For peer review only - http://bmjopen.bmj.com/site/about/guidelines.xhtml

Introduction

Regular physical activity is proven to have multiple health benefits including preventing and treating non communicable diseases (NCD), as well as improving mental health and quality of life [1]. Physical activity (PA) is defined by World Health Organization (WHO) as "any bodily movement produced by skeletal muscles that requires energy expenditure – including activities undertaken while working, playing, carrying out household chores, travelling, and engaging in recreational pursuits" [2]. WHO recommends at least, 150 min moderate intensity PA or 75min vigorous intensity PA throughout the week for adults/elderly and 60 min moderate to vigorous intensity PA daily for 5-17-year-old children [2]. Insufficient physical activity (IPA) or physical inactivity is defined as absence or insufficient level of physical activity required to meet the current physical activity recommendations [1].

Of all lifestyle behaviours, physical activity has one of the largest positive impact on the risk of all-cause mortality (33% reduction) [3]. Insufficient physical activity accounts for 3.2 million deaths annually (including 9% premature deaths) and in 2010, 69.3 million DALYs were attributed to physical inactivity in worldwide [4]. Non communicable diseases are a major global health challenge and insufficient physical activity is estimated to cause a large share of the burden of main NCDs; 6% (3.2% to 7.8%) of chronic heart diseases burden, 7% (3.9% to 9.6%) of type II diabetes, 10%, of breast cancer(5.6% to 14.1%) and 10% (5.7% to 13.8%) of colon cancer burden [5]. Mortality attributed to NCDs is projected to increase from 38 million in 2012 to 52 million by 2030[3]. WHO reports that NCD s are disproportionately rising in LMIC and in 2016 over three quarter of NCD deaths (31.5 million) occurred in LMIC of which about 46% of deaths occurred before the age of 70 in these countries [6].

For peer review only - http://bmjopen.bmj.com/site/about/guidelines.xhtml

The prevalence of insufficient PA among adults in 2016 was 27.5% (25·0–32.2%) globally, 16·2% (14·2–17·9%) in low income countries and 26·0% (22·6–31·8) in middle income countries [7].The rate was even higher among school-going adolescents in low income countries (84·9%) and middle income countries (81%) [8]. Prevalence of insufficient PA has been stagnant over last 15 years in these countries [7,8].To achieve the WHO target of 15% relative reduction in the prevalence of insufficient PA by 2030 in these countries requires urgent, physical activity promotion. To date, progress of implementation has been plagued by lack of awareness and investment [1]. This is partially attributable to the paucity of evidence base on the economic gains accruable from promoting physical activity in LMIC settings [9,10]. There is an unmet demand for an evidence base on economics of physical activity in LMIC which can be used as an advocacy tool to prioritize physical activity promotion. This is critical especially in LMIC, given the unlimited competing health needs and developing economies. Whilst there few economic studies on physical activity, the evidence base is scattered. Systematic review is a high-quality source to inform formulation of policies and setting of budget priorities and build a coherent research base [9], but such studies are lacking in economics of physical activity. A few exceptions include a comprehensive review of studies estimating economic burden of physical inactivity worldwide [11]. This current study has a broader scope in terms of the economic evidence (all types of economic analyses) but with limited geographic settings. In line with the standard definition of economics [12], we categorise economic research on physical activity in to; examining socio-economic determinants of physical activity, impacts of physical (in) activity on economic outcomes such as costs, quality of life, labour market outcomes and economic evaluation studies on physical activity interventions [9,10]. This broad perspective is to facilitate a broader mapping of the multiple facets of the evidence base in LMIC. A specific review for LMIC is required considering

For peer review only - http://bmjopen.bmj.com/site/about/guidelines.xhtml

the differing dynamics of the health systems [13] and the urgent need to better understand the research base and map gaps in knowledge given the growing burden of NCDs [2]. Health research in low- and middle-income countries (LMICs) is warranted to overcome global health challenges [14].

Thus, the aim of this review was to (i) Summarize available evidence on economics of physical activity in LMIC (ii) Describe the focus and methods underpinning research on economics of physical activity in LMIC (iii) Identify potential target variables and cost effective interventions in LMIC for policy formation with regard to physical activity promotion (iv) Identify and report gaps in research on economics of physical activity in LMIC and provide recommendations for economic research agenda in LMIC.

Methods

Study protocol with detailed methods for this review was published prior to the commencement of this review [15] and shown in supplementary file 1.

Search strategy

Systematic literature search was conducted to identify studies on economics of physical activity in LMIC using the following data bases; Scopus (covers 100% of MEDLINE coverage, 100% of EMBASE coverage, 100% of Compendex coverage [16]), Web of Science and SPORTDiscus. Websites of World Health Organization (WHO), National Institute for Health and Care Excellence (NICE) international, World Bank and reference lists of included studies were searched for any relevant studies. The search period covered beginning of records to December 2017 in line with study protocol. To identify any publications after the search end

For peer review only - http://bmjopen.bmj.com/site/about/guidelines.xhtml

For peer review only

date (2017), a more recent search was conducted in August 2020 using SCOPUS, the biggest bibliographic database. The search strategy was informed by a scoping review that covered relevant reviews on economics studies on physical activity [17-23], reviews on physical activity [24-26] and reviews on economic evaluations [27-29]. Final search strategy including search terms for each data base is given in supplementary file 2.

Eligibility criteria and screening

All results were screened by titles to remove irrelevant studies and duplications independently by two reviewers (PDR, NKA). Disagreements were discussed with a third reviewer (SP). Remaining studies were further screened by titles and abstracts prior to full text screening. Studies were included if the following criteria were met; (i) Study setting: Any setting of LMIC in accordance with the definition of LMIC by World Bank classification 2017[13] (ii) Population: any age group across life course (iii) Interventions: any physical activity intervention (iv) Comparator: normal routine, no intervention (intervention and comparator applicable only for intervention studies) (v) Outcomes: (a) cost effectiveness ratio(CE ratio), QALY, incremental cost effectiveness ratio (ICER) assessed as the outcome of physical activity intervention, (b) cost of physical (in) activity in terms of health care cost and/or productivity loss and/or total cost of physical inactivity (vi) Study design: Observational studies (cohort, case control, cross sectional), experimental studies including randomized control trials(RCT),quasi experimental studies including natural experiments and economic evaluation studies (vii) Studies reported in English language

For peer review only - http://bmjopen.bmj.com/site/about/guidelines.xhtml

Case reports, case series, letters to the editor, editorials, reviews, qualitative studies, unpublished thesis, conference abstracts and any unobtainable texts were excluded. Studies that did not report cost, economic evaluations, and economic correlates of physical activity, separately from other lifestyle risk factors, were excluded from the review by consensus of all three reviewers. List of excluded studies with reasons given in supplementary file 3.

Data extraction

Data on following main items; general information, characteristics of the study, characteristics of population/intervention/comparator/outcome/study design(PICOS), data sources, analysis, results, conclusions, and limitations were extracted using a word template. Data extraction form was adapted from data extraction forms from relevant reviews [21, 30,31]. The full data extraction form is shown in supplementary 4.

Risk of bias and quality appraisal

Larg and Moss checklist [32], check list by NICE [33] were used to assess risk of bias of cost of illness studies and studies on correlations or associations respectively. Drummond [34] and Phillips check lists [35] were used for economic evaluations studies. The latter more specifically for model based economic evaluations.

Overall quality grading was given for both internal validity and external validity using NICE recommendations [33]. The internal validity of studies on correlations was assessed based on section 2-4 and external validity section 1 of NICE check list [33]. For cost of illness studies,

For peer review only - http://bmjopen.bmj.com/site/about/guidelines.xhtml

For peer review only

external validity was assessed by Q2b (III) and internal validity by remaining questions in Larg and Moss checklist [32].

Studies meeting all or most (>75%) were scored '++', some criteria met (50%-75%) had '+' and few (<50%) had '-'. The cut-offs were set with full agreement of all three reviewers prior to commencement of the study.

Data from included studies were extracted by one reviewer (PDR). A second reviewer (NKA) independently extracted data from 50% (9/18) of the included papers, randomly selected. Disagreements were discussed with a third reviewer (SP). Same pattern was repeated for quality appraisal.

Data synthesis

Descriptive synthesis of data was performed in order to describe the methods, operationalization of methods, quality, major limitations, outcome, suggestions and recommendations for future research. Data from research evidence on economics of physical activity in LMIC was used to prepare summary tables. Identification of potential target variables for policy and the gaps in research on economic of physical activity was gleaned from descriptive analysis of available evidence.

Patient and Public Involvement

The review was based on already available published research. Patients or public were not directly involved in designing and conduct of the study. The results of the review will be disseminated through this scientific publication, conference presentations, and social media networks available in public domain.

For peer review only - http://bmjopen.bmj.com/site/about/guidelines.xhtml

Results

Search outcomes

The systematic literature search (in 2018) retrieved 16 724 records from the initial data base search, 11 records from other sources and 2962 records from updated search (2020) search. After removing duplicates and irrelevant records by screening of titles, 97 articles were selected for abstract screening. Thirty-four records were assessed for eligibility at full text screening. Eighteen articles were selected for data extraction [36-53]. Flow diagram of this process is illustrated in figure 1.

Overview of studies

The 18 studies originated from Brazil (n=9, 50.0%), China (n=3, 16.7%), Malaysia (n=2, 11.1%), South Africa and Iran (n=1, 5.6% from each). There were two global analysis (n=2, 11.1%) (Figure 2). Majority of studies were conducted in upper middle-income countries (n=16,88.8%) [36-37, 39-51, 53]. All studies were published after 2012. Supplementary file 5, provides an overview of the aim of the study. Four studies focused on economic burden of physical inactivity [38,43,47,51] (n=4, 22.2%), six studies assessed association of physical (in)activity and direct or indirect cost [36,39,40,42,44,46] (n=6,33.3%) and seven studies examined the association of physical (in)activity and economic factors [37,41,45,48,49,50,51] (n=7, 38.8%). Only one economic evaluation study was identified (n=1, 5.6%) [53].

For peer review only - http://bmjopen.bmj.com/site/about/guidelines.xhtml

Methods of reviewed studies

The summary of studies by methods is given in table 1. Majority of the studies have used cross sectional design (n=14,77.8%) (36-44,47-49,51,52).

All four studies on economic burden of physical activity, used secondary data to estimate the cost of inactivity while prevalence data on inactivity was obtained from population surveys or GBD study.

DALYs lost due to physical inactivity in LMIC, was estimated by one study as a part of a global analysis [38]. Similar methodology was applied in the other three studies using population attributable fraction (based on prevalence of inactivity, relative risk values (RR), cost of main NCDs related to physical inactivity) [38, 43, 51]. Estimates for RR were obtained from developed countries due to unavailability of data for LMIC. One study has assessed only the physical inactivity related inpatient costs of chronic non communicable diseases [47].

Majority of studies examined the association of physical (in)activity and economic factors. The economic variables identified were direct health cost [39,40,42,44,46], productivity lost [36], and socioeconomic indicators [37, 41,45,48,50,52]. The method of analyses was regression based particularly for the direct health care cost and productivity loss analyses [36,40,42,44,46]., and bivariate for the rest [37,41,45,48,49,50,52]. A summary of included studies by PICOS, is shown in table 2.

Table 3 shows that the method of operationalization of physical activity varied across the studies. Only two studies used the WHO recommended definition of sufficient physical activity; 150min of physical activity per week [38,39]. One study used more strict definition of sufficient physical activity; 300min during last 7 days [45]. All physical activity data were

For peer review only

based on self-reports with the Baecke questionnaire identified as the most frequently used survey instrument [36,40,42,44,53].

Cost was measured either as direct health care cost or indirect cost. Direct health care cost was specified in practice as cost for medications, laboratory tests and medical consultations [39,40,42,44] and/or inpatient cost [39,46] and/or outpatient cost, prescription cost [38](Table 3). Indirect cost was operationalized as loss of productivity due to premature mortality [38], absenteeism and disability retirement [36].

Quality appraisal of included studies

Table 4 summarises quality appraisal of included studies and supplementary file 6 details the results of quality appraisal by Larg and Moss check list [38,43,47,51], NICE check list for studies on correlations and association [36,37,39-42,44-46,48-50,52], and Drummond check list for the economic evaluation study[53].

Majority of studies fulfilled most of the criteria (n=11,61.1%) [36, 37, 38, 40, 41, 42, 44, 45, 47, 48,52], while five studies fulfilled some of the criteria (6/18, 33.3%) [49, 50, 43,47,53] for internal validity. Insufficient information on methods precluded quality appraisal for one study [51]. Similarly, external validity was good in all studies.

Empirical results from reviewed studies

The empirical evidence from the reviewed papers are summarised below in the account of the main aims of the papers (Table 5).

For peer review only - http://bmjopen.bmj.com/site/about/guidelines.xhtml

For peer review

Economic burden of physical inactivity in LMIC

Direct cost

The direct health care cost in LMIC attributable to physical inactivity was found to be 10.3 billion INT$ in 2013 which is 19.1% of direct health care cost attributable to physical inactivity worldwide. Upper middle-income countries contributed to 86% of this economic burden [38]. Estimated direct health cost in China attributed to physical inactivity in 2007 was 3.5 billion USD [43] while cost of hospital admissions in Brazil in 2013 was estimated as 0.73 billion USD by local studies [47]. Estimated direct health care cost attributed to physical inactivity was 3.1 billion INT$ for China and 1.6 billion INT$ for Brazil in 2013 by global analysis [38]. Inpatient cost and outpatient cost attributed to physical inactivity in 2000 was 0.4 billion USD and 0.9 billion USD in China [51]. Cost of chronic diseases attributed to physical inactivity in China accounted for 15.2% of total cost of main NCDs in 2007 while 15% of inpatient costs from Brazilian Health Care System in 2013 was related to insufficient PA.

Indirect cost and DALYs

Physical inactivity related deaths in LMIC settings contributed to productivity loss of 5.3 billion INT$ covering 38.7% of productivity loss due to deaths related to physical inactivity worldwide [38]. LMIC bear 10.1 million DALYs attributed to physical inactivity which is 75% DALYs worldwide [38]. Indirect cost of physical inactivity in China was estimated as 3.3 billion USD in 2007 [43]. Estimated indirect cost due to productivity loss by deaths related to physical inactivity in China was reported as 1.78 billion INT$.

For peer review only - http://bmjopen.bmj.com/site/about/guidelines.xhtml

Association of physical (in) activity and health care cost in LMIC

Walking (always) during leisure time was showed to be associated with lower health care expenditure in primary care (OR_{adj} 0.59, 0.39-0.89) [40]. Higher health care expenditure for medicine was associated with lower participation in physical activity at work (OR 1.58 (1.06-1.35) and sport (OR1.57 (1.12-2.18) in Brazil [42]. Further, expenditure related to medicine (r= 0.109, 95%CI 0.046- 0.171) and overall expenditure (r= 0.092, 95%CI 0.046-0.171) was significantly associated with physical inactivity in Brazil [42].In addition, a significant association of expenditure on medication was with moderate physical activity showed by another Brazilin study (OR 0.56, 0.38-0.81) [44]. A retrospective longitudinal study in South Africa showed that the odds of hospital admissions were 13% lower for two additional gym visits per week among participants of incentive-based fitness related activity programme [46]. Members who were not on a health promotion programme had a significantly higher probability of hospital admissions (p<0.01) and higher claims than participants who changed their physical activity status to "active to no change" group or "active to more active group" in South Africa [46]. However, a Brazilian study showed inconclusive results for association of adherence to an incentive based physical activity program and direct health care cost among employees of a private health care company [39].

Physical inactivity was found to be correlated with productivity losses. A Brazilian study showed that physical activity was associated with productivity loss due to absenteeism OR_{adj} 0.29(0.09-0.88) [36].

For peer review only - http://bmjopen.bmj.com/site/about/guidelines.xhtml

Economic factors associated with physical (in) activity in LMIC

A number of economic factors were showed to be potential target variables for physical activity promotion policies.

Socio economic status

Two Malaysian studies revealed that individuals with higher levels of income, are less likely to participate in physical activity [37,41]. This was corroborated by a Brazilian study using data from Brazil`s National household sample survey 2008 to analyse income quintiles by active commuting. The study revealed that in urban population lowest income quintile had higher active transport (walking and cycling) level than the wealthiest population (2-5 times higher) [49] ($p<0.001$). Similarly, in China income levels were associated with lower physical activity [48].

Cross sectional study carried out in China reported that people from upper socioeconomic status (≥ 12 index scores – score was based on educational attainment, occupation and income per capita) use active transport more compared to lower social classes ($p <0.0001$) [48]. However, this study further, revealed that people in upper socioeconomic classes had less overall intensity of physical activity in MET hours per week (114.4, 95%CI 106.6-122.5) compared to lower class (140.3, 95%CI 132.3-148.5), lower middle class (167.4, 95%CI 163.8-171), upper middle class (141.1. 95%CI 135.6-146.6) [48].

Employment

Cross sectional study conducted in a national representative sample in Malaysia revealed that employed individuals were more likely to participate in physical activity compared to unemployed individuals (LR 0.035, $p<0.05$) [41]. A global analysis of occupational structure

For peer review only - http://bmjopen.bmj.com/site/about/guidelines.xhtml

and physical inactivity reported that individuals working in white-collar industry compared to agriculture were more likely to be physically inactive (OR=1.84, CI 1.73-1.95) [52].

Health Insurance

In Malaysia, individuals who have insurance coverage were found to be 3.2% more likely to participate in physical activity, but do not spend more time than their counterparts who are not covered by insurance [41].

Attractiveness of leisure time physical activity/cost for leisure time physical activity

Attractiveness of recreational leisure time physical activity (LTPA) was more likely to predict the participation in LTPA among students of Tehran Universities (OR 1.073), but time and money costs were not significant predictors [50]. Attractiveness of LTA was specified as how participation in LTPA could promote diversity, excitement and competiveness.

M**acroeconomic indicators**

In Brazil, Gini index accounted for 12% variance in Physical activity (adjusted R^2=0.12), if Gini index of the city increase by 1%, prevalence of sufficient physical activity would decrease by 41.5% for girls and 60.2% for boys [45]. Global analysis among forty-seven LMIC, reported that increase of Human Development Index (HDI) value is associated with decreased levels of insufficient physical activity (OR=0.98, 95%CI 0.97-0.99) [52].

For peer review only - http://bmjopen.bmj.com/site/about/guidelines.xhtml

Cost effectiveness of physical activity interventions

Recent study conducted in Brazil revealed the value for money of combining physical activity and drug medication for the treatment of chronic diseases as the treatment scheme for >50year old attendees of selected primary health care settings [53]. Higher cost utility ratio ($ 3.92 / QALY) was reported among individuals on "medications only" compared to those on "medication and regular physical activity" ($ 3.21 / QALY) and "no medication or physical activity" groups ($ 0.12 / QALY).

Research gaps

Box 1 summarises the gaps in the evidence base. Majority of the studies either assessed the socioeconomic factors associated with physical activity or the economic burden of physical inactivity. Limited evidence on economic evaluation in LMIC.

BOX1

Gaps in evidence on economics of physical activity in LMIC

- Economic evaluations of interventions and practices for physical activity promotion is minimal
- A standard method to quantify economic burden of physical inactivity at country level is lacking
- No standard method of operationalization of physical inactivity to enable transferability of evidence
- Limited evidence available on economics of PA in LMIC compared to HIC
- No evidence from local led research from low income countries.

For peer review only - http://bmjopen.bmj.com/site/about/guidelines.xhtml

Discussion

Economic burden of insufficient physical activity is consistently highlighted as problem of HIC while larger proportion of disease burden is a problem of LMIC [38]. Thus, the economic burden of physical inactivity in LMIC could be predicted to be escalated in near future. However, considering LMIC as separate entity, even the current estimates of economic burden is huge in comparison to the income level of these countries.

Economic burden of insufficient PA [43,47] and the inverse association of PA with economic cost of chronic diseases [36,40,42,44,46], revealed by locally led research in this review. This is an important evidence to draw attention of authorities and policy makers on PA promotion as a priority measure in LMIC. Global analysis on economic burden of insufficient PA including LMIC, further enhanced this evidence [38]. However, locally led research were mostly contributed by Brazilian studies. The situation could be the same in other LMIC, though the evidence is limited from other countries. It is well evident that investing on physical activity promotion will pave the path towards achieving the sustainable development goals 2030 in LMIC [1].

The cost estimates of studies from China and Brazil cannot be directly compared with the results from global analysis due to methodological differences and difference in year of cost estimate of these studies. There is no evidence on economic burden of physical inactivity from LIC by local studies. Thus, capacity building of researchers of LMIC should be done to apply more consistent, robust methodology of cost estimation and to obtain better epidemiological and economic data for cost estimation, particularly in LMIC [38].

For peer review only - http://bmjopen.bmj.com/site/about/guidelines.xhtml

Majority of research from economic evidence base in LMIC were focused on socioeconomic factors associated with participation in PA. These are also important to identify prioritize population groups for physical activity promotion. Upper socioeconomic class, people in higher income category, unemployed individuals and white-collar job employees are at higher risk of less physical activity participation, low intensity and shorter duration of PA. Hence, these groups need to be prioritized in physical activity promotion in LMIC.

This review has shown that macroeconomic indicators of the country are significantly associated with physical activity of the population. Physical activity level increases with the increase of HDI [52] and decreases with Gini index, which is a measure of inequality of income and wealth distribution of populations [45]. Though income level alone has shown a negative association with level of PA [37, 41, 48, 49] in LMIC, socioeconomic status of population subgroups as a whole have shown a positive association with the quality and quantity of physical activity in high income countries [54]. This would have contributed to the positive association of level of physical activity and HDI of the countries [45, 52]. Based on one study we are unable to generalize the usefulness of health insurance schemes and attractiveness of PA options in physical activity promotion. However, more studies need to be conducted to conclude on effect of health insurance on PA and effect of attractiveness of PA intervention compared to time and cost variables.

It is of paramount importance to understand cost effective interventions and strategies for physical activity promotion in LMIC. There was only one recent research on cost utility analysis. This initiation to conduct economic evaluation studies in LMIC is an achievement. Prescribing "PA along with medication" or "PA alone" as a treatment regime for chronic diseases, which was proved to be advantageous through cost utility analysis, should be

For peer review only - http://bmjopen.bmj.com/site/about/guidelines.xhtml

considered to implement in LMIC. This is with great value for treatment policy for NCD in LMIC. Positive association of regular walking with less medical expenditure, further supported above evidence [40]. Health impact of exercise prescription was also well documented by studies from HIC [55].

Economic evidence base on PA is biased towards MIC, especially towards Brazil, as most of these researches were conducted in Brazil. Another global review on economic burden of physical inactivity related to cardiovascular diseases also noted scarcity of research from LMIC other than few Brazilian studies [56]. Locally led research from low income countries not available. Same study population of >50-year-old adults seeking treatment from primary health care settings were studied in majority of Brazilian studies. Adolescents as a study population need to be prioritized in the future research agenda on economic aspects of PA. This review showed that economics of physical activity is a grossly neglected area in the research agenda of LMIC. Furthermore, locally led research in LMIC are much needed to overcome global health challenges [57]. Thus, more local research needs to be encouraged in this field in both middle income and low-income countries as a global priority to combat the global pandemic of physical inactivity. It is of paramount importance that researchers should explore and identify cost effective interventions to improve physical activity within the context of LMIC. Health research capacity of LMIC is insufficient and developing their capacity is not prioritized globally [56]. Overview of economic evaluations in LMIC reports the challenge of conducting economic evaluations in this context due to absence of routine cost accounting system and limited patient information systems [58]. Further, they reveal the limited capacity and funding to conduct health economic research in most LMIC [58]. An important suggestion that the findings of present review agree with the overview of economic

For peer review only

evaluations in LMIC [58] is to encourage economic evaluation studies in LMIC by advocating funders to request economic evaluations to be included for trails and other evaluations to ensure funding for those researches.

Despite the limited number of studies in LMIC on economics of PA, they reported a good methodological quality. Though, sensitivity analysis not performed in the study on cost utility analysis, it also reported a considerable quality, based on Drummond check list criteria.

The policy makers and authorities need to be advocated on the consistent evidence from LMIC on increased health care cost and productivity loss due to physical inactivity [34, 37,38, 40, 42,44], possibility of escalating the economic burden related to physical inactivity in near future [36] and the need include physical activity promotion across all policies. Further, prioritization and investment for research on economics of PA in LMIC need to be a global priority.

Limitations: Meta-analysis of data was not possible in this review due to wide variation in focus and methods of the included studies for the review. All locally led research were from MIC. Though we interpret the results for LMIC, there is a considerable socio-economic disparity between LIC and MIC.

Conclusions and Recommendations: Considerable cost of chronic diseases and hospital admissions are connected to physical inactivity in LMIC. High income, high socio-economic level, unemployment status and white-collar jobs associated with low PA levels. PA should be prescribed alone or along with medication to reduce the cost per QALY among patients with chronic disease. Research on economics of PA is grossly inadequate in LMIC. It should be a

For peer review only - http://bmjopen.bmj.com/site/about/guidelines.xhtml

For peer review only

priority research area in LMIC. Especially the research on economic evaluations related to physical activity should be considered as a priority in research agenda in LMIC.

Contributors: SP and NKA developed the idea for the review with inputs from PDR. PDR and NKA conducted the review with the guidance of SP. PDR wrote the first draft. SP and NKA revised the manuscript. NKA will act as guarantor of the review.

Conflicts of interest: None

Funding: This research received no specific grant from any funding agency in the public, commercial or not-for-profit sectors

Data sharing statement: All data relevant to the study are included in the article and uploaded as supplementary files. If any further information is required, will be provided on request to authors by email.

Completed reporting checklist for systematic review and meta-analysis based on the PRISMA guidelines given in supplementary file 7.

Figure 1: Flow diagram of article selection process

Figure 2: Number of articles on economics of physical activity by country

List of references

1. World Health Organization. Action plan on physical activity 2018–2030: more active people for a healthier world. Geneva: World Health Organization; 2018. Licence: CC BY-NC-SA 3.0 IGO.

For peer review only - http://bmjopen.bmj.com/site/about/guidelines.xhtml

2. World Health Organization.2018, *Detail-Physical activity*, viewed on 21.05.2018, http://www.who.int/en/news-room/fact-sheets/detail/physical-activity

3. Centres for Disease Control and Prevention. 2020, Benefits of Physical Activity, Accessed on [23.07.2020] https://www.cdc.gov/ physicalactivity/basics/pa-health/index.htm

4. World Health Organization. Global Status Report on Non communicable Diseases 2014. Geneva: World Health Organization; 2014. http://apps.who.int/iris/bitstream/10665/148114/1/9789241564854_eng.pdf?ua=1

5. Lee, I. M., Shiroma, E. J., Lobelo, F., Puska, P., Blair, S. N., Katzmarzyk, P. T., & Lancet Physical Activity Series Working Group (2012). Effect of physical inactivity on major non-communicable diseases worldwide: an analysis of burden of disease and life expectancy. *Lancet (London, England)*, *380*(9838), 219–229. https://doi.org/10.1016/S0140-6736(12)61031-9

6. World Health Organization. 2016, Global Health Observatory (GHO) data, NCD mortality and morbidity, Accessed on 30.07.2020 https://www.who.int/gho/ncd/mortality_morbidity/ncd_four/en/

7. Guthold, R., Stevens, G.A., Riley, L.M., Bull, F.C., (2018). Worldwide trends in insufficient physical activity from 2001 to 2016: a pooled analysis of 358 population-based surveys with 1·9 million. *Lancet Glob Health,* 6: e1077–86 http://dx.doi.org/10.1016/ S2214-109X(18)30357-7

8. Guthold, R., Stevens, G.A., Riley, L.M., Bull, F.C., (2020). Global trends in insufficient physical activity among adolescents: a pooled analysis of 298 population-based surveys with 1·6 million participants, *Lancet Child Adolesc Health*, 4: 23–35 https://doi.org/10.1016/ S2352-4642(19)30323-2

9. National Coalition for Promoting Physical Activity. 2005. Economics of physical activity promotion United States. Accessed on 25.07.2020

For peer review only - http://bmjopen.bmj.com/site/about/guidelines.xhtml

http://www.ncppa.org/sites/default/files/Economics%20of%20Physical%20Activity%20Final.pdf

10. Sturm R. (2005). Economics and physical activity: a research agenda. *Am J Prev Med*, 28:141–9. 13.

11. Ding D, Kolbe-Alexander T, Nguyen B, et al. (2017). The economic burden of physical inactivity: a systematic review and critical appraisal. *British Journal of Sports Medicine*; 51:1392-1409.

12. Backhouse, R.E., (2019). Founder of Modern Economics: Paul A. Samuelson: Volume 1: Becoming Samuelson, 1915-1948 (Oxford Studies in History of Economics), 1st Edition, Oxford University Press 2019, ISBN: 0190664118

13. Bank W. World bank country and leading groups: country classification. 2017, https://datahelpdesk.worldbank.org/ knowledgebase/articles/906519-world-bank-country-and-lendinggroups (Accessed on 1st Dec 2017)

14. Dye, C., Boerma, T., Evans, D., et al. The World Health Report 2013: research for universal health coverage. Geneva: World Health Organisation, 2013.

15. Ranasinghe, P.D., Pokhrel, S., Anokye, N.K. (2019) The economics of physical activity in low-income and middle-income countries: protocol for a systematic review. *BMJ Open*, 9:e022686. doi: 10.1136/bmjopen-2018-022686

16. Burnham, J. F. (2006). Scopus database: a review. *Biomed Digit Libr,* 3(1), 1742-5581.

17. . Oldridge, N. B. (2008). Economic burden of physical inactivity: healthcare costs associated with cardiovascular disease. *European Journal of Cardiovascular Prevention & Rehabilitation*, 15(2), 130-139. doi: 10.1097/HJR.0b013e3282f19d42 15.

18. Jeroen C. M. Barte & G. C. Wanda Wendel-Vos (2017) A Systematic Review of Financial Incentives for Physical Activity: The Effects on Physical Activity and Related Outcomes, *Behavioral Medicine*, 43:2, 79-90, DOI: 10.1080/08964289.2015.1074880 16.

For peer review only - http://bmjopen.bmj.com/site/about/guidelines.xhtml

19. Abu-Omar, K., Rütten, A., Burlacu, I., Schätzlein, V., Messing, S., & Suhrcke, M. (2017). The cost-effectiveness of physical activity interventions: A systematic review of reviews. *Preventive Medicine Reports*, 8, 72–78. http://doi.org/10.1016/j.pmedr.2017.08.006 17.

20. Brown, V., Zapata Diomedi, B., Moodie, M., Veerman, J., & Carter, R. (2016). A systematic review of economic analyses of active transport interventions that include physical activity benefits. Transport Policy, 45(18)190-208.

21. Pavey, T. G., Taylor, A. H., Fox, K. R., Hillsdon, M., Anokye, N., Campbell, J. L., Taylor, R. S. (2011). Effect of exercise referral schemes in primary care on physical activity and improving health outcomes: systematic review and meta-analysis. *BMJ,* 343. doi: 10.1136/bmj.d6462 19

22. Campbell, F., Holmes, M., Everson-Hock, E., Davis, S., Woods, H. B., Anokye, N., Kaltenthaler, E. (2015). A systematic review and economic evaluation of exercise referral schemes in primary care: a short report. *Health Technology Assessment,* 19(60), 1-+. doi: 10.3310/hta19600 Page 24 of 95

23. Suhrcke, M., Boluarte, T. A., & Niessen, L. (2012). A systematic review of economic evaluations of interventions to tackle cardiovascular disease in low- and middle-income countries. [journal article]. *BMC Public Health,* 12(1), 2. doi: 10.1186/1471-2458-12-2

24. Milton, K., Macniven, R., & Bauman, A. (2014). Review of the epidemiological evidence for physical activity and health from low- and middle-income countries. [Review]. *Glob Public Health*, 9(4), 369-381.

25. Lachat, C., Otchere, S., Roberfroid, D., Abdulai, A., Seret, F. M., Milesevic, J., Kolsteren, P. (2013). Diet and physical activity for the prevention of noncommunicable diseases in low and middle-income countries: a systematic policy review. [Meta-Analysis Review]. *PLoS Med,* 10(6), 11. 23.

26. Hunter, R. F., Christian, H., Veitch, J., Astell-Burt, T., Hipp, J. A., & Schipperijn, J. (2015). The impact of interventions to promote physical activity in urban green space: a systematic

For peer review only - http://bmjopen.bmj.com/site/about/guidelines.xhtml

review and recommendations for future research. [Research Support, Non-U S Gov't Review]. *Soc Sci Med,* 124, 246-256.

27. Sutton, L., Karan, A., & Mahal, A. (2014). Evidence for cost-effectiveness of lifestyle primary preventions for cardiovascular disease in the Asia-Pacific Region: a systematic review. *Globalization and Health*, 10, 79. http://doi.org/10.1186/s12992-014-0079-3

28. Santatiwongchai, B., Chantarastapornchit, V., Wilkinson, T., Thiboonboon, K., Rattanavipapong, W., Walker, D. G., Teerawattananon, Y. (2015). Methodological Variation in Economic Evaluations Conducted in Low- and Middle-Income Countries: Information for Reference Case Development. *PLoS ONE*, 10(5), e0123853. doi: 10.1371/journal.pone.0123853

29. Nosratnejad, S., Rashidian, A., & Dror, D. M. (2016). Systematic Review of Willingness to Pay for Health Insurance in Low- and Middle-Income Countries. [Review]. *PLoS One,* 11(6).

30. Pokhrel, S., Quigley, M. A., Fox-Rushby, J., McCormick, F., Williams, A., Trueman, P., Renfrew, M. J. (2015). Potential economic impacts from improving breastfeeding rates in the UK. [Research Support, Non-U.S. Gov't]. *Arch Dis Child,* 100(4), 334-340. doi: 10.1136/archdischild-2014-306701

31. Murthy, S., John, D., Godinho, I. P., Godinho, M. A., Guddattu, V., & Nair, N. S. (2017). A protocol for a systematic review of economic evaluation studies conducted on neonatal systemic infections in South Asia. *Syst Rev,* 6(1), 252. doi: 10.1186/s13643-017-0648-7

32. Larg, A., & Moss, J. R. (2011). Cost-of-illness studies: a guide to critical evaluation. [Research Support, Non-U S Gov't]. *Pharmacoeconomics, 29*(8), 653-671.

33. National Institute for Health and Excellence (NICE), 2012. Methods for the development of NICE public health guidance (third edition), Appendix G Quality appraisal checklist-quantitative studies reporting correlations and associations. https://www.nice.org.uk/process/pmg4/chapter/appendix-g-quality-appraisal-checklist-quantitative-studies-reporting-correlations-and

For peer review only - http://bmjopen.bmj.com/site/about/guidelines.xhtml

34. Drummond, M. F., & Jefferson, T. O. (1996). Guidelines for authors and peer reviewers of economic submissions to the BMJ. *BMJ, 313*(7052), 275-283. doi: 10.1136/bmj.313.7052.275

35. Philips, Z., Ginnelly, L., Sculpher, M., Claxton, K., Golder, S., Riemsma, R., Glanville, J. (2004). Review of guidelines for good practice in decision-analytic modelling in health technology assessment. [Review]. *Health Technol Assess, 8*(36), 1-158.

36. Araujo, M. Y. C., et al. (2017). Association between Costs Related to Productivity Loss and Modified Risk Factors among Users of the Brazilian National Health System. *Journal of Occupational and Environmental Medicine*, **59**(3): 313-319.

37. Cheah, Y. K., et al. (2017). The Income and Demographic Determinants of Physical Activity Intensity: Evidence from a Developing Country. *Margin-Journal of Applied Economic Research*, **11**(3): 348-364.

38. Ding, D., et al. (2016). The economic burden of physical inactivity: a global analysis of major non-communicable diseases. *The Lancet*, **388**(10051): 1311-1324.

39. Mitsunaga, P.K., Bandini, M.,Lucca, S.R.,Moreira-Filho, D.C. (2018). Assessment of physical activity program on the medical-hospital costs of a health care company. *Rev Bras Med Trab*.2018;16(2):121-7

40. Turi, B. C., et al. (2015). Walking and health care expenditures among adult users of the Brazilian public healthcare system: Retrospective cross-sectional study. *Ciencia e Saude Coletiva*, **20**(11): 3561-3568.

41. Cheah, Y. K., et al. (2017). Factors affecting participation decision and amount of physical activity among urban dwellers in Malaysia. *Public Health*, **46**: 84-91.

42. Codogno, J. S., et al. (2015). "Physical inactivity of adults and 1-year health care expenditures in Brazil." International Journal of Public Health **60**(3): 309-316.

43. Zhang, J. and J. Chaaban (2013). The economic cost of physical inactivity in China. *Preventive Medicine*, **56**(1): 75-78.

For peer review only - http://bmjopen.bmj.com/site/about/guidelines.xhtml

44. Turi, B. C., et al. (2017). Determinants of outpatient expenditure within primary care in the Brazilian national health system. *Sao Paulo Medical Journal,* **135**(3): 205-212.

45. Silva, D. A. S. (2015). Relationship between Brazilian adolescents physical activity and social and economic indicators of the cities where they live. *Perceptual and Motor Skills,* **120**(2): 355-366.

46. Patel, D., et al. (2011). Participation in Fitness-Related Activities of an Incentive-Based Health Promotion Program and Hospital Costs: A Retrospective Longitudinal Study. *American Journal of Health Promotion*, **25**(5): 341-348.

47. Bielemann, R. M., et al. (2015). Burden of physical inactivity and hospitalization costs due to chronic diseases. *Rev Saude Publica,* **49**(89102015000100307): 20.

48. Chen, M., et al. (2015). Socioeconomic Status and Physical Activity in Chinese Adults: A Report from a Community-Based Survey in Jiaxing, China. *PLOS ONE,* **10**(7): e0132918.

49. Thiago. S., H., et al. (2016). Socioeconomic and regional differences in active transportation in Brazil. *Revista de Saúde Pública*,**50**: 37.

50. Abdi, K. et al. (2014). Analysis Role of Cost, Time and Attractiveness at leisure time physical activities in students of Tehran universities, Iran. *International Journal of Research in Management*(ISI),1(4): 16-29

51. Popkin, B. M., et al. (2006). Measuring the full economic costs of diet, physical activity and obesity-related chronic diseases. *Obes Rev***, 7**(3): 271-293.

52. Atkinson, K., Lowe, S., & Moore, S. (2016). Human development, occupational structure and physical inactivity among 47 low and middle income countries. *Preventive Medicine Reports, 3*, 40-45.

53. Queiroz,C.D., Turi, B.C., Sarti, F.M., Santos Ferro, I., Morais , L.C., Codogno, J.S.(2020). Association between quality of life, physical activity, use of medication and costs of treatment for chronic diseases in Primary Care, *The Journal of Sports Medicine and Physical Fitness*, 60(3):456-63 DOI: 10.23736/S0022-4707.19.10170-3

For peer review only - http://bmjopen.bmj.com/site/about/guidelines.xhtml

54. Earl S. Ford, Robert K. Merritt, Gregory W. Heath, Kenneth E. Powell, Richard A. Washburn, Andrea Kriska, Gwendolyn Haile, Physical Activity Behaviors in Lower and Higher Socioeconomic Status Populations, *American Journal of Epidemiology*, Volume 133, Issue 12, 15 June 1991, Pages 12461256, https://doi.org/10.1093/oxfordjournals.aje.a115836

55. Robert Sallis (2015) Exercise is medicine: a call to action for physicians to assess and prescribe exercise, *The Physician and Sports medicine,* 43:1, 22-26, DOI: 10.1080/00913847.2015.1001938

56. Oldridge, N.B., (2008). Economic burden of physical inactivity: healthcare costs associated with cardiovascular disease. *Eur J Cardiovasc Prev Rehabil.* 15(2):130-9. doi: 10.1097/HJR.0b013e3282f19d42.

57. Franzen, S.R.P., Chandler, C, Lang, T., Health research capacity development in low and middle income countries: reality or rhetoric? A systematic meta-narrative review of the qualitative literature. *BMJ Open*, 2017;7:e012332. doi:10.1136/bmjopen-2016- 012332

58. Pitt, C., Vassall, A., Teerawattananon, Y., Griffiths, U. K., Guinness, L., Walker, D., Hanson, K. (2016). Foreword: Health Economic Evaluations in Low- and Middle-income Countries: Methodological Issues and Challenges for Priority Setting. *Health economics*, 25 Suppl 1(Suppl Suppl 1), 1–5. doi:10.1002/hec.3319

For peer review only - http://bmjopen.bmj.com/site/about/guidelines.xhtml

Table 1: Summary of studies by focus and study design

	**Number (n)**	**studies**
**Focus**		
Economic burden of physical inactivity	4	Ding 2016[38] Zhang & Chaban 2012[43] Bielemann 2015[47] Popkin 2006[51]
Association of physical (in)activity and direct health cost	5	Mitsunga 2018[39] Turi 2015 [40] Codongo 2015[42] Turi 2017[44] Patel 2011[46]
Association of physical (in)activity and productivity lost	1	Araujo 2017[36]
Socioeconomic factors associated with physical activity participation and intensity	7	Cheah 2017[37] Cheah 2017[41] Silva A.S 2015[45], Chen 2015[48] Thiago 2016[49] Abdi 2014[50]Atkinson 2016[52]
Economic evaluations related to physical activity	1	Queiroz et al 2020[53]
Study design		
Cross sectional	14	Araujo 2017[36] Cheah 2017[37] Ding 2016[38] Mitsunga 2018[39]Turi 2015[40] Cheah 2017[41] Codongo 2015[42] Zhang & Chaban 2012[43] Turi 2017[44] Bielemann 2015[47] Chen 2015[48] Thiago 2016[49] Popkin 2006[51] Atkinson 2016[52]
Ecological/Correlation study	2	Silva A.S 2015[45], Abdi 2014[50]
longitudinal	1	Patel 2011[46]
Intervention studies	0	
Economic evaluation studies	1	Queiroz et al 2020[53]

For peer review only - http://bmjopen.bmj.com/site/about/guidelines.xhtml

Table 2: Summary of studies by PICOS

Author year	*Country/ Country category*	*Type of economic analysis*	*Population*	*Intervention*	*comparator*	*outcome*	*study design*
Araujo 2017[36]	Brazil- UMC	Economic correlates of PA*	≥50y adults	NA	NA	Strength of association between physical inactivity and productivity loss(OR,95%CI)	Cross sectional
Cheah 2017[37]	Malaysia-UMC	Economic correlates of PA	General population	NA	NA	Strength of association of income and physical activity (Coefficient of income)	Cross sectional
Ding 2016[38]	Global analysis-UMC/LMC/LIC	Economic burden	General population	NA	NA	Cost estimates, DALY	Cross sectional
Mitsunaga 2018[39]	Brazil-UMC	Association of PA program with medical hospital cost	Employees of a private health care company	NA	NA	Correlation of program attendance frequency with cost per capita Association of program adherence level with the average cost per capita/year	Cross sectional (study design not specified in the original article. Based on the study pattern and type of analysis we categorized as cross sectional)
Turi 2015[40]	Brazil-UMC	Association of PA and economic costs	≥50y adults	NA	NA	Association of walking and health care expenditure (OR,95% CI)	Cross sectional
Cheah 2017[41]	Malaysia-UMC	Economic correlates of PA	Urban dwellers	NA	NA	Strength of association between participation decision, amount of physical activity with income, time, employment, insurance (coefficient)	Cross sectional

For peer review only - http://bmjopen.bmj.com/site/about/guidelines.xhtml

Codongo 2015[42]	Brazil - UMC	\Association of PA with economic costs	≥50y adults	NA	NA	Strength of association between physical inactivity and health care expenditure (OR, 95%CI)	Cross sectional
Zhang & Chaban 2012[43]	China - UMC	Economic burden	General population	NA	NA	Cost estimate of physical inactivity	Cross sectional
Turi 2017[44]	Brazil- UMC	Association of PA and economic cost	≥50 years adults	NA	NA	Strength of association of physical activity and outpatient expenditure (OR, 95%CI)	Cross sectional
Silva A.S 2015[45]	Brazil- UMC	Economic correlates of PA	Year 9 students Public and private elementary school's	NA	NA	Strength of association of macroeconomic variables with physical activity(Pearson r,95% CI)	Correlation study
Patel 2011[46]	South Africa- UMC	Association of PA with economic cost	Adults who are members of discovery health medical plan	Incentive based health promotion program	Not enrolled in the program	Changes in level of participation in fitness related activities, Subsequent probability of hospital admissions and cost	Retrospective observational study. Not a intervention study
Bielemann 2015[47]	Brazil- UMC	Economic burden	Above 40 year old adults Brazil	NA	NA	Inpatient cost estimates attributed to physical activity	Cross sectional
Chen 2015[48]	China - UMC	Economic correlates of PA	30-65 year permanent residents Jiaxing China	NA	NA	Strength of association of socioeconomic level and PA	Cross sectional
Thiago 2016[49]	Brazil- UMC	Economic correlates of PA	≥14 year general population Brazil	NA	NA	Strength of association of socioeconomic status and active transport	Cross sectional
Abdi 2014 [50]	Iran- UMC	Economic correlates of PA	University students Teheran Universities	NA	NA	Strength of association of time, cost and attractiveness with LTPA	Correlational survey
Popkin 2006[51]	China- UMC	Economic burden	General population	NA	NA	Cost estimate of physical inactivity	Cross sectional(case study

For peer review only - http://bmjopen.bmj.com/site/about/guidelines.xhtml

							developed from a review)
Atkinson 2016[52]	Global analysis- UMC/LMC/LIC	Economic correlates of PA	General population	NA	NA	Strength of association (OR) of physical activity and macroeconomic variables in 47 LMIC	Cross sectional
Queiroz et al 2020[53]	Brazil- UMC	Cost utility analysis	General population	NA	NA	cost-utility and incremental cost-utility of the treatments involved in physical activity compared to drug only treatment group. QALY values and the mean cost of 12 months for each treatment scheme.	Economic evaluation study

*PA- physical activity

For peer review only

Table 3: Methods of operationalization of physical activity and cost in economic studies of physical activity

	Number	studies
**Method of operationalization of physical activity**		
By WHO definition	1	Ding 2016[38], Mitsunaga 2018[39]
Physical activity for >300 min during last 7 days	1	Silva A.S 2015[45]
Based on IPAQ	1	Atkinson 2016[52]
By Baecke questionnaire,	3	Araujo 2017[36],Turi 2015[40] Codongo,2015[42]Turi2017[44] Queiroz et al 2020[53]
Self-reported vigorous or moderate physical activity during last 7 days	2	Cheah2017[37], Cheah 2017[41]
Based on number of Gym visits per annum	1	Patel 2011[46]
Moderate or vigorous physical activity during leisure	1	Abdi 2014[50]
Performing any physical activity during leisure	1	Bielemann 2015[47]
Walking or cycling to work regardless of duration	1	Thiago 2016[49]
Not reported	1	Zhang & Chaban 2012[43]
**Operationalization of cost in economic studies of physical activity**		
**Method of operationalization of direct cost**		
cost of disease using population attributable fraction related to physical inactivity	3	Ding 2016[38] Zhang & Chaban 2012[43]
Inpatient cost	1	Bielemann 2015[47]
Total cost of consultations, diagnostic tests, hospital admissions, therapeutic procedures, emergency care paid by insurance	1	Mitsunaga 2018[39]
Total cost of Medication dispensed/laboratory tests/ medical consultations	3	Turi 2015[40], Codongo 2015[42] Turi 2017[44]
**Method of operationalization of indirect cost**		
Loss of productivity due to premature mortality	1	Ding 2016[38]
Loss of productivity due to absenteeism and disability retirement	1	Araujo 2017[36]

For peer review only - http://bmjopen.bmj.com/site/about/guidelines.xhtml

Table 4: Quality appraisal of included studies

		Number of studies	Studies
By NICE check list for quantitative studies on correlations/associations (n=13)			
Internal validity (section 2-4)	++*	10	Araujo 2017[36] Cheah2017[37] Turi 2015[40] Cheah 2017[41] Codongo,2015[42] Turi2017[44] Silva A.S 2015[45] Patel 2011[47] Chen 2015[48] Atkinson 2016[52]
	+**	3	Mitsunaga[39],Thiago 2016[49], Abdi 2014[50]
	-***	0	
External validity (section 1)	++	9	Turi 2015[40] Min J.Y. & Min K.B. 2016[39] Cheah 2017[41] Codongo,2015[42]Turi2017[44] Silva A.S 2015[45] Patel 2011[46] Chen 2015[48] Atkinson 2016[52]
	+	3	Araujo 2017[36] Cheah2017[37] Abdi 2014[50]
	-	0	
Check list by Larg & Moss for cost of illness studies (n=4****)			
Internal validity	++	1	Ding 2016[38]
	+	2	Zhang & Chaban 2012[43] Bielemann 2015[47]
	-	0	
External validity (Q2b(III))	++	0	
	+	1	Ding2016[38] Zhang & Chaban 2012[43] Bielemann 2015[47]
	-	0	
check list for economic evaluation studies by Drummond and Jefferson 1996 (n=1)			
Validity	+	1	Queiroz 2020[53]

***** (++) All or most check list criteria fulfilled (>75%) fulfilled

For peer review only - http://bmjopen.bmj.com/site/about/guidelines.xhtml

** (+) some checklist criteria (50%-75%) fulfilled

*** (-) None or few checklist criteria (<50%) fulfilled

**** [48] is a case study based on a review, thus information on methods is not adequate to report on quality

Table 5: Evidence from economic studies related to physical activity in LMIC

Evidence	studies
**Economic burden**	
**Direct cost**	
UMC 8886(16.5%), LMC 1366(2.5%), LI 75(0.1%) (INT$ 100000, global %) in 2013	Ding 2016[38]
China 3.075INT$, Brazil 1.634billion INT$ in 2013	Ding 2016[38]
China 3.5 billion USD in 2007	Zhang & Chaban 2012[43]
Brazil hospital admission cost 0.73 billion USD in 2013	Bielemann 2015[47]
Inpatient and outpatient cost of physical inactivity in China 0.4 billion USD and 0.9 billion USD in 2000	Popkin 2006[51]
**Indirect cost** (INT $1000000, global %)	
UMC 3814(27.8%), LMC 1350(9.9%), 130(0.9%)	Ding 2016[38]
China 3.3 billion USD (2007)	Zhang & Chaban 2012[43]
**Association of physical a(in)activity and economic cost**	
**Association of physical (in)activity and direct health cost**	
Health care cost significantly higher for physically inactive individuals than active individuals	Turi 2015[40] Codongo 2015[42] Turi 2017[44] Patel 2011[46]
association of adherence to a physical activity program by employees of a private health care company and direct medical costs is inconclusive	Mitsunaga 2018[39]
**Association of physical (in)activity and productivity lost**	
Significant effect from Physical inactivity on productivity loss associated health problem	Araujo 2017[36]

For peer review only - http://bmjopen.bmj.com/site/about/guidelines.xhtml

Socioeconomic factors associated with physical activity participation and intensity	
negative association of income with PA	Cheah2017[37]Cheah 2017[41] Abdi 2014[50] Thiago 2016[49] Atkinson 2016[52]
Employment status is positively associated with PA	Cheah 2017[41] Chen 2015[48]
socioeconomic disparities associated with PA	Thiago 2016[49]
Association of PA and macroeconomic indicators<br>• PA positively associated with HDI<br>• PA negatively associated with Gini index	 Atkinson 2016[52]<br>Silva A.S 2015[45]
Attractiveness of LTPA* is the predictor of participation of university students in PA over the time and cost factor of PA	Abdi 2014[50]
**Economic evaluation studies**	
The cost-utility ratio was higher among those who reported taking medications(M) compared to group with medication and regular physical activity (PA),<br>• M only- $ 3.92 / QALY<br>• M+PA -$ 3.21 / QALY<br>• No on medication OR PA only- $ 0.12 / QALY<br>The incremental utility cost advantageous for the medication + regular physical activity group	Queiroz 2020 [53]

*leisure time physical activity

For peer review only - http://bmjopen.bmj.com/site/about/guidelines.xhtml

For peer review only

For peer review only - http://bmjopen.bmj.com/site/about/guidelines.xhtml

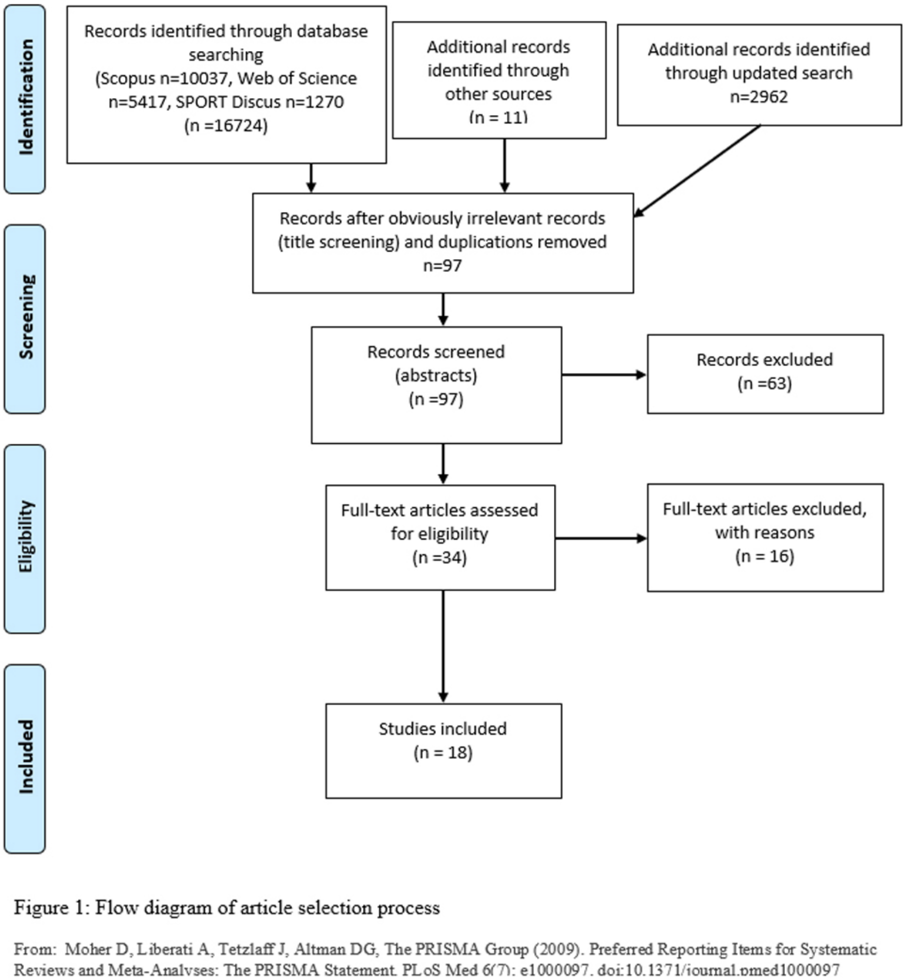

Figure 1: Flow diagram of article selection process

90x90mm (300 x 300 DPI)

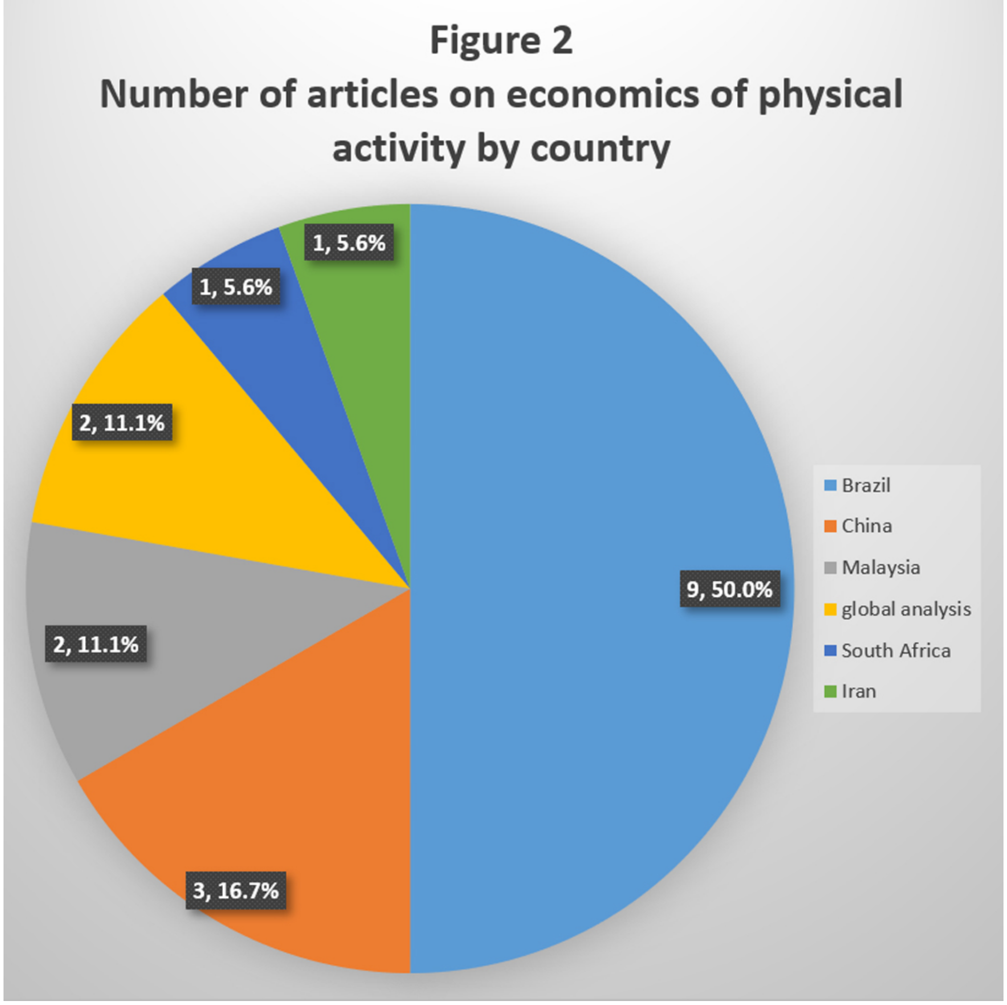

Figure 2: Number of articles on economics of physical activity by country

90x90mm (300 x 300 DPI)

Open access Protocol

BMJ Open

The economics of physical activity in low-income and middle-income countries: protocol for a systematic review

Priyanga Diloshini Ranasinghe,[1,2] Subhash Pokhrel,[3] Nana Kwame Anokye[3]

To cite: Ranasinghe PD, Pokhrel S, Anokye NK. The economics of physical activity in low-income and middle-income countries: protocol for a systematic review. *BMJ Open* 2019;**9**:e022686. doi:10.1136/bmjopen-2018-022686

► Prepublication history and additional material for this paper are available online. To view these files, please visit the journal online (http://dx.doi.org/10.1136/bmjopen-2018-022686).

Received 5 March 2018
Revised 9 October 2018
Accepted 23 November 2018

Check for updates

© Author(s) (or their employer(s)) 2019. Re-use permitted under CC BY-NC. No commercial re-use. See rights and permissions. Published by BMJ.

[1]Department of Health, Ministry of Health, Nutrition and Indigenous Medicine, Colombo, Sri Lanka
[2]Division of Health Sciences, Department of Clinical Sciences, Collage of Health and Life Sciences, Brunel University, London, UK
[3]Health Economics Theme, Institute of Environment, Health and Societies, Brunel University, London, UK

Correspondence to
Dr Priyanga Diloshini Ranasinghe;
priyangaran@yahoo.com,
Priyanga.Ranasinghe@brunel.ac.uk

ABSTRACT

Introduction Evidence on the economic costs of physical inactivity and the cost-effectiveness of physical activity interventions in low-income and middle-income countries (LMICs) is sparse, and fragmented where they are available. This is the first review aimed to summarise available evidence on economics of physical activity in LMICs, identify potential target variables for policy, and identify and report gaps in the current knowledge on economics of physical activity in LMICs.

Methods and analysis Peer-reviewed journal articles of observational, experimental, quasi-experimental and mixed-method studies on economics of physical activity in LMICs will be identified by a search of electronic databases; Scopus, Web of Science and SPORTDiscus. Websites of WHO, the National Institute for Health and Care Excellence international, World Bank and reference lists of included studies will be searched for relevant studies. The study selection process will be a two-stage approach; title and abstract screen for inclusion, followed by a review of selected full-text articles by two independent reviewers. Disagreements will be resolved by consensus and discussion with a third reviewer. Data will be extracted using standardised piloted data extraction forms. Risk of bias will be critically appraised using standard checklists based on study designs. Descriptive synthesis of data is planned. Where relevant, summaries of studies will be classified according to type of economic analysis, country or country category, population, intervention, comparator, outcome and study design. Meta-analysis will be performed where appropriate. This protocol for systematic review is prepared according to the Preferred Reporting Items for Systematic review and Meta-analysis for Protocols –2015 statement.

Ethics and dissemination Ethical approval is not obtained as original data will not be collected as part of this review. The completed review will be submitted for publication in a peer-reviewed journal and presented at conferences.

PROSPERO registration number CRD42018099856.

Strengths and limitations of this study

- This is the first synthesis of literature on economics of physical activity in low-income and middle-income countries (LMICs).
- This study provides evidence-based recommendations for economic research practice on physical activity.
- This review presents evidence on the business case for physical activity interventions for decision making by policy makers in LMIC settings.
- This review could suffer from publication bias as it excludes studies that are not peer reviewed.
- This review focuses on studies written in the English language and could miss out on relevant literature published in other languages.

INTRODUCTION

Rationale

Physical inactivity is a global health challenge, the fourth leading cause of mortality worldwide[1] and the key risk factor for non-communicable diseases (NCDs) such as cardiovascular diseases, cancer and diabetes.[2] Almost a third of the global health burden comprises NCDs which account for 1.4billion disability-adjusted life years (DALYs).[3] At present most of the global NCD burden is from low-income and middle-income countries (LMICs) (as defined by[4] the World Bank, Classification 2017)[4] and this burden is largely attributable to levels of physical inactivity in the population.[5 6] Prevalence of physical inactivity in 2010 in upper middle-income countries was 25.4% (19.1%–33.7%), followed by 16.8% (11.8%–26.4%)%) in lower middle-income countries and 16.6% (11.8%–26.4%)%) in low-income countries showing that large populations in LMICs[7] are affected by physical inactivity and are therefore at risk of developing NCDs. The data available for the year 2004 show that 3.2million deaths worldwide were due to physical inactivity of which 2.6million were reported from LMICs.[8] LMICs have experienced urbanisation, shift of occupations from agriculture to industrial practice and modernisation including automation, and this has resulted in increased levels of physical inactivity in these populations.[9]

Increasing physical activity and reducing the burden of NCDs is the main goal of public health policies in LMICs.[10] It is a personal, corporate and government responsibility to

For peer review only - http://bmjopen.bmj.com/site/about/guidelines.xhtml

BMJ Open: first published as 10.1136/bmjopen-2018-022686 on 17 January 2019. Downloaded from http://bmjopen.bmj.com/ on August 18, 2020 at Sri Lanka:BMJ-PG Sponsored. Protected by copyright.

reduce the economic burden of unhealthy lifestyles in LMICs.[10] Economic analysis could uncover subtle characteristics of individual decision making on lifestyle choices, estimate the opportunity costs of not doing anything to improve population levels of physical activity and help evaluate what interventions work efficiently to achieve the goal of addressing the physical inactivity pandemic, particularly in LMICs.[11] The current focus of promoting physical activity has shifted from the traditional education approach to environment and policy approaches. Economic perspectives and public health perspectives complement each other in promotion of physical activity.[12] Economic perspectives inform the design of effective physical activity interventions (identifying economic barriers to physical activity) and the efficient allocation of resources—critical to the economies of LMICs.[12]

Our scoping exercise did not identify any published reviews that address the economics of physical activity in LMICs. Thus economic evidence on physical activity in LMICs could be limited or scattered.[13] Therefore, we designed this review to address the following research questions: (1) What is the available evidence base and research gaps on the economics of physical activity in LMICs? (2) What is the focus of and methods for underpinning economic research on physical activity in LMICs? (3) What are the target variables and cost-effective interventions for physical activity policy in LMICs?

This review will add to the scientific literature, provide an overview of the economic evidence base of physical activity in LMICs and fill the gaps in the available evidence regarding this. Providing an up-to-date synthesis of the economic evidence base is an efficient way of highlighting current research practices and new findings to inform researchers, and formulation of cost-effective physical activity programmes and policies.

Objectives

The review will:

- Summarise available evidence on economics of physical activity in LMICs.
- Describe the focus and methods underpinning research on economics of physical activity in LMICs.
- Identify potential target variables and cost-effective interventions for physical activity in LMICs for policy formation.
- Identify and report gaps in research on economics of physical activity in LMICs to provide recommendations for the economic research agenda in LMICs.

METHODS

The methods for this review were informed by previous reviews of economic analyses of physical activity[10 13–19] and are in line with recommendations on review of economic evidence.[20–22] This will be based on the ‘Preferred Reporting Items for Systematic Review and Meta Analyses Protocols (PRISMA-P)’ Statement[23] (online supplementary file 1).

Eligibility criteria

Economic studies on physical activity in LMICs will be included. Specifically, the following study types will be included: (1) Economic evaluations of physical activity interventions. (2) Economic burden of physical inactivity. (3) Cost of physical activity participation. (4) Demand for physical activity. (5) Economic correlates of physical (in) activity. Physical activity is defined as any bodily movement produced by skeletal muscles that require energy expenditure—including activities undertaken while walking, playing, carrying out household chores, travelling and engaging in recreational pursuits.[24] Physical inactivity is considered as a lack of physical activity.[25]

Physical activity interventions will not be specifically defined for this review in order to identify any physical activity intervention in LMICs. However, studies of which an economic analysis was carried out along with the intervention will be included in this review. This strategy will enable consolidation of all available evidence on economics of physical activity related to physical activity interventions.

Eligibility criteria are determined by relevant elements of the population, intervention, comparator, outcome, study design (PICOS criteria).[26] Interventions and comparators will be applicable only to intervention studies.

Inclusion criteria

- Study setting: Any setting of LMICs in accordance with the definition of LMIC by World Bank, Classification 2017.[4]
- Population: Any population in any age group across the life course.
- Intervention: Any physical activity intervention in which the economic evaluation of the intervention has been carried out.
- Comparator: Normal routine, no intervention.
- Outcomes: (1) Cost-effectiveness ratio, quality-adjusted life years (QUALY), incremental cost-effectiveness ratio assessed as the outcomes of physical activity interventions. (2) Cost of physical (in) activity in terms of healthcare cost and/or productivity loss and/or total cost of physical inactivity. (3) Measures of association of any economic variable with physical activity are defined as the primary outcomes of this review.
- Study design: Observational studies (cohort, case control, cross-sectional); correlational studies, experimental studies including randomised controlled trials; quasi-experimental studies; natural experiments; and economic evaluation studies.
- Studies reported only in the English language.

Exclusion criteria

- Case reports, case series, letters to the editor, editorials, reviews, qualitative studies, unpublished theses, conference abstracts and any unobtainable texts.
- Studies published in a language other than English.

For peer review only - http://bmjopen.bmj.com/site/about/guidelines.xhtml
BMJ Open: first published as 10.1136/bmjopen-2018-022686 on 17 January 2019. Downloaded from http://bmjopen.bmj.com/ on August 18, 2020 at Sri Lanka:BMJ-PG Sponsored. Protected by copyright.

Search strategy

The selection of databases was performed by a scoping review of methods of systematic reviews in the field.[13 17 18 27 28] We will search the following electronic databases to identify studies: Scopus (covers 100% MEDLINE coverage, 100% of EMBASE coverage and 100% of Compendex coverage[29]), Web of Science and SPORTDiscus. Websites of WHO, National Institute for Health and Care Excellence (NICE) international and World Bank will be searched for relevant studies. The reference lists of included studies will be searched for any relevant articles. Searches will include publications up to December 2017.

The search strategy was developed based on the scoping review that covered relevant reviews on economic studies on physical activity,[10 13–19] reviews on physical activity[27 30 31] and reviews on economic evaluations.[28 32 33] The draft search strategy was then reviewed by subject experts and a subject liaison librarian to optimise the sensitivity and specificity of the search. Online supplementary file 2 shows a sample search strategy.

Study selection

A two-stage approach involving three independent reviewers will be used to select relevant papers.

At the first stage, two reviewers (PDR and NKA) will independently screen the titles and abstracts of identified papers. Disagreement will be resolved through discussions with a third reviewer (SP). We will include a study if in doubt about its inclusion. For example, if the country setting of a study is unclear in the title and abstract, we will take it forward to the next stage.

At the second stage, the full text of papers selected from stage 1, will be reviewed independently by two reviewers (PDR and NKA). Selected and excluded papers, with reasons, will be discussed by the two reviewers and a third reviewer (SP) at a consensus meeting and disagreement will be resolved by real time consensus. Corresponding authors will be contacted via email for clarification if needed. The study selection process will be presented in a PRISMA flow chart[34] along with the reasons for the exclusion of studies.

Data management and extraction

Endnote X7 software will be used to manage the search results. Data extraction will be performed using a standardised pilot-tested data extraction form developed based on relevant data extraction forms from relevant reviews.[17 35 36] The adaptation process involved matching the review objectives with the available data extraction forms. Data from the final selected full-text articles will be extracted by one reviewer (PDR). To ensure quality of data extraction, a second reviewer (NKA) will independently extract data from a random selection of 50% of the final articles. Disagreements between the two reviewers will be discussed and resolved through discussions with the third reviewer (SP). Any unresolved disagreements will be reported in the final report. Online supplementary file 3 shows the draft data extraction form. Data will be extracted for the following items: general information, characteristics of the study, characteristics of the population/condition/intervention, data sources/data analysis/outcomes, conclusions and the way forward suggested by the authors, challenges and quality assessment.

Risk of bias and quality assessment

Risk of bias will be assessed by a one reviewer (PDR) for all the selected articles using standard checklists based on the study design. A second reviewer (NKA) will also independently assess the risk of bias on 50% of the randomly selected articles included.

Checklists are considered a reliable means of ensuring that all studies included are critically appraised in a standard way.[26] These will assess the information bias which could occur due to the methods, study design, data collection, data analysis and interpretation, and selection bias, where relevant. The most appropriate checklists for each expected study design for risk of bias assessment were selected based on relevant literature in this area[15 17 36–40] and by consensus with all three reviewers (online supplementary file 4). Risk of bias will be assessed using the following tools:

- Economic evaluation studies
  The Drummond checklist[41] which consists of 35 items will be used. It mainly assesses the quality of the study in relation to methods, study design, data collection, data analysis and interpretations. If an economic evaluation study is based on a decision analytical model, the Phillips[42] checklist will be used in addition.
- Cost of illness studies
  The Larg & Moss checklist[43] will be used to assess the quality of the study. It would assess the possible biases related to the analytical framework: what costs should have been measured, methodology and data; how well were resources used and productivity losses measured including representativeness of data for the study population, analysis and reporting. Both internal validity and external validity will be appraised.
- Studies on correlation or association
  The checklist recommended by NICE[44] will be used. Risk of bias related to sample selection, method of selection of exposure/comparison group, outcomes and analysis will be assessed. Both internal and external validity will be appraised.

Quality grading will be given to each included study based on the overall quality of the study. Grading for internal validity and external validity will be reported separately, where relevant. The grading process will follow the NICE recommendations[37] (figure 1):

- Good quality (++): almost all checklist criteria have been fulfilled; where they have not been fulfilled the conclusions are very unlikely to alter.
- Moderate quality (+): some of the checklist criteria are fulfilled; where they have not been fulfilled or not adequately described, conclusions are unlikely to alter.

For peer review only
For peer review only - http://bmjopen.bmj.com/site/about/guidelines.xhtml
BMJ Open: first published as 10.1136/bmjopen-2018-022686 on 17 January 2019. Downloaded from http://bmjopen.bmj.com/ on August 18, 2020 at Sri Lanka:BMJ-PG Sponsored. Protected by copyright.

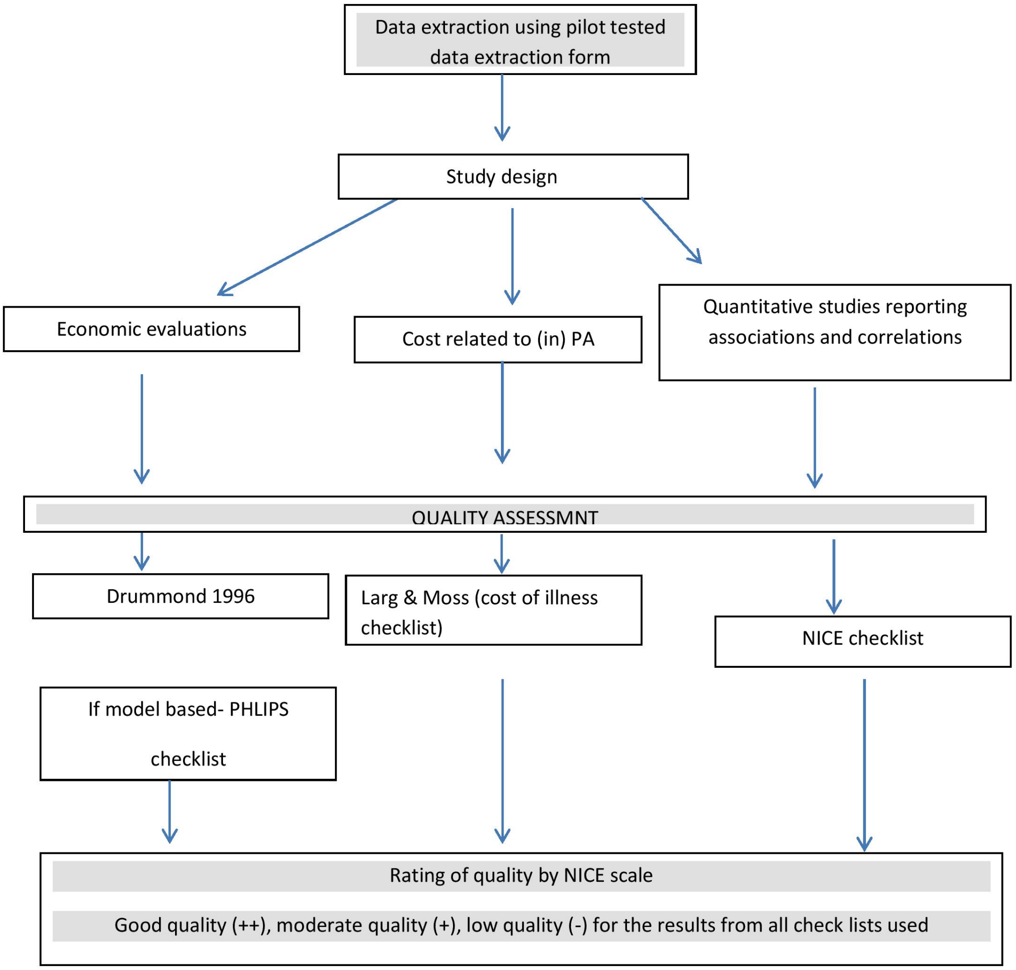

Figure 1 Economics of physical activity (PA) in low-income and middle-income countries (LMICs)—plan for data extraction and the quality assessment process. NICE, National Institute for Health and Care Excellence.

- Low quality (−): few or no checklist criteria are fulfilled, and conclusions are likely or very likely to alter.
- The quality of the included studies will be reported and critically appraised for each study. However, studies will not be excluded based on quality, as this review is particularly aimed to capture the evidence base on economics of physical activity in LMICs and appraise the methods underpinning the evidence base.

Data synthesis and reporting

A descriptive synthesis of data is planned due to the expected heterogeneity of the studies. Summaries of studies will be classified according to type of economic analysis, country or country category, characteristics of the study by PICOS criteria (where applicable); population, intervention, comparator, outcome, study design and quality of studies to describe the overview of available economic evidence base of physical activity in LMICs. Where the numbers permit, summaries of the studies will be categorised based on the focus of the study; economic burden of physical inactivity, economic analysis of physical activity interventions, economic correlates of physical activity including association of physical activity and cost (healthcare cost and/or productivity loss). Where appropriate, we will conduct a meta-analysis to quantify the effect sizes. Such analyses will adjust for between-study heterogeneity using random-effects models. The I-squared (I^2) statistic will be used to evaluate the heterogeneity related to the findings of the papers. Quantitative analysis will be conducted using STATA V.13 software.

We will report the number of studies where data extraction items were not applicable, as an indicator of the quality of reporting. Author-stated limitations of included studies and recommendations for future research and policy will be presented. This review will be reported in accordance with the PRISMA 2009 statement.[34] The study selection process will be illustrated by a flow diagram that will include the reasons for exclusion of studies at each stage. The search strategy, data extraction forms and quality assessment tools will be published as online supplementary files. This protocol will not be amended and any changes will be described and discussed in the final report.

For peer review only - http://bmjopen.bmj.com/site/about/guidelines.xhtml

BMJ Open: first published as 10.1136/bmjopen-2018-022686 on 17 January 2019. Downloaded from http://bmjopen.bmj.com/ on August 18, 2020 at Sri Lanka:BMJ-PG Sponsored. Protected by copyright.

Study status

A scoping review of economics of physical activity in LMICs was carried out in November 2017 to inform the methods of the current review. It identified systematic reviews conducted on economics of physical activity and the gaps in knowledge. It has not been submitted for publication. A review of economics of physical activity in LMICs was designed and methods were developed in December 2017. The search strategy was piloted in January 2018 and the protocol was developed in February 2018. The first submission of the protocol for peer review was on 1 March 2018. Data collection will be commenced upon approval of the protocol for publication and will be completed in 2 months.

Patient and public involvement

Patients and public are not directly involved in this study as original data will not be collected.

Best practice guidelines to conduct systematic reviews[33] will be followed.

DISCUSSION

The level of physical inactivity of the population in LMICs is likely to increase giving rise to a multitude of public health and economic consequences, including rising healthcare costs due to increased NCD-related treatments, increased DALYs and productivity losses. Economic research on physical activity from the perspective of LMICs should therefore be considered a priority. This is because increased physical inactivity leads to uncontrolled healthcare needs in LMICs, where resources (including that for healthcare) are limited and often shrinking in real terms. Pooling of the available research evidence on economics of physical activity in LMICs will thus reveal the current knowledge on economics of physical activity in LMICs, thereby guiding the future of national, regional and local policies around physical activity.

This will be the first study to review the evidence base on economics of physical activity in the context of LMICs which can be considered as the main strength of this study. This will identify gaps in knowledge to support future studies in this area. However, we will only include those studies that are published as peer-reviewed articles and are published in the English language. Thus, possibility of publication bias and language bias cannot be excluded. Furthermore, by assessing and reporting the quality of included studies using standard quality assessment checklists, the possibility of reporting bias of this review can be minimised.

WHO is in the process of drafting the 'Global action plan on physical activity (GAPPA) to be implemented from 2018 to 2030'.[45] This is a timely study as the evidence from this research will provide useful information for implementation and prioritise the actions of GAPPA in LMICs. Evidence from this research will be useful for policy makers and stakeholders dealing with physical activity promotion at individual, local, national, regional and global levels, with special reference to LMICs. Further, this review will be an important base for a research agenda on economics of physical activity in LMICs. This will be a useful guide for researchers to design research on economics and physical activity with sound methodology, based on the research needs of LMICs. Furthermore, information gathered from this research will guide funding agencies for effective allocation of resources.

Contributors SP and NKA developed the idea for the review with inputs from PDR. PDR wrote the first draft. SP and NKA revised the protocol. NKA will act as guarantor of the review.

Funding The authors have not declared a specific grant for this research from any funding agency in the public, commercial or not-for-profit sectors.

Competing interests None declared.

Patient consent for publication Not required.

Provenance and peer review Not commissioned; externally peer reviewed.

Open access This is an open access article distributed in accordance with the Creative Commons Attribution Non Commercial (CC BY-NC 4.0) license, which permits others to distribute, remix, adapt, build upon this work non-commercially, and license their derivative works on different terms, provided the original work is properly cited, appropriate credit is given, any changes made indicated, and the use is non-commercial. See: http://creativecommons.org/licenses/by-nc/4.0/.

REFERENCES

1. World Health Organization. *Global recommendations on physical activity for health*. Geneva: World Health Organization, 2010.
2. World Health Organization. Physical activity [Fact Sheet – Media centre]. 2017 http://www.who.int/mediacentre/factsheets/fs385/en/ (Accessed 6 Feb 2018).
3. Murray CJ, Barber RM, Foreman KJ, *et al*. Global, regional, and national disability-adjusted life years (DALYs) for 306 diseases and injuries and healthy life expectancy (HALE) for 188 countries, 1990-2013: quantifying the epidemiological transition. *Lancet* 2015;386:2145–91.
4. Bank W. World bank country and lending groups: country classification. 2017 https://datahelpdesk.worldbank.org/knowledgebase/articles/906519-world-bank-country-and-lending-groups (Accessed 1st Dec 2017).
5. Abegunde DO, Mathers CD, Adam T, *et al*. The burden and costs of chronic diseases in low-income and middle-income countries. *Lancet* 2007;370:1929–38.
6. Hallal PC, Andersen LB, Bull FC, *et al*. Global physical activity levels: surveillance progress, pitfalls, and prospects. *Lancet* 2012;380:247–57.
7. WHO. Prevalence of insufficient physical activity among adults, data by world bank income groups. 2015 http://apps.who.int/gho/data/view.main.2487?lang=en (Accessed 29th Jan 2018).
8. WHO. Global health risks: mortality and burden of disease attributable to selected major risks. ISBN 978 92 4 156387 1. 2009. Retrieved from http://www.who.int/healthinfo/global_burden_disease/GlobalHealthRisks_report_full.pdf (Accessed 25th Jan 2018).
9. Atkinson K, Lowe S, Moore S. Human development, occupational structure and physical inactivity among 47 low and middle income countries. *Prev Med Rep* 2016;3:40–5.
10. Oldridge NB. Economic burden of physical inactivity: healthcare costs associated with cardiovascular disease. *Eur J Cardiovasc Prev Rehabil* 2008;15:130–9.
11. Islam SM, Purnat TD, Phuong NT, *et al*. Non-communicable diseases (NCDs) in developing countries: a symposium report. *Global Health* 2014;10:81.
12. Sturm R. Economics and physical activity: a research agenda. *Am J Prev Med* 2005;28:141–9.
13. Ding D, Kolbe-Alexander T, Nguyen B, *et al*. The economic burden of physical inactivity: a systematic review and critical appraisal. *Br J Sports Med* 2017;51:1392–409.
14. Barte JCM, Wendel-Vos GCW. A systematic review of financial incentives for physical activity: the effects on physical activity and related outcomes. *Behav Med* 2017;43:79–90.
15. Abu-Omar K, Rütten A, Burlacu I, *et al*. The cost-effectiveness of physical activity interventions: a systematic review of reviews. *Prev Med Rep* 2017;8:72–8.

For peer review only - http://bmjopen.bmj.com/site/about/guidelines.xhtml

BMJ Open: first published as 10.1136/bmjopen-2018-022686 on 17 January 2019. Downloaded from http://bmjopen.bmj.com/ on August 18, 2020 at Sri Lanka:BMJ-PG Sponsored. Protected by copyright.

For peer review only

16. Brown V, Zapata Diomedi B, Moodie M, *et al*. A systematic review of economic analyses of active transport interventions that include physical activity benefits. *BMJ Open* 2016;45.
17. Pavey TG, Taylor AH, Fox KR, *et al*. Effect of exercise referral schemes in primary care on physical activity and improving health outcomes: systematic review and meta-analysis. *BMJ* 2011;343:d6462.
18. Campbell F, Holmes M, Everson-Hock E, *et al*. A systematic review and economic evaluation of exercise referral schemes in primary care: a short report. *Health Technol Assess* 2015;19:1–110.
19. Suhrcke M, Boluarte TA, Niessen L. A systematic review of economic evaluations of interventions to tackle cardiovascular disease in low- and middle-income countries. *BMC Public Health* 2012;12:2.
20. Kaltenthaler E, Tappenden P, Paisley S. Reviewing the evidence to inform the population of cost-effectiveness models within health technology assessments. *Value Health* 2013;16:830–6.
21. Zechmeister-Koss I, Schnell-Inderst P, Zauner G. Appropriate evidence sources for populating decision analytic models within health technology assessment (HTA): a systematic review of HTA manuals and health economic guidelines. *Med Decis Making* 2014;34:288–99.
22. NICE. *The guideline manual process and methods (PMG6)*: NICE, 2012.
23. Moher D, Shamseer L, Clarke M, *et al*. Preferred reporting items for systematic review and meta-analysis protocols (PRISMA-P) 2015 statement. [Research Support, Non-U S Gov't Research Support, U S Gov't, P H S]. *Syst Rev* 2015;4:2046–4053.
24. WHO. *Physical Activity*. [Fact sheet]. 2018 http://www.who.int/en/news-room/fact-sheets/detail/physical-activity (Accessed 15th Jan 2018).
25. WHO. Global Strategy on Diet, Physical activity and Health. 2018. Retrieved from https://www.who.int/dietphysicalactivity/pa/en/ (Accessed 6th Oct 2018).
26. University of York. *Systematic reviews: CRD's guidance for undertaking reviews in health care*. York: University of York, 2009.
27. Milton K, Macniven R, Bauman A. Review of the epidemiological evidence for physical activity and health from low- and middle-income countries. *Glob Public Health* 2014;9:369–81.
28. Nosratnejad S, Rashidian A, Dror DM. Systematic review of willingness to pay for health insurance in low and middle income countries. *PLoS One* 2016;11:e0157470.
29. Burnham JF. Scopus database: a review. *Biomed Digit Libr* 2006;3:1742–5581.
30. Lachat C, Otchere S, Roberfroid D, *et al*. Diet and physical activity for the prevention of noncommunicable diseases in low- and middle-income countries: a systematic policy review. *PLoS Med* 2013;10:e1001465.
31. Hunter RF, Christian H, Veitch J, *et al*. The impact of interventions to promote physical activity in urban green space: a systematic review and recommendations for future research. *Soc Sci Med* 2015;124:246–56.
32. Sutton L, Karan A, Mahal A. Evidence for cost-effectiveness of lifestyle primary preventions for cardiovascular disease in the Asia-Pacific Region: a systematic review. *Global Health* 2014;10:79.
33. Santatiwongchai B, Chantarastapornchit V, Wilkinson T, *et al*. Methodological variation in economic evaluations conducted in low- and middle-income countries: information for reference case development. *PLoS One* 2015;10:e0123853.
34. Moher D, Liberati A, Tetzlaff J, *et al*. Preferred reporting items for systematic reviews and meta-analyses: the PRISMA statement. [Guideline Research Support, Non-U S Gov't]. *J Clin Epidemiol* 2009;62:1006–12.
35. Pokhrel S, Quigley MA, Fox-Rushby J, *et al*. Potential economic impacts from improving breastfeeding rates in the UK. [Research Support, Non-U.S. Gov't]. *Arch Dis Child* 2015;100:334–40.
36. Murthy S, John D, Godinho IP, *et al*. A protocol for a systematic review of economic evaluation studies conducted on neonatal systemic infections in South Asia. *Syst Rev* 2017;6:252.
37. Aminde LN, Veerman L. Interventions for the prevention of cardiovascular diseases: a protocol for a systematic review of economic evaluations in low-income and middle-income countries. *BMJ Open* 2016;6:e013668.
38. Ruifrok AE, Rogozinska E, van Poppel MN, *et al*. Study protocol: differential effects of diet and physical activity based interventions in pregnancy on maternal and fetal outcomes--individual patient data (IPD) meta-analysis and health economic evaluation. *Syst Rev* 2014;3:131.
39. Gc V, Wilson EC, Suhrcke M, *et al*. Are brief interventions to increase physical activity cost-effective? A systematic review. *Br J Sports Med* 2016;50:408–17.
40. Tonmukayakul U, Calache H, Clark R, *et al*. Systematic review and quality appraisal of economic evaluation publications in dentistry. *J Dent Res* 2015;94:1348–54.
41. Drummond MF, Jefferson TO. Guidelines for authors and peer reviewers of economic submissions to the BMJ. *BMJ* 1996;313:275–83.
42. Philips Z, Ginnelly L, Sculpher M, *et al*. Review of guidelines for good practice in decision-analytic modelling in health technology assessment. *Health Technol Assess* 2004;8:1–158.
43. Larg A, Moss JR. Cost-of-illness studies: a guide to critical evaluation. [Research Support, Non-U S Gov't]. *Pharmacoeconomics* 2011;29:653–71.
44. National Institute for Health and Excellence (NICE). Methods for the development of NICE public health guidance (third edition), Appendix G Quality appraisal checklist-quantitative studies reporting correlations and associations. 2012 https://www.nice.org.uk/process/pmg4/chapter/appendix-g-quality-appraisal-checklist-quantitative-studies-reporting-correlations-and (Accessed 2nd Jan 2018).
45. World Health Organization. *Non communicable diseases and their risk factors, governance*: Development of draft global action plan to promote physical activity, 2018.

For peer review only

BMJ Open: first published as 10.1136/bmjopen-2018-022686 on 17 January 2019. Downloaded from http://bmjopen.bmj.com/ on August 18, 2020 at Sri Lanka:BMJ-PG Sponsored. Protected by copyright.

Search Strategy

Scopus

((TITLE-ABKEY

((*cost* W/2 (*analyses* OR *effectiveness* OR *benefit* OR *minimization* OR *utility* OR *saving* OR *outcome* OR *consequence*))) AND DOCTYPE (*ar* OR *re*)) OR (TITLE-ABS-KEY ((*"demand"* OR *"supply"* OR *"market"* OR *"price"*)) AND DOCTYPE (*ar* OR *re*)) OR (TITLE-ABS-KEY ((*"incentives"* OR *"willingness to pay"* OR *"time use"*)) AND DOCTYPE (*ar* OR *re*)) OR (TITLE-ABS-KEY (*"economics"*) AND DOCTYPE (*ar* OR *re*)) OR (TITLE-ABS-KEY ((*"economic evaluation"* OR *"economic burden"* OR *"health care cost"*)) AND DOCTYPE (*ar* OR *re*))) AND ((TITLE-ABS-KEY (((((*active* W/2 (*lifestyle* OR *living* OR *leisure* OR *play* OR *recreation* OR *commuting*)))))) AND DOCTYPE (*ar* OR *re*)) OR (TITLE-ABS-KEY ((*"physical activity"* OR *"physical exercise"*)) AND DOCTYPE (*ar* OR *re*)) OR (TITLE-ABS-KEY ((*"physical inactivity"* OR *"physical fitness"*)) AND DOCTYPE (*ar* OR *re*)) OR (TITLE-ABS-KEY (*"Sedentary"*) AND DOCTYPE (*ar* OR *re*))) AND (LIMIT-TO (LANGUAGE , *"English"*))

SPORTDiscus

((TITLE-ABS-KEY ((*cost* n/2 (*analyses* OR *effectiveness* OR *benefit* OR *minimization* OR *utility* OR *saving* OR *outcome* OR *consequence*))) AND DOCTYPE (*ar* OR *re*)) OR (TITLE-ABS-KEY ((*"demand"* OR *"supply"* OR *"market"* OR *"price"*)) AND DOCTYPE (*ar* OR *re*)) OR (TITLE-ABS-KEY ((*"incentives"* OR *"willingness to pay"* OR *"time use"*)) AND DOCTYPE (*ar* OR *re*)) OR (TITLE-ABS-KEY (*"economics"*) AND DOCTYPE (*ar* OR *re*)) OR (TITLE-ABS-KEY ((*"economic evaluation"* OR *"economic burden"* OR *"health care cost"*)) AND DOCTYPE (*ar* OR *re*))) AND ((TITLE-ABS-KEY (((((*active* n/2 (*lifestyle* OR *living* OR *leisure* OR *play* OR *recreation* OR *commuting*)))))) AND DOCTYPE (*ar* OR *re*)) OR (TITLE-ABS-KEY ((*"physical activity"* OR *"physical exercise"*)) AND DOCTYPE (*ar* OR *re*)) OR (TITLE-ABS-KEY ((*"physical inactivity"* OR *"physical fitness"*)) AND DOCTYPE (*ar* OR *re*)) OR (TITLE-ABS-

For peer review only

KEY (*"Sedentary"*) AND DOCTYPE (*ar* OR *re*))) AND (LIMIT-TO (LANGUAGE , *"English"*))

Web of Science

((TITLE-ABS-KEY ((*cost* near/15 (*analyses* OR *effectiveness* OR *benefit* OR *minimization* OR *utility* OR *saving* OR *outcome* OR *consequence*))) AND DOCTYPE (*ar* OR *re*)) OR (TITLE-ABS-KEY ((*"demand"* OR *"supply"* OR *"market"* OR *"price"*)) AND DOCTYPE (*ar* OR *re*)) OR (TITLE-ABS-KEY ((*"incentives"* OR *"willingness to pay"* OR *"time use"*)) AND DOCTYPE (*ar* OR *re*)) OR (TITLE-ABS-KEY (*"economics"*) AND DOCTYPE (*ar* OR *re*)) OR (TITLE-ABS-KEY ((*"economic evaluation"* OR *"economic burden"* OR *"health care cost"*)) AND DOCTYPE (*ar* OR *re*))) AND ((TITLE-ABS-KEY (((((*active* near/15 (*lifestyle* OR *living* OR *leisure* OR *play* OR *recreation* OR *commuting*)))))) AND DOCTYPE (*ar* OR *re*)) OR (TITLE-ABS-KEY ((*"physical activity"* OR *"physical exercise"*)) AND DOCTYPE (*ar* OR *re*)) OR (TITLE-ABS-KEY ((*"physical inactivity"* OR *"physical fitness"*)) AND DOCTYPE (*ar* OR *re*)) OR (TITLE-ABS-KEY (*"Sedentary"*) AND DOCTYPE (*ar* OR *re*))) AND (LIMIT-TO (LANGUAGE , *"English"*))

For peer review only

Table 1: List of excluded studies following full text screening with reasons

1. Is Exercise a Good Investment for Adolescents? (1991). Journal of Sport & Exercise Psychology, 13(2), 208-208.	It is a brief one page document and not a research article. country studied also not mentioned
2. Codogno, J. S., Fernandes, R. A., & Monteiro, H. L. (2012). Physical activity and healthcare cost of type 2 diabetic patients seen at basic units of healthcare. [Article]. Arquivos Brasileiros de Endocrinologia e Metabologia, 56(1), 6-11	Article in Portuguese. (though English abstract available)
3. Ng, S. W., & Popkin, B. M. (2012). Time use and physical activity: A shift away from movement across the globe. [Article]. Obesity Reviews, 13(8), 659-680. doi: 10.1111/j.1467-789X.2011.00982.x	It only reports trends in energy expenditure including sedentary time and various physical activities for brazil, India and china and 2 other developed countries. It does not report how time affect the physical activity.
4. Oldridge, N. B. (2008). Economic burden of physical inactivity: healthcare costs associated with cardiovascular disease. European Journal of Cardiovascular Prevention & Rehabilitation, 15(2), 130-139. doi: 10.1097/HJR.0b013e3282f19d42	Though prevalence estimated for developing countries. cost is estimated only for developed countries
5. Kolbe-Alexander, T. L., Conradie, J., & Lambert, E. V. (2013). Clustering of risk factors for non-communicable disease and healthcare expenditure in employees with private health insurance presenting for health risk appraisal: a cross-sectional study. Bmc Public Health, 13. doi: 10.1186/1471-2458-13-1213	Studied clustering of other NCD risk factors with Physical inactivity, and association of clustered risk factors with health care expenditure. Data on expenditure due to physical inactivity not shown separately.
6. Huang, W. Y., & Wong, S. H. (2016). Time use clusters in children and their associations with sociodemographic factors. [Article]. Journal of Public Health (United Kingdom), 38(2), e106-e113. doi: 10.1093/pubmed/fdv088	Identified clusters according to time use for physical activity and sedentary behaviour. It does not assess time effect on physical activity participation.
7. Jim, C. Y., & Chen, W. Y. (2009). Leisure Participation Pattern of Residents in a New Chinese City. Annals of the Association of American Geographers, 99(4), 657-673. doi: 10.1080/00045600903066482	Assessed the association of leisure time and socioeconomic variables. Barriers to leisure participation identified by using a part of the survey questionnaire as a part of this study. HOWEVER, ASSESSED ONLY THE PERCIEVED BARRIERS.
8. Lopes, A. S., Silva, K. S., Filho, V. C. B., Bezerra, J., De Oliveira, E. S. A., & Nahas, M. V. (2014). Trends in screen time on week and weekend days in a representative sample of Southern Brazil students. [Article]. Journal of Public Health (United Kingdom), 36(4), 608-614. doi: 10.1093/pubmed/fdt133	It basically reports the trend of sedentary screen time.
9. Matsudo, V. K. R., Matsudo, S. M., Araújo, T. L., Andrade, D. R., Oliveira, L. C., & Hallal, P. C. (2010). Time Trends in physical activity in the state of São Paulo, Brazil: 2002-2008. [Article].	Reported only the trend in PA participation by socioeconomic status

For peer review only

Medicine and Science in Sports and Exercise, 42(12), 2231-2236. doi: 10.1249/MSS.0b013e3181e1fe8e	
10. Pratt, M., Norris, J., Lobelo, F., Roux, L., & Guijing, W. (2014). The cost of physical inactivity: moving into the 21st century. British Journal of Sports Medicine, 48(3), 171-173.	This is a review
11. Araujo, M. Y. C., et al. (2016). "Association between disability pension, nutritional condition and physical inactivity in adults from a middle-size Brazilian City." Journal of Physical Education (Maringa) 27(1).	Not directly relevant to economics of physical activity as it assesses the association of disability pension and physical activity.
12. Sujatha, T., et al. (2003). "Timed activity studies for assessing the energy expenditure of women from an urban slum in south India." Food and Nutrition Bulletin 24(2): 193-199.	It does not assess the time effect on physical activity
13. Cecchini, M., et al. "Tackling of unhealthy diets, physical inactivity, and obesity: health effects and cost-effectiveness." The Lancet 376(9754): 1775-1784.	This is only a report, proposed a model and analysed cost for interventions for all healthy life styles by intervention type. Not assessed the cost for physical activity promotion by interventions separately or economic evaluation of such intervention
14. Meng, L., et al. (2013). "The Costs and Cost-Effectiveness of a School-Based Comprehensive Intervention Study on Childhood Obesity in China." PLOS ONE 8(10): e77971.	Cost effectiveness assessed only for combined intervention, not for physical activity alone
15. Moraisa. L.C., et al. Health indicators and costs among outpatients according to physical activity level and obesity. *Diabetes & Metabolic Syndrome: Clinical Research & Reviews,* Volume 13, Issue 2, March–April 2019, Pages 1375-137	This has not assessed physical activity separately but coupled with obesity
16. Min, J. Y. and K. B. Min (2016). Excess Medical Care Costs Associated with Physical Inactivity among Korean Adults: Retrospective Cohort Study. *International Journal of Environmental Research and Public Health,* **13**(1).	This study directly does not indicate which part of the Korea. However, the insurance scheme in this study is functioning in South Korea, which is a high-income country.

For peer review only

DATA EXTRACTION FORM FOR REVIEW ON ECONONOMICS AND PYSICAL ACTIVITY IN LOW AND MIDDLE INCOME COUNTRIES (ADAPTED FROM MURTHEY ET AL., 2017, POKHREL ET AL., 2014, PAVEY ET AL., 2011)

**1. GENERAL INFORMATION**		
1.1	Study ID	
1.2	Review author ID	
1.3	Date of review	
1.4	Title	
1.5	Author(s)	
1.6	Year of publication	
1.7	Country studied	
1.8	Income level of the country	
**2. CHRACTERISTIC OF THE STUDY**		
2.1	Aims and objectives/research question	
2.2	Eligibility decision Inclusion and exclusion criteria (?)	
2.3	Study design	
2.4	Study setting	
2.5	Total study duration	
2.6	Sample size	
2.7	Sample size calculation	
2.8	Recruitment	
2.9	Sample selection	
2.10	Method of allocation	
2.11	Perspective (i.e. Societal, health care system, service provider etc.)	
**3. CHARACTERISTICS OF POPULATION/CONDITION/INTERVENTION**		
3.1	**Population/participants**	
3.1.1	Type of population	

	(i.e. General population university students, group of employees)	
3.1.2	Age	
3.1.3	Gender	
3.1.4	Ethnicity	
3.2	**Condition**	
3.2.1	Definition of physical activity /physical inactivity/sedentary	
3.2.2	Definition of disease condition	
3.3	**Intervention**	
3.3.1	Type of physical activity/programme	
3.3.2	Comparator	
4.	**Data sources/Data analysis/results**	
4.1	**scope**	
4.1.1	Type of analysis<br>(Ie.Costing/economic evaluation/association of economic factors with physical activity/other)	
4.1.2	Form of economic evaluation	
4.1.3	Time horizon	
4.1.4	Study assumptions	
4.2	**Cost**	
4.2.1	Reported costs/cost components	
4.2.2	Data sources for cost measures	
4.2.3	How cost was reported<br>(i.e. marginal cost/ average cost/incremental cost/total cost/other)	
4.2.4	Discount rate	
4.2.5	Year of costing	
4.2.6	Currency unit	
4.2.7	Currency conversion rate	

For peer review only

4.2.8	Inflation adjustments	
4.3	**Outcomes**	
4.3.1	Reported outcomes (i.e. measures of effectiveness/efficacy/measures of effect)	
4.3.2	Data sources for the outcome measures	
4.3.4	Discount rate	
4.4	**Main Analysis**	
4.4.1	Statistical tests used	
4.4.2	Models used	
4.4.3	Economic evaluations	
4.5	**Sensitivity analysis**	
4.5.1	Performed/ not	
4.5.2	Type of analysis (i.e. deterministic/ stochastic)	
4.5.3	Variables used for sensitivity analysis	
4.5.4	Results of sensitivity analysis	
4.6	**Main results**	
4.6.1	Cost / cost effectiveness	
4.6.2	Other	
5.	**CONCLUSIONS AND WAY FORWARD**	
5.1	Key Conclusion(s)	
5.2	Suggestions by author for further research	
6.	**CHALLENGES**	
6.1	Author stated limitations	
6.2	Author stated strengths	
6.3	Miscellaneous comments from author	
6.4	Funding source	
6.5	Conflicts of interest	
**7.**	**QUALITY ASSESSMENT OF STUDIES**	

For peer review only

7.1	Is the study economic evaluation?	Yes/No, if *yes go to* Check list A
7.2	Is the economic evaluation model based?	Yes/No , if *yes* go to Check list B
7.3	Is it a cost of illness/cost of risk factor study?	Yes/No , if *yes* go to Check list C
7.4	Is it a quantitative study reporting correlation or association?	Yes/No , If *yes* go to Checklist D

**Method of rating the quality of studies from each check list (A, B, C, D) in accordance to the quality appraisal by NICE scale (Methods for the development of NICE public health guidance (third edition))**	
• Reviewer is requested to complete in detail the comment section of quality appraisal check lists so that the grade awarded for each study aspect is as transparent as possible<br>• At the end of completion of the check list each study is awarded an overall quality grading for internal validity (IV)and a separate one for external validity (EV)	
All or most of the checklist criteria have been fulfilled, where they have not been fulfilled the conclusions are very unlikely to alter	++
Some of the checklist criteria have been fulfilled, where they have not been fulfilled, or not adequately described, the conclusions are unlikely to alter	+
Few or no checklist criteria have been fulfilled and the conclusions are likely or very likely to alter.	-

<u>CHECKLIST A</u>

For peer review only

QUALITY ASSESSMENT FOR ECONOMIC EVALUATION(CHECKLIST FROM DRUMMOND AND JEFFERSON 1996)	Yes	No	Not clear	Not appropriate
**Study design**				
1) The research question is stated				
2) The economic importance of the research question is stated				
3) The viewpoint(s) of the analysis are clearly stated and justified				
4) The rationale for choosing the alternative programmes orb interventions compared is stated				
5) The alternatives being compared are clearly described				
6) The form of economic evaluation used is stated				
7) The choice of form of economic evaluation is justified in relation to the questions addressed				
**Data collection**				
8) The source(s) of effectiveness estimates used are stated				
9) Details of the design and results of effectiveness study are given (if based on a single study)				
10) Details of the method of synthesis or meta-analysis of estimates are given (if based on an overview of a number of effectiveness studies)				
11) The primary outcome measure(s) for the economic evaluation are clearly stated				
12) Methods to value health states and other benefits are stated				

For peer review only

QUALITY ASSESSMENT FOR ECONOMIC EVALUATION(CHECKLIST FROM DRUMMOND AND JEFFERSON 1996)	Yes	No	Not clear	Not appropriate
13) Details of the subjects from whom valuations were obtained are given				
14) Productivity changes (if included) are reported separately				
15) The relevance of productivity changes to the study question is discussed				
16) Quantities of resources are reported separately from their unit costs				
17) Methods for the estimation of quantities and unit costs are described				
18) Currency and price data are recorded				
19) Details of currency of price adjustments for inflation or currency conversion are given				
20) Details of any model used are given				
21) The choice of model used and the key parameters on which it is based are justified				
**Analysis and interpretation of results**				
22) Time horizon of costs and benefits is stated				
23) The discount rate(s) is stated				
24) The choice of rate(s) is justified				
25) An explanation is given if costs or benefits are not discounted				
26) Details of statistical tests and confidence intervals are given for stochastic data				

For peer review only

QUALITY ASSESSMENT FOR ECONOMIC EVALUATION(CHECKLIST FROM DRUMMOND AND JEFFERSON 1996)	Yes	No	Not clear	Not appropriate
27) The approach to sensitivity analysis is given				
28) The choice of variables for sensitivity analysis is justified				
29) The ranges over which the variables are varied are stated				
30) Relevant alternatives are compared				
31) Incremental analysis is reported				
32) Major outcomes are presented in a disaggregated as well as aggregated form				
33) The answer to the study question is given				
34) Conclusions follow from the data reported				
35) Conclusions are accompanied by the appropriate caveats				

Did the economic evaluation use a decision-analytic modelling framework?	Response: Yes(✓), No (×) , Not applicable (NA)<br><br>Instruction: If *Yes*, assess paper using the questions in Check list B, If *No/NA*, skip check list B

CHECKLIST B

For peer review only

QUALITY ASSESSMENT FOR DECISION-ANALYTIC MODELS(CHECKLIST FROM PHILIPS ET AL. 2004)			
**Quality criterion**	**Question(s)**	**Response (✓, ✗, NA)**	**Comments**
S1	Is there a clear statement of the decision problem?		
	Is the objective of the evaluation and model specified and consistent with the stated decision problem?		
	Is the primary decision maker specified?		
S2	Is the perspective of the model stated clearly?		
	Are the model inputs consistent with the stated perspective?		
	Has the scope of the model been stated and justified?		
	Are the outcomes of the model consistent with the perspective, scope and overall objective of the model?		
S3	Is the structure of the model consistent with a coherent theory of the health condition under evaluation?		
	Are the sources of data used to develop the structure of the model specified?		
	Are the causal relationships described by the model structure justified appropriately?		
S4	Are the structural assumptions transparent and justified?		

For peer review only

QUALITY ASSESSMENT FOR DECISION-ANALYTIC MODELS(CHECKLIST FROM PHILIPS ET AL. 2004)			
**Quality criterion**	**Question(s)**	**Response (✓, ×, NA)**	**Comments**
	Are the structural assumptions reasonable given the overall objective, perspective and scope of the model?		
S5	Is there a clear definition of the options under evaluation?		
	Have all feasible and practical options been evaluated?		
	Is there justification for the exclusion for the exclusion of feasible options?		
S6	Is the chosen model type appropriate given the decision problem and specified causal relationships within the model?		
S7	Is the time horizon of the model sufficient to reflect all important differences between options?		
	Are the time horizon of the model, the duration of treatment and the duration of treatment effect described and justified?		
S8	Do the disease states (state transition model) or the pathways (decision tree model) reflect the underlying biological process of the disease in question and the impact of interventions?		
S9	Is the cycle length defined and justified in terms of natural history of disease?		

For peer review only

QUALITY ASSESSMENT FOR DECISION-ANALYTIC MODELS(CHECKLIST FROM PHILIPS ET AL. 2004)			
**Quality criterion**	**Question(s)**	**Response (✓, ✗, NA)**	**Comments**
D1	Are the data identification methods transparent and appropriate given the objectives of the model?		
	Where choices have been made between data sources, are these justified appropriately?		
	Has particular attention been paid to identifying data for the important parameters in the model?		
	Has the quality of the data been assessed appropriately?		
	Where expert opinion has been used, are the methods described and justified?		
D2	Is the data modelling methodology based on justifiable statistical and epidemiological techniques?		
D2a	Is the choice of baseline data described and justified?		
	Are transition probabilities calculated appropriately?		
	Has a half-cycle correction been applied to both cost and outcome?		
	If not, has this omission been justified?		

For peer review only

QUALITY ASSESSMENT FOR DECISION-ANALYTIC MODELS(CHECKLIST FROM PHILIPS ET AL. 2004)			
**Quality criterion**	**Question(s)**	**Response (✓, ✗, NA)**	**Comments**
D2b	If relative treatment effects have been derived from trail data, have they been synthesised using appropriate techniques?		
	Have the methods and assumptions used to extrapolate short-term results to final outcomes been documented and justified?		
	Have alternative extrapolation assumptions been explored through sensitivity analysis?		
	Have assumptions regarding the continuing effect of treatment once treatment is complete been documented and justified?		
	Have alternative assumptions regarding the continuing effect of treatment been explored through sensitivity analysis?		
D2c	Are the costs incorporated into the model justified?		
	Has the source for all costs been described?		
	Have discount rates been described and justified given the target decision-maker?		
D2d	Are the utilities incorporated into the model appropriate?		
	Is the source for the utility weights referenced?		

For peer review only

QUALITY ASSESSMENT FOR DECISION-ANALYTIC MODELS(CHECKLIST FROM PHILIPS ET AL. 2004)			
**Quality criterion**	**Question(s)**	**Response (✓, ✗, NA)**	**Comments**
	Are the methods of derivation for the utility weights justified?		
D3	Have all data incorporated into the model been described and referenced in sufficient detail?		
	Has the use of mutually inconsistent data been justified (i.e. are assumptions and choices appropriate)?		
	Is the process of data incorporation transparent?		
	If data have been incorporated as distributions, has the choice of distribution for each parameter been described and justified?		
	If data have been incorporated as distributions, is it clear that second order uncertainty is reflected?		
D4	Have the four principal types of uncertainty been addressed?		
	If not, has the omission of particular forms of uncertainty been justified?		
D4a	Have methodological uncertainties been addressed by running alternative versions of the model with different methodological assumptions?		

For peer review only

QUALITY ASSESSMENT FOR DECISION-ANALYTIC MODELS(CHECKLIST FROM PHILIPS ET AL. 2004)			
**Quality criterion**	**Question(s)**	**Response (✓, ✗, NA)**	**Comments**
D4b	Is there evidence that structural uncertainties have been addressed via sensitivity analysis?		
D4c	Has heterogeneity been dealt with by running the model separately for different subgroups?		
D4d	Are the methods of assessment of parameter uncertainty appropriate?		
	If data are incorporated at point estimates, are the ranges used for sensitivity analysis stated clearly and justified?		
C1	Is there evidence that the mathematical logic of the model has been tested thoroughly before use?		
C2	Are any counterintuitive results from the model explained and justified?		
	If the model has been calibrated against independent data, has any differences been explained and justified?		
	Have the results of the model been compared with those of previous models and any differences in results explained?		

For peer review only

For peer review only

CHECKLIST C

QUALITY ASSESSMNET FOR COST OF ILLNESS STUDIES (ADAPTED FROM LARG AND MOSS, 2011)

Responses (marked Similar to NICE check list, write the answer where relevant under the comment section)

++ Indicates that for that particular aspect of study design, the study has been designed or conducted in such a way as to minimise the risk of bias.
+ Indicates that either the answer to the checklist question is not clear from the way the study is reported, or that the study may not have addressed all potential sources of bias for that particular aspect of study design.
- Should be reserved for those aspects of the study design in which significant sources of bias may persist.
NR (Not Reported) - Should be reserved for those aspects in which the study under review fails to report how they have (or might have) been considered.
NA (Not Applicable) Should be reserved for those study design aspects that are not applicable given the study design under review (for example, allocation concealment would not be applicable for case–control studies).

	Question	Response<br>***++/+/-NR/NA***	Comment
1.	Analytical framework: what costs should have been measured?<br><br>(a) What was the motivation and perspective of the study?<br><br>(b) Was the appropriate epidemiologic approach taken?<br><br>(c) Was the study question well specified?<br>(i) Were all relevant, non-trivial cost components and their stakeholders identified?<br>(ii) Were necessary timeframes specified?<br>(iii) Was a case of disease or risk factor adequately and appropriately defined?<br>(iv) Was the counterfactual population occurrence plausible and meaningful?		
2.	(2) Methodology and data: how well were resource use and productivity losses measured?<br><br>(a) Was an appropriate method(s) of quantification used, such that;		

	(i) Additional, or excess, costs were measured?<br>(ii) only costs specific to (caused by) the health problem were included (confounders controlled)?<br>(iii) All important effects were captured?<br>(iv) Important differences across subpopulations were accounted for?<br>(v) The required level of detail could be provided?<br><br>(b) Was the resource quantification method(s) well executed?<br>(i) For population-based studies, were cost allocation methods, data and assumptions valid?<br>(ii) For person-based studies, were appropriate statistical tests performed and reported?<br>(iii) Were data representative of the study population?<br>(iv) Were there any other relevant resource quantification issues?<br><br>(c) Were healthcare resources valued appropriately?<br><br>(d) Was the approach for valuing production losses justified, and assumptions valid?<br><br>(e) Was the inclusion of intangible costs appropriate:<br>(i) Was double counting of mortality-related production losses avoided?<br>(ii) Were losses valued appropriately, given the study's perspective?		
3	Analysis and reporting<br>(a) Did the analysis address the study question?<br><br>(b) Was a range of estimates presented?<br><br>(c) Were the main uncertainties identified?		

For peer review only

	(d) Was a sensitivity analysis performed on:<br><br>(i) important (uncertain) parameter estimates?<br>(ii) key assumptions? (including the counterfactual)<br>(iii) point estimates? (based on confidence or credible intervals)<br><br>(e) Was adequate documentation and justification given for cost components, data and sources, assumptions and methods?<br><br>(f) Was uncertainty around the estimates and its implications adequately discussed?<br><br>(g) Were important limitations discussed regarding the cost components, data, assumptions and methods?<br><br>(h) Were the results presented at the appropriate level of detail to answer the study<br>question (cost components; disease subtypes, severity, stage; subpopulation groups, cost bearers)?		

For peer review only

CHECKLIST D

**QUALITY ASSESSMENT FOR QUANTITATIVE STUDIES REPORTING CORRELATIONS AND ASSOCIATIONS(NICE,2012 BASED ON 'GRAPHICAL APPRAISAL TOOL FOR EPIDEMIOLOGICAL STUDIES(GATE)` BY JACKSON ET AL. (2006)**			
Responses:<br><br>++ Indicates that for that particular aspect of study design, the study has been designed or conducted in such a way as to minimise the risk of bias.<br><br>+ Indicates that either the answer to the checklist question is not clear from the way the study is reported, or that the study may not have addressed all potential sources of bias for that particular aspect of study design.<br><br>- Should be reserved for those aspects of the study design in which significant sources of bias may persist.<br><br>NR (Not Reported) - Should be reserved for those aspects in which the study under review fails to report how they have (or might have) been considered.<br><br>NA (Not Applicable) Should be reserved for those study design aspects that are not applicable given the study design under review (for example, allocation concealment would not be applicable for case–control studies).			
**Section 1: Population**			
**Numb er**	**Question**	**Response**<br><br>**++/+/-/ NR/NA**	**Comments**
1.1	Is the source population or source area well described?		

For peer review only

	Was the country (e.g. developed or non-developed, type of health care system), setting (primary schools, community centres etc), location (urban, rural), population demographics etc adequately described?		
1.2	Is the eligible population or area representative of the source population or area?<br><br>Was the recruitment of individuals, clusters or areas well defined (e.g. advertisement, birth register)?<br><br>Was the eligible population representative of the source? Were important groups underrepresented?		
1.3	Do the selected participants or areas represent the eligible population or area?<br><br>Was the method of selection of participants from the eligible population well described?<br><br>What % of selected individuals or clusters agreed to participate? Were there any sources of bias?<br><br>Were the inclusion or exclusion criteria explicit and appropriate?		
**Section 2: Method of selection of exposure (or comparison) group**			
2.1	Selection of exposure (and comparison) group. How was selection bias minimised?<br><br>How was selection bias minimised?		
2.2	Was the selection of explanatory variables based on a sound theoretical basis?<br><br>How sound was the theoretical basis for selecting the explanatory variables?		

For peer review only

2.3	Was the contamination acceptably low?<br><br>Did any in the comparison group receive the exposure?<br><br>If so, was it sufficient to cause important bias?		
2.4	How well were likely confounding factors identified and controlled?<br><br>Were there likely to be other confounding factors not considered or appropriately adjusted for?<br><br>Was this sufficient to cause important bias?		
**Section 3: Outcomes**			
3.1	Were the outcome measures and procedures reliable?<br><br>Were outcome measures subjective or objective (e.g. biochemically validated nicotine levels ++ vs self-reported smoking −)?<br><br>How reliable were outcome measures (e.g. inter- or intra-rater reliability scores)?<br><br>Was there any indication that measures had been validated (e.g. validated against a gold standard measure or assessed for content validity)?		
3.2	Were the outcome measurements complete?<br><br>Were all or most of the study participants who met the defined study outcome definitions likely to have been identified?		
3.3	Were all important outcomes assessed?		

For peer review only

	Were all benefits and harms assessed?<br><br>Was it possible to determine the overall balance of benefits and harms of intervention versus comparisons?		
3.4	Was there a similar follow-up time in exposure and comparison groups?<br><br>If groups are followed for different lengths of time, then more events are likely to occur in the group followed-up for longer distorting the comparison.<br><br>Analyses can be adjusted to allow for differences in length of follow-up (e.g. using person-years).		
3.5	Was follow-up time meaningful?<br><br>Was follow-up long enough to assess long-term benefits and harms?<br><br>Was it too long, e.g. participants lost to follow-up?		
**Section 4: Analyses**			
4.1	Was the study sufficiently powered to detect an intervention effect (if one exists)?<br><br>A power of 0.8 (i.e. it is likely to see an effect of a given size if one exists, 80% of the time) is the conventionally accepted standard.<br><br>Is a power calculation presented? If not, what is the expected effect size? Is the sample size adequate?		

For peer review only

4.2	Were multiple explanatory variables considered in the analyses?<br><br>Were there sufficient explanatory variables considered in the analysis?		
4.3	Were the analytical methods appropriate?<br><br>Were important differences in follow-up time and likely confounders adjusted for?		
4.4	Was the precision of association given or calculable? Is association meaningful?<br><br>Were confidence intervals or p values for effect estimates given or possible to calculate?<br><br>Were CIs wide or were they sufficiently precise to aid decision-making? If precision is lacking, is this because the study is under-powered?		
**Section 5: Summary**			
5.1	Are the study results internally valid (i.e. unbiased)?<br><br>How well did the study minimise sources of bias (i.e. adjusting for potential confounders)?<br><br>Were there significant flaws in the study design?		
5.2	Are the findings generalizable to the source population (i.e. externally valid)?<br><br>Are there sufficient details given about the study to determine if the findings are generalizable to the source population?		

For peer review only

	Consider: participants, interventions and comparisons, outcomes, resource and policy implications		

For peer review only

CHECKLIST D

**QUALITY ASSESSMENT FOR QUANTITATIVE STUDIES REPORTING CORRELATIONS AND ASSOCIATIONS(NICE,2012 BASED ON 'GRAPHICAL APPRAISAL TOOL FOR EPIDEMIOLOGICAL STUDIES(GATE)` BY JACKSON ET AL. (2006)**
Responses:<br><br>++ Indicates that for that particular aspect of study design, the study has been designed or conducted in such a way as to minimise the risk of bias.

+ Indicates that either the answer to the checklist question is not clear from the way the study is reported, or that the study may not have addressed all potential sources of bias for that particular aspect of study design.

- Should be reserved for those aspects of the study design in which significant sources of bias may persist.

NR (Not Reported) - Should be reserved for those aspects in which the study under review fails to report how they have (or might have) been considered.

NA (Not Applicable) Should be reserved for those study design aspects that are not applicable given the study design under review (for example, allocation concealment would not be applicable for case–control studies).

Section 1: Population

**Number**	**Question**	**Response**<br><br>**++/+/-/ NR/NA**	**Comments**
1.1	Is the source population or source area well described?<br><br>Was the country (e.g. developed or non-developed, type of health care system), setting (primary schools, community centres etc), location (urban, rural), population demographics etc adequately described?		
1.2	Is the eligible population or area representative of the source population or area?<br><br>Was the recruitment of individuals, clusters or areas well defined (e.g. advertisement, birth register)?<br><br>Was the eligible population representative of the source? Were important groups underrepresented?		
1.3	Do the selected participants or areas represent the eligible population or area?		

For peer review only

	Was the method of selection of participants from the eligible population well described?<br><br>What % of selected individuals or clusters agreed to participate? Were there any sources of bias?<br><br>Were the inclusion or exclusion criteria explicit and appropriate?		
**Section 2: Method of selection of exposure (or comparison) group**			
2.1	Selection of exposure (and comparison) group. How was selection bias minimised?<br><br>How was selection bias minimised?		
2.2	Was the selection of explanatory variables based on a sound theoretical basis?<br><br>How sound was the theoretical basis for selecting the explanatory variables?		
2.3	Was the contamination acceptably low?<br><br>Did any in the comparison group receive the exposure?<br><br>If so, was it sufficient to cause important bias?		
2.4	How well were likely confounding factors identified and controlled?<br><br>Were there likely to be other confounding factors not considered or appropriately adjusted for?		

For peer review only

	Was this sufficient to cause important bias?		
**Section 3: Outcomes**			
3.1	Were the outcome measures and procedures reliable?<br><br>Were outcome measures subjective or objective (e.g. biochemically validated nicotine levels ++ vs self-reported smoking −)?<br><br>How reliable were outcome measures (e.g. inter- or intra-rater reliability scores)?<br><br>Was there any indication that measures had been validated (e.g. validated against a gold standard measure or assessed for content validity)?		
3.2	Were the outcome measurements complete?<br><br>Were all or most of the study participants who met the defined study outcome definitions likely to have been identified?		
3.3	Were the outcome measurements complete?<br><br>Were all or most of the study participants who met the defined study outcome definitions likely to have been identified?		
3.4	Was there a similar follow-up time in exposure and comparison groups?		

For peer review only

	If groups are followed for different lengths of time, then more events are likely to occur in the group followed-up for longer distorting the comparison.<br>Analyses can be adjusted to allow for differences in length of follow-up (e.g. using person-years).		
3.5	Was follow-up time meaningful?<br>Was follow-up long enough to assess long-term benefits and harms?<br>Was it too long, e.g. participants lost to follow-up?		
**Section 4: Analyses**			
4.1	Was the study sufficiently powered to detect an intervention effect (if one exists)?<br>A power of 0.8 (i.e. it is likely to see an effect of a given size if one exists, 80% of the time) is the conventionally accepted standard.<br>Is a power calculation presented? If not, what is the expected effect size? Is the sample size adequate?		
4.2	Were multiple explanatory variables considered in the analyses?<br>Were there sufficient explanatory variables considered in the analysis?		
4.3	Were the analytical methods appropriate?<br>Were important differences in follow-up time and likely confounders adjusted for?		

For peer review only

4.4	Was the precision of association given or calculable? Is association meaningful?<br><br>Were confidence intervals or p values for effect estimates given or possible to calculate?<br><br>Were CIs wide or were they sufficiently precise to aid decision-making? If precision is lacking, is this because the study is under-powered?		
**Section 5: Summary**			
5.1	Are the study results internally valid (i.e. unbiased)?<br><br>How well did the study minimise sources of bias (i.e. adjusting for potential confounders)?<br><br>Were there significant flaws in the study design?		
5.2	Are the findings generalizable to the source population (i.e. externally valid)?<br><br>Are there sufficient details given about the study to determine if the findings are generalizable to the source population?<br><br>Consider: participants, interventions and comparisons, outcomes, resource and policy implications		

For peer review only

Summary of studies by country, country category, year of publication and focus

	Study	Country*	Category**	Year of publication	Focus of the study
1	Araujo et al[36]	Brazil	UMC	2017	Association between costs related to productivity loss and modified risk factors among uses of Brazilian National Health System
2	Cheah et al [37]	Malysia	UMC	2017a	Test the hypothesis “income is positively associated with the intensity of physical activity” by using data from a developing country(Malaysia)
3	Ding et al [38]	LMIC	LMIC	2016	Estimate direct health care cost, productivity losses and disability adjusted life years attributable to physical inactivity with standardized methods for 142 countries
4	Mitsunaga et al [39]	Brazil	UMC	2018	Assess the financial impact of a regular physical activity incentive program on medical hospital cost of private health care company
5	Turi et al [40]	Brazil	UMC	2015	Walking and health care expenditure among adults users of Brazilian health care system
6	Cheah et al [41]	Malaysia	UMC	2017b	Factors associated with participation decision and amount of participation among urban dwellers in Malaysia
7	Codongo et al[42]	Brazil	UMC	2015	Association between physical inactivity in different domains and direct public health care expenditure in adults
8	Zhang & Chaban [43]	China	UMC	2012	Total economic burden of physical inactivity in China

For peer review only

9	Turi et al [44]	Brazil	UMC	2017	Identify demographic, socioeconomic , life style and clinical factors associated with direct health care expenditure in primary care among adults through Brazilian health care system
10	Silva A.S [45]	Brazil	UMC	2015	Relationship between sufficient amount of physical activity among Brazilian adolescents and economic and social indicators of cities where they live
11	Patel et al [46]	South Africa	UMC	2011	Participation in fitness related activities of an incentive based health promotion programme and hospital costs
12	Bielmen et al[47]	Brazil	UMC	2014	Physical inactivity related inpatient costs of chronic non communicable diseases
13	Chen et al[48]	China	UMC	2015	Association of socioeconomic status with intensity of different types of physical activity in Chineese adults aiming to outline and projecting socioeconomic disparities among population undergoing rapid nutrient transition
14	Thiago et al[49]	Brazil	UMC	2015	Socioeconomic and regional difference in active transport in Brazil
15	Abdi et al [50]	Iran	UMC	2013	Compare the role of cost, time and attractiveness in predicting the participation level of student of Teheran Universities during their leisure time physical activities
16	Popkin et al[51]	China	UMC	2006	Case study from review of economic costs of diet, physical inactivity and obesity related chronic diseases to provide guidance to utilize the review for economic analysis of obesity
17	Atkinson et al[52]	LMIC	LMIC	2015	Association of occupational category and their physical inactivity and the association of human development, economic development, urbanization and physical inactivity

For peer review only

18	Queiroz et al [53]	Brazil	UMC	2020	Association between quality of life, physical activity, use of medication and costs of treatment for chronic diseases in Primary Care

For peer review only

Quality appraisal of included studies based on standard check lists

1. **Summary of quality appraisal by Larg & Moss Check list for cost of illness studies**

	Ding et al 2016 [38]	Zhang & Chaaban 2013 [43]	Bielemann et al 2015 [47]	Popikin et al 2006 [51]*
Q1(a)	++	++	+	+
Q1(b)	++	+	+	+
Q1(c) i	++	+	+	+
(c) ii	+	++	+	+
(c) iii	+	+	+	NR*
(c) iv	+(counterfactual population)	NA**	NA	NA
Q2(a) i	-	-	-	-
(a)ii	+	+	+	NR
(a)iii	+	+	++	NR
(a)iv	+	-	+	NR
(a)v	+	+	+	+
Q2(b)i	+	+	+	+
(b)ii	NA	NA	NA	NA
(b)iii	+	+	+	+
(b)iv	+	+	+	-
Q2(c)	+	NR(cost from national data)	NR	NR
Q2(d)	+	NR(cost from national data)	NA	NR
Q2(e)i	+	NR	NA	NR
(e)ii	++	NR	NA	NR
Q3 (a)	++	++	+	+
Q3(b)	++	-	-	-
Q3 (c)	+	NR	-	NR
Q3(d)i	+	Secondary data	-	NR
(d)ii	-	Secondary data	NR	NR
(d)iii	?	secondary data		NR

For peer review only

			+(CI not reported)NR	
Q3(e)	++	+	+	-
Q3(f)	++	+	-	NR
Q3(g)	++	+	+	+
Q3(h)	++	+	+	+

- Popkin et al case study developed from a review adequate information not available to comment on quality of costing study
- *NR- Not reported
- **NA-Not applicable

For peer review only

2. Summary of quality appraisal by NICE check list for correlations and associations

	Araujo et al 2017[36]	Cheah et al 2017a [37]	Mitsunaga 2018 [39]	Turi et al 2015 [40]	Cheah et al 2017b [41]	Codongo et al 2015 [42]	Turi et al 2017 [44]	Silva A.S 2015 [45]	Patel et al 2011 [46]	Thiago et al 2016 [49]	Abdi et al 2014 [50]	Chen et al 2015 [48]	Atkinson et al 2015 [52]
Q1.1	+	+	+	+	+	++	++	++	+	+	+	+	++
Q1.2	-	+	-	+	+	+	+	+	+	+	+	+	++
Q1.3	+	NR	+	+	+	+	+	+	+	+	-	+	++
Q2.1	NA	NA	+	NA	NA	NA	NA	NA	-	NA	NA	NA	NA
Q2.2	+	+	+	+	+	++	+	+	+	+	+	+	+
Q2.3	NA	NA	+	NA	NA	NA	NA	NA	+	NA	NA	NA	NA
Q2.4	+	+	-	+	+	++	+	-	-	-	-	-	+
Q2.5	This question not applicable for this review												
Q3.1	+	-	+	+	-	+	+	+	+	-	+	+	+
Q3.2	+	+	+	+	+	+	+	+	+	+	+	+	+
Q3.3	+	+	+	+	+	+	+	+	+	+	+	+	+
Q3.4	NA	NA	+	NA	NA	NA	NA	NA	+	NA	NA	NA	NA
Q3.5	NA	NA	+	NA	NA	NA	NA	NA	+	NA	NA	NA	NA
Q4.1*	+	+	-	+	+	+	+	+	+	-	+	+	+

Q4.2	+	+	-	+	+	++	+	-	+	-	-	+	+
Q4.3	+	+	+	+	+	++	+	+	+	-	-	+	+
Q4.4	+	+	-	+	+	++	+	+	+	-	-	+	+
Q5.1(IV)	+	+	++	++	++	++	++	++	++	++	+	++	++
Q5.2(EV)	++	++	++	++	++	++	++	++	++	+	+	++	++
Score	(13/13) (2/3),	(12/13) (2/3)	(9/13) (2/3)	(13/13) (3/3)	(12/13) (3/3)	(13/13) (3/3)	(13/13) (3/3)	(11/13) (3/3)	(11/13) (3/3)	(9/13) (3/3)	(9/13) (2/3)	(13/13) (3/3)	(13/13) (3/3)

*Most of the studies power calculations not done. However, large samples have been used

Quality score
Internal Validity >= 10/13=++ (>75%)
6-9/13=+ (50-75%)
0-5/13=- (<50%)

External Validity =3/3 (++),
= 2/3(+)
=≤1/3(-)

3. Summary of quality appraisal by Drummond and Jefferson 1996- check list for economic evaluation studies

	Queiroz et al 2020[53]
Study design	
Q1	+
Q2	+
Q3	+
Q4	+

	Queiroz et al 2020[53]
Q5	+
Q6	+
Q7	-
Data collection	
Q8	+
Q 9	-
Q 10	NA
Q11	+
Q12	+
Q13	+
Q14	NA
Q 15	NA
Q 16	NC
Q 17	-
Q 18	+
Q 19	-
Q 20	-
Q 21	-
Analysis and interpretation of results	
Q 22	+
Q 23	-
Q 24	-
Q 25	-
Q 26	NC
Q 27	-
Q 28	-
Q 29	-
Q 30	+
Q 31	+

For peer review only

	Queiroz et al 2020[53]
Q 32	NC
Q 33	+
Q 34	+
Q 35	+

Did the economic evaluation use a decision-analytic modelling framework?	No
Overall assessment by Drummond and Jefferson checklist	
Yes +<br>No -<br>Not clear NC<br>Not applicable NA	23 /35<br>(65.7%)

For peer review only

Reporting checklist for systematic review and meta-analysis.

Based on the PRISMA guidelines.

Instructions to authors

Complete this checklist by entering the page numbers from your manuscript where readers will find each of the items listed below.

Your article may not currently address all the items on the checklist. Please modify your text to include the missing information. If you are certain that an item does not apply, please write "n/a" and provide a short explanation.

Upload your completed checklist as an extra file when you submit to a journal.

In your methods section, say that you used the PRISMA reporting guidelines, and cite them as:

Moher D, Liberati A, Tetzlaff J, Altman DG, The PRISMA Group. Preferred Reporting Items for Systematic Reviews and Meta-Analyses: The PRISMA Statement

		Reporting Item	Page Number
**Title**			
	#1	Identify the report as a systematic review, meta-analysis, or both.	01
**Abstract**			
Structured summary	#2	Provide a structured summary including, as applicable: background; objectives; data sources; study eligibility criteria, participants, and interventions; study appraisal and synthesis methods; results; limitations; conclusions and implications of key findings; systematic review registration number	02
**Introduction**			
Rationale	#3	Describe the rationale for the review in the context of what is already known.	05/06

For peer review only

Objectives	#4	Provide an explicit statement of questions being addressed with reference to participants, interventions, comparisons, outcomes, and study design (PICOS).	07
**Methods**			
Protocol and registration	#5	Indicate if a review protocol exists, if and where it can be accessed (e.g., Web address) and, if available, provide registration information including the registration number.	03/07 Supplementary file 1
Eligibility criteria	#6	Specify study characteristics (e.g., PICOS, length of follow-up) and report characteristics (e.g., years considered, language, publication status) used as criteria for eligibility, giving rational	08
Information sources	#7	Describe all information sources in the search (e.g., databases with dates of coverage, contact with study authors to identify additional studies) and date last searched.	07
Search	#8	Present full electronic search strategy for at least one database, including any limits used, such that it could be repeated.	Supplementary file 2
Study selection	#9	State the process for selecting studies (i.e., for screening, for determining eligibility, for inclusion in the systematic review, and, if applicable, for inclusion in the meta-analysis).	08
Data collection process	#10	Describe the method of data extraction from reports (e.g., piloted forms, independently by two reviewers) and any processes for obtaining and confirming data from investigators.	09
Data items	#11	List and define all variables for which data were sought (e.g., PICOS, funding sources), and any assumptions and simplifications made.	9
Risk of bias in individual studies	#12	Describe methods used for assessing risk of bias in individual studies (including specification of whether this	9/10

For peer review only

		was done at the study or outcome level, or both), and how this information is to be used in any data synthesis.	
Summary measures	#13	State the principal summary measures (e.g., risk ratio, difference in means).	NA
Planned methods of analyis	#14	Describe the methods of handling data and combining results of studies, if done, including measures of consistency (e.g., I2) for each meta-analysis.	10
Risk of bias across studies	#15	Specify any assessment of risk of bias that may affect the cumulative evidence (e.g., publication bias, selective reporting within studies).	NA
Additional analyses	#16	Describe methods of additional analyses (e.g., sensitivity or subgroup analyses, meta-regression), if done, indicating which were pre-specified.	NA
**Results**			
Study selection	#17	Give numbers of studies screened, assessed for eligibility, and included in the review, with reasons for exclusions at each stage, ideally with a flow diagram.	11<br>Figure 1
Study characteristics	#18	For each study, present characteristics for which data were extracted (e.g., study size, PICOS, follow-up period) and provide the citation.	09/10<br>Table2
Risk of bias within studies	#19	Present data on risk of bias of each study and, if available, any outcome-level assessment (see Item 12).	13
Results of individual studies	#20	For all outcomes considered (benefits and harms), present, for each study: (a) simple summary data for each intervention group and (b) effect estimates and confidence intervals, ideally with a forest plot.	14-19
Synthesis of results	#21	Present the main results of the review. If meta-analyses are done, include for each, confidence intervals and measures of consistency.	Table 5
Risk of bias across studies	#22	Present results of any assessment of risk of bias across studies (see Item 15).	NA

For peer review only

Additional analysis	#23	Give results of additional analyses, if done (e.g., sensitivity or subgroup analyses, meta-regression [see Item 16]).	NA
**Discussion**			
Summary of Evidence	#24	Summarize the main findings, including the strength of evidence for each main outcome; consider their relevance to key groups (e.g., health care providers, users, and policy makers	19-22
Limitations	#25	Discuss limitations at study and outcome level (e.g., risk of bias), and at review level (e.g., incomplete retrieval of identified research, reporting bias).	22/23
Conclusions	#26	Provide a general interpretation of the results in the context of other evidence, and implications for future research.	23
**Funding**			
Funding	#27	Describe sources of funding or other support (e.g., supply of data) for the systematic review; role of funders for the systematic review.	23

None The PRISMA checklist is distributed under the terms of the Creative Commons Attribution License CC-BY. This checklist can be completed online using https://www.goodreports.org/, a tool made by the EQUATOR Network in collaboration with Penelope.ai

For peer review only
